# Slow Release of Bioactive Alcohols From Butenolide Polymers and Coatings

**DOI:** 10.1002/chem.70951

**Published:** 2026-03-28

**Authors:** Andries Jensma, Jesk T. Jaeger, J. George H. Hermens, Thomas Freese, Jelmer T. Meijer, Rafael Tarozo, Jan L. Sneep, Niels Elders, Keimpe van den Berg, Ben L. Feringa

**Affiliations:** ^1^ Stratingh Institute for Chemistry Advanced Research Center Chemical Building Blocks Consortium (ARC CBBC) University of Groningen Groningen The Netherlands; ^2^ Department Resin Technology Akzo Nobel Car Refinishes BV Sassenheim The Netherlands; ^3^ R&D Department Circolide Circolide Technologies B.V. Groningen The Netherlands

**Keywords:** polymers, sustainable chemistry, post‐polymerization modification

## Abstract

Over the past decades, an increased interest is observed for sustainable building blocks for polymers and the wide range of omnipresent plastic products. Concurrently, polymer‐based controlled release systems have gained significant attention from the fragrance, agricultural, and pharma industries for release of bioactive compounds, odors, and pesticides. Here, we report the controlled release of bioactive alcohols from biobased alkoxy butenolide derived polymers and coatings, without making use of post‐polymerization modifications. Utilizing the acetal functionality of the butenolide scaffold, bioactive alcohols can be linked to the butenolide monomer and selectively released from the produced polymer using mild acidic conditions (pH ≤ 7). The alcohol release rate can be tuned by altering the polymer polarity by the choice of the monomers, higher acid catalyst loadings and longer exposure time.

## Graphical abstract

In this study we have reported the acid‐mediated release of bioactive alcohols from biobased alkoxy butenolide derived copolymers and coatings. It can potentially be applied for slow release of odors, pesticides, or drug‐related compounds from biobased polybutenolides.

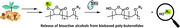

## 1 Introduction

Controlled release of bioactive compounds such as odors, pesticides, and drugs has gained significant attention in recent years [1, 2, 3]. Inducing a controlled release of different moieties is typically achieved by encapsulating the target molecule in a micro‐ or nano capsule and releasing it slowly through diffusion [4]. However, this requires complex syntheses of capsules and causes microplastic pollution [5].

The growing demand for functional biobased macromolecules has led to increased efforts toward development of high‐performance, sustainable polymers. Of particular interest are macromolecules that can take advantage of post‐polymerization reactivity for controlled release of bioactive compounds. This could be utilized, among others, in the field of medicinal chemistry for controlled delivery of drugs [1, 2, 3], in the agricultural industries for controlled release of pesticides and in the fragrance industry for controlled release of aroma compounds. Controlled release from macromolecular scaffolds holds advantages over diffusion‐based controlled release methods, as higher payload loadings [6, 7, 8, 9] and external stimuli control [10] can be achieved. After attaching a suitable payload on the polymeric backbone, payload release can be triggered via several stimuli such as temperature, oxygen, light, enzymes, microorganisms, or hydrolysis [1]. A key stimuli is hydrolysis, as it is the preferred methodology in the field of medicinal chemistry. Here, the payload is covalently attached to the polymeric backbone, using acid labile groups such as acetals [11, 12, 13, 14, 15], esters [16, 17], or imines [18]. The rate of release is directly related to the cleavage of the conjugate linkage, which is influenced by pH, steric, electronic, and polar effects. The amount of payload that is released is predetermined by the amount of payload linkages attached to the polymer. The application in the fields of medicinal and fragrance industry shows the importance of stimuli‐responsive materials, via the incorporation of labile bonds in polymer chains.

**SCHEME 1:**
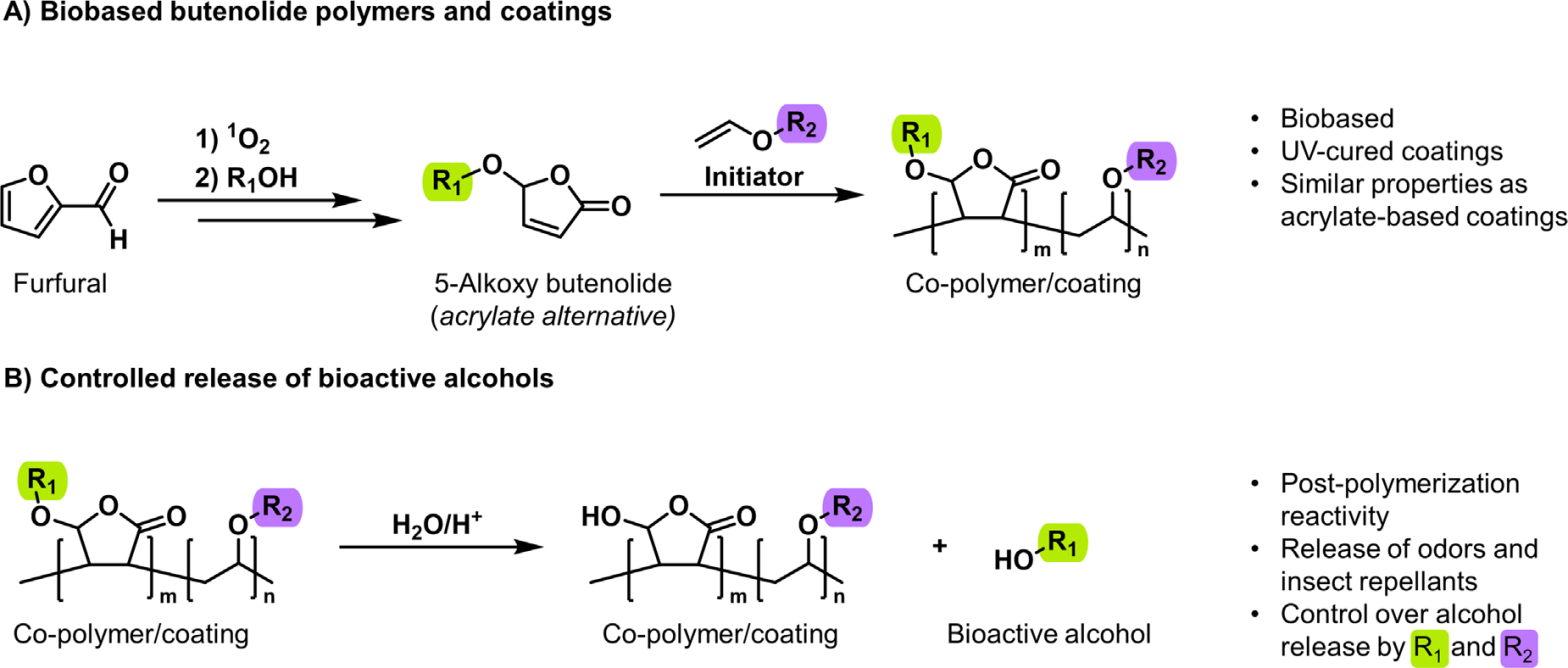
(A) Biobased copolymers and coatings based on alkoxy butenolides using photooxidation, condensation, and radical copolymerization with electron‐rich vinyl comonomers. (B) Controlled release of bioactive alcohols from biobased butenolide copolymers and coatings using acid‐catalyzed hydrolysis.

Previously, our group introduced butenolides as biobased alternative for conventional acrylates (Scheme 1A) [19, 20, 21]. Starting from xylan‐rich lignocellulosic biomass, furfural is produced using acid‐catalyzed hydrolysis [22, 23, 24, 25]. This platform chemical is then quantitatively converted into hydroxy butenolide using a [4+2] cycloaddition with photochemically generated singlet oxygen [26, 27, 28, 29]. Subsequently, hydroxy butenolide is converted into alkoxy butenolides [19] or acyloxy butenolides [30, 31, 32, 33], which can be utilized as comonomers in free radical polymerization reactions using solventborne conditions [19, 20, 30, 31, 34], waterborne conditions [35], and solvent free conditions [19, 21, 30, 32, 33, 34]. Typically, copolymerizations with electron‐rich vinyl comonomers, such as vinyl ethers, vinyl esters, and vinyl lactams, are performed to produce biobased copolymers for coating applications which demonstrated properties similar to the polyacrylate‐based references [19].

When investigating the structure of our butenolide based polymers and coatings, we were intrigued by the acetal functionality present in the monomer and polymer. Since acetals are potential reactive groups that allows for post‐polymerization reactivity, we hypothesized that we can utilize the polymers and coatings for mild and selective alcohol release (Scheme 1B). The butenolide based polymers could be suitable candidates for release of e.g., odors, drugs, and insect repellents. In this work, we present our first endeavors investigating the hydrolysis of the acetal functional group on butenolide monomers, polymers, and coatings by using an external stimulus, in this case pH.

## 2 Results and Discussion

**FIGURE 1:**
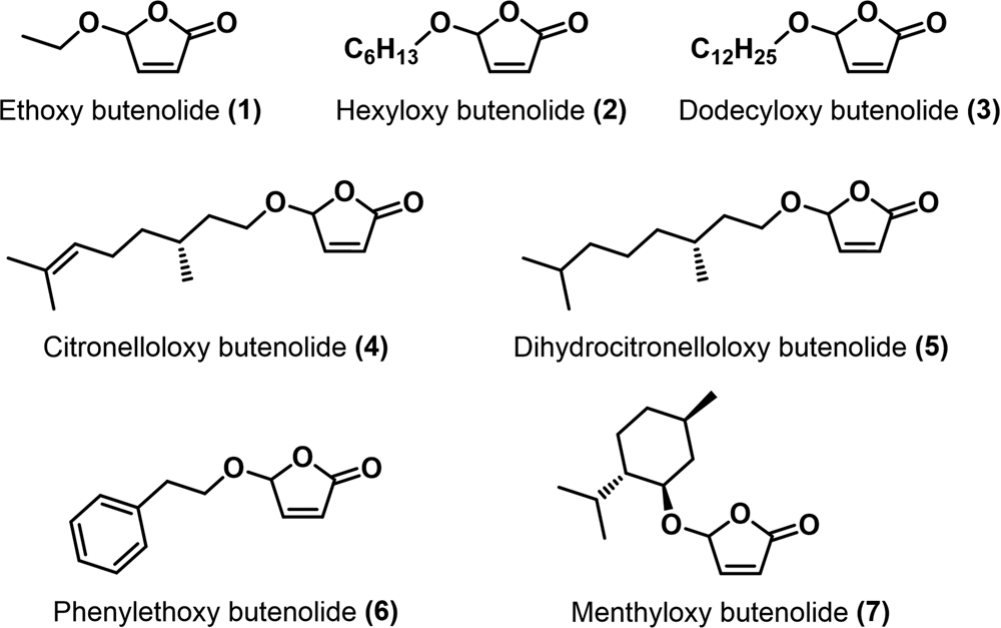
Overview of synthesized alkoxy butenolides [30].

The biobased monomers used in this study, that is, alkoxy butenolides, are readily synthesized in two steps starting from furfural. First, furfural is converted into hydroxy butenolide using a photooxidation reaction in our photoflow reactor [28, 29]. In the second step, hydroxy butenolide is converted into the alkoxy butenolide via a condensation reaction with the corresponding alcohol. This procedure has been recently optimized through the use of a solid acid catalyst, resulting in enhanced purification efficiency [36]. Using this method we obtain the alkoxy butenolides in relatively good yields of 60% to 95%, depending on the attached alcohol. In the present study we targeted several butenolides for potential release of odors (hexanol, citronellol, dihydrocitronellol, phenylethanol, and menthol) and insect repellants (dodecanol and menthol) (Figure 1, for synthesis and characterization see Supporting Information).

**FIGURE 2:**
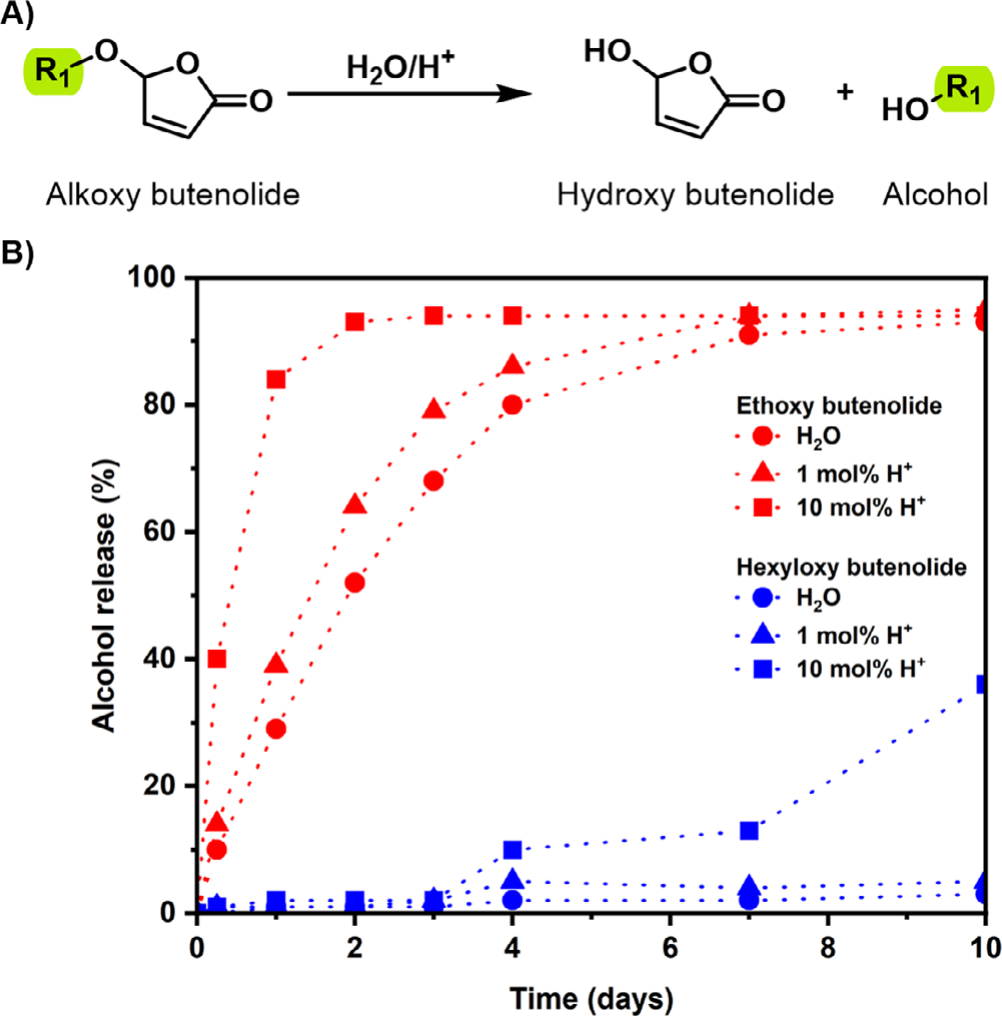
(A) General reaction scheme of the acid‐mediated release of alkoxy butenolide monomers (**1** and **2**). (B) Quantified acid mediated alcohol (ethanol and hexanol) release of ethoxy‐ (red) and hexyloxy butenolide (blue) for 10 d determined by ^1^H‐NMR spectroscopy (in DMSO‐d_6_) in water (circle), 1 mol% HCl vs. butenolide in water (triangle), and 10 mol% HCl versu. butenolide in water (square).

With the butenolide monomers in hand, we first investigated alcohol release properties of the monomers itself. Two alkoxy butenolides, ethoxy‐ and hexyloxy butenolide, were selected and the hydrolysis was studied in aqueous media with different acidities (Figure 2A). The stability of the monomers was followed over time by taking aliquots and measuring by ^1^H‐NMR in deuterated DMSO (Figure 2B). The amount of released alcohol was calculated through the ratio between the integrals of the alkoxy butenolide and the hydroxy butenolide signals in the ^1^H‐NMR spectrum. Gratifyingly, we observed hydrolysis of the acetal moiety in ethoxy butenolide (in red) in neutral aqueous solutions. In all three cases, that is, water (pH = 6.8), 1 mol% HCl compared to butenolide (pH = 2) and 10 mol% HCl compared to butenolide (pH = 1), for ethoxy butenolide, we observed a conversion of approximately 95% after 10 d. For the more hydrophobic hexyloxy butenolide (in blue) conversions of 5% to 10% after 10 d in water and 1 mol% HCl were observed. An increase in hydrolysis was found at increased acidity (10 mol% HCl) to around 40%. Hexyloxy butenolide is less soluble in water compared to ethoxy butenolide and therefore interaction with water (i.e., hydrolysis) is reduced. Interestingly, an effect was only observed at high acid concentrations (i.e., 10 mol% H+ compared to the butenolide) in both ethoxy‐ and hexyloxy butenolide. With the monomer hydrolysis experiments we show the potential for slow release of bioactive alcohols from our butenolide polymers and coatings.

Before evaluating the alcohol release properties of butenolide polymers, we decided to first investigate the copolymerization reaction using our novel monomers. Alkoxy butenolides were copolymerized with vinyl ethers under previously described solventborne conditions [19, 20, 30, 31, 34]. Using trigonox‐42S (*t*‐butyl peroxy‐3,5,5‐trimethylhexanoate) as radical initiator (t_1/2_ = 1 h at 114°C) and *n*‐butyl acetate as solvent, a selection of alkoxy butenolides were copolymerized with the hydrophobic dodecyl vinyl ether (DVE) and the hydrophilic ethylene glycol vinyl ether (EGVE). In order to quantify the release of the copolymers, it is crucial that all alkoxy butenolide monomer is fully incorporated into the copolymer as the monomer itself can hydrolyze (vide supra). Therefore, the copolymerization reactions were followed over time by ^1^H‐NMR using 1,3,5‐trimethoxybenzene as internal standard. Generally, copolymerizations with both DVE and EGVE provide full conversion of the alkoxy butenolide (Table 1). For the copolymerization with DVE a combined monomer conversion of around 80%–90% was observed, due to a slight mismatch in compatibility between DVE and the alkoxy butenolide. Typically, the conversion of DVE stops at 80% to 85% after 2 h. Changing to the more polar EGVE we observed near quantitative conversion for both alkoxy butenolides and the OH‐functionalized comonomer. In addition, the polymerization rate is increased by replacing DVE for EGVE. From the above observations it is evident that a better match exists in reactivity between EGVE and an alkoxy butenolide, which is possibly due to comparable polarity of both monomers versus DVE. Nearly all alkoxy butenolides demonstrated quantitative conversions, only citronelloxy butenolide (Entry 4, Table 1, both DVE and EGVE) showed lower conversions. This is probably due to the radical scavenging ability of the double bond of the attached alcohol. As expected, changing the substituent from citronellol to dihydrocitronellol (i.e., without an olefinic double bond) (5) resulted in full conversion of the butenolide monomer during radical copolymerization.

**TABLE 1:** Comparison of the monomer conversions, initial copolymerization rates and molecular weight distribution for equimolar mixtures of alkoxy butenolide and dodecyl vinyl ether (DVE, R = C_12_H_25_) or ethylene glycol vinyl ether (EGVE, R = C_2_H_4_OH) in *n*‐butyl acetate (AcOBu) at 120°C, initiated by the addition of trigonox 42S (3 mol % vs. total monomer content).^a^

Entries	Comonomer	Alkoxy butenolide	Conversion butenolide (%)	Conversion vinyl ether (%)	*K*_ini_ [10*^−^* ^4^ s^−1^]	*M*_n_ (kDa)	*Đ*	DP	Tg (°C)
1	DVE	Ethoxy‐	>99	76	7.85	1.4	2.3	8	−12
	EGVE		>99	>99	16.9	3.3	2.7	52	75
2	DVE	Hexyloxy‐	>99	74	6.53	1.9	2.3	8	−53
	EGVE		>99	98	16.3	9.3	2.2	48	33
3	DVE	Dodecyloxy‐	>99	85	8.47	1.9	2.3	8	n/a
	EGVE		>99	99	14.8	9.8	2.1	44	32
4	DVE	Citronelloxy‐	69	53	2.97	1.0	5.7	7	−34
	EGVE		92	94	4.35	4.7	3.4	21	25
5	DVE	Dihydrocitronelloxy‐	>99	71	9.15	1.4	3.3	6	−39
	EGVE		>99	>99	32.1	6.9	2.5	38	9
6	DVE	Phenylethoxy‐	>99	75	8.2	1.5	2.1	7	−32
	EGVE		>99	>99	22.6	6.0	3.8	28	40
7	DVE	Menthyloxy‐	>99	75	7.55	1.6	2.2	6	−2
	EGVE		>99	>99	12.1	2.6	2.2	23	11

^a^ *k*_ini_ = initial observed pseudo‐first‐order copolymerization rate; *M*_n_ = number‐average molecular weight; *Đ* = polydispersity index; DP = n + p, average degree of polymerization; *T*_g_ = glass transition temperature.

The synthesized copolymers were characterized using gel permeation chromatography (GPC) in THF (for DVE copolymers) or DMF (for EGVE copolymers) to determine the molecular weight (MW), polydispersity index (PDI) and the average degree of polymerization (DP), and the data are shown in Table 1. For all the copolymers with DVE a M_n_ between 1 and 2 KDa was found, with the exception of the one with citronelloloxy butenolide (4‐DVE). This copolymer has a lower M_n_ caused by the poor copolymerization reactivity. When switching from DVE to EGVE we observed a clear trend, as higher MWs and average DPs are observed. This is probably due to the fact that EGVE has a better reactivity match with the alkoxy butenolides resulting in higher degrees of polymerization. Furthermore, the copolymers were also characterized using differential scanning calorimetry (DSC) to determine the glass transition temperatures (Tg). Here, we observed that DVE copolymers have lower Tg compared to the EGVE copolymers in a similar trend as we observed in previous work [19, 30]. This can be attributed to the relatively low DP and the flexible alkyl chain of DVE. A similar trend is observed between the R groups of the alkoxy butenolide in both DVE and EGVE co‐polymers, in which a longer, flexible side chain leads to a lower Tg.

Having investigated the copolymerization reaction and the resulting polymer properties, we continued with the acid mediated release of the butenolide copolymers (Figure 4A). Similar to the monomers, a sample was prepared consisting of alkoxy butenolide copolymer and an aqueous solution (water, 10, and 100 mol% HCl) followed by shaking (using an orbital shaker) at room temperature. All release experiments were performed in sealed 20 mL headspace vials under orbital shaking (400 rpm) at room temperature. This method was chosen over magnetic stirring to handle viscous copolymers, maintain uniform agitation across parallel vials, and prevent bar–polymer contact. Unfortunately, hydrolysis could not be followed using ^1^H‐NMR due to overlap between the signals of the released alcohol and the polymer. As an alternative, we opted for gas chromatography mass spectrometry (GC–MS). However, this technique proved to be inconsistent due to tailing on the column, presumably by accumulating of polymer in the injection liner and/or column (see Supporting Information for extended discussion). By using a modified technique, headspace GC–MS, in which a sample is taken from the headspace (gas phase) of the vial containing the polymer/water mixture, the nonvolatile polymer is excluded from the analysis and we could successfully measure and quantify the release of volatile alcohols. Initially, the release was tested at a GC–MS oven temperature of 100°C, but this had a significant influence on the amount of released payload giving inconsistent results. Lowering the GC–MS oven temperature to 30°C led to more consistent results with the exception for the less volatile alcohols which could not be measured and thus required higher oven temperatures. As the alcohol in the headspace is in equilibrium with the alcohol in solution, calibration curves were made by measuring known amounts of alcohol/water mixtures. By integration of the area of the alcohol peak in the headspace and comparing with the calibration curve, the amount of alcohol (in moles) in the whole system can be calculated.

**FIGURE 3:**
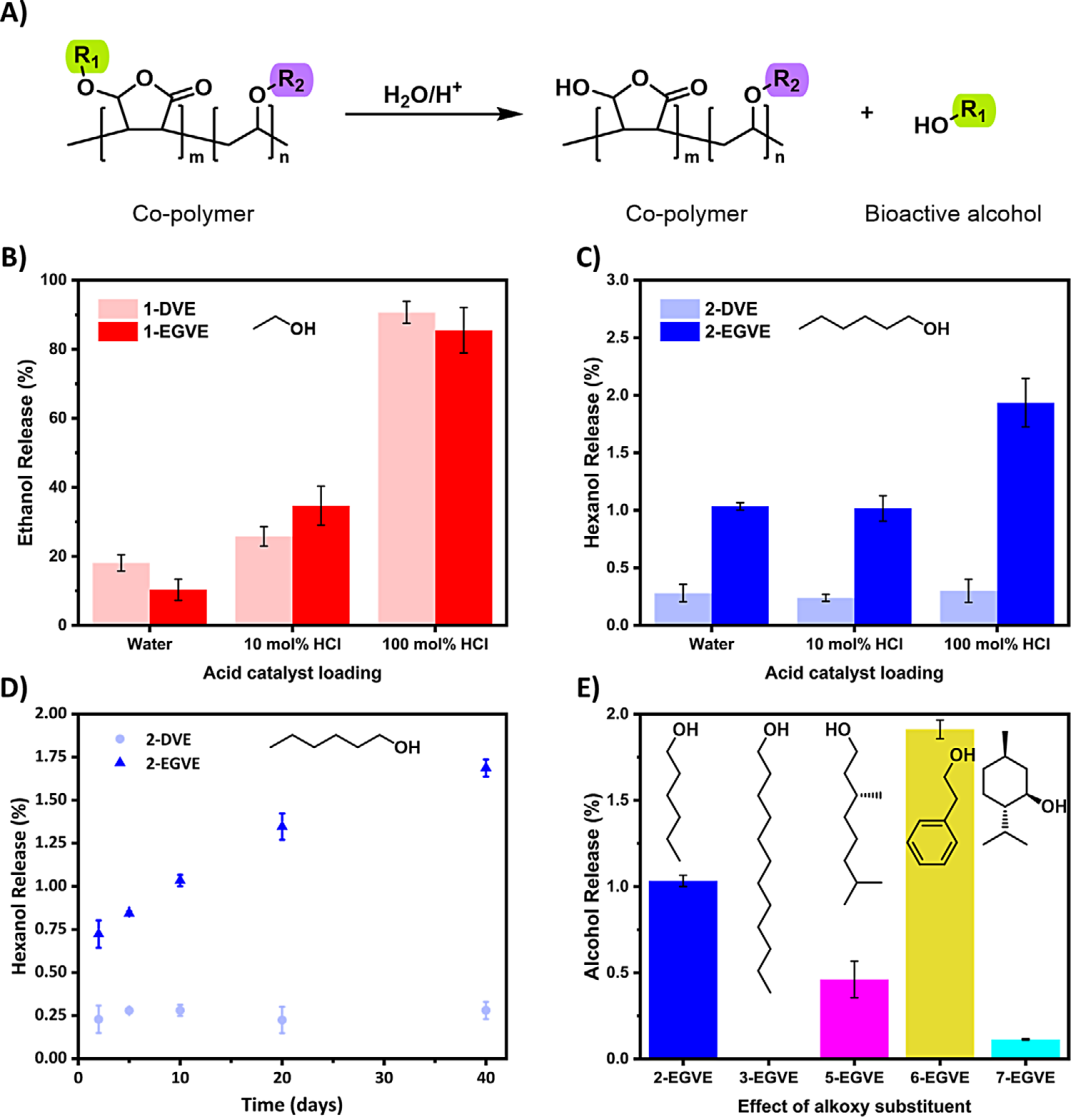
(A) Release of alcohols from alkoxy butenolide copolymers in aqueous media measured with headspace GC‐MS. (B) Release of ethanol from ethoxy butenolide copolymers with dodecyl vinyl ether (DVE, light red) and ethylene glycol vinyl ether (EGVE, red) measured after 10 d in water, HCl solution with two acid catalyst loadings (10 and 100 mol% compared to butenolide) (triplicate measurements). (C) Release of hexanol from hexyloxy butenolide copolymers with dodecyl vinyl ether (DVE, light blue) and ethylene glycol vinyl ether (EGVE, blue) measured after 10 d in water, HCl solution with two acid catalyst loadings (10 and 100 mol% compared to butenolide) (triplicate measurements). (D) Release of hexanol from hexyloxy butenolide copolymer with dodecyl vinyl ether (DVE, light blue) and ethylene glycol vinyl ether (EGVE, blue) in water followed over time (triplicate measurements). (E) Release after 10 d in water of hexanol (2‐EGVE, blue), dodecanol (3‐EGVE, colorless), dihydrocitronellol (5‐EGVE, magenta), phenylethanol (6‐EGVE, yellow), and menthol (7‐EGVE, cyan) from the corresponding copolymers with EGVE (triplicate measurements).

After developing a suitable method for quantifying the alcohol release of polymers, the release of ethanol and hexanol from the corresponding copolymers with DVE and EGVE was studied (Figure 3B). First, the effect of pH on copolymer release was investigated by preparing samples with three different acid concentrations (H_2_O, 10, and 100 mol% compared to butenolide units) through the addition of HCl in water. Alcohol release was then measured in triplicate after 10 d (Figure 3A). Initially, ethoxy butenolide copolymers (1‐DVE and 1‐EGVE) were investigated as depicted in Figure 3B. Relatively slow release (up to 20%) takes place in neutral water (pH = 6.8). Similarly to the ethoxy butenolide monomer hydrolysis, release of ethanol from the copolymer is increased at reduced pH. Switching from neutral water to an aqueous solution with 10 mol% HCl compared to butenolide units (pH = 1), we observed a small increase in release from 20% to 25% for the DVE copolymer. Only when equimolar amounts of HCl (100 mol% compared to butenolide units, pH = 0) were used a significant increase could be observed for ethoxy butenolide‐DVE copolymer. A similar trend was observed for the copolymer with EGVE as comonomer. Interestingly, no significant effect was observed when switching to the more hydrophilic copolymer with EGVE, compared to the copolymer with DVE.

A similar study using hexyloxy butenolide copolymers (2‐DVE and 2‐EGVE) was performed, as can be seen in Figure 3C. For 2‐DVE, a release of 0.2%–0.3% after 10 d was observed, in all three aqueous media. Here, the apolar hexyl chain of the butenolide backbone and the dodecyl chain of the vinyl ether backbone prohibit interactions with water and thus prevent hydrolysis (that is, hydrophobic shielding). As anticipated, increased hexanol release from 2‐EGVE was observed due to an increased polymer hydrophilicity. Similar to the monomer release studies, we observed significant increase in release (from 1% to 2%) with equimolar amounts of HCl compared to butenolide units.

While the focus lied upon the release of alcohols from the alkoxy butenolide unit in the copolymer, we were also interested in the hydrolysis of the ether from DVE and EGVE. Here, we did not observe any signals of 1‐dodecanol or ethylene glycol in the headspace GC‐MS chromatograms. There are two possible explanations for the absence of detectable signals in this context. First, hydrolysis of the ether may not be occurring under the experimental conditions employed. Alternatively, the hydrolysis does take place but cannot be observed due to the relatively low volatility of 1‐dodecanol and ethylene glycol.

The release of hexyloxy copolymers with both DVE and EGVE in water were also followed over time (Figure 3D). In the case of 2‐DVE copolymer, remarkably, there was no measured increase in released hexanol over time. Apparently, 0.25% released hexanol is maximum under the selected experimental conditions. Switching to the more hydrophilic EGVE copolymer, a gradual increase in hexanol is observed resulting in 1.75% over 40 d. This means that by carefully choosing the comonomers we should be able to control alcohol release like an on/off switch in our copolymers due to changes in polarity.

With the knowledge gained from release of the ethoxy‐ and hexyloxy butenolide copolymers, we targeted different alkoxy butenolides with possible release of functional alcohols. Here, we used EGVE copolymers with hexanol (2‐EGVE, blue, and odor), dodecanol (3‐EGVE, no color, and insect repellant), dihydrocitronellol (5‐EGVE, magenta, and odor), phenylethanol (6‐EGVE, yellow, and odor), and menthol (7‐EGVE, cyan, odor, and antibacterial). Copolymers with EGVE were chosen as they trigger alcohol release more easily since these copolymers are more polar. Samples (in triplicate) were prepared in water and measured after 10 d shaking using the orbital shaker. As can be seen in Figure 3E, the amount of hydrolysis is dependent on the alkoxy substituent. Consistent with our previous results (vide supra) is that a longer alkyl chain generally leads to a lower amount of release (hydrophobicity). Consequently, the amount of released 1‐dodecanol from dodecyloxy butenolide‐co‐EGVE copolymer (3‐EGVE) could not be quantified using headspace GC‐MS. While the chromatogram showed release of dodecanol, it was not possible to quantify using this method possibly due to its relatively low volatility compared to other alcohols used. Interestingly, the copolymer with dihydrocitronellol (5‐EGVE) showed around 0.46% release after 10 d. This corroborates with our findings with hexanol and dodecanol as this alcohol has an alkyl chain length of 8 carbons. The copolymer with 2‐phenylethanol (6‐EGVE) is slightly more polar due to its aromatic ring, leading to slightly increased hydrolysis rate (1.9% after 10 d). Interestingly, for 7‐EGVE only approximately 0.1% of menthol is released after 10 d, probably due to the rigid structure of the copolymer. With the secondary alcohol (menthol) attached, the copolymer exhibits a high hardness. This, in collaboration with steric hindrance, could make it more difficult for water to penetrate and reach the acetal moiety for hydrolysis. These findings show that by selectively choosing the attached alcohol (and thus the released alcohol), in combination with the selected comonomer, we can precisely control the release rate.

**FIGURE 4:**
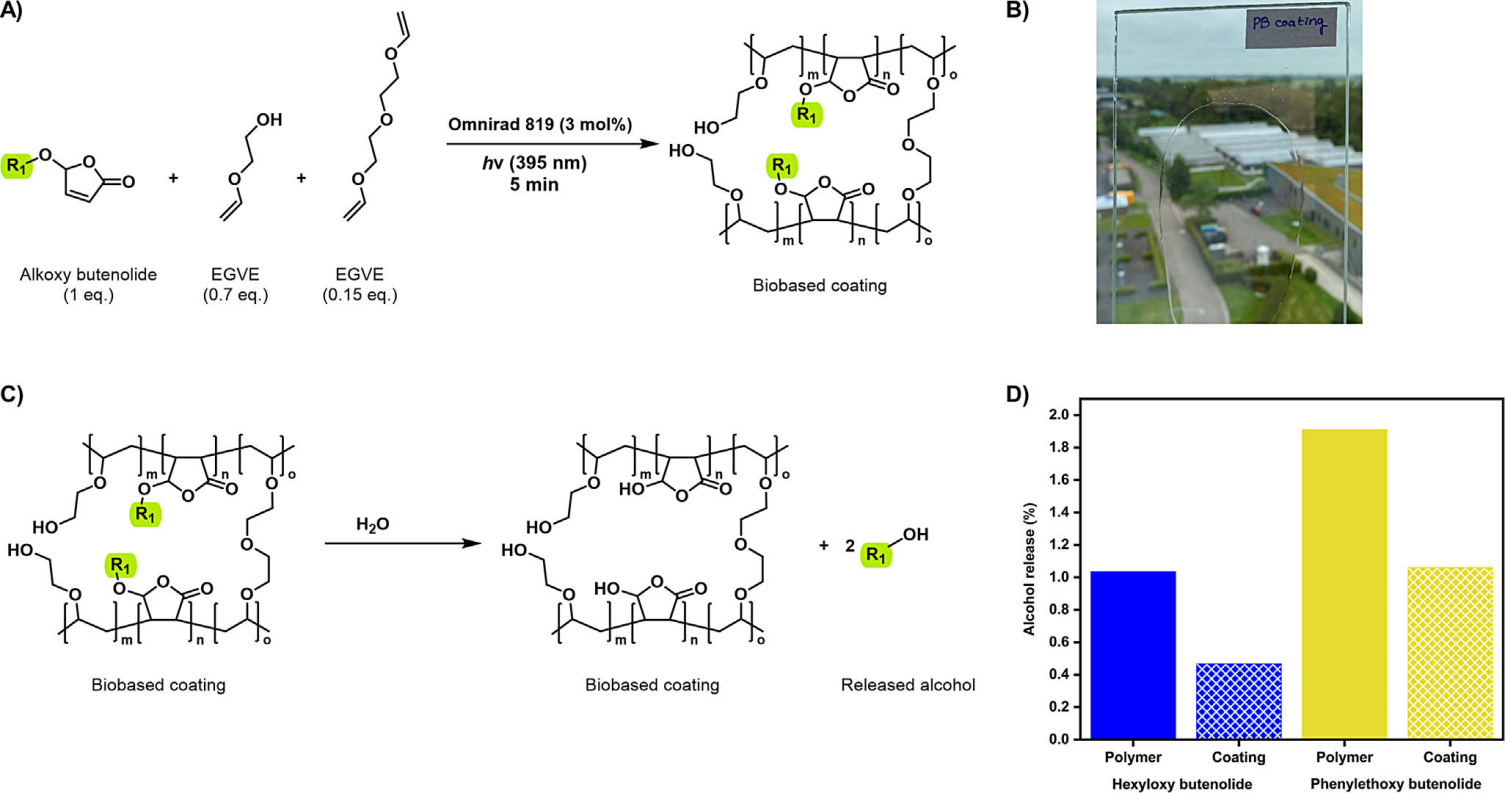
(A) Preparation of biobased coatings with alkoxy butenolide (1 eq.), ethylene glycol vinyl ether (EGVE, 0.7 eq.), and diethylene glycol divinyl ether (DEGDVE, 0.15 eq.). (B) Clear, hard coating based on phenyl ethoxy butenolide. (C) Slow release of bioactive alcohol from butenolide‐based coating in H_2_O. (D) Release of hexanol (blue) and phenylethanol (yellow) from the corresponding polymers (left) and coatings (right) after 10 d shaking in H_2_O.

With the slow release from linear butenolide polymers established, we switched to a cross‐linked polymeric system (Figure 3). Biobased butenolide coatings were prepared using UV curing under solvent‐free conditions using Omnirad 819 (3 mol% compared to all monomer) as photoinitiator [19]. For these coatings, a monomer mixture of alkoxy butenolide (1 eq.), EGVE (0.7 eq.), and diethylene glycol divinyl ether (DEGDVE, 0.15 eq.) as cross‐linker was used to ensure a 1:1 ratio of butenolide double bond and vinyl ether double bond. Clear, hard and uniform coatings with a thickness of 100 µm were obtained by applying the reaction mixture on a glass surface using a Byk applicator and applying UV light (395 nm) for 5 min (Figure 4A,B). The coatings were removed from the glass substrate and put into a headspace GC–MS vial for release studies (Figure 4C). After shaking for 10 d in water, the respective alcohol release was determined. Interestingly, less hexanol was released from the cross‐linked hexyloxy butenolide coating (0.47%) compared to the corresponding linear copolymer using EGVE (1.03%, Figure 3E). The odor of the 1‐hexanol was perceptible upon smelling the released coating, indicating that the controlled release of fragrance exceeded the odor detection threshold (ODT) of this compound (8 ppb). This was further confirmed by headspace GC analysis, which quantified the concentration at 113 ppm (See Supporting Information for extended discussion). A similar effect was observed for the phenylethoxy butenolide coating, where 1.06% phenylethanol was released after 10 d from the cross‐linked network compared to 1.91% phenylethanol from the thermoplastic copolymer (Figure 3E). This effect can be attributed to the cross‐linked network making the polymer more rigid and thus more difficult for water to migrate through the polymer matrix and onto the acetal group of the butenolide. It should be noted that hexyloxy butenolide coatings demonstrated excellent water resistance and no visual damage of the coating surface could be seen. This corroborates that the coatings can be used for slow payload release while keeping its decorative and protective properties intact.

## 3 Conclusion

In conclusion, we have demonstrated that our biobased butenolide polymers and coatings can be used for slow release of a payload. Taking advantage of the acetal functionality of the butenolide backbone, slow release of bioactive alcohols under neutral aqueous conditions was observed. Enhanced release was found under acidic conditions. By switching comonomers from the hydrophilic EGVE to the hydrophobic DVE the release of alcohols from the copolymer could be limited. By changing the monomer composition, we can selectively target a certain amount of alcohol release. Furthermore, it is shown that release of bioactive alcohols could be precisely tuned with applications for odor release or release of an antibacterial agent. Finally, slow release of bioactive alcohols was shown from biobased butenolide cross‐linked coatings while keeping its protective abilities intact. Based on these findings, we envision polybutenolides potential applications for controlled slow release of odors, pesticides, and possibly drugs which would demonstrate an added benefit of our bio‐based polybutenolide technology over petrochemical polyacrylates.

## Conflicts of Interest

The authors declare no conflicts of interest.

## Supporting information




**Supporting File**: The file contains experimental details, materials, and methods; ^1^H, ^13^C, COSY, and HSQC NMR spectra for all novel compounds; copolymerization data; differential scanning calorimetry (DSC) and gel permeation chromatography (GPC) analysis of polymers; and headspace GC‐MS data of alcohol release. Additional references cited within the Supporting Information [37, 38, 39, 40, 41, 42].

## Data Availability

The data that supports the findings of this study are available in the Supporting Information of this article.

## References

[1] A. Herrmann , “Controlled Release of Volatiles Under Mild Reaction Conditions: From Nature to Everyday Products,” Angewandte Chemie International Edition 46 (2007): 5836–5863, 10.1002/anie.200700264. 17605134

[2] N. Kamaly , B. Yameen , J. Wu , and O. C. Farokhzad , “Degradable Controlled‐Release Polymers and Polymeric Nanoparticles: Mechanisms of Controlling Drug Release,” Chemical Reviews 116, no. 4 (2016): 2602–2663, 10.1021/acs.chemrev.5b00346. 26854975 PMC5509216

[3] M. A. Beach , U. Nayanathara , Y. Gao , et al., “Polymeric Nanoparticles for Drug Delivery,” Chemical Reviews 124, no. 9 (2024): 5505–5616, 10.1021/acs.chemrev.3c00705. 38626459 PMC11086401

[4] S. Ghayempour and M. Montazer , “Micro/Nanoencapsulation of Essential Oils and Fragrances: Focus on Perfumed, Antimicrobial, Mosquito‐Repellent and Medical Textiles,” Journal of Microencapsulation 33 (2016): 497–510, 10.1080/02652048.2016.1216187. 27701985

[5] J. Boucher and D. Friot , Primary Microplastics in the Oceans: A Global Evaluation of Sources (IUCN 2017), 10.2305/IUCN.CH.2017.01.en.

[6] G. S. Banker , Medical Applications of Controlled Release (CRC Press, 2019): 1–34, 10.1201/9780429276620.

[7] K. J. Watson , D. R. Anderson , and S. T. Nguyen , “Toward Polymeric Anticancer Drug Cocktails From Ring‐Opening Metathesis Polymerization,” Macromolecules 34 (2001): 3507–3509, 10.1021/ma001916t.

[8] P. A. Bertin , K. J. Watson , and S. T. Nguyen , “Indomethacin‐Containing Nanoparticles Derived From Amphiphilic Polynorbornene: A Model ROMP‐Based Drug Encapsulation System,” Macromolecules 37 (2004): 8364–8372, 10.1021/ma035741+.

[9] P. A. Bertin , D. Smith , and S. T. Nguyen , “High‐Density Doxorubicin‐Conjugated Polymeric Nanoparticles Via Ring‐Opening Metathesis Polymerization,” Chemical Communications 30 (2005): 3793–3795, 10.1039/b504643b. 16041419

[10] X. Pang , Y. Jiang , Q. Xiao , A. W. Leung , H. Hua , and C. Xu , “pH‐Responsive Polymer–Drug Conjugates: Design and Progress,” Journal of Controlled Release 222 (2016): 116–129, 10.1016/j.jconrel.2015.12.024. 26704934

[11] Y. Gu , Y. Zhong , F. Meng , R. Cheng , C. Deng , and Z. Zhong , “Acetal‐Linked Paclitaxel Prodrug Micellar Nanoparticles as a Versatile and Potent Platform for Cancer Therapy,” Biomacromolecules 14 (2013): 2772–2780, 10.1021/bm400615n. 23777504

[12] D. Huang , Y. Zhuang , H. Shen , F. Yang , X. Wang , and D. Wu , “Acetal‐Linked PEGylated Paclitaxel Prodrugs Forming Free‐Paclitaxel‐Loaded pH‐Responsive Micelles With High Drug Loading Capacity and Improved Drug Delivery,” Materials Science and Engineering: C 82 (2018): 60–68, 10.1016/j.msec.2017.08.063. 29025675

[13] J. Zou , G. Jafr , E. Themistou , et al., “pH‐Sensitive Brush Polymer‐Drug Conjugates by Ring‐Opening Metathesis Copolymerization,” Chemical Communications 47 (2011): 4493–4495, 10.1039/c0cc05531j. 21399797

[14] J. Zou , Y. Yu , Y. Li , et al., “Well‐Defined Diblock Brush Polymer–Drug Conjugates for Sustained Delivery of Paclitaxel,” Biomaterials Science 3 (2015): 1078–1084, 10.1039/C4BM00458B. 26221941

[15] Y. Yu , C.‐K. Chen , W.‐C. Law , et al., “Well‐Defined Degradable Brush Polymer–Drug Conjugates for Sustained Delivery of Paclitaxel,” Molecular Pharmaceutics 10 (2013): 867–874, 10.1021/mp3004868. 23181264

[16] S. Zhang , J. Zou , M. Elsabahy , et al., “Poly(ethylene oxide)‐Block‐Polyphosphester‐Based Paclitaxel Conjugates as a Platform for Ultra‐High Paclitaxel‐Loaded Multifunctional Nanoparticles,” Chemical Science 4 (2013): 2122–2126, 10.1039/c3sc50252j. 25152808 PMC4138828

[17] C. Giacomelli , V. Schmidt , and R. Borsali , “Nanocontainers Formed by Self‐Assembly of Poly(Ethylene Oxide)‐b‐Poly(Glycerol Monomethacrylate)−Drug Conjugates,” Macromolecules 40 (2007): 2148–2157, 10.1021/ma062562u.

[18] Y. Yu , C.‐K. Chen , W.‐C. Law , et al., “Polylactide‐Graft‐doxorubicin Nanoparticles With Precisely Controlled Drug Loading for pH‐Triggered Drug Delivery,” Biomacromolecules 15 (2014): 524–532, 10.1021/bm401471p. 24446700

[19] J. G. H. Hermens , T. Freese , K. J. van den Berg , R. van Gemert , and B. L. Feringa , “A Coating From Nature,” Science Advances 6 (2020): eabe0026, 10.1126/sciadv.abe0026. 33328241 PMC7744085

[20] B. L. Feringa , J. G. H. Hermens , K. J. van den Berg , and R. van Gemert , WO2021/084066, (2021).

[21] B. L. Feringa , J. G. H. Hermens , K. J. van den Berg , and R. van Gemert , WO2021/259819, (2021).

[22] A. Jaswal , P. P. Singh , and T. Mondal , “Furfural—a Versatile, Biomass‐Derived Platform Chemical for the Production of Renewable Chemicals,” Green Chemistry 24 (2022): 510–551, 10.1039/D1GC03278J.

[23] A. P. Dunlop , US2536732A, (1951).

[24] K. J. Zeitsch , US6743928B1, (2004).

[25] L. Liu , H. M. Chang , H. Jameel , and S. Park , “Furfural Production From Biomass Pretreatment Hydrolysate Using Vapor‐Releasing Reactor System,” Bioresource Technology 252 (2018): 165–171, 10.1016/j.biortech.2018.01.006. 29324276

[26] G. O. Schenck , “Photochemische Reaktionen II. Über die Unsensibilisierte und Photosensibilisierte Autoxydation Von Furanen,” Justus Liebigs Annalen Der Chemie 584 (1953): 156–176, 10.1002/jlac.19535840111.

[27] B. L. Feringa , “Photo‐Oxidation of furans,” Recueil Des Travaux Chimiques Des Pays‐Bas 106 (1987): 469–488, 10.1002/recl.19871060902.

[28] J. G. H. Hermens , M. L. Lepage , A. Kloekhorst , et al., “Development of a Modular Photoreactor for the Upscaling of Continuous Flow Photochemistry,” Reaction Chemistry & Engineering 7 (2022): 2280–2284, 10.1039/D2RE00310D. 36352841 PMC9594834

[29] M. D. Edwards , M. T. Pratley , C. M. Gordon , et al., “Process Intensification of the Continuous Synthesis of Bio‐Derived Monomers for Sustainable Coatings Using a Taylor Vortex Flow Reactor,” Organic Process Research & Development 28 (2024): 1917–1928, 10.1021/acs.oprd.3c00462. 38783853 PMC11110062

[30] M. L. Lepage , G. Alachouzos , J. G. H. Hermens , N. Elders , K. J. van den Berg , and B. L. Feringa , “Electron‐Poor Butenolides: The Missing Link Between Acrylates and Maleic Anhydride in Radical Polymerization,” Journal of the American Chemical Society 145 (2023): 17211–17219, 10.1021/jacs.3c04314. 37498188 PMC10416300

[31] M. L. Lepage , B. L. Feringa , J. G. H. Hermens , K. J. van den Berg , and N. Elders , WO2024126828A1, (2024).

[32] M. L. Lepage , B. L. Feringa , J. G. H. Hermens , K. J. van den Berg , and N. Elders , WO2024126829A1, (2024).

[33] M. L. Lepage , B. L. Feringa , J. G. H. Hermens , K. J. van Berg , and N. Elders , WO2024126832A1, (2024).

[34] J. G. H. Hermens , T. Freese , G. Alachouzos , et al., “A Sustainable Polymer and Coating System Based on Renewable Raw Materials,” Green Chemistry 24 (2022): 9772–9780, 10.1039/D2GC03657F.

[35] A. Jensma , N. Elders , K. J. Van den Berg , and B. L. Feringa , “Waterborne Polymers and Coatings From Bio‐Based Butenolides,” Green Chemistry 26 (2024): 9676–9681, 10.1039/D4GC03466J. 39175958 PMC11333933

[36] T. Freese , J. P. Kaniraj , B. L. Feringa , A. Jensma , N. Elders , and K. J. van den Berg , P139663NL00, (2025).

[37] C. Bax , S. Sironi , and L. Capelli , “How Can Odors Be Measured? An Overview Of Methods and Their Applications,” Atmosphere 11 (2020): 92, 10.3390/atmos11010092.

[38] C. A. De March , S. Ryu , G. Sicard , C. Moon , and J. Golebiowski , “Structure–Odour Relationships Reviewed in the Postgenomic Era,” Flavour and Fragrance Journal 30, no. 5 (2015): 342–361, 10.1002/ffj.3249.

[39] J. E. Cometto‐Muñiz and M. H. Abraham , “Human Olfactory Detection of Homologues n‐Alcohols Measured Via Concentration‐Response Functions,” Pharmacology 89 (2008): 279–291, 10.1016/j.pbb.2007.12.023. PMC2323909 18258288

[40] M. Zarzo , “Effect of Functional Group and Carbon Chain Length on the Odor Detection Threshold of Aliphatic Compounds,” Sensors 12 (2012): 4105–4112, 10.3390/s120404105. 22666021 PMC3355402

[41] G. Leonardos , D. Kendall , and N. Barnard , “Odor Threshold Determinations of 53 Odorant Chemicals,” Journal of the Air Pollution Control Association 19, no. 2 (1969): 91–95, 10.1080/00022470.1969.10466465.

[42] M. Lapuerta , J. P. Hernández , and J. Agudelo , “An Equation for the Estimation of Alcohol‐Air Diffusion Coefficients for Modelling Evaporation Losses in Fuel Systems,” Applied Thermal Engineering 73, no. 1 (2014): 539–548, 10.1016/j.applthermaleng.2014.08.009.

