# Supplementary material for: Slow Release of Bioactive Alcohols From Butenolide Polymers and Coatings
Source: Chemistry. 2026 Mar 28;32(22):e70951. doi: 10.1002/chem.70951 (PMC13250347; doi:10.1002/chem.70951)
Supplement: Supplementary file 1 — Supporting File: The file contains experimental details, materials, and methods; 1H, 13C, COSY, and HSQC NMR spectra for all novel compounds; copolymerization data; differential scanning calorimetry (DSC) and gel permeation chromatography (GPC) analysis of polymers; and headspace GC‐MS data of alcohol release. Additional references cited within the Supporting Information [37, 38, 39, 40, 41, 42]. [file CHEM-32-e70951-s001.pdf]

Supporting Information

Slow Release of Bioactive Alcohols from Butenolide Polymers and Coatings

A. Jensma,[a] J.T. Jaeger,[a] J.G.H. Hermens,[b] T. Freese,[a][c] J.T. Meijer,[a] R. Tarozo,[a] J.L. Sneep,[a] N. Elders,[b] K.J. Van den Berg,[b] and B.L. Feringa[a],*

[a] Stratingh Institute for Chemistry, Advanced Research Center Chemical Building Blocks Consortium (ARC CBBC), University of Groningen, Nijenborgh 4, 9747 AG Groningen, The Netherlands. E-mail: b.l.feringa@rug.nl

[b] Department Resin Technology, AkzoNobel Car Refinishes BV, Rijksstraatweg 31, 2171 AJ Sassenheim, The Netherlands

[c] R&D Department Circolide, Circolide Technologies B.V., Nijenborgh 7, 9747AG Groningen, The Netherlands

Contents

General....4
Commercial reagents and solvents....4
Reagents and solvents....4
General considerations....4
Equipment....4
Synthesis....6
Ethoxy butenolide....6
Hexyloxy butenolide....10
Dodecyloxy butenolide....14
Citronelloxy butenolide....18
Dihydrocitronelloxy butenolide....22
Phenylethoxy butenolide....26
Menthyloxy butenolide....30
Methods....34
Co-polymerizations for conversion and kinetic rate measurement....34
Reaction kinetics....34
Co-polymerization for molecular weight distribution and glass transition temperature....35
General procedure for UV-curing of coatings....35
Extended Discussion on release and the analysis of released alcohols....36
Quantification of release from polymers and coatings....36
General procedure for release of alcohols from monomers....39
General procedure for release of alcohols from polymers/coatings....39
Co-polymerization kinetics data....39
Poly(ethoxy butenolide-co-dodecyl vinyl ether) (1-DVE)....40
Poly(ethoxy butenolide-co-ethylene glycol vinyl ether) (1-EGVE)....42
Poly(hexyloxy butenolide-co-dodecyl vinyl ether) (2-DVE)....44
Poly(hexyloxy butenolide-co-ethylene glycol vinyl ether) (2-EGVE)....46
Poly(dodecyloxy butenolide-co-dodecyl vinyl ether) (3-DVE)....48
Poly(dodecyloxy butenolide-co-ethylene glycol vinyl ether) (3-EGVE)....50
Poly(citronelloxy butenolide-co-dodecyl vinyl ether) (4-DVE)....52
Poly(citronelloxy butenolide-co-ethylene glycol vinyl ether) (4-EGVE)....54
Poly(dihydrocitronelloxy butenolide-co-dodecyl vinyl ether) (5-DVE)....56
Poly(dihydrocitronelloxy butenolide-co-ethylene glycol vinyl ether) (5-EGVE)....58
Poly(phenylethoxy butenolide-co-dodecyl vinyl ether) (6-DVE)....60

Poly(phenylethoxy butenolide-co-ethylene glycol vinyl ether) (6-EGVE) 62
Poly(menthyloxy butenolide-co-dodecyl vinyl ether) (7-DVE) 64
Poly(menthyloxy butenolide-co-ethylene glycol vinyl ether) (7-EGVE) 66
Gel permeation chromatography (GPC) 68
Differential Scanning calorimetry (DSC) 75
Release of alcohols from butenolide monomers 82
Ethoxy butenolide (pH 1 / 10 mol% H^+) 83
Ethoxy butenolide (pH 2 / 1 mol% H^+) 84
Ethoxy butenolide (pH 6 / 0.0001 mol% H^+) 85
Hexyloxy butenolide (pH 1 / 10 mol% H^+) 86
Hexyloxy butenolide (pH 2 / 1 mol% H^+) 87
Hexyloxy butenolide (pH 6 / 0.0001 mol% H^+) 88
Release of alcohols from butenolide copolymers and coatings 89
Summary of alcohol release from co-polymers and coatings 89
Odor Detection Threshold and Odor Perception Evaluation 91
Ethanol release from co-polymers 1-DVE and 1-EGVE 94
Hexanol release from co-polymers 2-DVE and 2-EGVE 96
Dihydrocitronellol release from co-polymer 5-EGVE 98
Phenylethanol release from co-polymer 6-EGVE 99
Menthol release from co-polymer 7-EGVE 100
DSC of polymers before and after release 101

General

Commercial reagents and solvents

Unless stated otherwise, all commercially available reagents and solvents were purchased from the following commercial sources: Sigma-Aldrich, Acros, Macron, AkzoNobel Coatings BV and were used as received. Anhydrous solvents were purified by passage through solvent purification columns (MBraun SPS-800). For aqueous solutions, demineralized water was used.

Reagents and solvents

Furfural (99%), methylene blue, hexanol anhydrous (≥99%), (-)-menthol (99%), 1,3,5-trimethoxybenzene (≥99%), phenylbis(2,4,6-trimethylbenzoyl)phosphine oxide (Omnirad 819, 97%), dodecyl vinyl ether (98%), diethylene glycol divinyl ether (99%), ethylene glycol vinyl ether (97%), 2-phenylethanol (>99%), 1-dodecanol (98%), citronellol (95%), dihydrocitronellol (95%), potassium permanganate (>99%), sodium sulphate (>99%), magnesium sulphate (>99%), Amberlyst®15 (hydrogen form, >50 mesh, strongly acidic), molecular sieves (3 Å, 4-8 mesh), aluminium oxide (activated, basic) and butyl acetate (anhydrous, ≥99%) were purchased from *Sigma-Aldrich* (Zwijndrecht, The Netherlands).

tert-Butyl peroxy-3,5,5-trimethylhexanoate (Trigonox 42S) was obtained from AkzoNobel Car Refinishes BV (Sassenheim, The Netherlands).

Tetrahydrofuran (THF, Ar grade) and *N*,*N*-dimethylformamide (DMF, AR grade) were purchased from *Biosolve BV* (Valkenswaard, The Netherlands)

Methanol anhydrous (MeOH, 99%, AR grade), ethanol anhydrous (EtOH, 99%, AR grade), ethyl acetate (EtOAc, HPLC grade), n- pentane (99%), n-heptane (HPLC grade), tetrahydrofuran (THF, HPLC grade), dimethylformide and diethyl ether (Et2O, stabilized with BHT, AR grade) were purchased from Macron.

General considerations

Thin Layer Chromatography (TLC) analyses were performed on commercial Kieselgel 60, F254 silica gel plates with fluorescence-indicator UV_{254} (Merck, TLC silica gel 60 F_{254}). For detection of components, UV light at 254 nm was used. Alternatively, oxidative staining using aqueous basic potassium permanganate solution ($KMnO_4$) was used. Drying of solutions was performed with Na_2SO_4 or $MgSO_4$ and volatiles were removed with a rotary evaporator. Flash column chromatography was conducted with a Büchi Pure Chromatography System at room temperature with pre-packed silica cartridges purchased from Büchi.

Equipment

UV Flood 36 (12 x 3 W, λ = 395 nm, 21 mW/cm^2) as light source for UV-curing of coatings.
Byk applicator (50-200 µm, 10 cm) to apply coatings.

General Analytical information

Nuclear Magnetic Resonance spectra were measured with an Agilent Technologies 400-MR (400/54 Premium Shielded) spectrometer (400 MHz). All spectra were measured at room temperature (22–24°C). Chemical shifts for the specific NMR spectra were reported relative to the residual solvent peak [in ppm; CDCl3:δ_H = 7.26; CDCl3: δ_C = 77.16; DMSO-d_6: δ_H = 2.50; DMSO-d_6: δ_C = 39.52]. The multiplicities of the signals are denoted by s (singlet), d (doublet),

t (triplet), q (quartet), m (multiplet), br s (broad signal), app (apparent). All ^{13}C-NMR spectra are ^{1}H-broadband decoupled.

High-resolution mass spectrometric measurements were performed using a Thermo scientific LTQ OrbitrapXL spectrometer with ESI or APPCI ionization. The results are given in *m/z*-units.

Molecular weight distribution was measured by GPC with a Hewlett Packard 1100 Series equipped with a GBC 1240 RID for detection. The flow rate of the mobile phase (THF or DMF) was set at 1 mL/min. 10 mM LiBr was added to the DMF mobile phase. The stationary phase was a set of 3 columns Agilent Technologies PLgel Mixed E 300 × 7.5 mm 3 µm, kept at 40°C. Injection volume was 20 µL for THF and 45 µL for DMF. The GPC was calibrated using narrow band polystyrene standards. Toluene was added to samples as an internal standard for correction of retention time.

Glass temperatures (T_g) were measured with a Differential Scanning Calorimeter (DSC) Q1000 (TA Instruments) in aluminum pans covered with lids, both prepared from 0.1 mm aluminum sheets.

(Headspace) Gas chromatography - mass spectrometry (GC-MS) was measured on a Shimadzu QP2010 with HP5 column (30 m x 0.25 mm x 0.25 µm) using Helium as carrier gas (flow 1 mL/min). The inlet was kept at 250 °C and the sample was injected in splitless mode for 1 min. The oven was kept at 40 °C (for 1 min) and then ramped up to 250 °C at 10 °C /min. Data was acquired in full scan mode (40-500 m/z) and processed using GCMS LabSolutions (GCMSsolution 4.52 software). Autosampler Shimadzu AOC-5000 plus was used to perform the headspace extraction. The incubation oven was at 30 °C (for 5 min) and with an agitation speed of 250 rpm. The syringe (2,5 mL) was also kept at 30 °C.

Synthesis

Hydroxy butenolide was synthesized at 100 g-scale by continuous flow photooxygenation using a modular photoreactor (Hermens *et al.*)[28]

Alkoxy butenolides (**1-7**) were synthesized as described by T. Freese and co-workers.[36]

Ethoxy butenolide

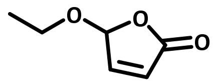

5-ethoxyfuran-2(5*H*)-one
Chemical Formula: $C_6H_8O_3$
Molecular Weight: 128.13

Hydroxy butenolide (10.00 g, 0.1 mol, 1 eq.) and Amberlyst®15 (1.0 g, 10wt%) in 250 mL ethanol (43 eq.) were added to a 1000 mL round-bottom flask. The reaction mixture was rotated in the flask at 20 °C overnight using a rotatory evaporator. The product mixture was filtered to remove the solid catalyst and the solution was concentrated *in vacuo*, yielding a colorless oil. The crude product was purified by column chromatography (silica gel, *n*-pentane:ethyl acetate/ 90:10), yielding 5-ethoxyfuran-2(5H)-one **2** (9.34 g, 73 mmol, 72.9%) as a colorless oil.

^{1}H-NMR (400 MHz, $CDCl_3$) δ 7.20 (dd, J = 5.7, 1.0 Hz, 1H), 6.21 (d, J = 1.0 Hz, 1H), 5.92 (d, J = 1.1 Hz, 1H), 3.92 (dq, J = 9.3, 7.1, 0.8 Hz, 1H), 3.74 (dq, J = 9.4, 7.1, 0.8 Hz, 1H), 1.27 (t, J = 0.8 Hz, 3H).

^{13}C-NMR (101 MHz, $CDCl_3$) δ 170.65, 150.54, 124.96, 103.28, 66.21, 15.05.

HRMS ESI-pos $[M+H]^+$ $C_6H_9O_3$ calc. 129.0546, found 129.0544.

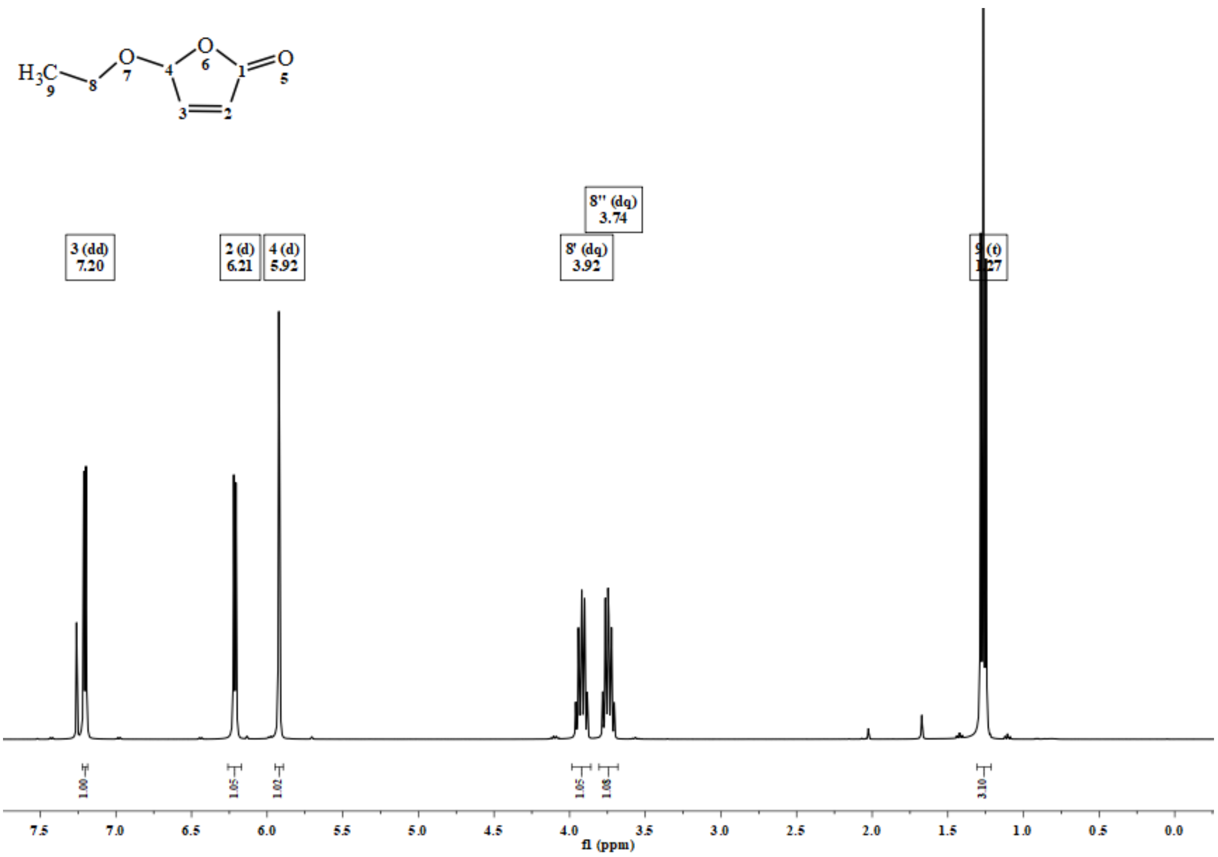

Figure S1. ^{1}H-NMR spectrum (400 MHz) of ethoxy butenolide measured at 298 K in $CDCl_3$.

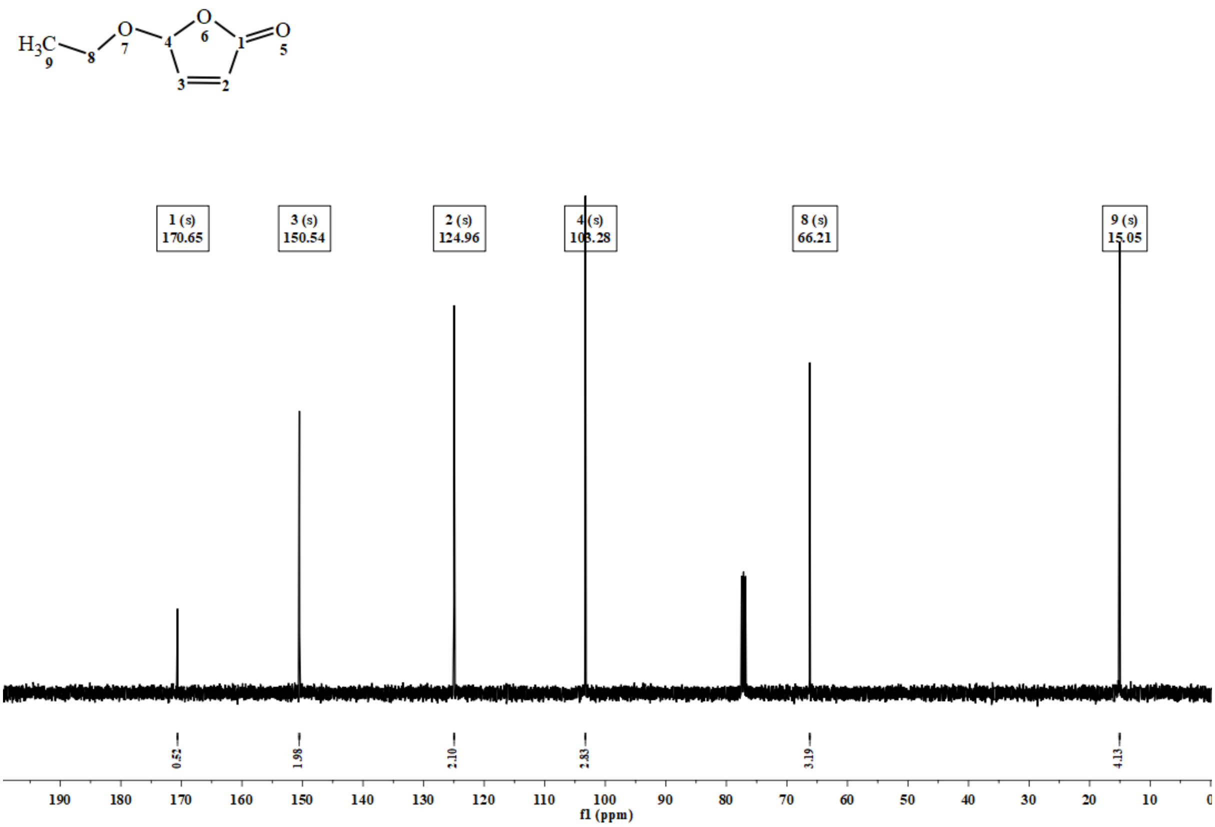

Figure S2. ^{13}C-NMR spectrum (101 MHz) of ethoxy butenolide measured at 298 K in $CDCl_3$.

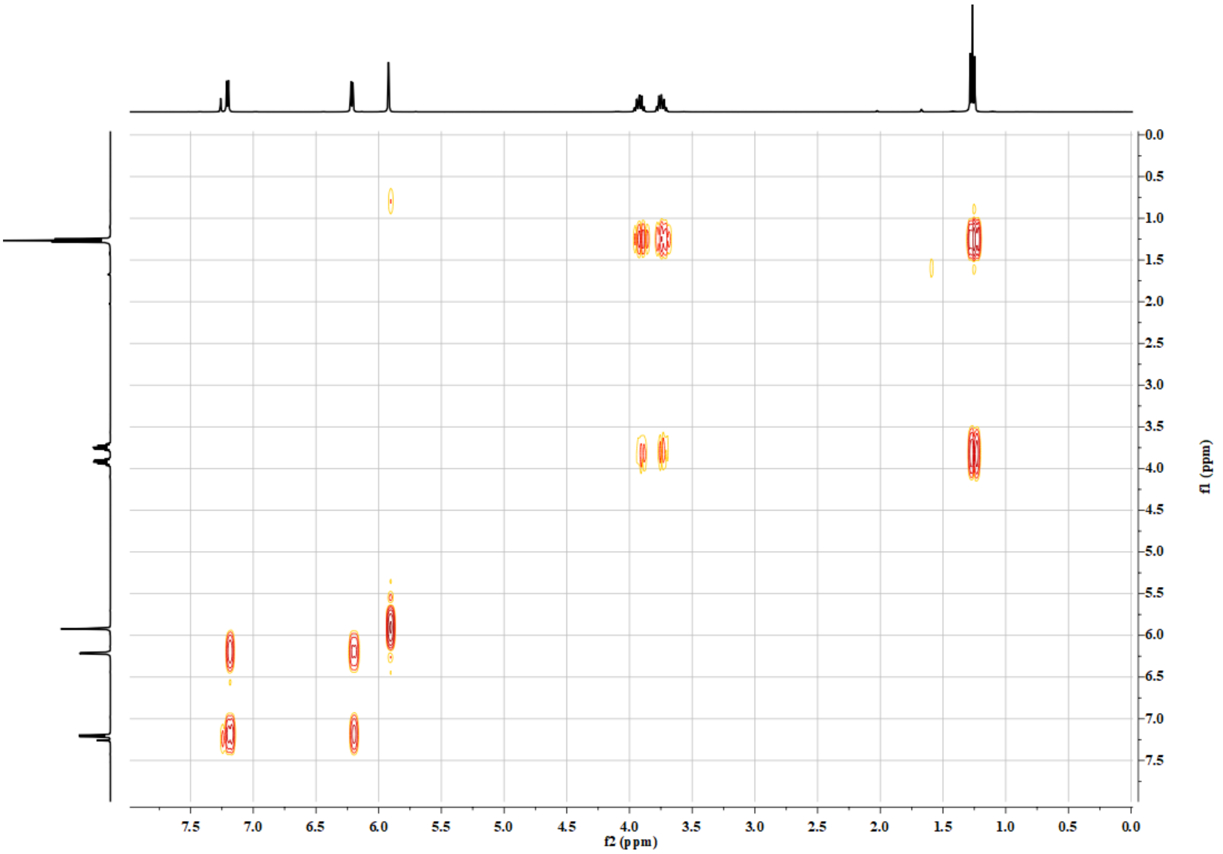

Figure S3. COSY NMR spectrum of ethoxy butenolide measured at 298 K in $CDCl_3$.

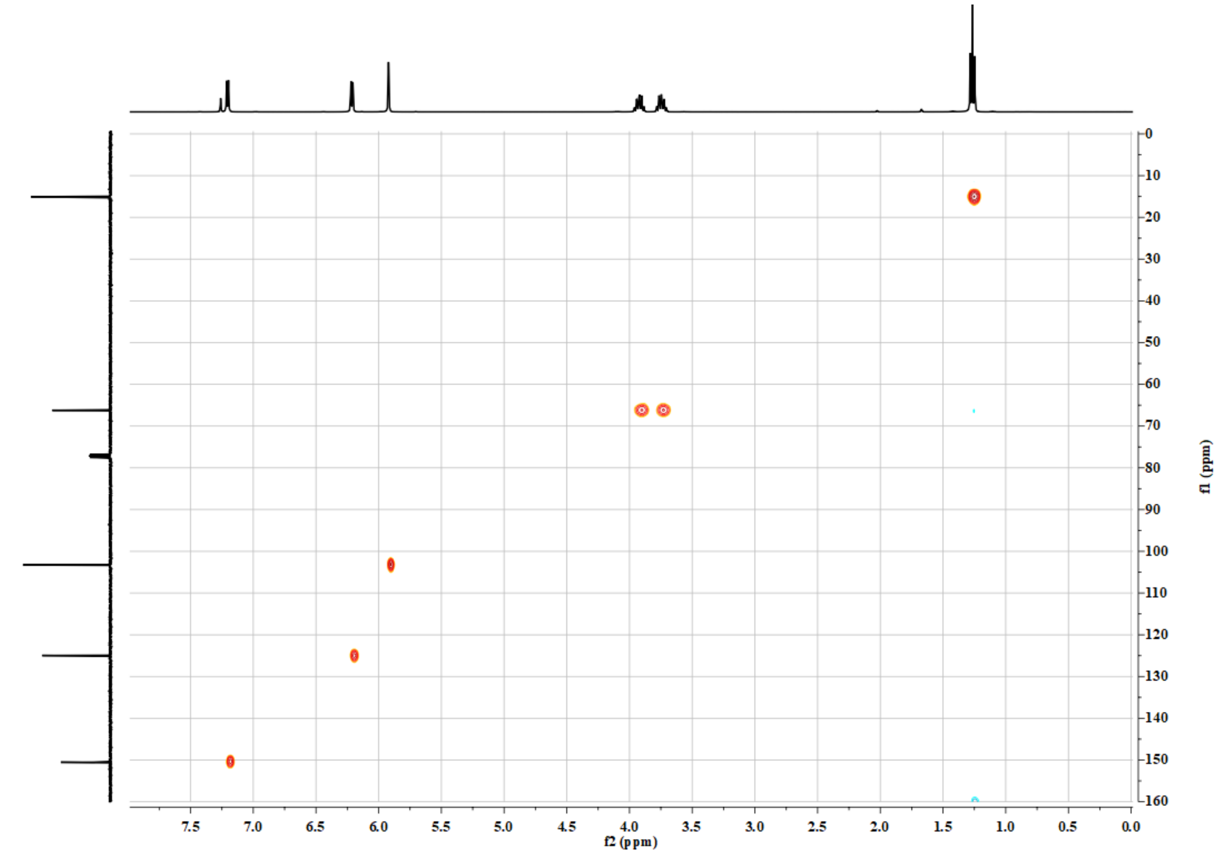

Figure S4. HSQC NMR spectrum of ethoxy butenolide measured at 298 K in $CDCl_3$.

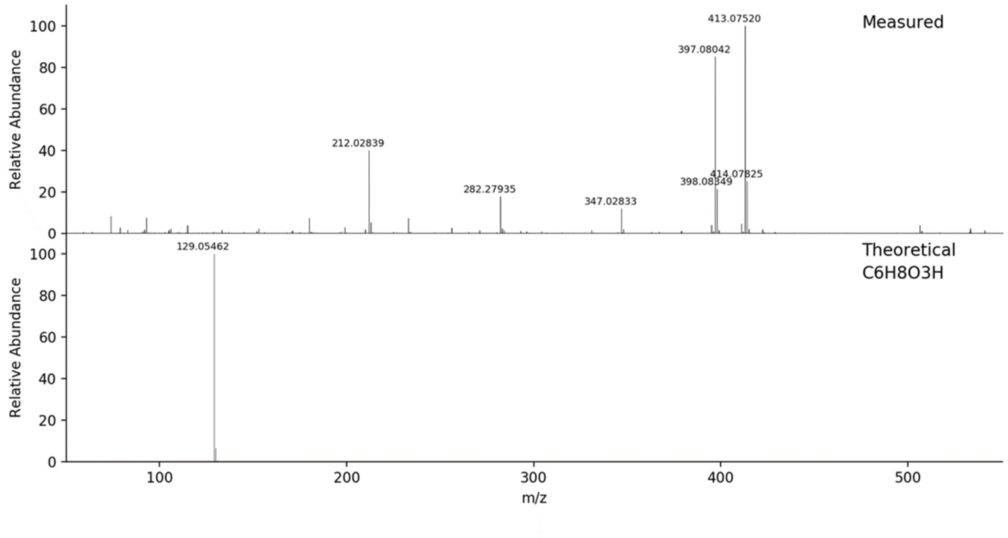

Figure S5. HRMS of ethoxy butenolide (top: measured, bottom: calculated), LTQ Orbitrap XL (ESI+).

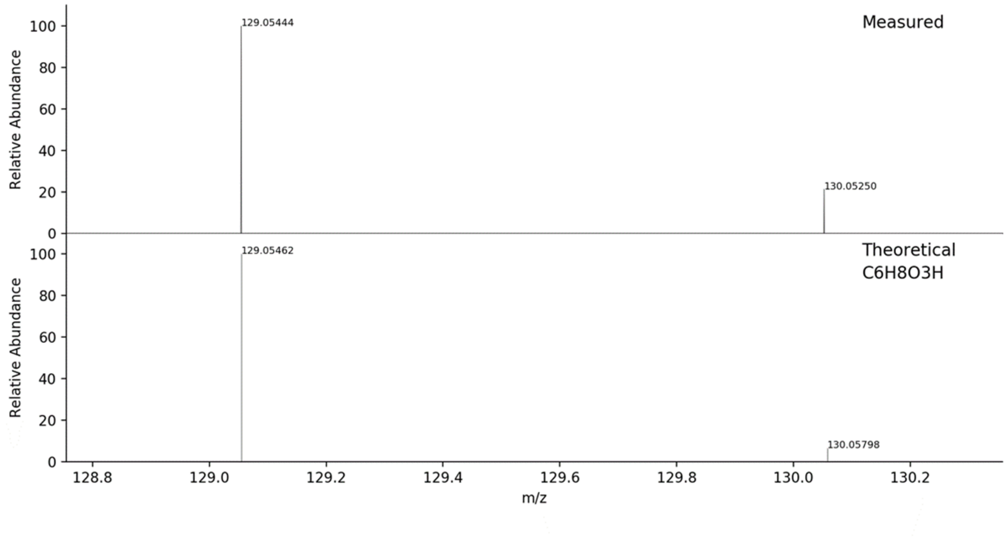

Figure S6. HRMS (zoomed in) of ethoxy butenolide (top: measured, bottom: calculated), LTQ Orbitrap XL (ESI+).

Hexyloxy butenolide

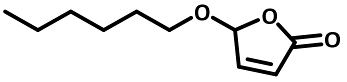

5-(hexyloxy)furan-2(5*H*)-one
Chemical Formula: $C_{10}H_{16}O_3$
Molecular Weight: 184.24

A mixture of hydroxy butenolide (13.21 g, 132 mmol, 1.1 eq.), hexanol (12.26 g, 120 mmol, 1 eq.), Amberlyst®15 (3.7 g, 30 wt%), and activated molecular sieves (3.7 g, 30 wt%) in 110 mL dry toluene was prepared in a 500 mL three-necked flask under nitrogen. The flask was stirred at max. 200 rpm using a magnetic stirring egg (in order to prevent damaging the solid acid catalyst) and kept at 40 °C overnight. The product mixture was filtered to remove the solid catalyst and the solution was concentrated *in vacuo*.

The crude product was dissolved in ethyl acetate and washed with $NaHCO_3$ solution (3 x 50 mL, 1M). Subsequently, it was washed with water (2 x 50 mL) and brine (1 x 50 mL) before drying over $MgSO_4$. The solution was concentrated *in vacuo* and 5-hexyloxyfuran-2(5H)-one 3 (19.24 g, 104 mmol, 87.0%) was obtained as a colorless oil.

^{1}H-NMR (400 MHz, $CDCl_3$) δ 7.21 (dd, J = 5.7, 0.9 Hz, 1H), 6.22 (d, J = 1.0 Hz, 1H), 5.92 (d, J = 1.2 Hz, 1H), 3.86 (dt, J = 9.2, 6.6 Hz, 1H), 3.66 (dt, J = 9.4, 6.7 Hz, 1H), 1.67-1.58 (m, 2H), 1.39-1.22 (m, 7H), 0.88 (t, J = 6.7 Hz, 3H).

^{13}C-NMR (101 MHz, $CDCl_3$) δ 170.72, 150.49, 125.13, 103.53, 70.82, 31.61, 29.53, 25.66, 22.66, 14.13.

HRMS ESI-pos $[M+H]^+$ $C_{10}H_{17}O_3$ calc. 185.1172, found 185.1172

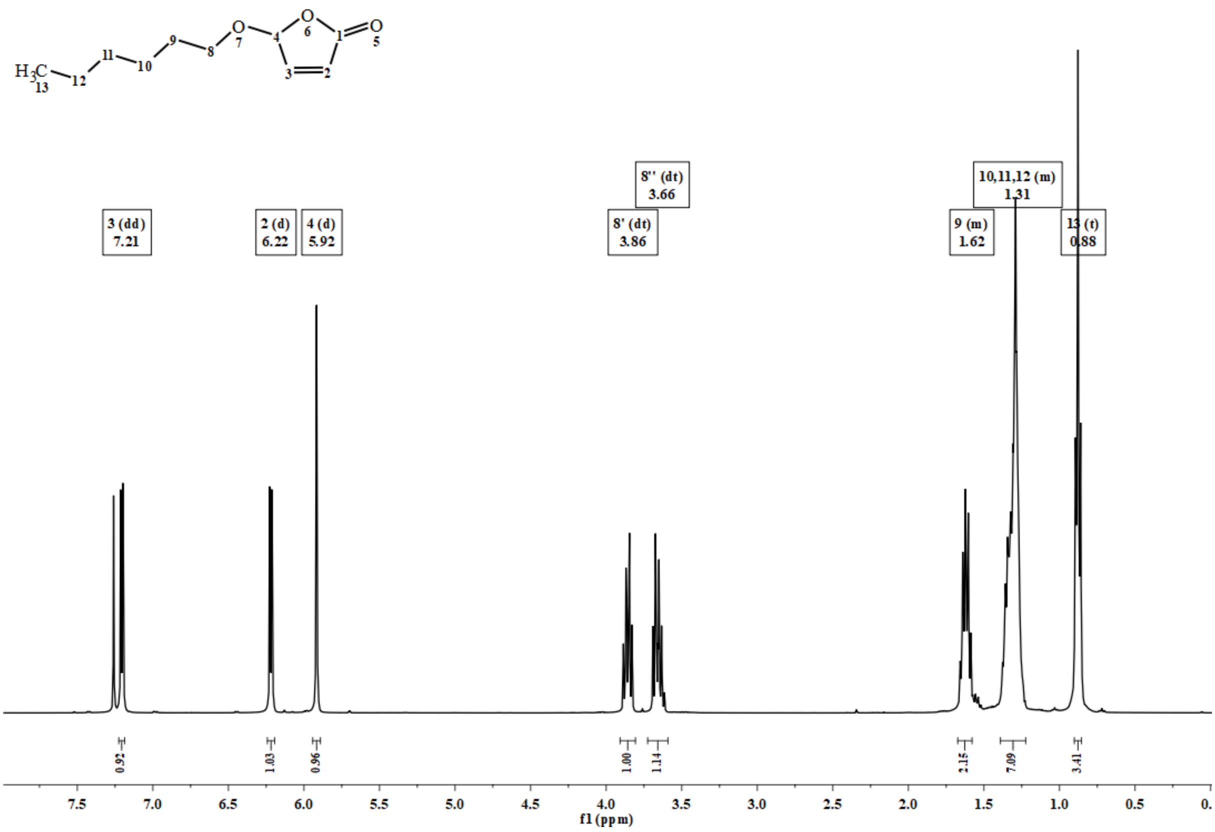

Figure S7. ^{1}H-NMR spectrum (400 MHz) of hexyloxy butenolide measured at 298 K in $CDCl_3$.

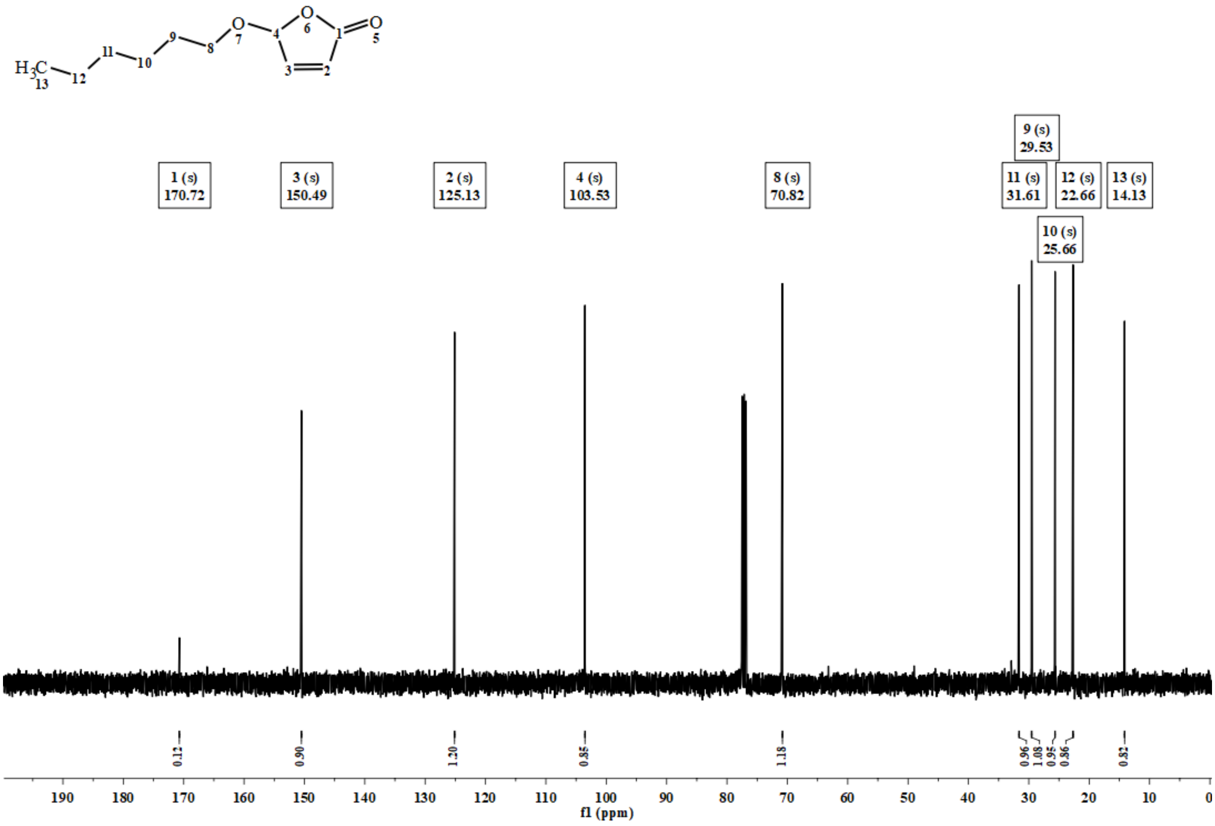

Figure S8. ^{13}C-NMR spectrum (101 MHz) of hexyloxy butenolide measured at 298 K in $CDCl_3$.

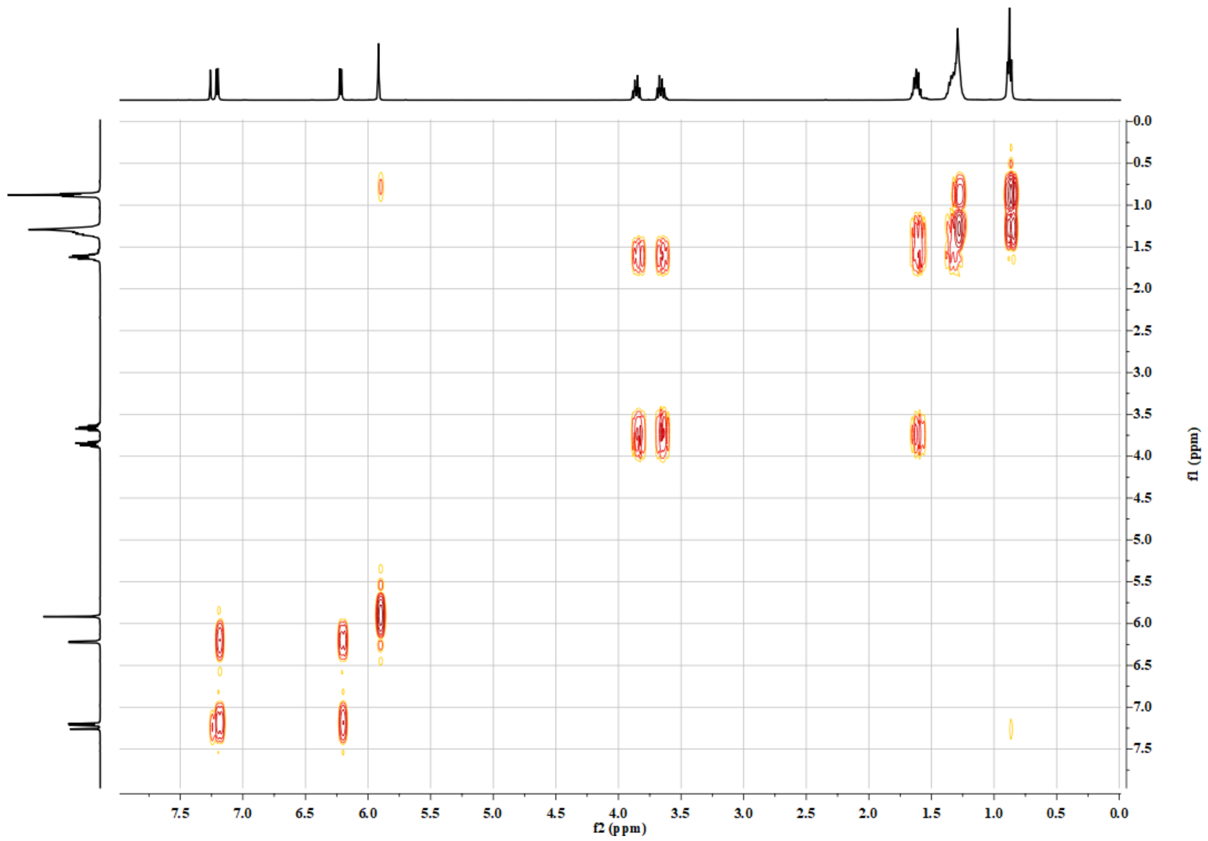

Figure S9. COSY NMR spectrum of hexyloxy butenolide measured at 298 K in $CDCl_3$.

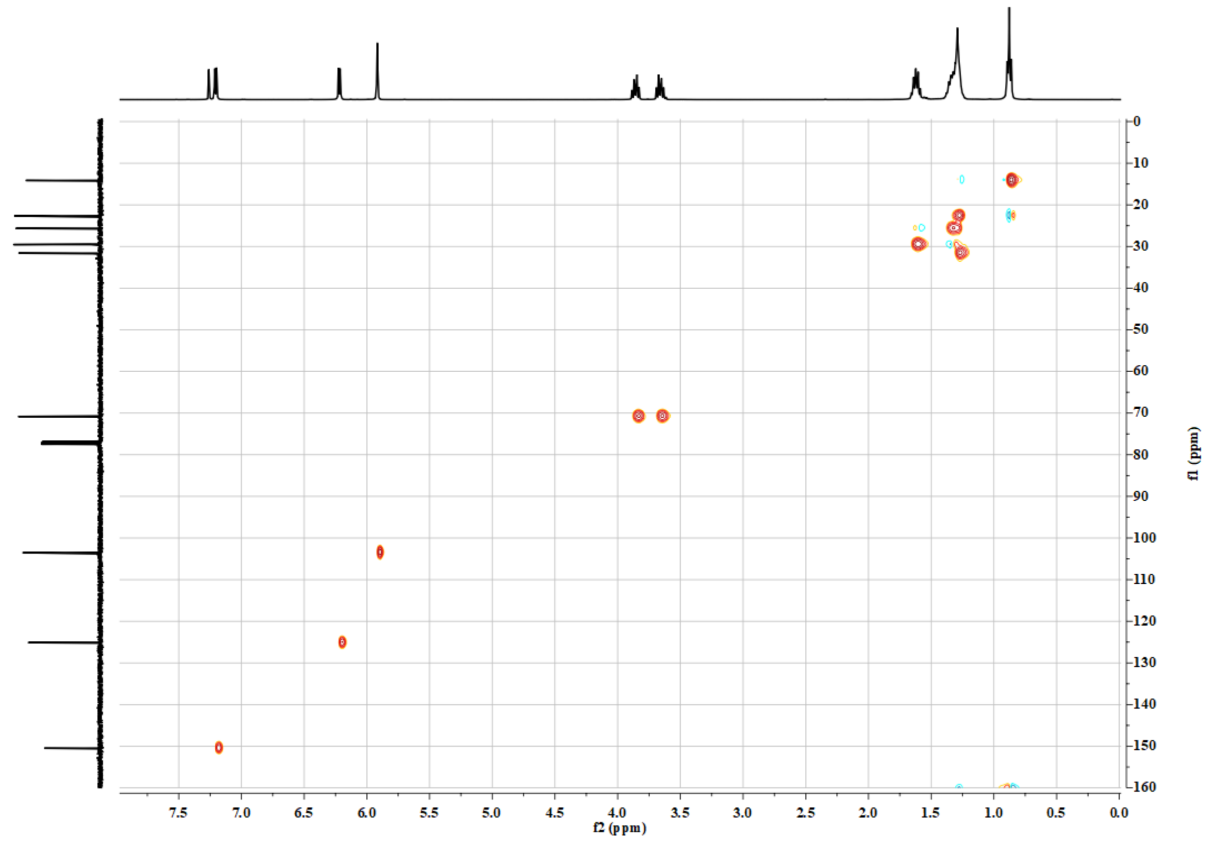

Figure S10. HSQC NMR spectrum of hexyloxy butenolide measured at 298 K in $CDCl_3$.

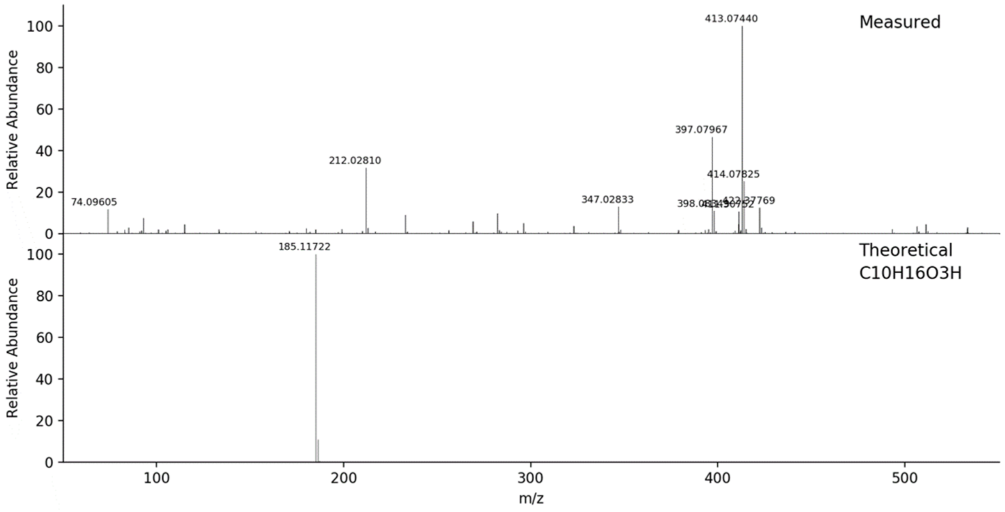

Figure S11. HRMS of hexyloxy butenolide (top: measured, bottom: calculated), LTQ Orbitrap XL (ESI+).

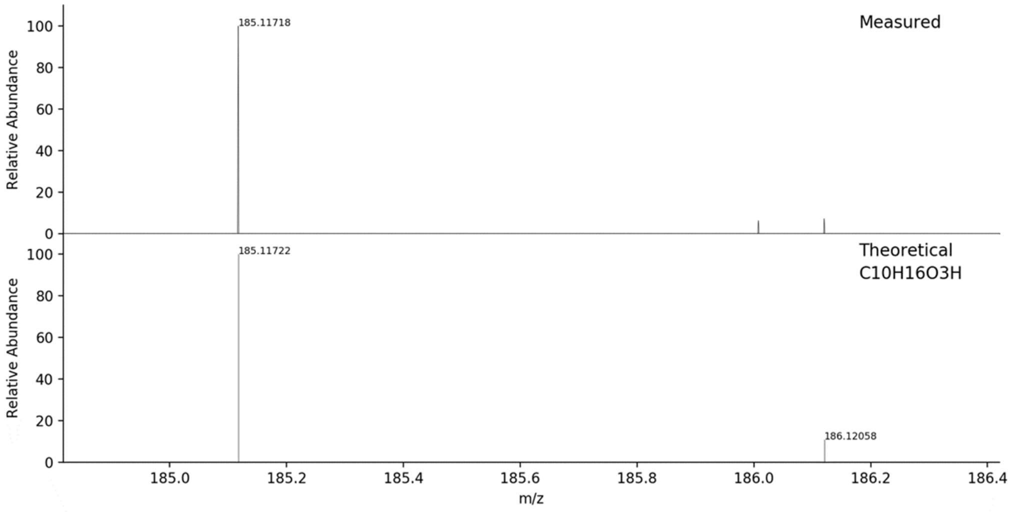

Figure S12. HRMS (zoomed in) of hexyloxy butenolide (top: measured, bottom: calculated), LTQ Orbitrap XL (ESI+).

Dodecyloxy butenolide

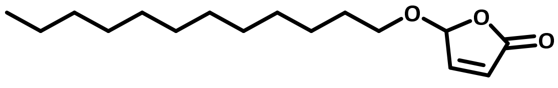

5-(dodecyloxy)furan-2(5*H*)-one
Chemical Formula: $C_{16}H_{28}O_3$
Molecular Weight: 268.40

A mixture of hydroxy butenolide (2.16 g, 22 mmol, 1.1 eq.), dodecanol (3.66 g, 20 mmol, 1 eq.), Amberlyst®15 (1.0 g, 30 wt%), and activated molecular sieves (1.0 g, 30 wt%) in 20 mL dry toluene was prepared in a 100 mL three-necked flask under nitrogen. The flask was stirred at max. 200 rpm using a magnetic stirring egg (in order to prevent damaging the solid acid catalyst) and kept at 40 °C overnight. The product mixture was filtered to remove the solid catalyst and the solution was concentrated *in vacuo*.

The crude product was dissolved in ethyl acetate (40 mL) and washed with $NaHCO_3$ solution (3 x 20 mL, 1M). Subsequently, it was washed with water (2 x 20 mL) and brine (1 x 20 mL) before drying over $MgSO_4$. The solution was concentrated *in vacuo* and 5-dodecyloxyfuran-2(5H)-one 3 (4.09 g, 15 mmol, 70.6%) was obtained as a viscous colorless oil that solidified upon standing.

^{1}H-NMR (400 MHz, $CDCl_3$) δ 7.19 (dd, 1H), 6.21 (d, 1H), 5.91 (d, J = 1.3 Hz, 1H), 3.84 (td, J = 8.1, 5.9 Hz, 1H), 3.65 (dt, J = 10.0, 6.6 Hz, 1H), 1.66-1.58 (m, 2H), 1.24 (s, 18H), 0.87 (t, 3H).

^{13}C-NMR (101 MHz, $CDCl_3$) δ 150.47, 125.18, 103.53, 70.85, 32.06, 29.79, 29.77, 29.72, 29.67, 29.61, 29.49, 29.46, 26.02, 22.84, 14.27.

HRMS ESI-pos $[M+H]^+$ $C_{16}H_{29}O_3$ calc. 269.2111, found 269.2113.

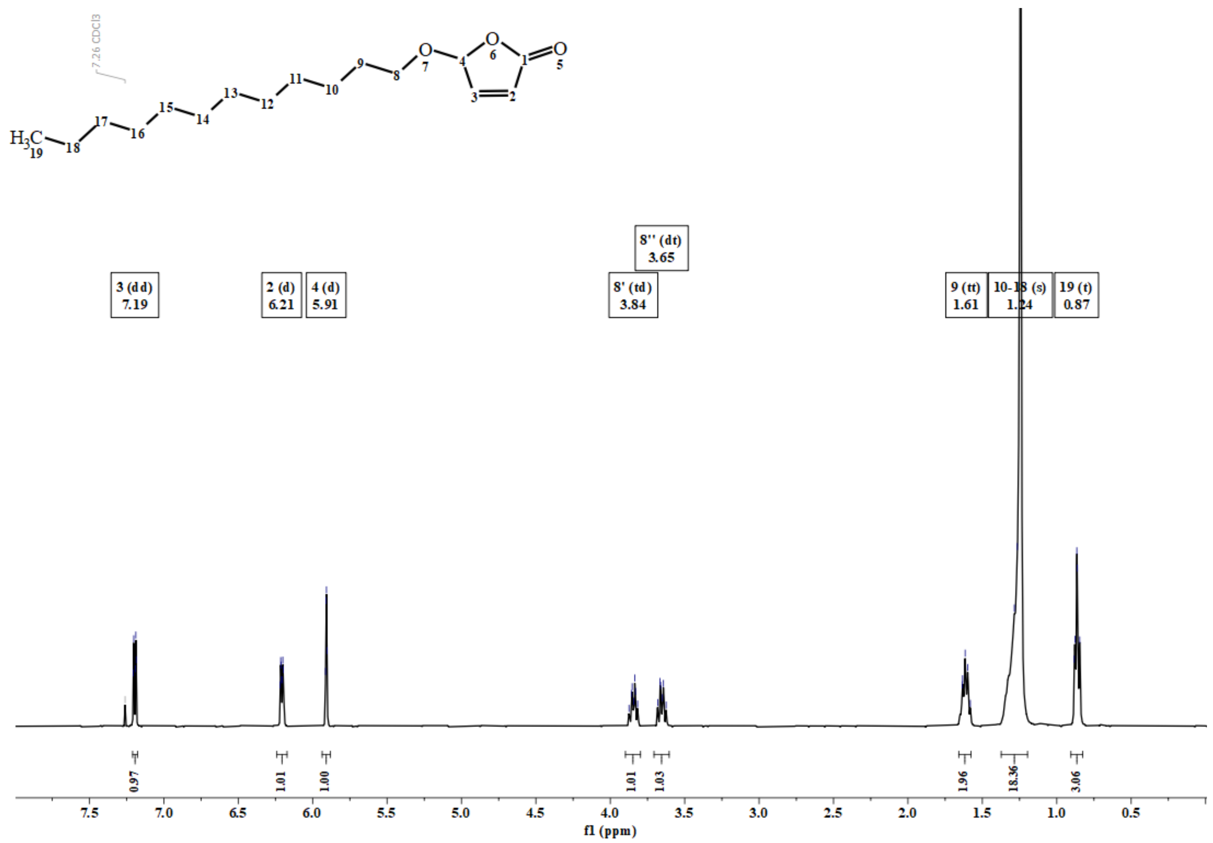

Figure S13. ^{1}H-NMR spectrum (400 MHz) of dodecyloxy butenolide measured at 298 K in $CDCl_3$.

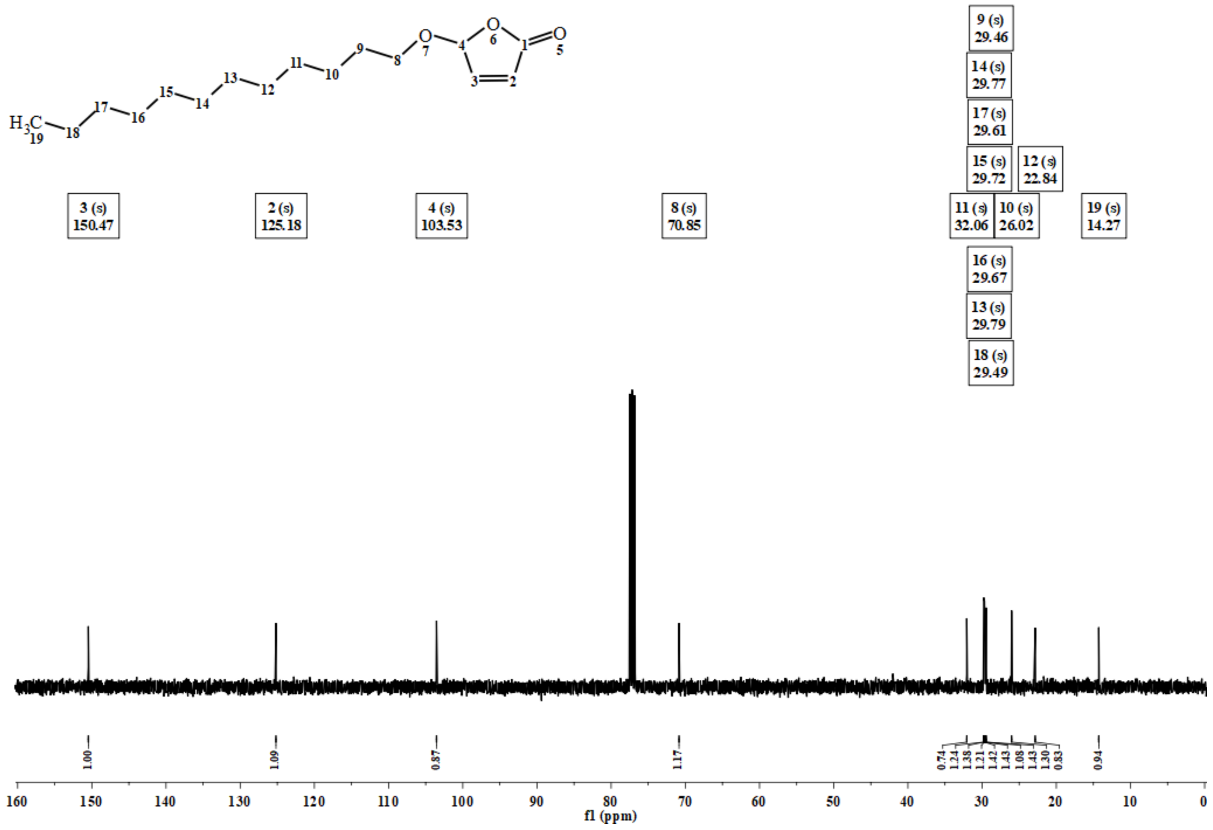

Figure S14. ^{13}C-NMR spectrum (101 MHz) of dodecyloxy butenolide measured at 298 K in $CDCl_3$.

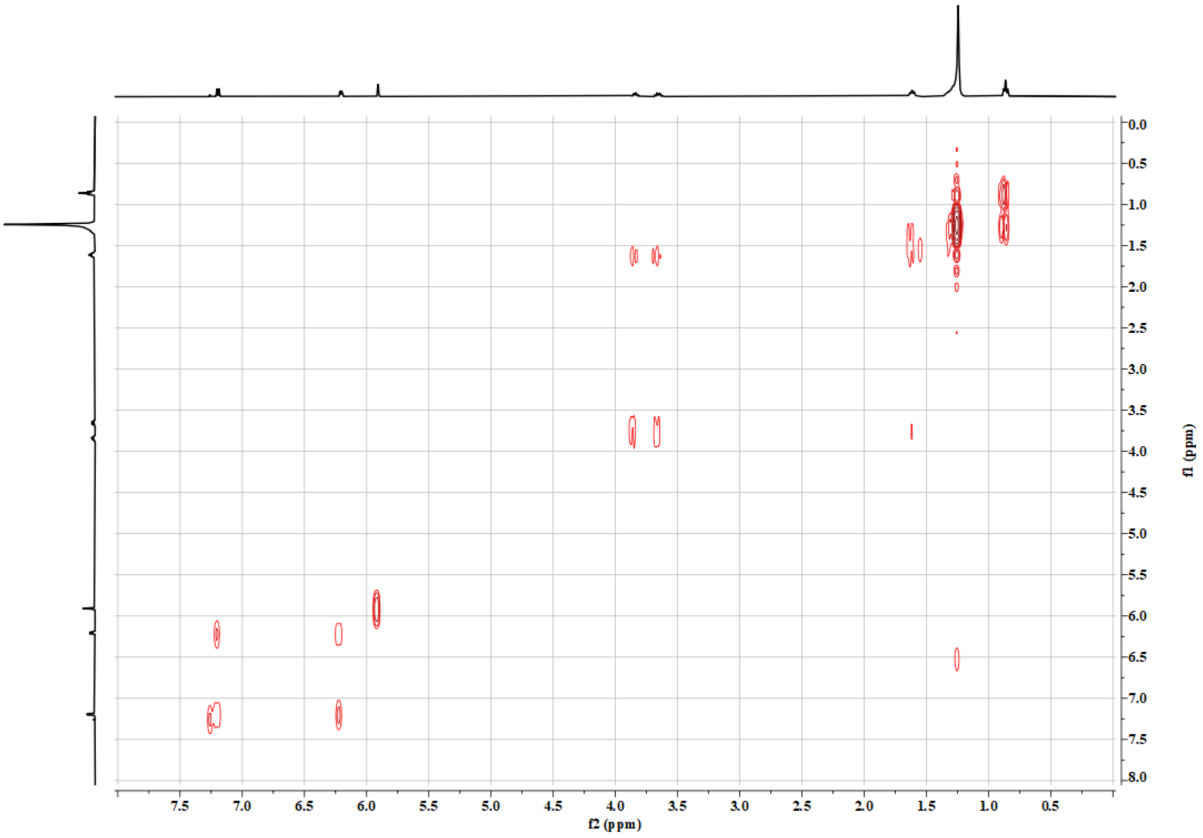

Figure S15. COSY NMR spectrum of dodecyloxy butenolide measured at 298 K in $CDCl_3$.

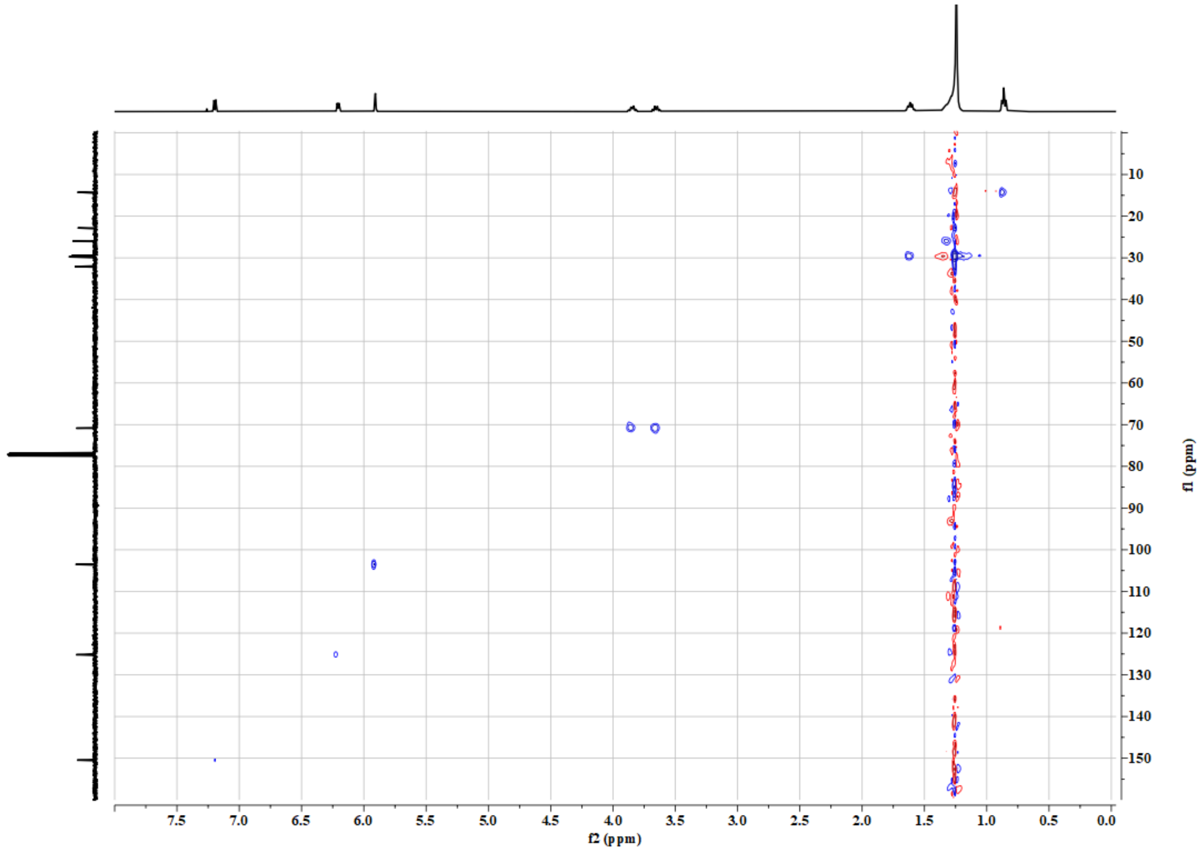

Figure S16. HSQC NMR spectrum of dodecyloxy butenolide measured at 298 K in $CDCl_3$.

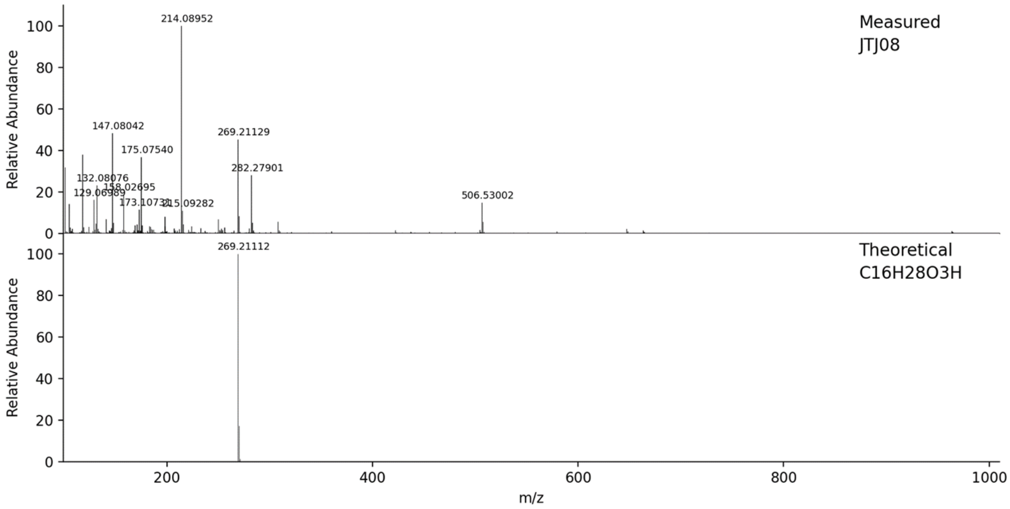

Figure S17. HRMS of dodecyloxy butenolide (top: measured, bottom: calculated), LTQ Orbitrap XL (ESI+).

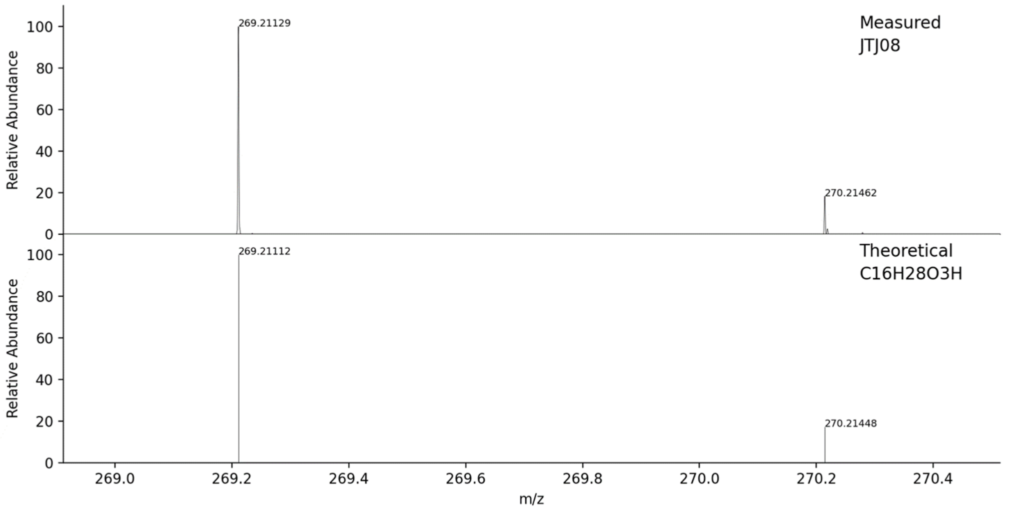

Figure S18. HRMS (zoomed in) of dodecyloxy butenolide (top: measured, bottom: calculated), LTQ Orbitrap XL (ESI+).

Citronelloxy butenolide

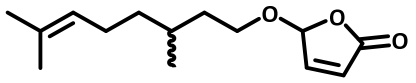

5-((3,7-dimethyloct-6-en-1-yl)oxy)furan-2(5*H*)-one
Chemical Formula: $C_{14}H_{22}O_3$
Molecular Weight: 238.33

A solution of hydroxy butenolide (2.16 g, 22 mmol, 1.1 eq.), citronellol (3.06 g, 20 mmol, 1 eq.), Amberlyst®15 (0.90 g, 30 wt%), activated molecular sieves (0.90 g, 30 wt%) in 19.6 mL dry toluene was prepared in a 250 mL three-necked flask under nitrogen. The flask was stirred at max. 200 rpm using a magnetic stirring egg (in order to prevent damaging the solid acid catalyst) at 40 °C for 22 hours at inert atmosphere. The product mixture was filtered to remove the solid catalyst and molecular sieves. The solution was concentrated *in vacuo*, yielding a yellow oil.

The crude was dissolved in diethyl ether (40 mL), and washed with $NaHCO_3$ solution (3 x 20 mL, 1 M), with water (2 x 20 mL) and with brine (1 x 20 mL). The organic layer was dried over $MgSO_4$. The solution was concentrated *in vacuo* and 5-((3,7- dimethyloct-6-en-1-yl)oxy)furan-2(5H)-one 4 (2.30 g, 10 mmol, 49.3%) was obtained as a yellow oil with a diastereomeric ratio of 14:1 (according to ^{1}H-NMR).

The diastereomers were further purified by column chromatography (silica gel, n-pentane:dichloromethane/ 50:50), yielding diastereomerically pure 5-((3,7-dimethyloct-6-en-1-yl)oxy)furan-2(5H)-one 4 (2.11 g, 9 mmol, 45.2%) as a colorless oil.

^{1}H-NMR (400 MHz, $CDCl_3$) δ 7.20 (dd, J = 5.7, 1.4 Hz, 1H), 6.23 (d, J = 5.7, 1.3 Hz, 1H), 5.92 (d, J = 1.3 Hz, 1H), 5.08 (t, J = 7.0, 1.6 Hz, 1H), 3.97 – 3.85 (m, 1H), 3.77 – 3.64 (m, 1H), 1.97 (pt, J = 14.4, 6.8 Hz, 2H), 1.74 – 1.63 (m, 4H), 1.62 – 1.50 (m, 4H), 1.50 – 1.39 (m, 1H), 1.39 – 1.27 (m, 1H), 1.24 – 1.10 (m, 1H), 0.90 (d, J = 6.6 Hz, 3H).

^{13}C-NMR (101 MHz, $CDCl_3$) δ 170.66, 150.47, 131.43, 125.07, 124.65, 103.47, 69.03, 37.13, 36.38, 29.38, 25.82, 25.46, 19.49, 17.74.

HRMS ESI-pos $[M+H]^+$ $C_{14}H_{23}O_3$ calc. 239.1642, found 239.1643

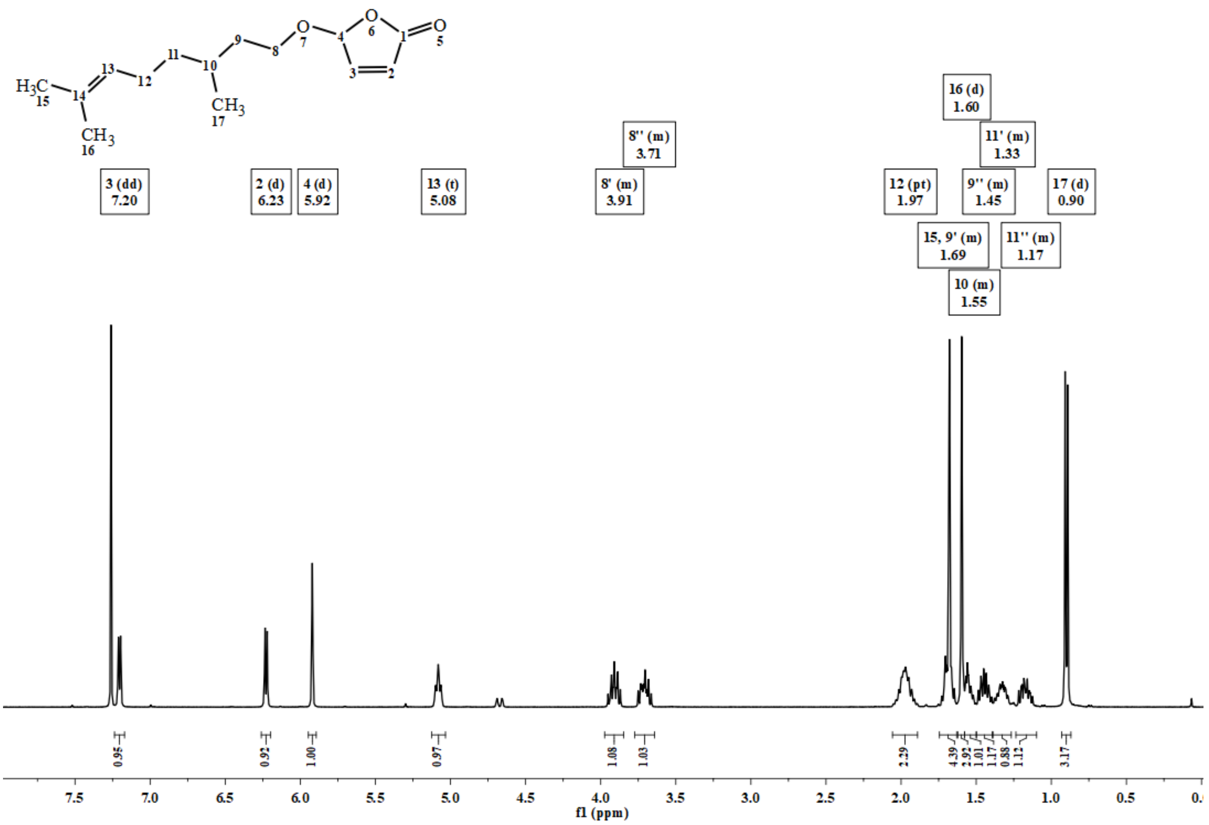

Figure S19. ^{1}H-NMR spectrum (400 MHz) of citronelloxy butenolide (diasteromerically pure) measured at 298 K in $CDCl_3$.

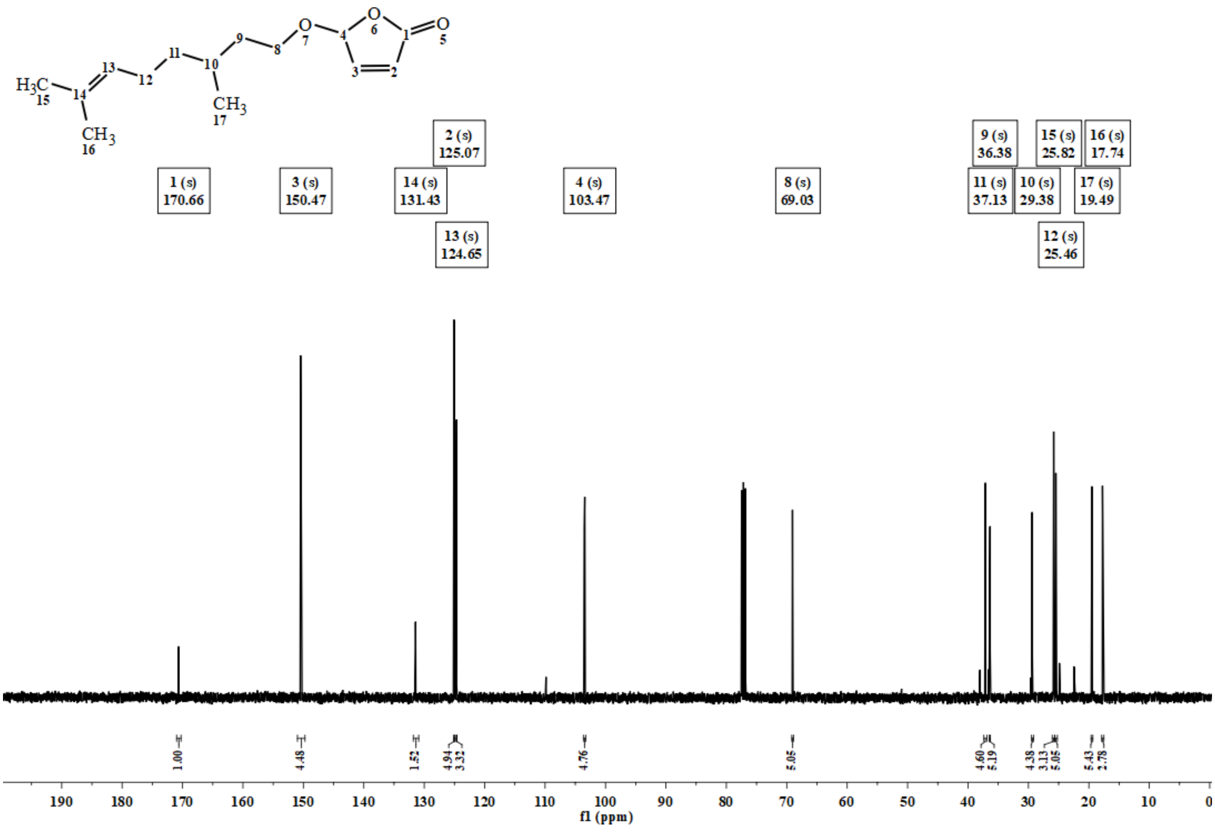

Figure S20. ^{13}C-NMR spectrum (101 MHz) of citronelloxy butenolide (diasteromerically pure) measured at 298 K in $CDCl_3$.

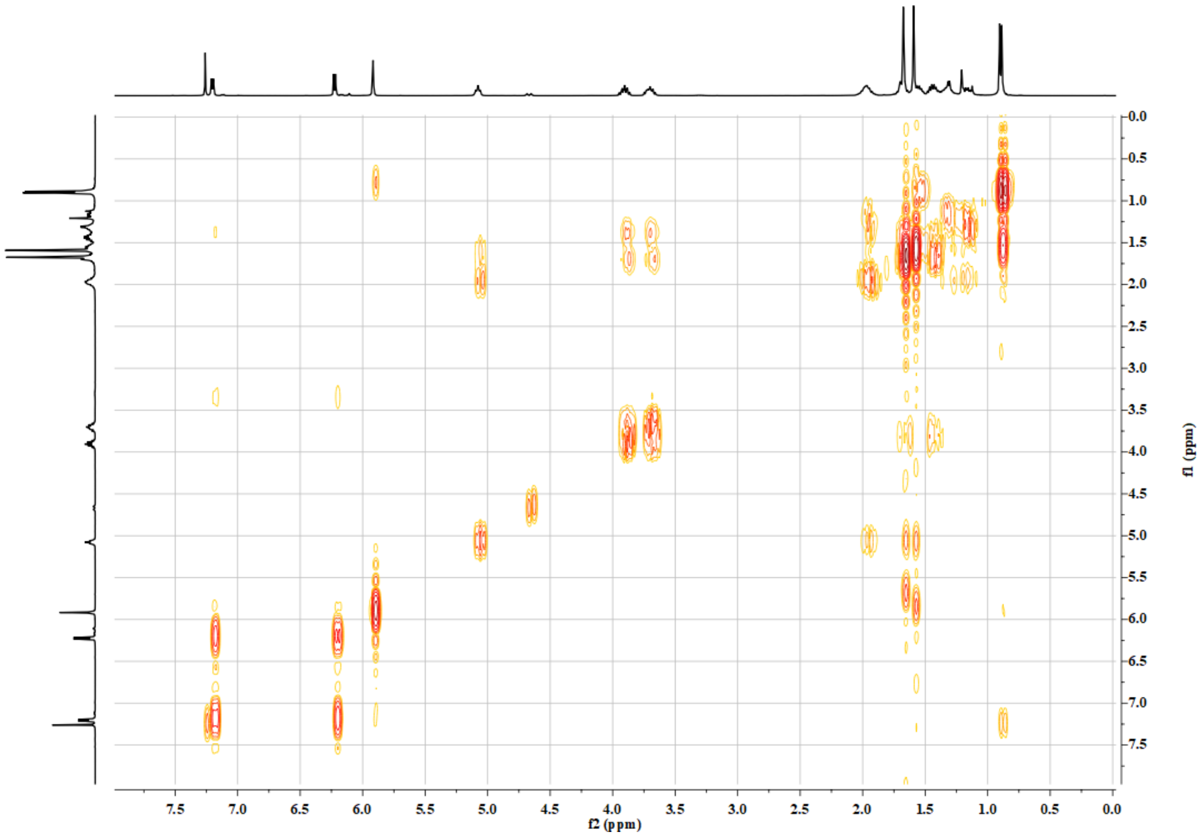

Figure S21. COSY NMR spectrum of citronelloxy butenolide (diasteromerically pure) measured at 298 K in $CDCl_3$.

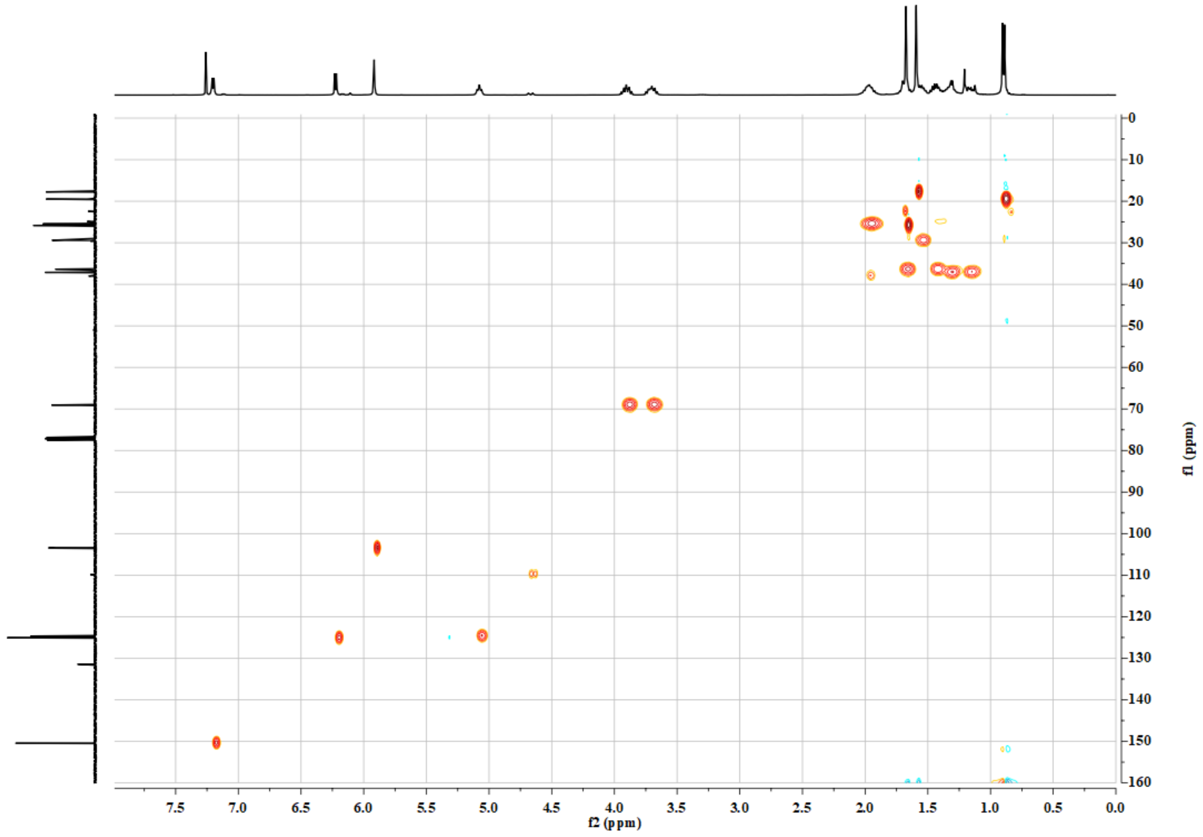

Figure S22. HSQC NMR spectrum of citronelloxy butenolide (diasteromerically pure) measured at 298 K in $CDCl_3$.

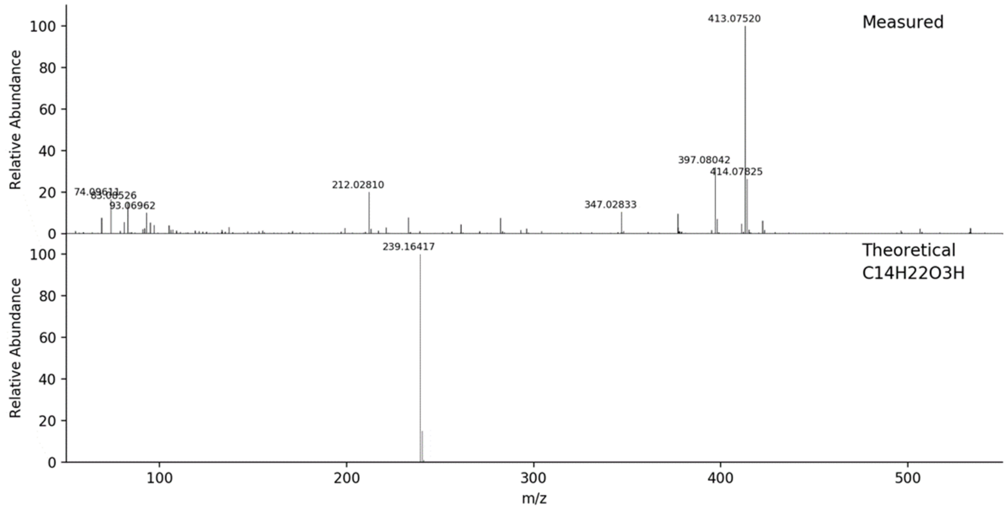

Figure S23. HRMS of citronelloxy butenolide (top: measured, bottom: calculated), LTQ Orbitrap XL (ESI+).

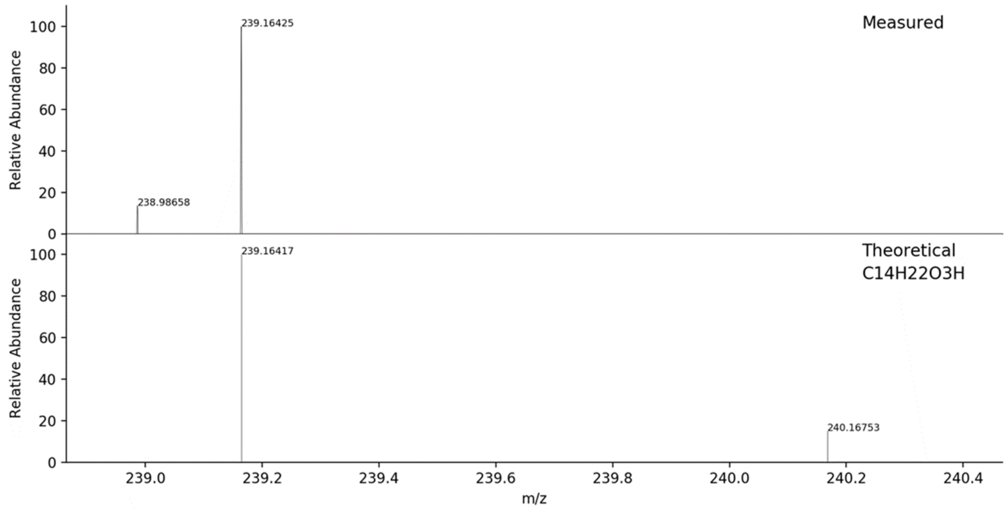

Figure S24. HRMS (zoomed in) of citronelloxy butenolide (top: measured, bottom: calculated), LTQ Orbitrap XL (ESI+).

Dihydrocitronelloxy butenolide

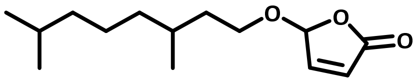

5-((3,7-dimethyloctyl)oxy)furan-2(5*H*)-one
Chemical Formula: $C_{14}H_{24}O_3$
Molecular Weight: 240.34

A solution of hydroxy butenolide (2.20 g, 22 mmol, 1.1 eq.), 3,7-dimethyloctan-1-ol (3.17 g, 20 mmol, 1 eq.), Amberlyst®15 (0.9 g, 30 wt%) and activated molecular sieves (0.9 g, 30 wt%) in 20 mL dry toluene was prepared in a 100 mL three-necked flask under nitrogen. The flask was stirred at max. 200 rpm at 40 °C overnight. The product mixture was filtered to remove the solid catalyst. The crude product dissolved in diethyl ether was washed with $NaHCO_3$ solution (3 x 20 mL, 1 M) and subsequently washed with water (2 x 20 mL) and brine (1 x 20 mL) and dried over $MgSO_4$. The solution was concentrated *in vacuo* and 5-((3,7-dimethyloctyl)oxy)furan-2(5H)-one 7 (3.16 g, 13 mmol, 65.7%) was obtained as a viscous colorless oil.

^{1}H-NMR (400 MHz, $CDCl_3$) δ 7.20 (dd, J = 5.7, 1.1 Hz, 1H), 6.23 (d, J = 1.3 Hz, 1H), 5.92 (d, J = 1.2 Hz, 1H), 3.97-3.84 (m, 1H), 3.77-3.62 (m, 1H), 1.67 (ddd, J = 14.7, 7.3, 5.3 Hz, 1H), 1.60-1.47 (m, 2H), 1.42 (ddt, 1H), 1.36-1.19 (m, 3H), 1.19-1.05 (m, 3H), 0.92-0.80 (m, 9H).

13C-NMR (101 MHz, CDCl3) δ 170.73, 150.48, 125.14, 103.52, 69.17 (d, J = 4.5 Hz), 39.34, 37.33, 36.51, 29.70, 28.09, 24.76, 22.78, 19.65 (d, J = 1.5 Hz).

HRMS ESI-pos $[M+H]^+$ $C_{14}H_{25}O_3$ calc. 241.1798, found 241.1798.

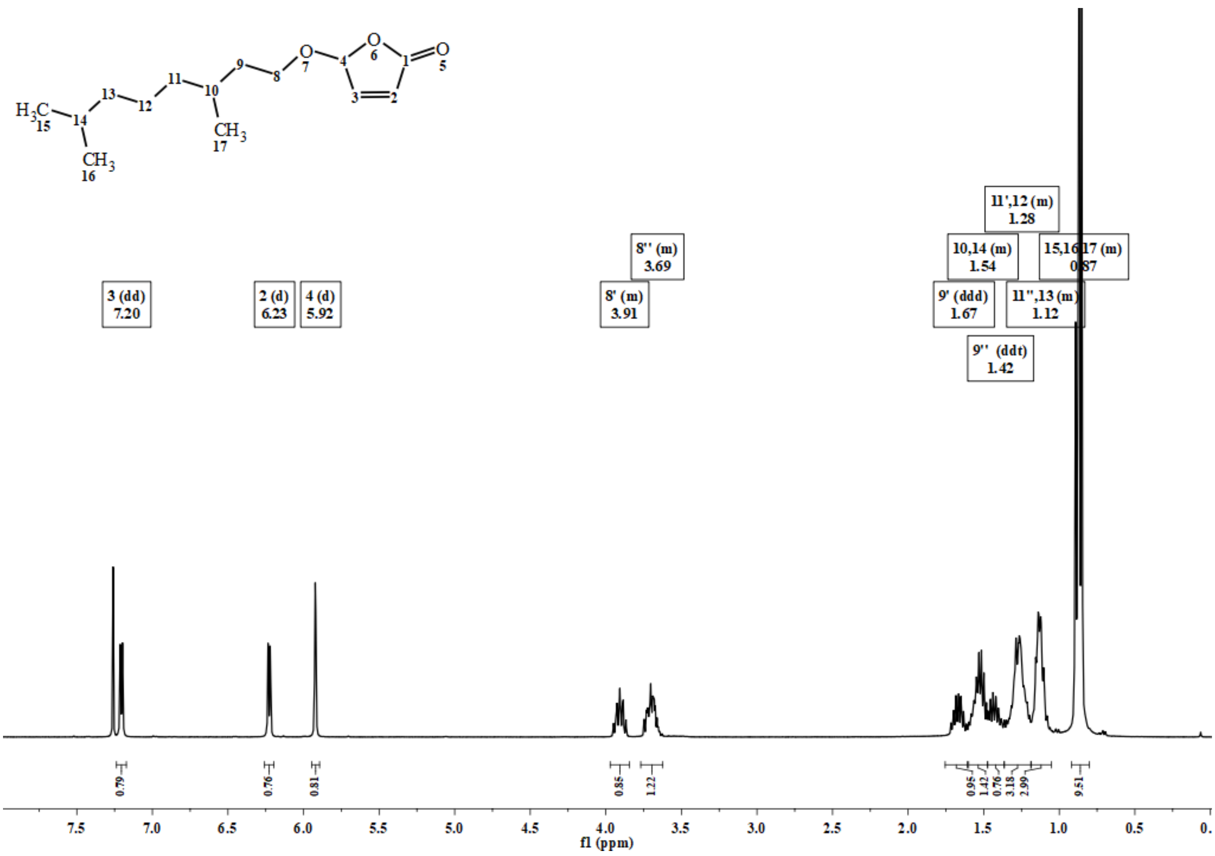

Figure S25. ^{1}H-NMR spectrum (400 MHz) of dihydrocitronelloxy butenolide measured at 298 K in $CDCl_3$.

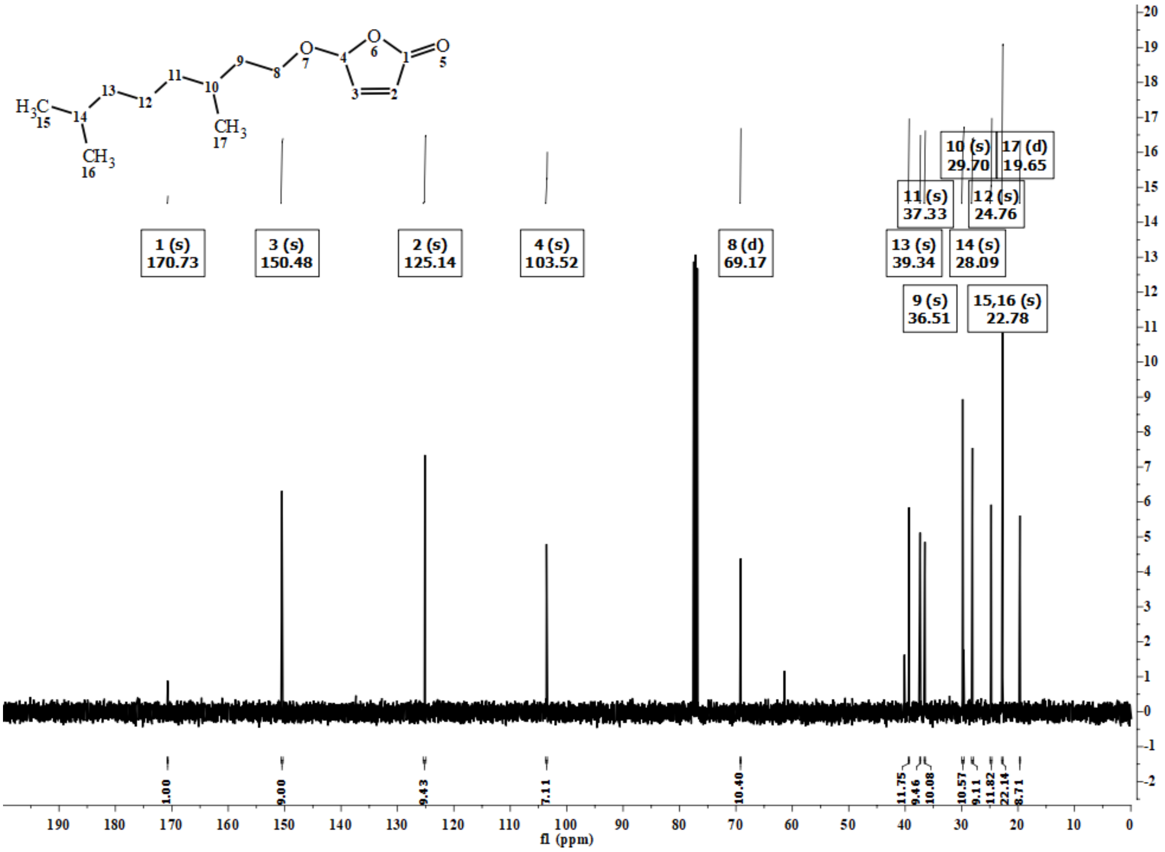

Figure S26. ^{13}C-NMR spectrum (101 MHz) of dihydrocitronelloxy butenolide measured at 298 K in $CDCl_3$.

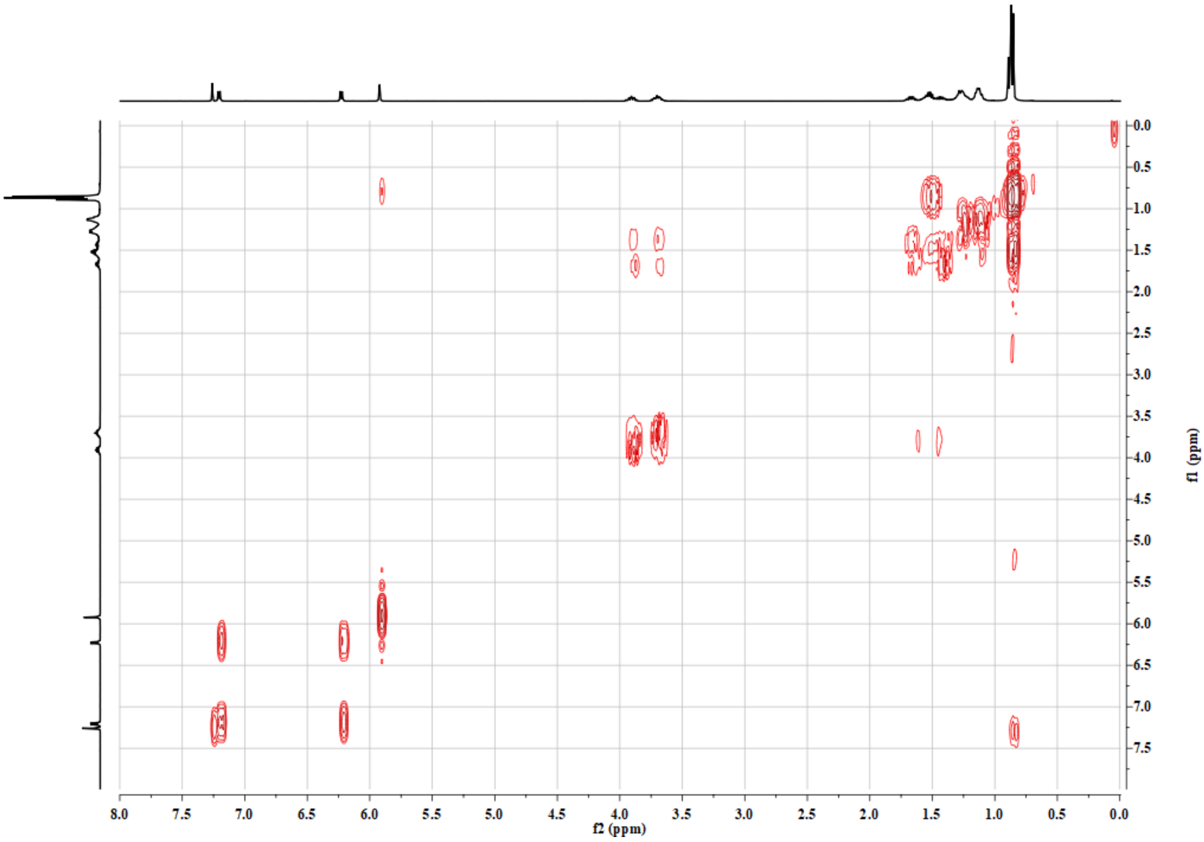

Figure S27. COSY NMR spectrum of dihydrocitronelloxy butenolide measured at 298 K in $CDCl_3$.

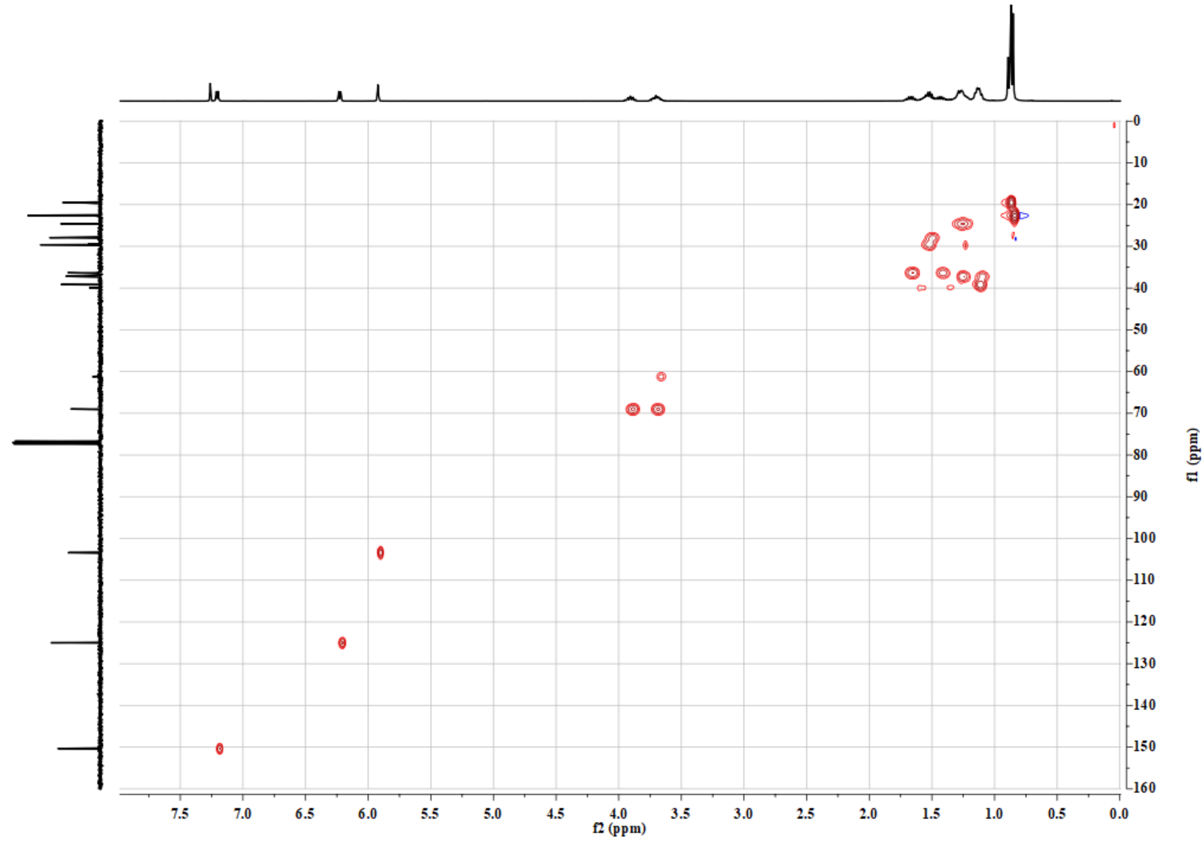

Figure S28. HSQC NMR spectrum of dihydrocitronelloxy butenolide measured at 298 K in $CDCl_3$.

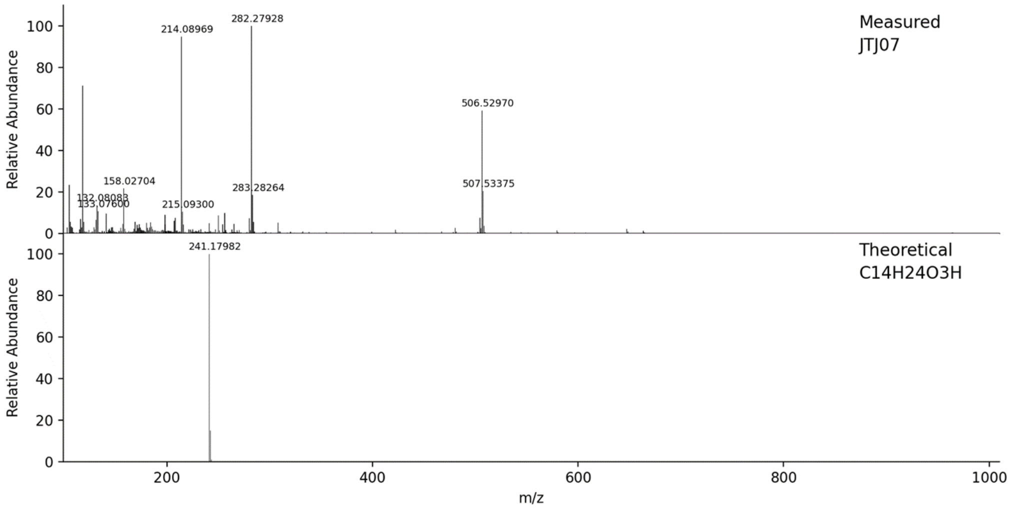

Figure S29. HRMS of dihydrocitronelloxy butenolide (top: measured, bottom: calculated), LTQ Orbitrap XL (ESI+).

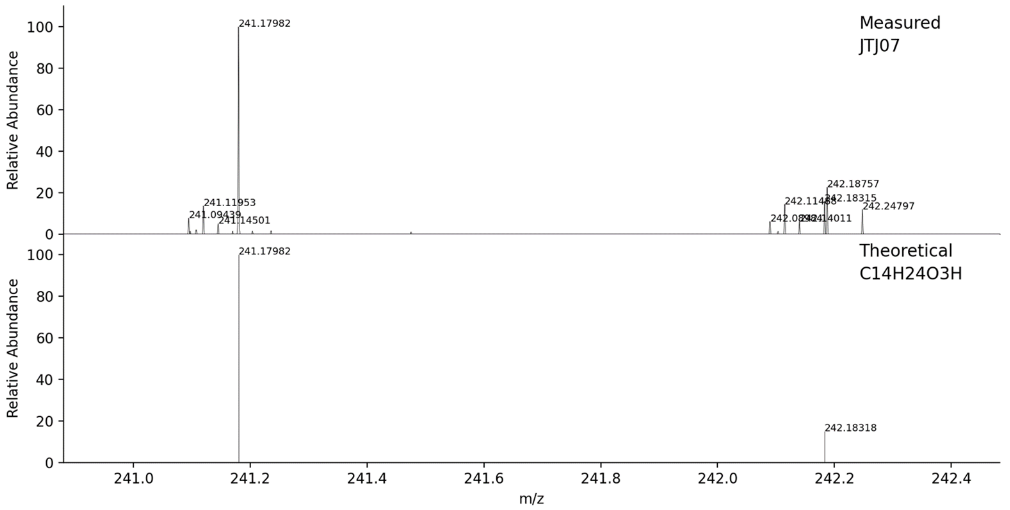

Figure S30. HRMS (zoomed in) of dihydrocitronelloxy butenolide (top: measured, bottom: calculated), LTQ Orbitrap XL (ESI+).

Phenylethoxy butenolide

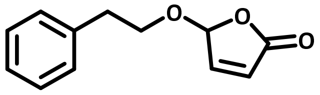

5-phenethoxyfuran-2(5*H*)-one
Chemical Formula: $C_{12}H_{12}O_3$
Molecular Weight: 204.23

A solution of hydroxy butenolide (2.16 g, 22 mmol, 1.1 eq.), 2-phenylethanol (2.39 g, 20 mmol, 1 eq.), Amberlyst®15 (0.7 g, 30 wt%) and activated molecular sieves (0.7 g, 30 wt%) in 19.6 mL dry toluene was prepared in a 100 mL three-necked flask under nitrogen. The flask was stirred at max. 200 rpm at 40 °C overnight. The product mixture was filtered to remove the solid catalyst and the solution was concentrated *in vacuo*.

The crude product was dissolved in diethyl ether (40 mL), washed with $NaHCO_3$ solution (3 x 20 mL, 1 M) and subsequently washed with water (2 x 20 mL) and brine (1 x 20 mL) and dried over $MgSO_4$. The solution was concentrated *in vacuo* and 2-phenylethoxyfuran-2(5H)-one 5 (2.71 g, 13 mmol, 67.8%) was obtained as a viscous, slightly yellow oil.

^{1}H-NMR (400 MHz, $CDCl_3$) δ 7.31 (dd, J = 8.0, 6.5 Hz, 2H), 7.27-7.19 (m, 3H), 7.16 (dd, J = 5.7, 1.2 Hz, 1H), 6.22 (d, J = 5.7, 1.3 Hz, 1H), 5.90 (d, J = 1.2 Hz, 1H), 4.10 (dt, J = 9.5, 6.9 Hz, 1H), 3.89 (dt, 1H), 2.96 (t, J = 7.1 Hz, 2H).

^{13}C-NMR (101 MHz, $CDCl_3$) δ 170.59, 150.37, 137.94, 129.05, 128.65, 126.71, 125.21, 103.45, 71.12, 36.17.

HRMS ESI-pos $[M+H]^+$ $C_{12}H_{13}O_3$ calc.205.0859, found 205.0860.

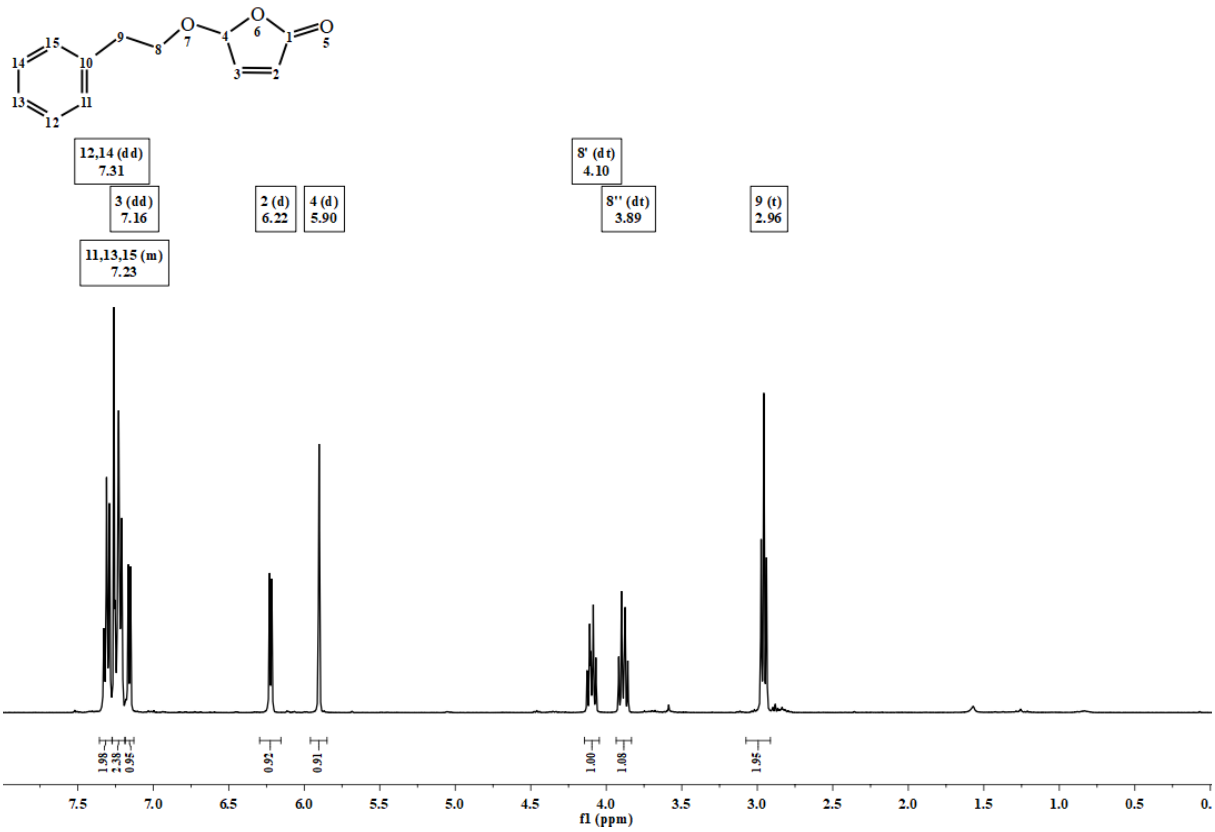

Figure S31. ^{1}H-NMR spectrum (400 MHz) of phenylethoxy butenolide measured at 298 K in $CDCl_3$.

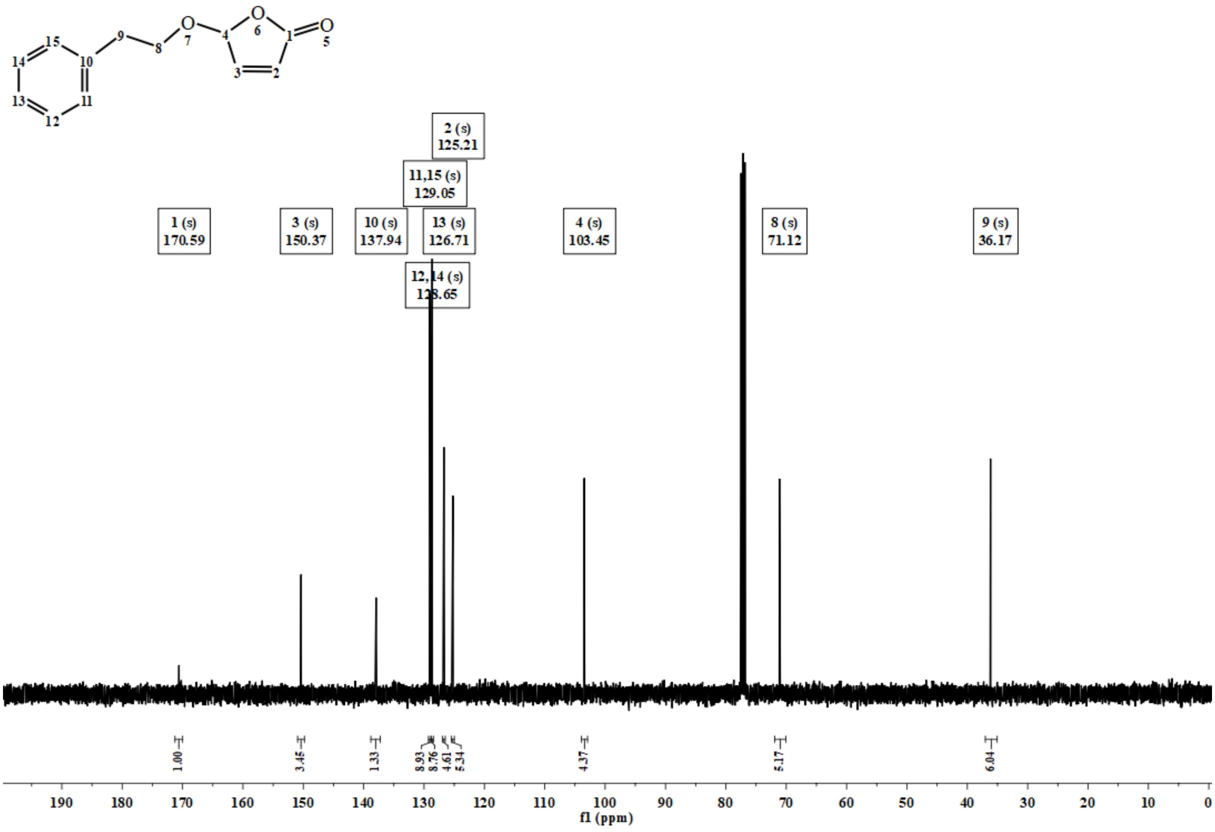

Figure S32. ^{13}C-NMR spectrum (101 MHz) of phenylethoxy butenolide measured at 298 K in $CDCl_3$.

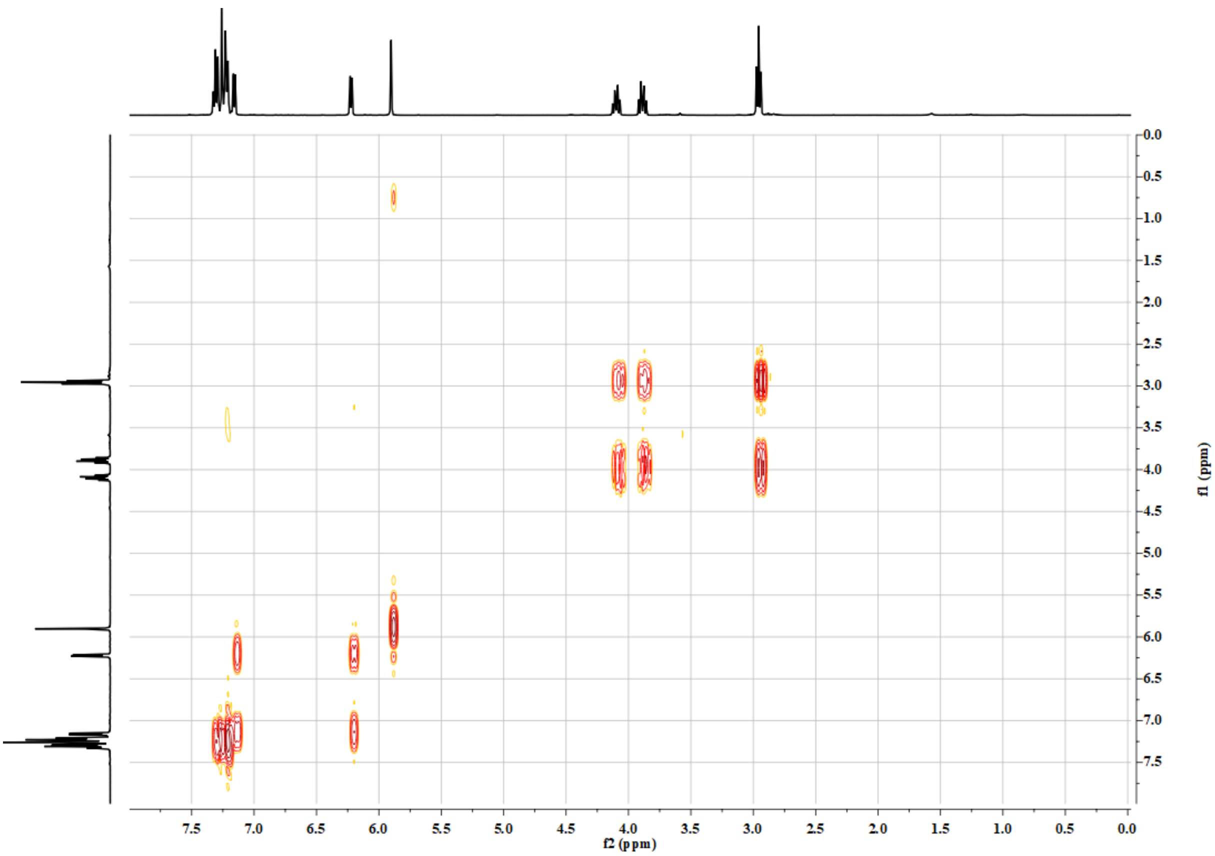

Figure S33. COSY NMR spectrum of phenylethoxy butenolide measured at 298 K in $CDCl_3$.

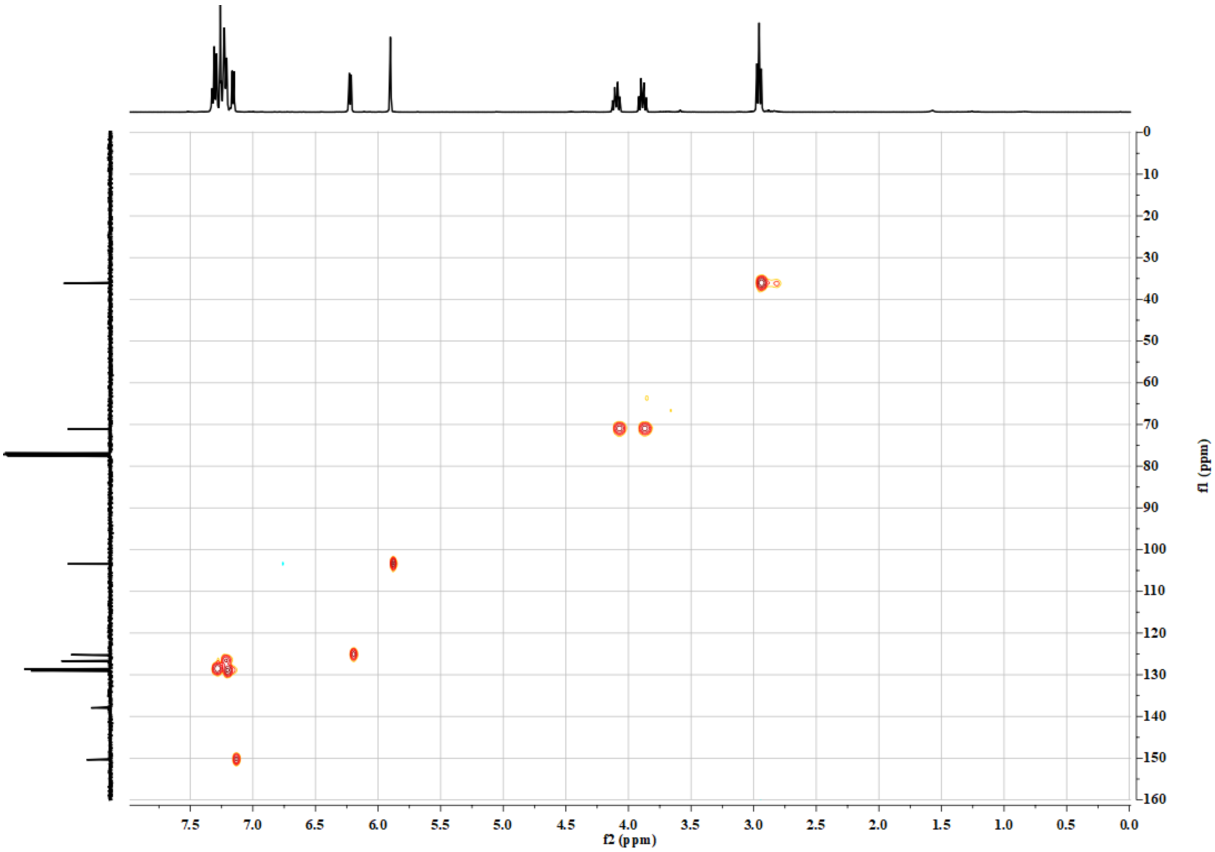

Figure S34. HSQC NMR spectrum of phenylethoxy butenolide measured at 298 K in $CDCl_3$.

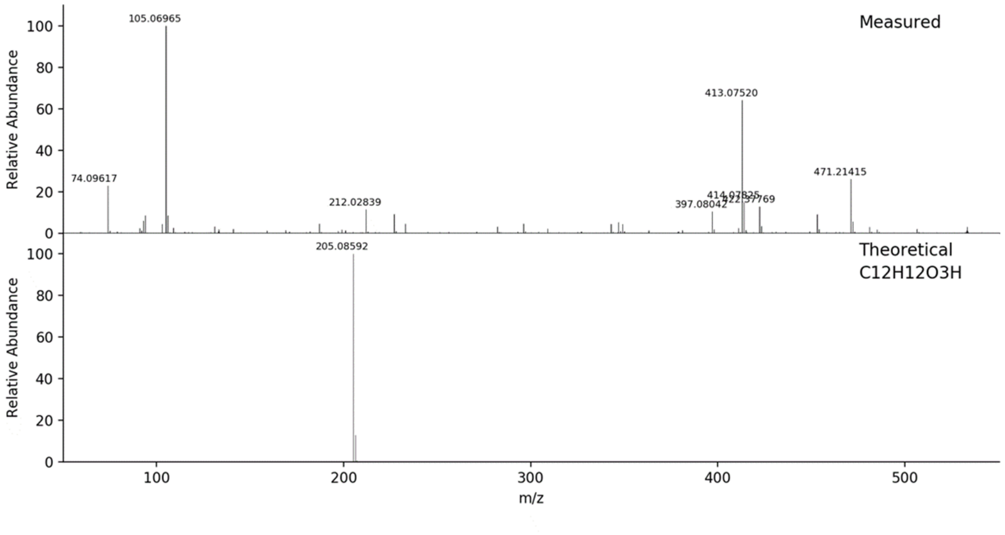

Figure S35. HRMS of phenylethoxy butenolide (top: measured, bottom: calculated), LTQ Orbitrap XL (ESI+).

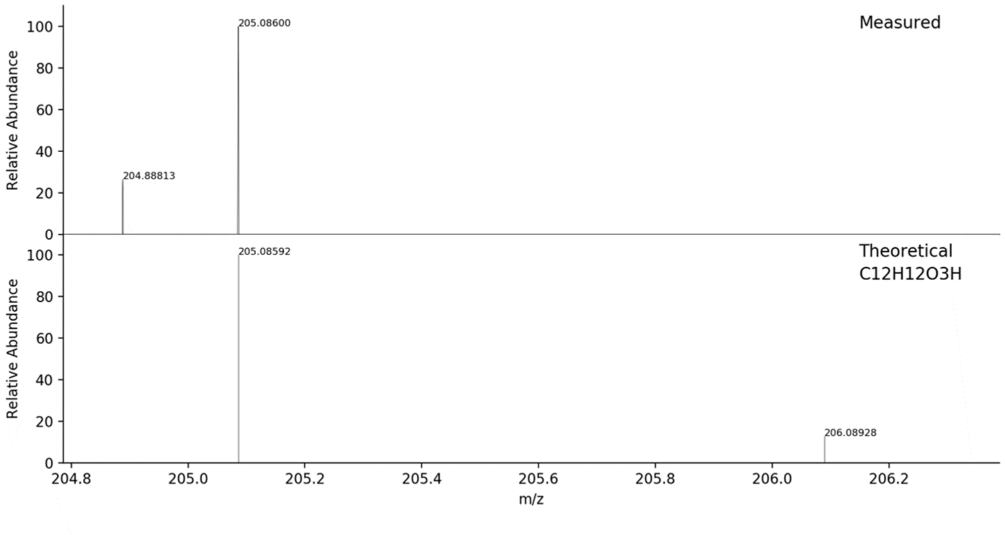

Figure S36. HRMS (zoomed in) of phenylethoxy butenolide (top: measured, bottom: calculated), LTQ Orbitrap XL (ESI+).

Menthyloxy butenolide

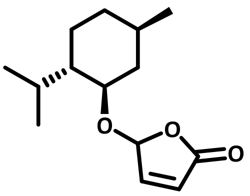

5-(((1*R*,2*S*,5*R*)-2-isopropyl-5-methylcyclohexyl)oxy)furan-2(5*H*)-one
Chemical Formula: $C_{14}H_{22}O_3$
Molecular Weight: 238.33

Hydroxy butenolide (10.00 g, 100 mmol, 1.1 eq.) and (*L*)-menthol (14.20 g, 91 mmol, 1 eq.) Amberlyst®15 (4.2 g, 30 wt%) and activated molecular sieves (4.2 g, 30 wt%) in 92 mL toluene was prepared in a 250 mL three-necked flask under nitrogen. The flask was stirred at max. 200 rpm at 40 °C overnight. The product mixture was filtered to remove the solid catalyst and the solution was concentrated *in vacuo*.

The crude product showed an impurity of unsaturated aldehyde, which was removed through stirring in a saturated solution of $NaHSO_3$ (20 mL) in diethylether (20 mL). The solution was extracted with diethyl ether (5 x 20 mL). The combined organic layers were washed with water (2 x 20 mL) and brine (1 x 20 mL), dried over $MgSO_4$ and concentrated under *in vacuo*. The obtained white solid showed two diastereomers during analysis by ^{1}H-NMR spectroscopy (ratio 52:48).

The crude product was recrystallized from *n*-heptane and washed with ice-cold *n*-heptane. The recrystallization was repeated two times to increase the yield. 5-(((1R,2S,5R)-2-isopropyl-5-methylcyclohexyl)oxy)furan-2(5H)-one 6 (5.60 g, 23 mmol, 25.9%) was obtained as a white crystalline solid.

^{1}H-NMR (400 MHz, $CDCl_3$) δ 7.16 (dd, J = 5.7, 1.2 Hz, 1H), 6.20 (d, J = 5.7, 1.2 Hz, 1H), 6.08 (d, J = 1.3 Hz, 1H), 3.66 (td, J = 10.7, 4.3 Hz, 1H), 2.20-2.03 (m, 3H), 1.74 – 1.62 (m, 1H), 1.41 (tdt, J = 12.7, 6.4, 3.4 Hz, 1H), 1.30-1.21 (m, 1H), 1.05-0.99 (m, 1H), 0.95 (d, J = 6.6 Hz, 3H), 0.88 (d, J = 7.0 Hz, 4H), 0.80 (d, J = 6.9 Hz, 3H).

^{13}C-NMR (101 MHz, $CDCl_3$) δ 170.88, 151.02, 124.94, 100.59, 79.25, 47.93, 40.47, 34.35, 31.63, 25.48, 23.30, 22.37, 21.00, 15.93.

HRMS ESI-pos $[M+Na]^+$ $C_{14}H_{22}O_3Na$ calc. 261.1461, found 261.1463.

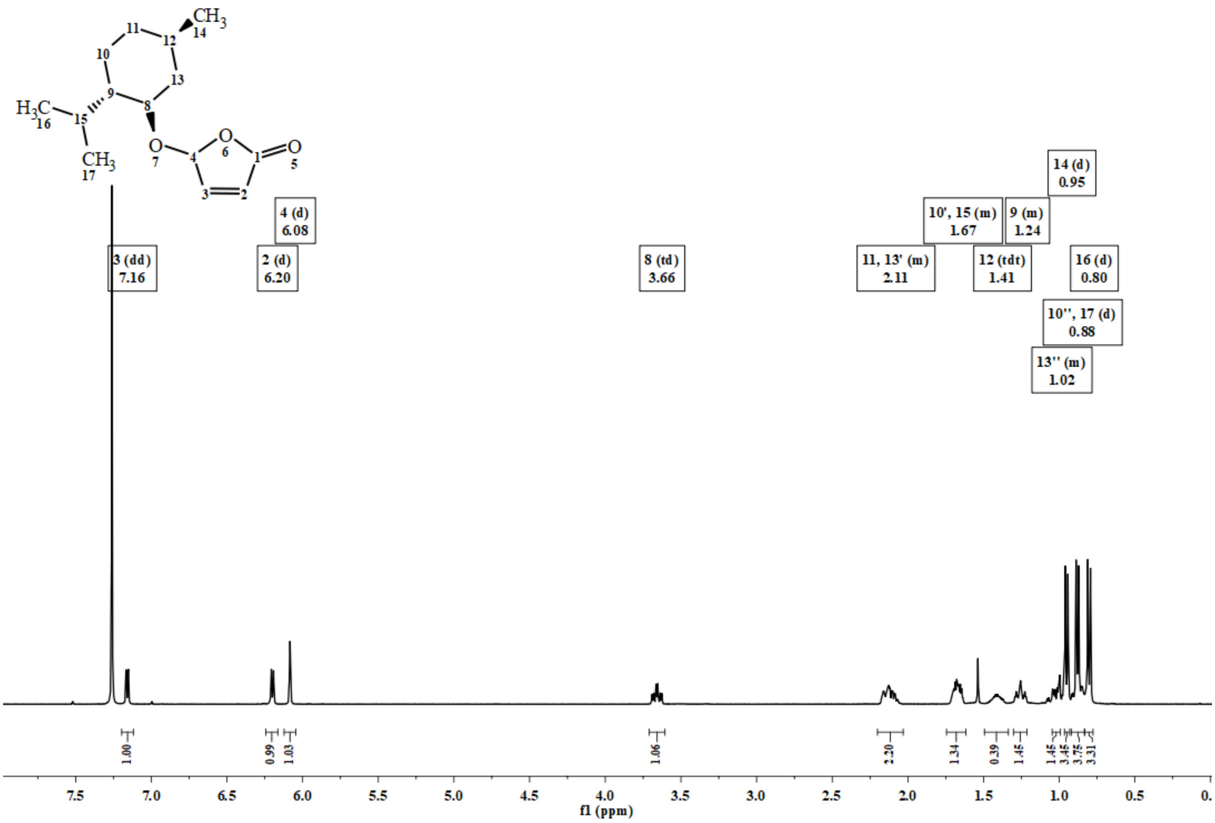

Figure S37. ^{1}H-NMR spectrum (400 MHz) of menthyloxy butenolide measured at 298 K in $CDCl_3$.

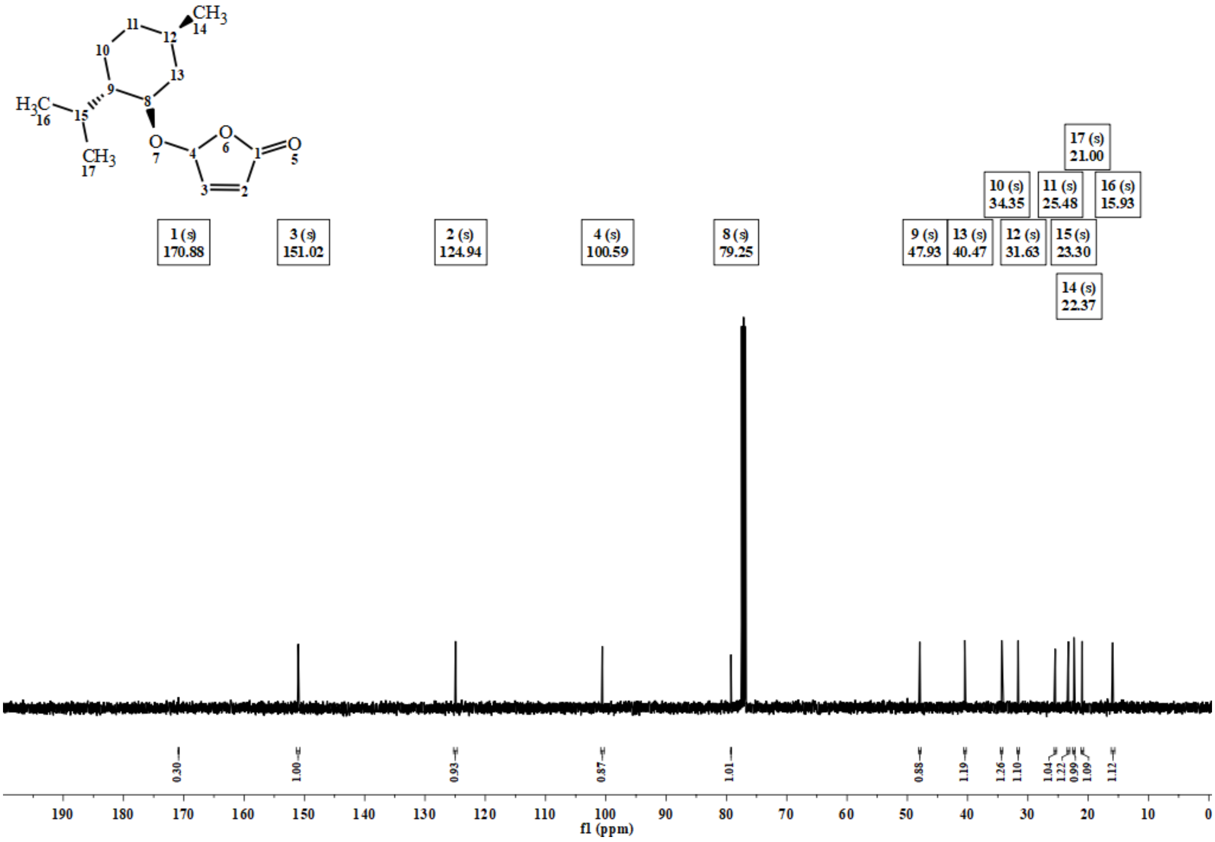

Figure S38. ^{13}C-NMR spectrum (101 MHz) of menthyloxy butenolide measured at 298 K in $CDCl_3$.

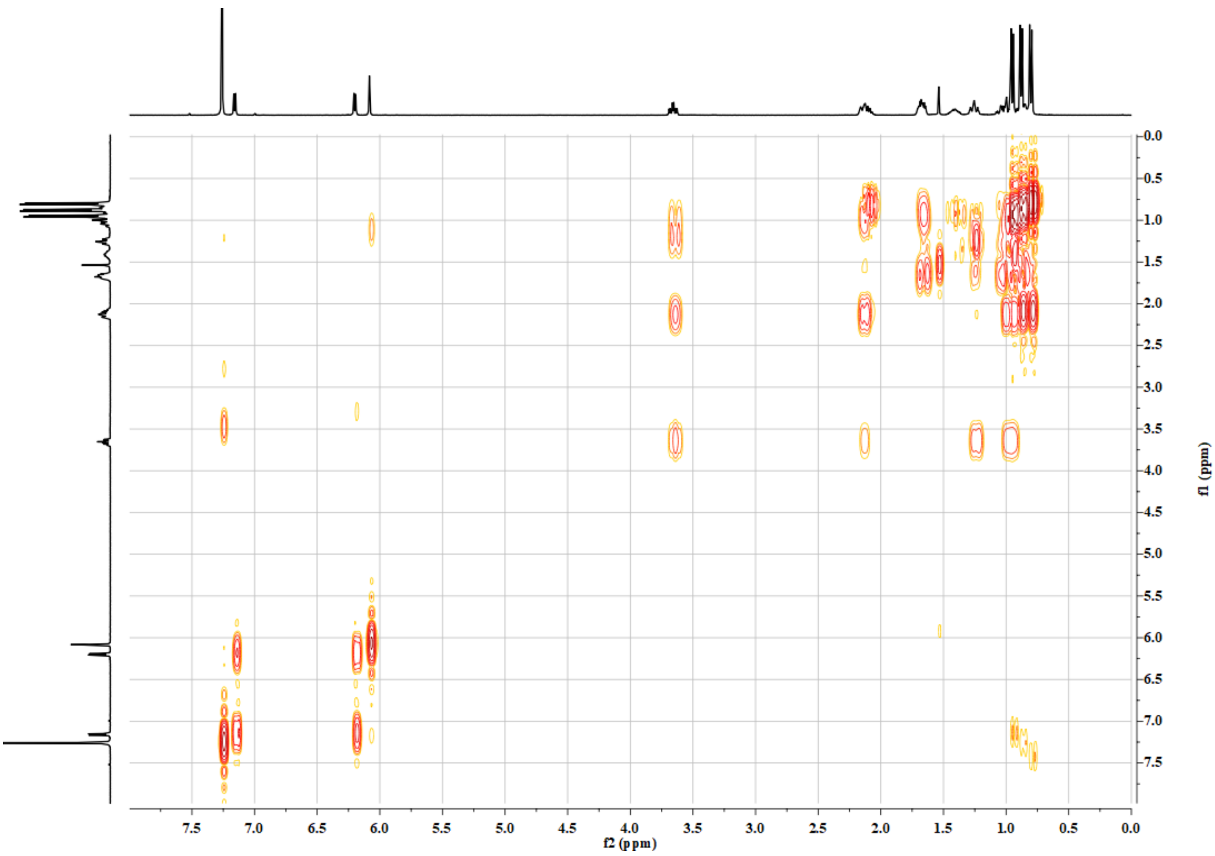

Figure S39. COSY NMR spectrum of menthyloxy butenolide measured at 298 K in $CDCl_3$.

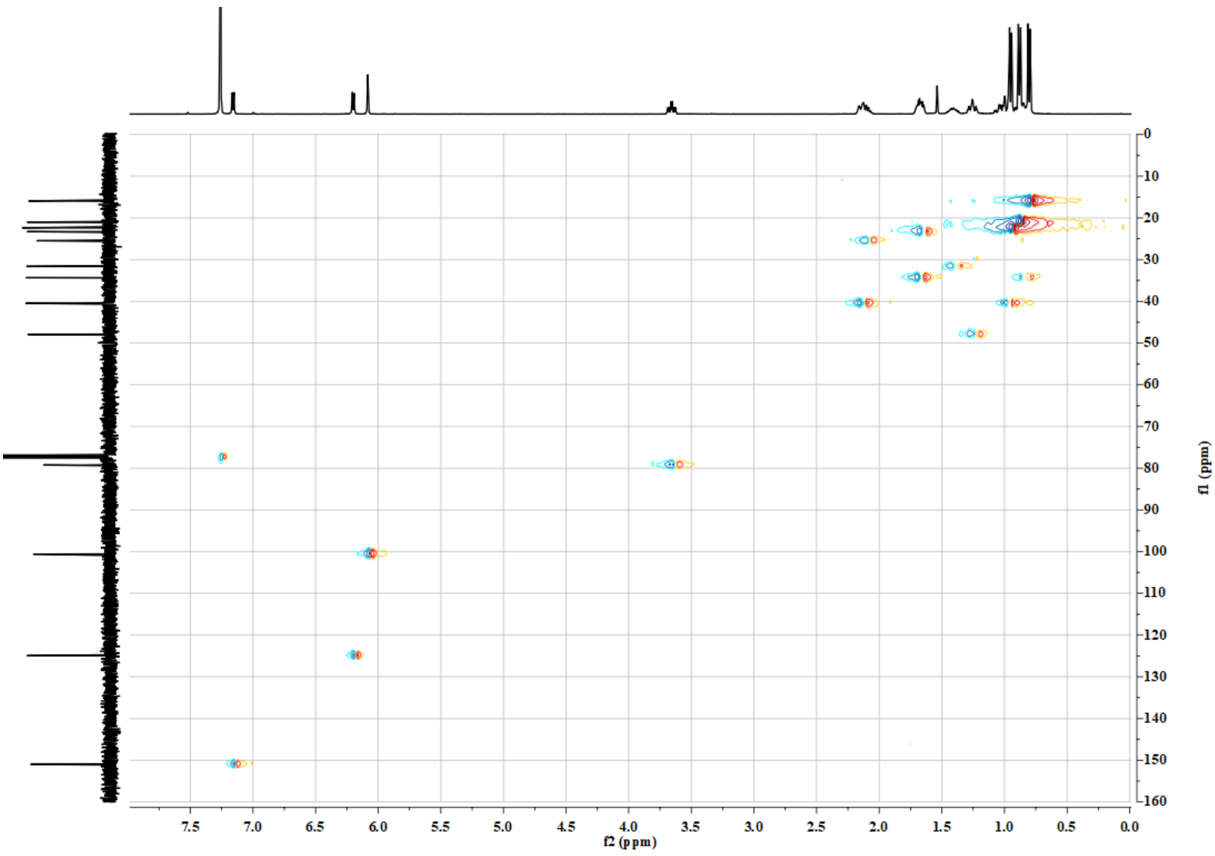

Figure S40. HSQC NMR spectrum of menthyloxy butenolide measured at 298 K in $CDCl_3$.

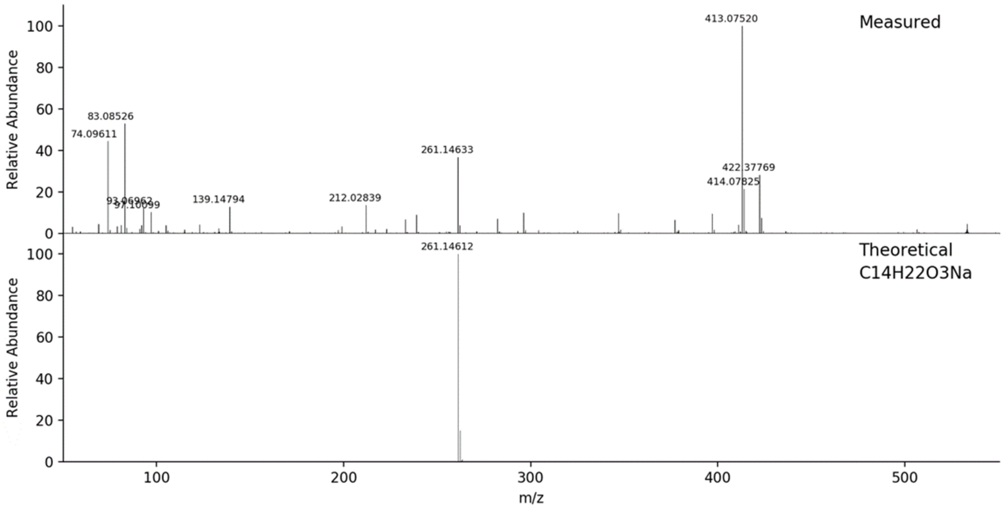

Figure S41. HRMS of menthyloxy butenolide (top: measured, bottom: calculated), LTQ Orbitrap XL (ESI+).

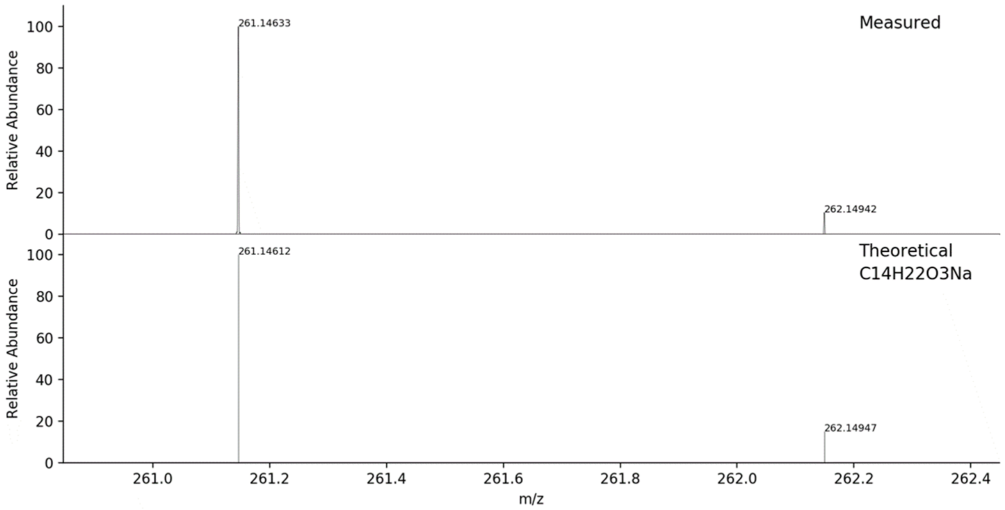

Figure S42. HRMS (zoomed in) of menthyloxy butenolide (top: measured, bottom: calculated), LTQ Orbitrap XL (ESI+).

Methods

Co-polymerizations for conversion and kinetic rate measurement

To a screw-cap 4-mL vial equipped with a 10-mm stirring bar and a septum were added alkoxy butenolide (0.876 mmol, 1 eq.), vinyl ether (0.876 mmol, 1 eq.), 1,3,5-trimethoxybenzene (0.438 mmol, 0.5 eq.) and *N*-butyl acetate (0.408 mL, 2.15 M). The mixture was homogenized and a 20 µL aliqout was diluted in an NMR tube with $CDCl_3$ (500 µL) for reference. The vial was closed and pre-heated at 120 °C for 1-2 minutes. Trigonox 42S (0.053 mmol, 0.06 eq., 3 mol% versus total monomer) was added to the mixture via a microsyringe through the septum, corresponding to t = 0. At various time points, aliquots (20 µL) were taken from the reaction mixture and diluted in $CDCl_3$ (500 µL). All samples were analyzed by ^{1}H-NMR on a 400 MHz spectrometer (typically D1 = 5, ns = 8). After correcting processed spectra for phase and baseline, integration and normalization of relevant peaks allowed monitoring of monomer conversion over time. Rates of the co-polymerization reaction were calculated according to the method described in the next section.

Note: The solvents and reagents were not degassed prior to experiments, leading to a small amount of oxygen inhibition. As we are using 3 mol% initiator (versus total monomer) we typically see inhibition within the first minute of polymerization.

Note: For NMR, the $CDCl_3$ was neutralized by flushing it through basic aluminum oxide. This prevents side-reactions in the NMR tube after sampling, where the vinyl group of the vinyl ether can undergo acid-catalyzed acetalization with nucleophiles (*e.g.*, traces of water or alcohol).

Reaction kinetics

The rate of polymerization $\boldsymbol{R_p}$ has been determined for each co-polymer by:

Equation S1. Rate of polymerization.

$$R_p = -\frac{dc_M}{dt} = k_p \cdot \left(f\frac{k_d}{k_t}\right)^{0,5} \cdot c_M \cdot (c_{ini})^{0,5}$$

Where the monomer concentration is determined by:

Equation S2. Substitution of c_M in copolymerization.

$$c_M = ([M_1] + [M_2])$$

Equation S1 is integrated from 0 to t giving:

Equation S3. Integration of rate of polymerization from 0 to t.

$$\ln\frac{c_M^0}{c_M} = k_p\left(f\frac{k_d}{k_t}c_{ini}\right)^{0,5} \cdot t$$

The conversion of copolymerization is determined by:

Equation S4. Conversion of polymer.

$$U = \frac{c_M^0 - c_M}{c_M^0}$$

Substituting ***U*** in equation S3 and combining the constants and variables we obtain:

Equation S5. Rate of polymerization as a function of t.

$$\ln\left(\frac{1}{1-U}\right) = k_{obs} \cdot t$$

By plotting $\ln\left(\frac{1}{1-U}\right)$ against t (equation S5) it is possible to obtain the rate constant k_{obs} in s^{-1}. This rate constant is a combination of different parameters, which are specific to reaction and reaction conditions. It includes:

$$k_p\left(\frac{L}{mol\ s}\right) = \text{rate constant for propagation}$$

$$k_d(s^{-1}) = \text{rate constant for dissociation}$$

$$k_t\left(\frac{L}{mol\ s}\right) = \text{rate constant for termination}$$

Additionally, it is assumed that the radical concentration c_{ini} is constant and does not change over time. A substance specific correction value f is included as well. Without specific determination of the different rate constants (k_p, k_d, k_t), the relative reactivity between the different butenolides and the reactivity between butenolides and commonly used monomers can be compared through the comparison of the value for k_{obs}, provided that these values are obtained under the same reaction conditions.

Co-polymerization for molecular weight distribution and glass transition temperature

To measure the molecular weight distribution (Mn, Mw and PDI) and glass transition temperature (Tg) of the co-polymers prepared, copolymerization reactions were performed as previously described, but without internal standard. The remaining *n*-butyl acetate was removed by rotatory evaporation under strong vacuum at 70-80 °C. The dried polymers were then analyzed by gel permeation chromatography (GPC) in THF or DMF (10-20 mg/mL), with detection by refraction index against a polystyrene calibration. Injection volume was 20 µL for samples in THF and 45 µL for DMF.

The glass transition temperature (T_g) was measured by differential scanning calorimetry (DSC) on Heat-Cool-Heat cycles between temperatures ranging from – 75 °C to 150 °C at 10 °C/min. The T_g was determined from the 2nd heating ramp.

General procedure for UV-curing of coatings

To a screw-cap 4-mL vial were added alkoxy butenolide (2 mmol, 1eq.), ethylene glycol vinyl ether (1.4 mmol, 0.7 eq.), di(ethylene glycol) divinyl ether (0.3 mmol, 0.15 eq.) and phenylbis(2,4,6-trimethylbenzoyl)phosphine oxide (Omnirad 819 or BAPO) (0.06 mmol, 0.03 eq.). The mixture was homogenized and briefly heated if needed (*e.g.*, in case of insoluble

monomers). The mixture was applied uniformly on a glass plate (100 μm) using a Byk applicator. The glass surface (10 x 20 cm) was irradiated with λ = 395 nm (UV Flood 36. 12 x 3 W) at a 5 cm distance for 20 min.

Extended Discussion on release and the analysis of released alcohols

Quantification of release from polymers and coatings

Acid-mediated release of alcohols from butenolide polymers and coatings were performed. Samples were prepared consisting of alkoxy butenolide co-polymers and an aqueous solution (water, 10 mol% HCl, and 100 mol% HCl). The samples were shaken by an orbital shaker (Fisherbrand™ Multi-Platform Shaker, model 88861022). All release experiments were performed in sealed 20 mL headspace vials under orbital shaking (400 rpm) at room temperature. This method was chosen over magnetic stirring to handle viscous copolymers, maintain uniform agitation across parallel vials, and prevent bar–polymer contact.

Initially, quantification of alcohol release was attempted using ^{1}H-NMR in DMSO-d_6. However, this was not feasible due to significant overlap between the signals of the released alcohol and the polymer. When switching to gas chromatography-mass spectrometry (GC-MS) inconsistent results were acquired, due to severe tailing and carry-over originating from polymer build-up in the inlet liner and interactions between non-volatile polymer and released alcohols. The tailing of the released alcohols was attributed to interactions between polymer and released alcohol molecules. The polymer solutions used for analysis contained polymer, released odor molecules, and solvent. In GC–MS, a liquid sample (1 μL) is taken from the prepared vial and injected into the inlet liner, where it is vaporized before entering the column. The liner contains glass wool, which promotes vaporization by providing a high surface area and prevents non-volatile compounds from reaching the column. The polymers produced are non-volatile and adhere to the wool. The released odor molecules can then interact with the polymer trapped in the wool. Both the polymer and the released odor molecules contain free hydroxyl groups, allowing dipole–dipole (hydrogen-bonding) interactions between them. These interactions prevent the odor molecules from eluting at a single retention time, leading to pronounced peak tailing, signal stacking, and carry-over into subsequent measurements. As a result, the carry-over of odor molecules prohibits accurate quantification of the release.

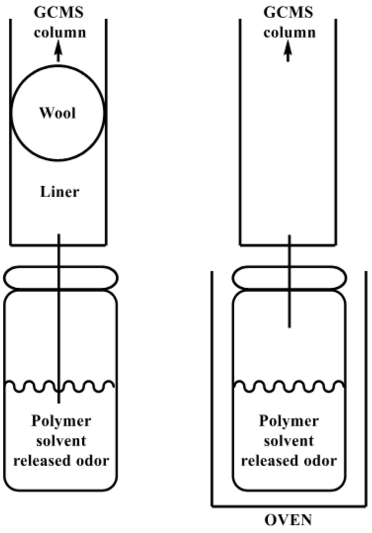

Figure S43. Gas chromatography-mass spectrometry (left) versus headspace gas chromatography-mass spectrometry (right).

Switching to headspace GC-MS, which samples only the volatile headspace and avoids contact with the polymer fraction, resolved these issues and yielded reproducible quantification. For example, in a typical measurement (3-EGVE after 20 days mixing in water) one can quantify the amount of alcohol released by integrating peak area. Since the alcohol concentration in the headspace is in equilibrium with that in the solution, calibration curves with the respective alcohol were established. The peak area could then be correlated with the total amount of alcohol present in the solution. To prevent carry-over between measurements, a blank run was included after each sample, confirming the absence of residual alcohols—an issue previously observed with conventional GC–MS.

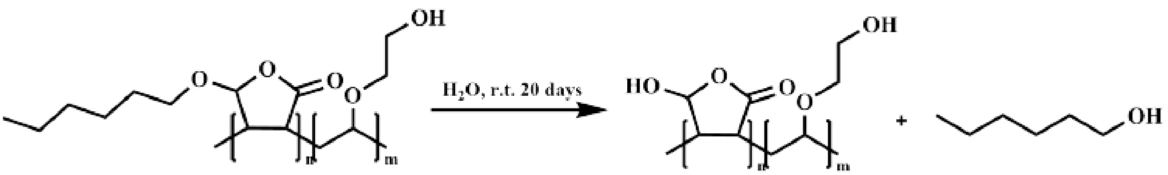

3-co-EGVE released 3-co-EGVE hexanol

(a)

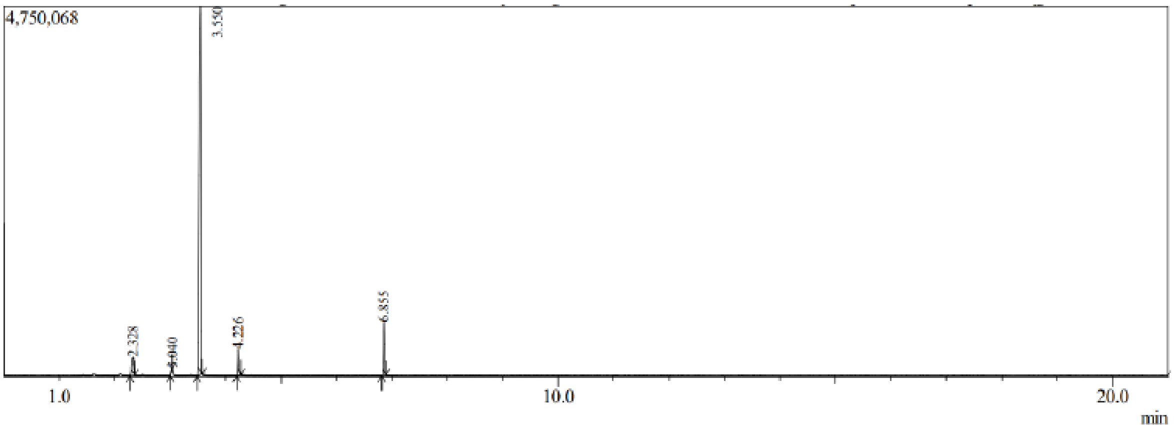

Peak Report TIC

Peak#	R.Time	Area	Area%	Height	Height%	Name	Base m/z	Base Int.
1	2.328	578196	4.17	230008	3.85	Pentane, 2,2,4-trimethyl-	57.05	144080
2	3.040	14761	0.11	0	0.00		70.10	79
3	3.550	11843317	85.39	4725301	79.08	Acetic acid, butyl ester	56.10	2035268
4	4.226	480216	3.46	335342	5.61	1-Hexanol	56.10	143099
5	6.855	953537	6.87	685065	11.46	Ether, 2-ethylhexyl tert-butyl	57.10	325757
		13870027	100.00	5975716	100.00			

(b)

Figure S44. Hexanol release from 3-*co*-EGVE measured by headspace GC-MS after 20 days mixing in water. (a) Reaction scheme of release from hexyloxy butenolide-*co*-EGVE copolymer. (b) Headspace GC chromatogram.

General procedure for release of alcohols from monomers

Alkoxy butenolide (5 mmol) and an aqueous HCl solution (5mL, 100/10 % H^+ compared to the alkoxy butenolide) or H_2O (5 mL, pH 6.8) were added into a headspace GC-MS vials (20 mL). The vial was sealed with a screw cap with septum and shaken using an orbital shaker at r.t. for 10 d. An aliquot (20 µL) was taken at various time points, and diluted in DMSO-d_6 (500 µL). The samples were analyzed by ^{1}H-NMR on a 400 MHz spectrometer (typically D1 = 5, ns = 8).

General procedure for release of alcohols from polymers/coatings

Alkoxy butenolide-co-vinyl ether copolymer/coating (2 mmol) and an aqueous HCl solution (5mL, 100/10 % H^+ compared to the alkoxy butenolide) or H_2O (5 mL, pH 6.8) were added to a headspace GC-MS vial (20 mL). The vial was sealed with a screw cap with septum and shaken using an orbital shaker at r.t. for 2/5/10/20/40 days. The alcohol quantity was measured using headspace GC-MS. The headspace GC-MS measurements were conducted at an oven temperature and injection temperature of 30/50/80 °C, depending on the volatility of the released alcohol.

Note: the coating was removed from the glass plate and added to the headspace GC-MS vial.

Co-polymerization kinetics data

The reaction kinetics of copolymerization of the different alkoxy butenolides and vinyl ethers were determined using ^{1}H-NMR spectroscopy, according to the method previously described on page S34. The concentrations of the monomers were followed during the reaction by integrating the ^{1}H-NMR signals of the alkoxy butenolide and the vinyl ether and normalizing the integrations against the internal standard(1,3,5-trimethoxybenzene). The followed NMR shifts for the internal standard and the monomers are as follows:

Table S1. Followed ^{1}H-NMR shifts in polymerization kinetic measurements.

Compound	^{1}H-NMR shift
1,3,5-Trimethoxy benzene	δ 6.08 (3H)
Ethoxy butenolide	δ 6.21 (1H)
Hexyloxy butenolide	δ 6.22 (1H)
Dodecyloxy butenolide	δ 6.46 (1H)
Citronelloxy butenolide	δ 6.23 (1H)
Dihydrocitronelloxy butenolide	δ 6.22 (1H)
Phenylethoxy butenolide	δ 6.22 (1H)
Menthyloxy butenolide	δ 6.19 (1H)
Dodecyl vinyl ether	δ 6.46 (1H)
Ethylene glycol vinyl ether	δ 6.50 (1H)

Poly(ethoxy butenolide-co-dodecyl vinyl ether) (1-DVE)

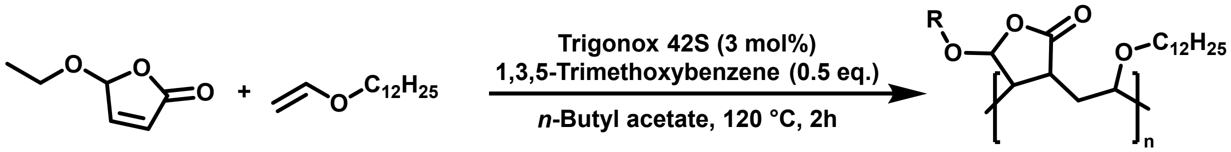

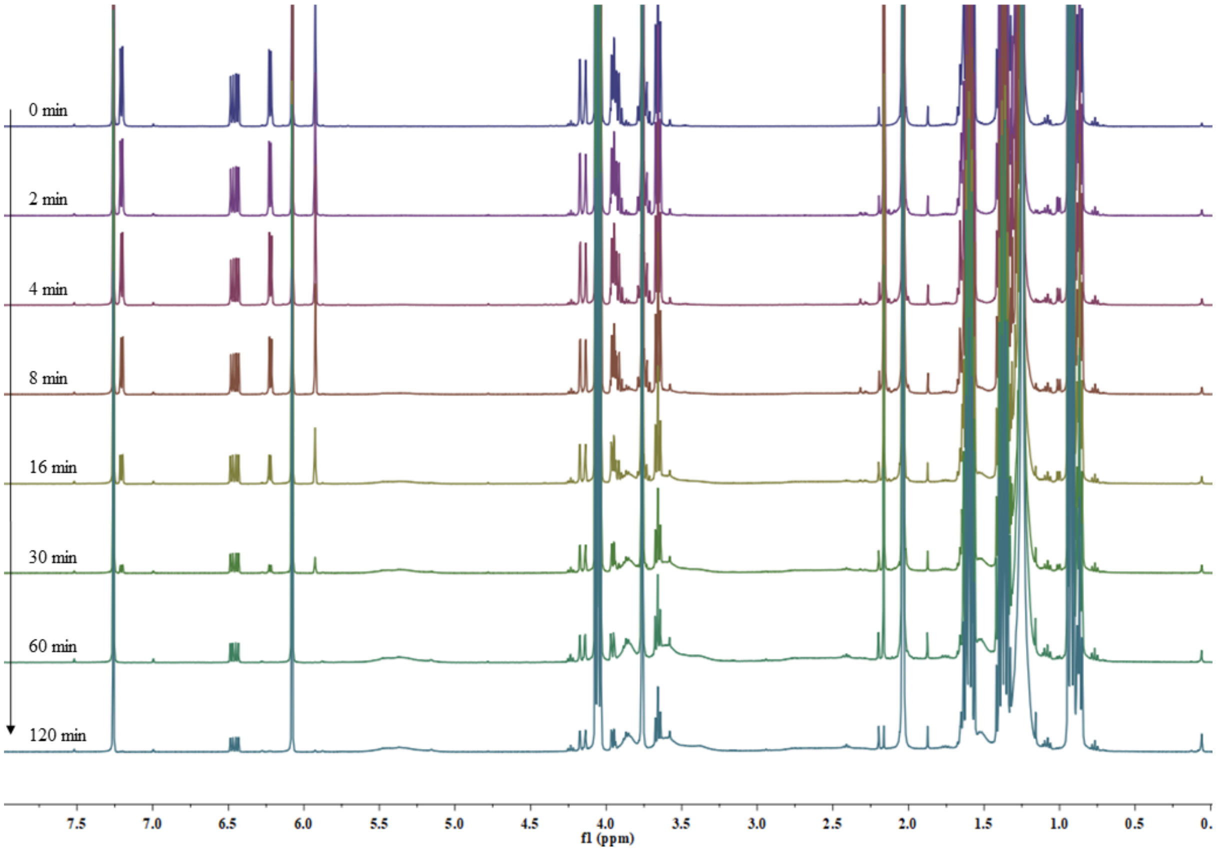

Figure S45. ^{1}H-NMR spectra of the co-polymerization of ethoxy butenolide with DVE over time.

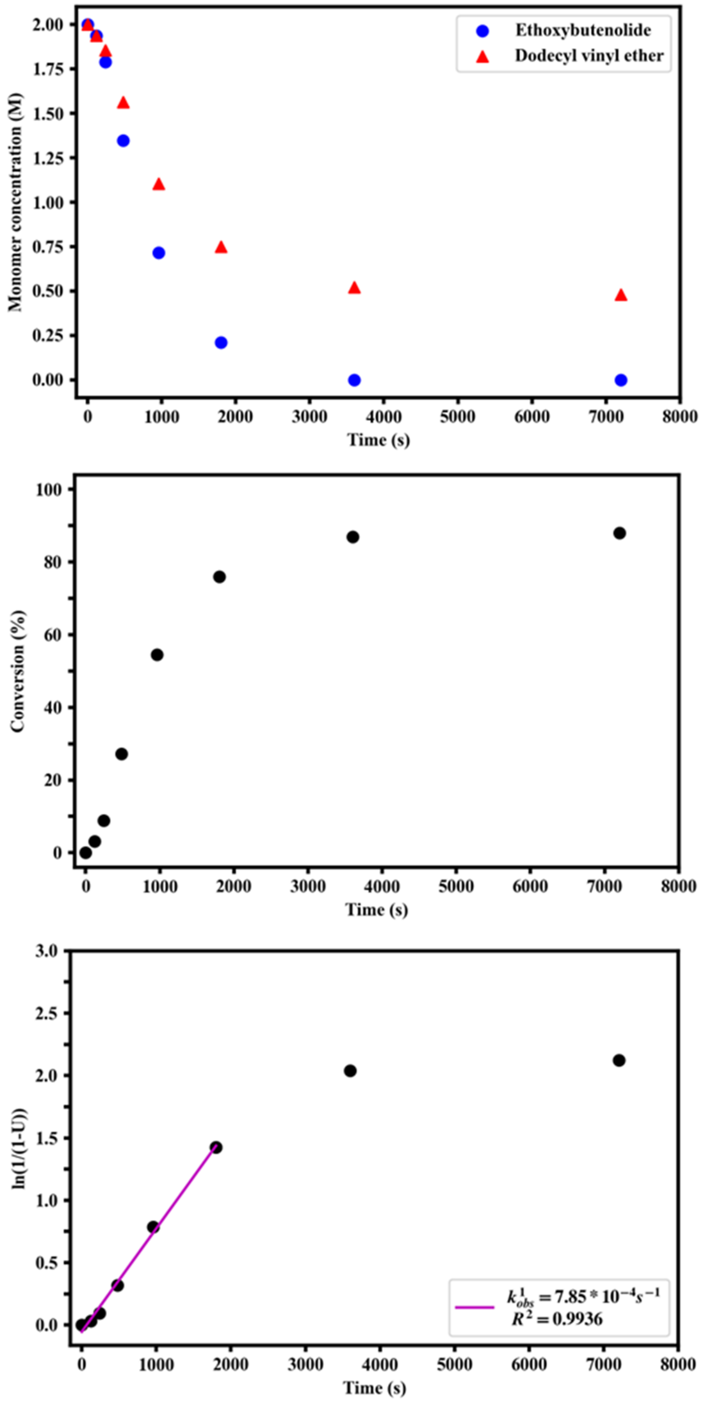

Figure S46. Kinetics of the copolymerization using ethoxy butenolide and DVE followed by ^{1}H-NMR spectroscopy at regular intervals throughout the reaction. Top: concentration of monomers over time. Middle: conversion of monomers over time. Bottom: initial rate of copolymerization.

Poly(ethoxy butenolide-co-ethylene glycol vinyl ether) (1-EGVE)

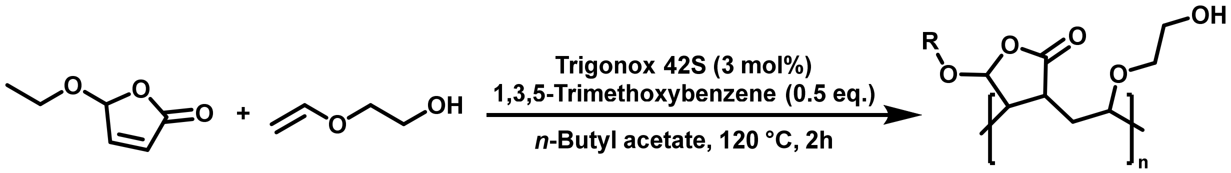

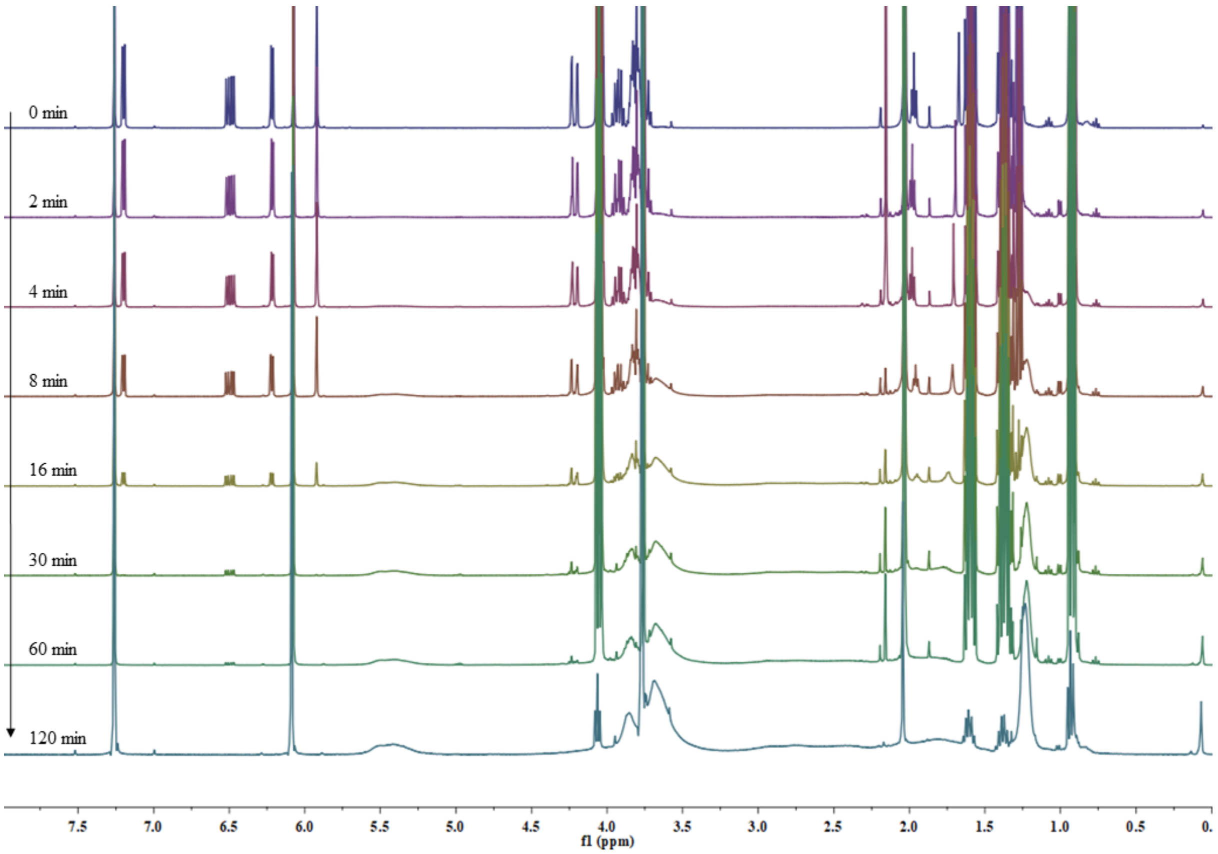

Figure S47. ^{1}H-NMR spectra of the co-polymerization of ethoxy butenolide with EGVE over time.

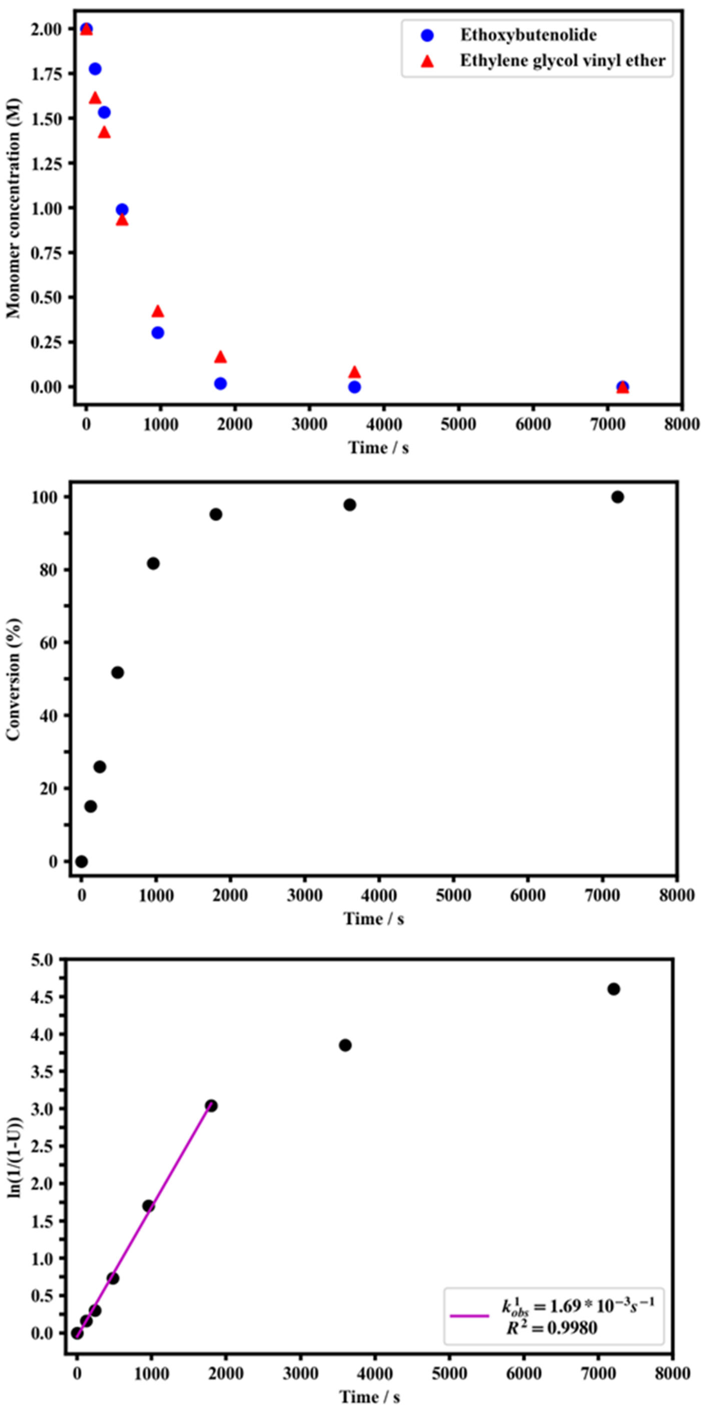

Figure S48. Kinetics of the copolymerization using ethoxy butenolide and EGVE followed by ^{1}H-NMR spectroscopy at regular intervals throughout the reaction. Top: concentration of monomers over time. Middle: conversion of monomers over time. Bottom: initial rate of copolymerization.

Poly(hexyloxy butenolide-co-dodecyl vinyl ether) (2-DVE)

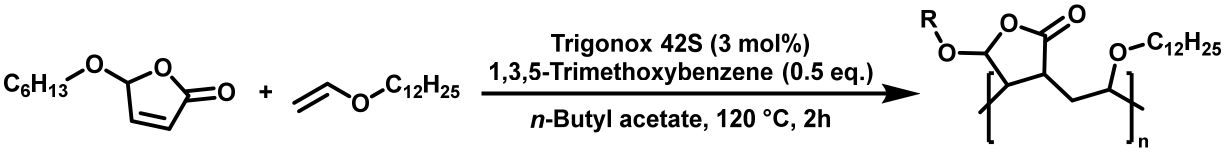

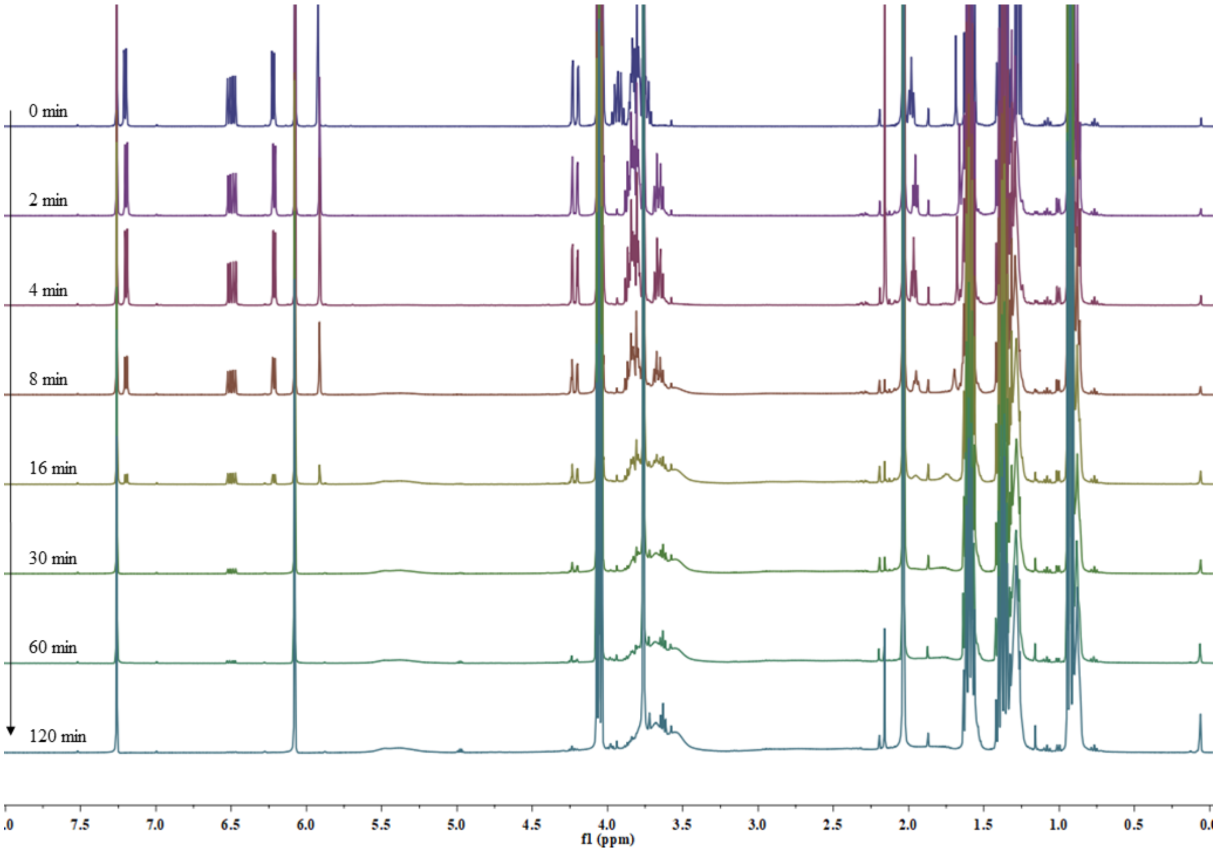

Figure S49. ^{1}H-NMR spectra of the co-polymerization of hexyloxy butenolide with DVE over time.

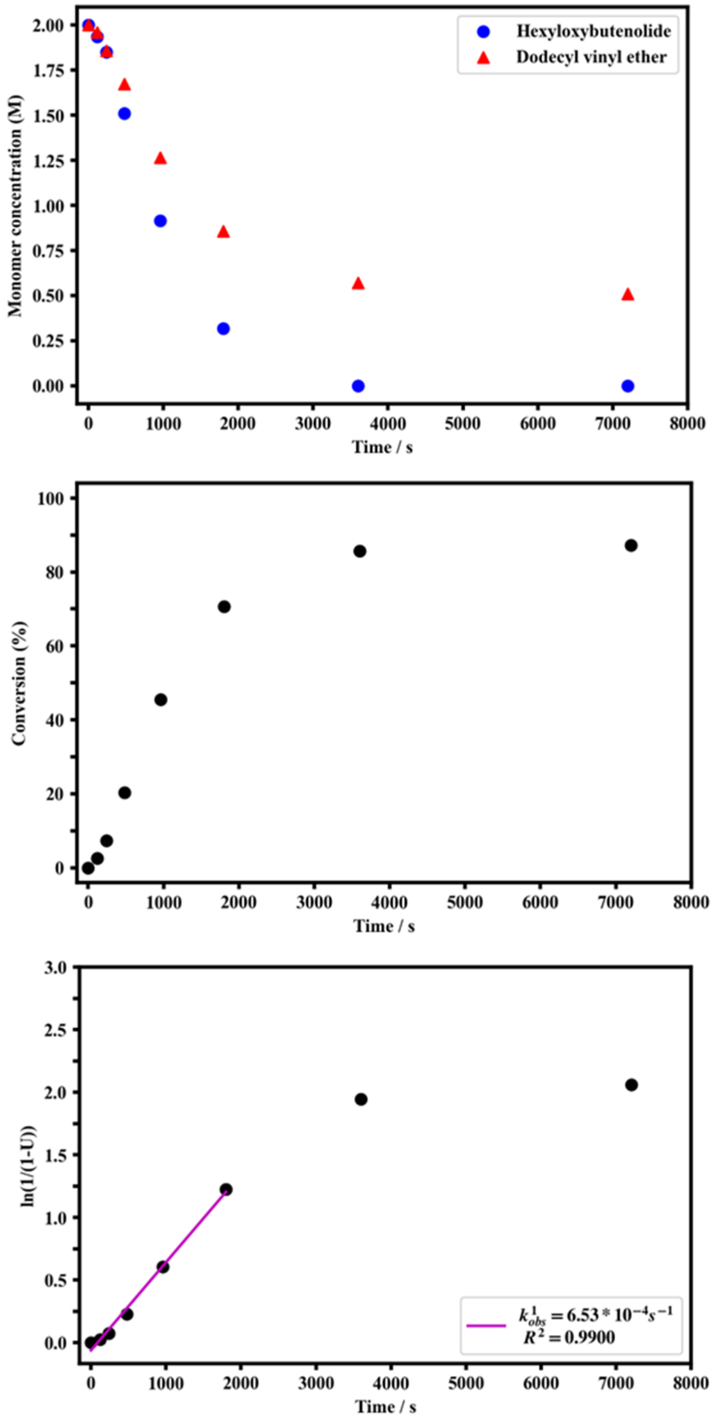

Figure S50. Kinetics of the copolymerization using hexyloxy butenolide and DVE followed by ^{1}H-NMR spectroscopy at regular intervals throughout the reaction. Top: concentration of monomers over time. Middle: conversion of monomers over time. Bottom: initial rate of copolymerization.

Poly(hexyloxy butenolide-co-ethylene glycol vinyl ether) (2-EGVE)

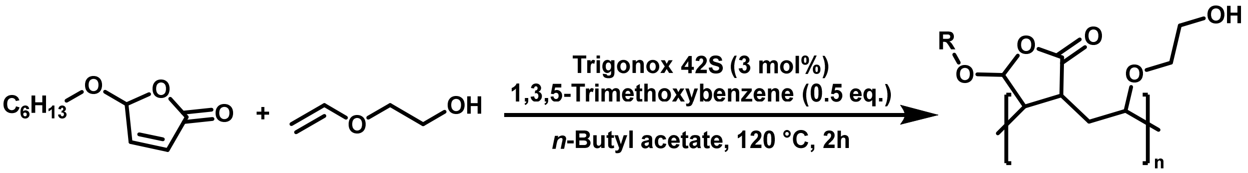

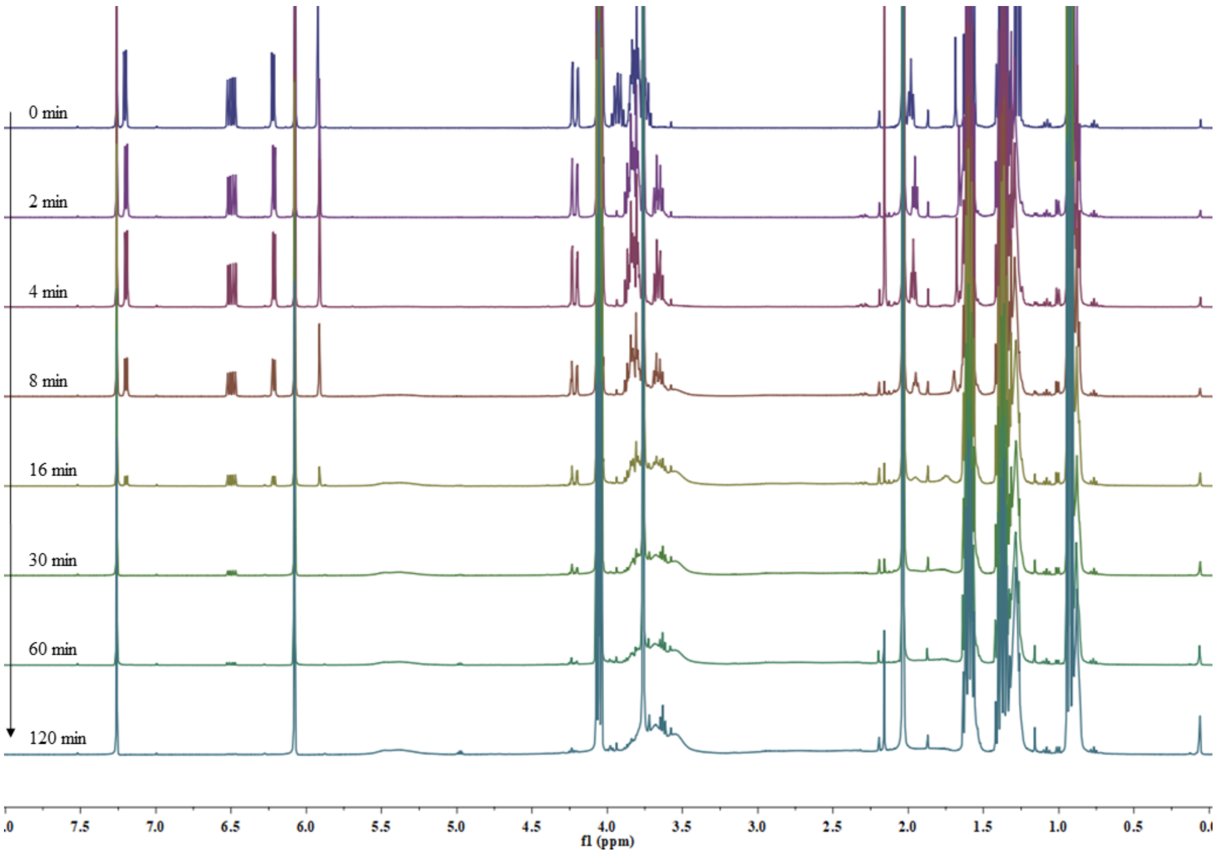

Figure S51. ^{1}H-NMR spectra of the co-polymerization of hexyloxy butenolide with EGVE over time.

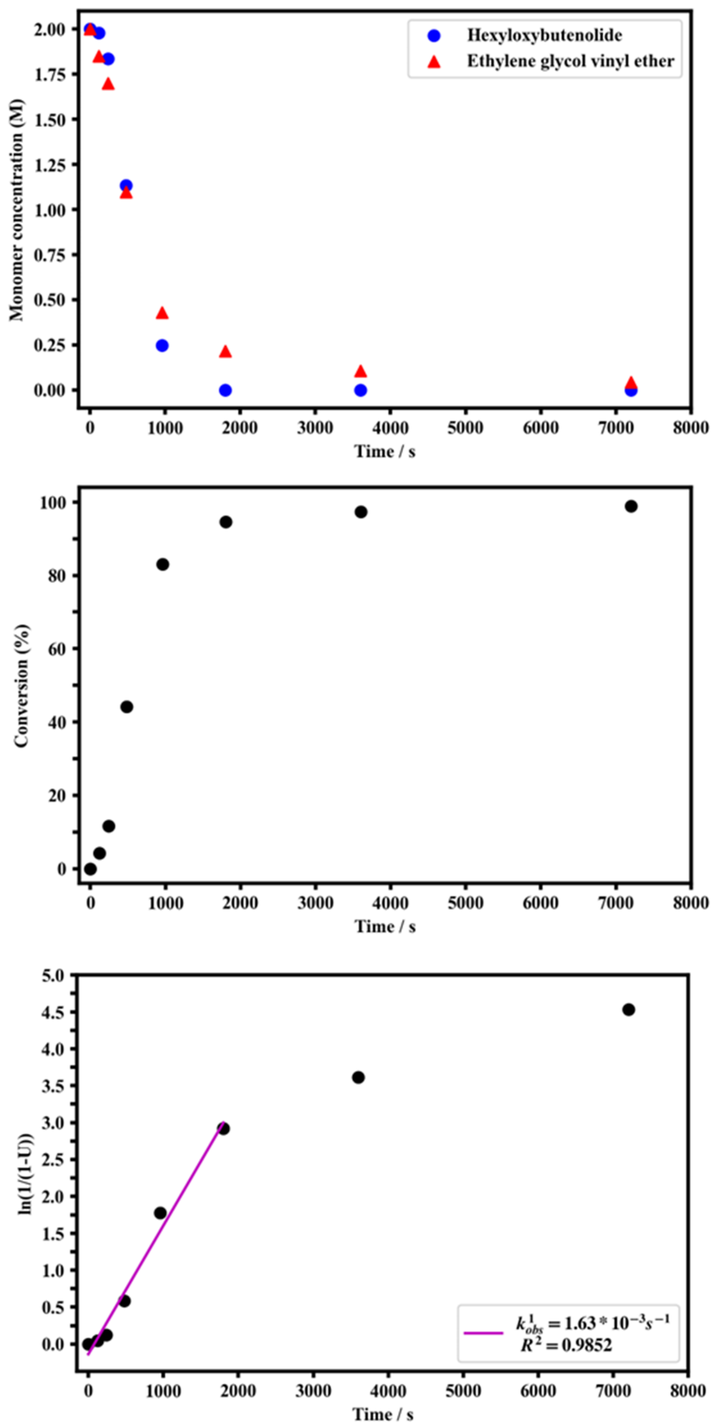

Figure S52. Kinetics of the copolymerization using hexyloxy butenolide and EGVE followed by ^{1}H-NMR spectroscopy at regular intervals throughout the reaction. Top: concentration of monomers over time. Middle: conversion of monomers over time. Bottom: initial rate of copolymerization.

Poly(dodecyloxy butenolide-co-dodecyl vinyl ether) (3-DVE)

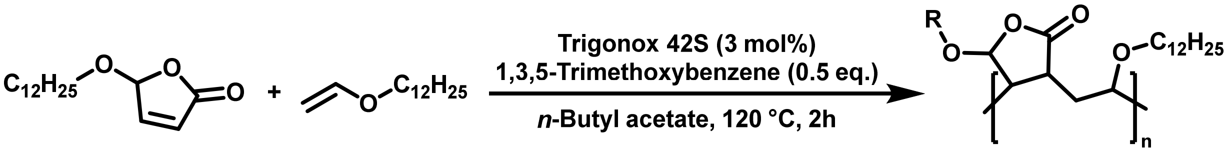

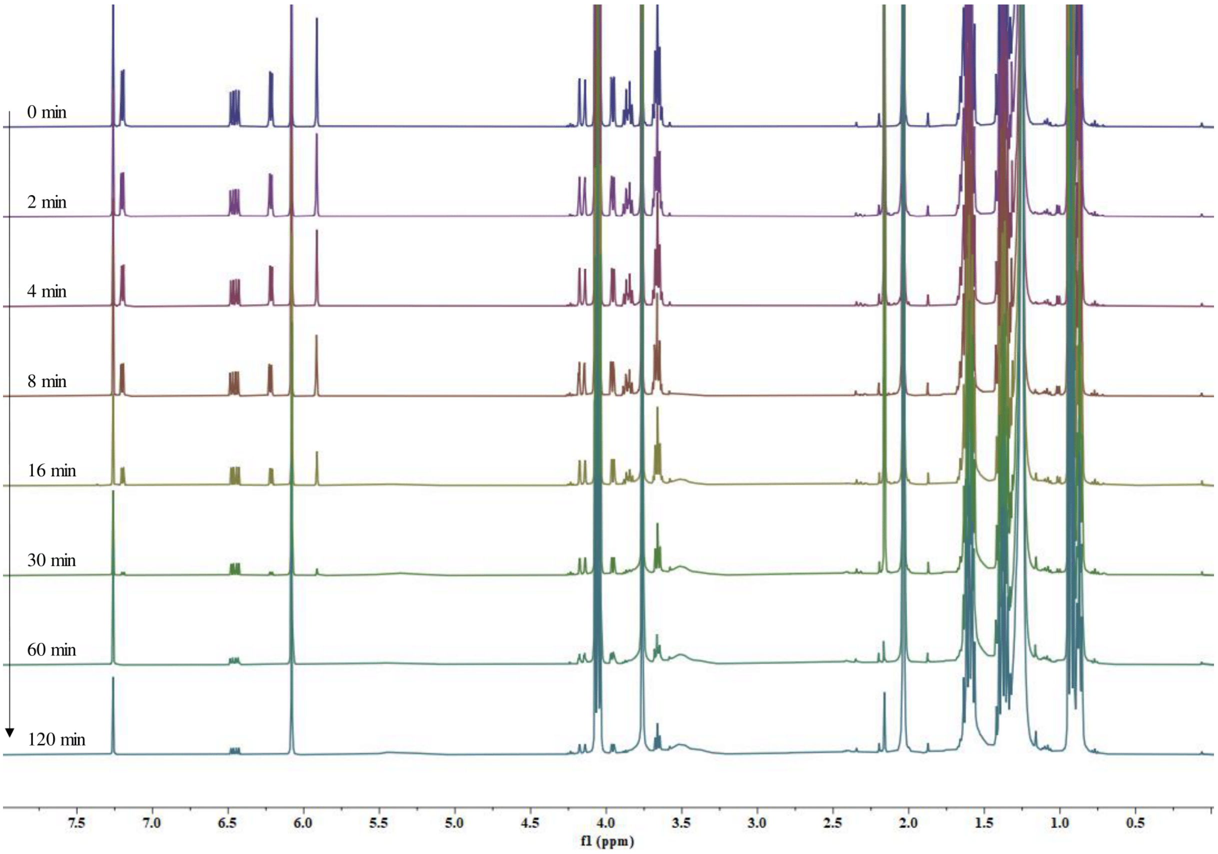

Figure S53. ^{1}H-NMR spectra of the co-polymerization of dodecyloxy butenolide with DVE over time.

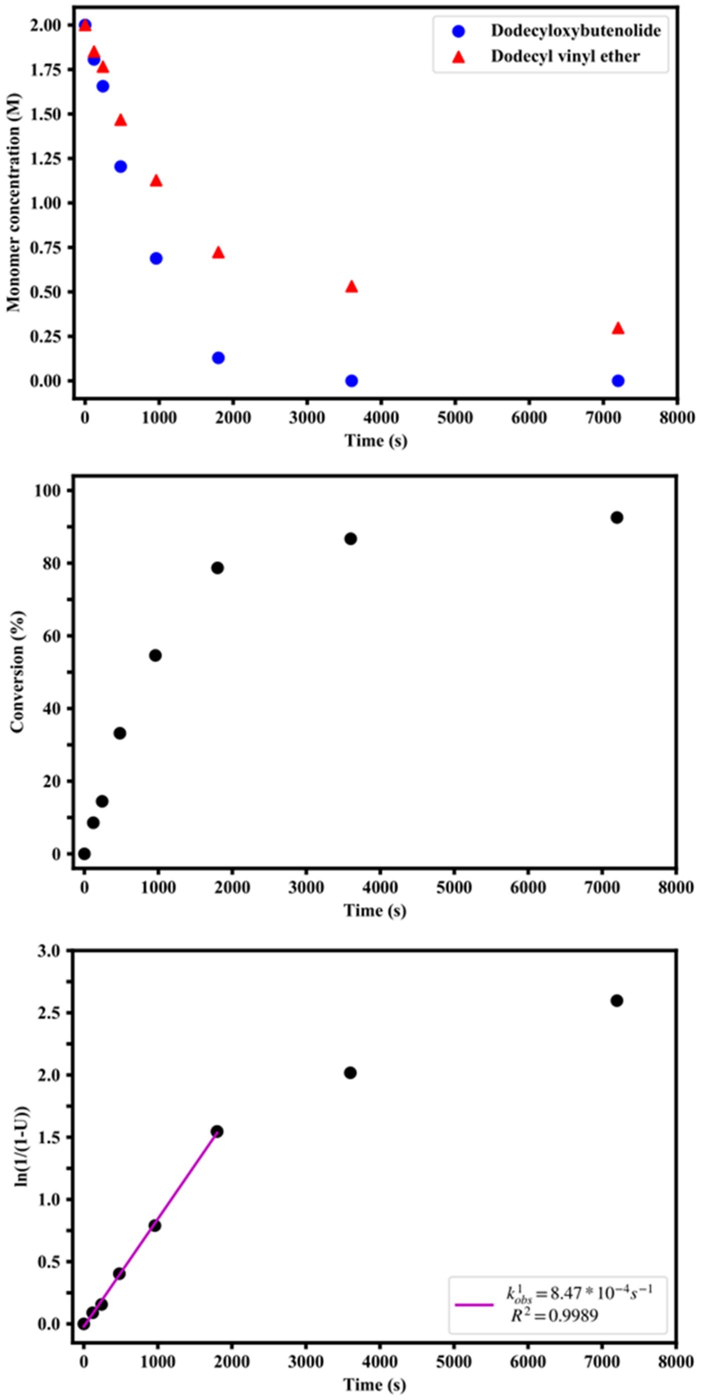

Figure S54. Kinetics of the copolymerization using dodecyloxy butenolide and DVE followed by ^{1}H-NMR spectroscopy at regular intervals throughout the reaction. Top: concentration of monomers over time. Middle: conversion of monomers over time. Bottom: initial rate of copolymerization.

Poly(dodecyloxy butenolide-co-ethylene glycol vinyl ether) (3-EGVE)

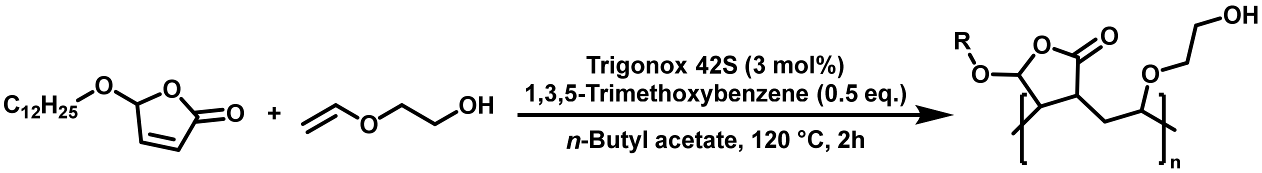

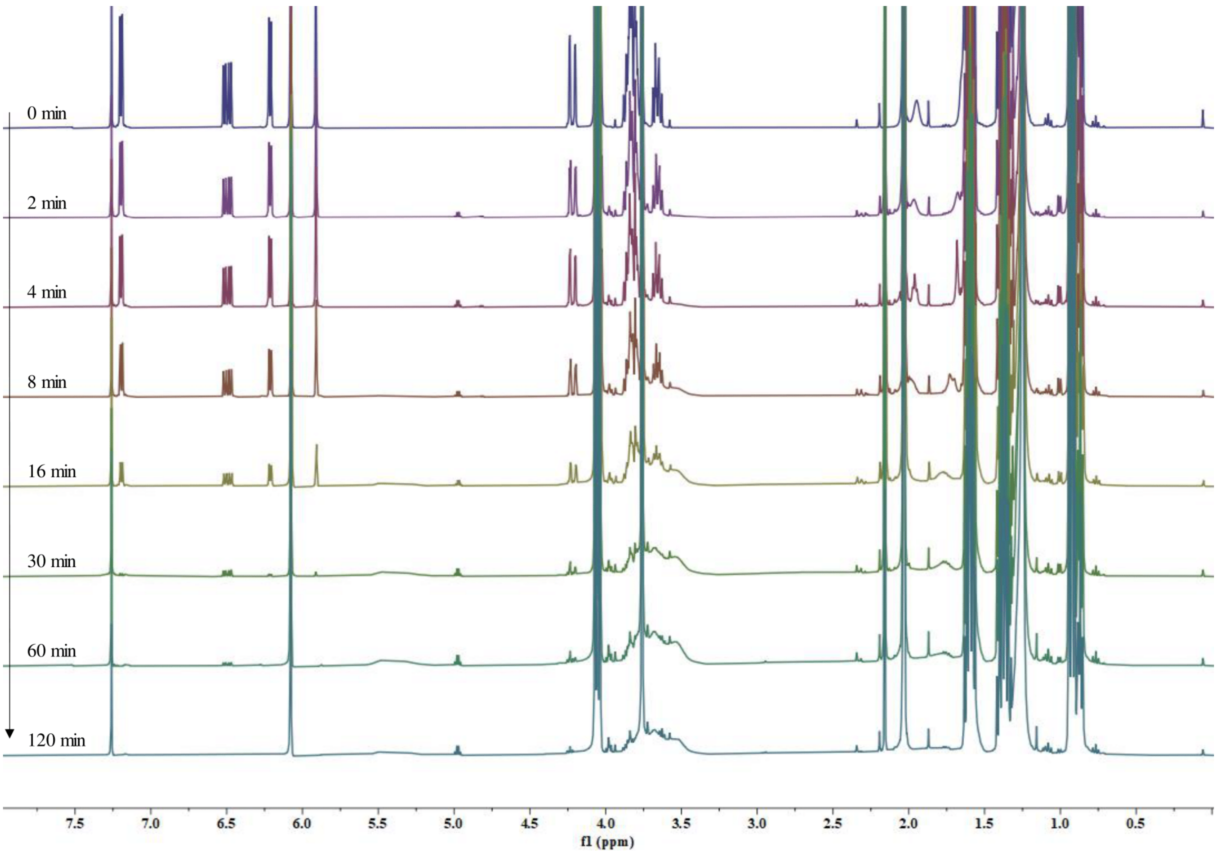

Figure S55. ^{1}H-NMR spectra of the co-polymerization of dodecyloxy butenolide with EGVE over time.

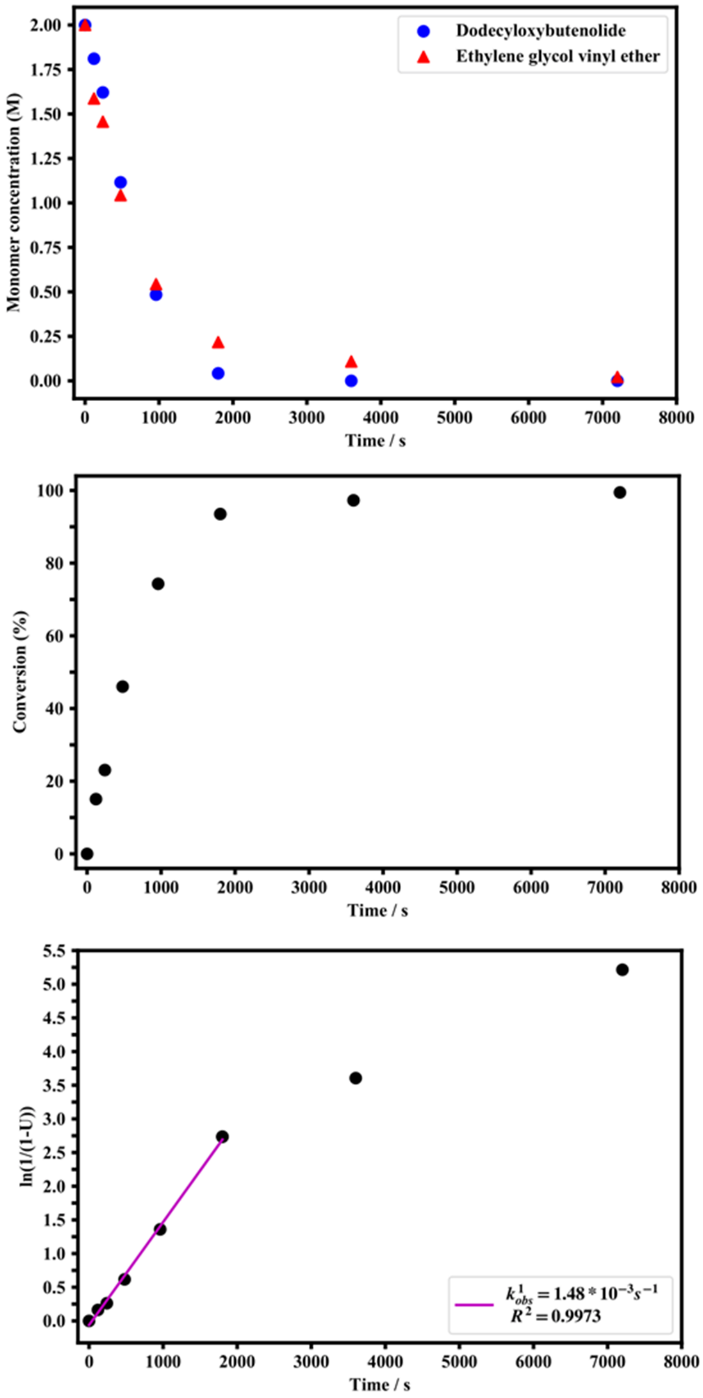

Figure S56. Kinetics of the copolymerization using dodecyloxy butenolide and EGVE followed by ^{1}H-NMR spectroscopy at regular intervals throughout the reaction. Top: concentration of monomers over time. Middle: conversion of monomers over time. Bottom: initial rate of copolymerization.

Poly(citronelloxy butenolide-co-dodecyl vinyl ether) (4-DVE)

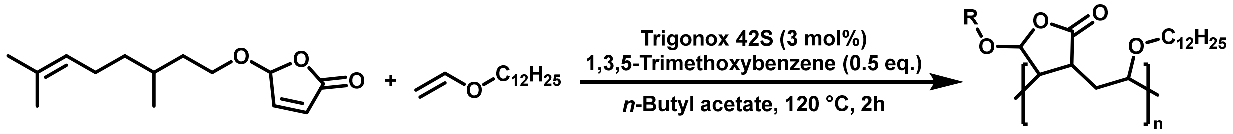

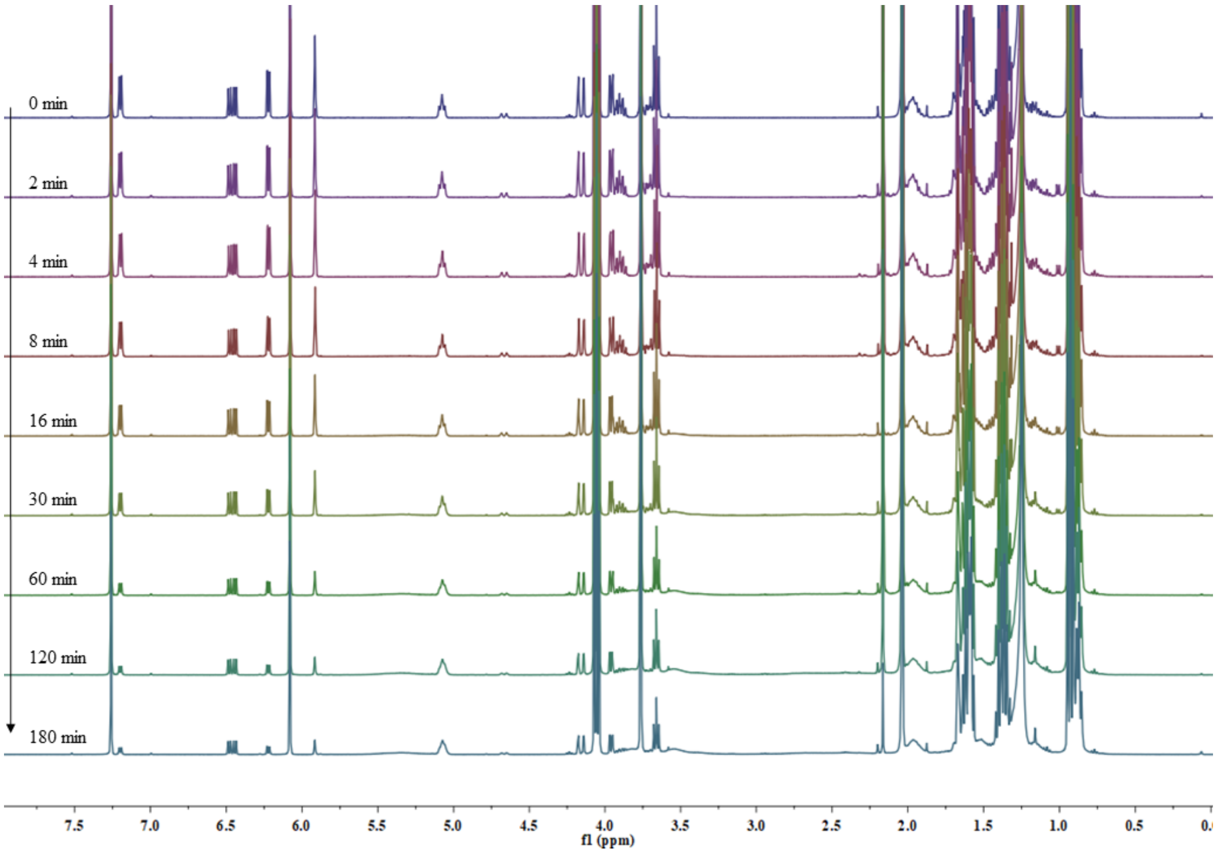

Figure S57. ^{1}H-NMR spectra of the co-polymerization of citronelloxy butenolide with DVE over time.

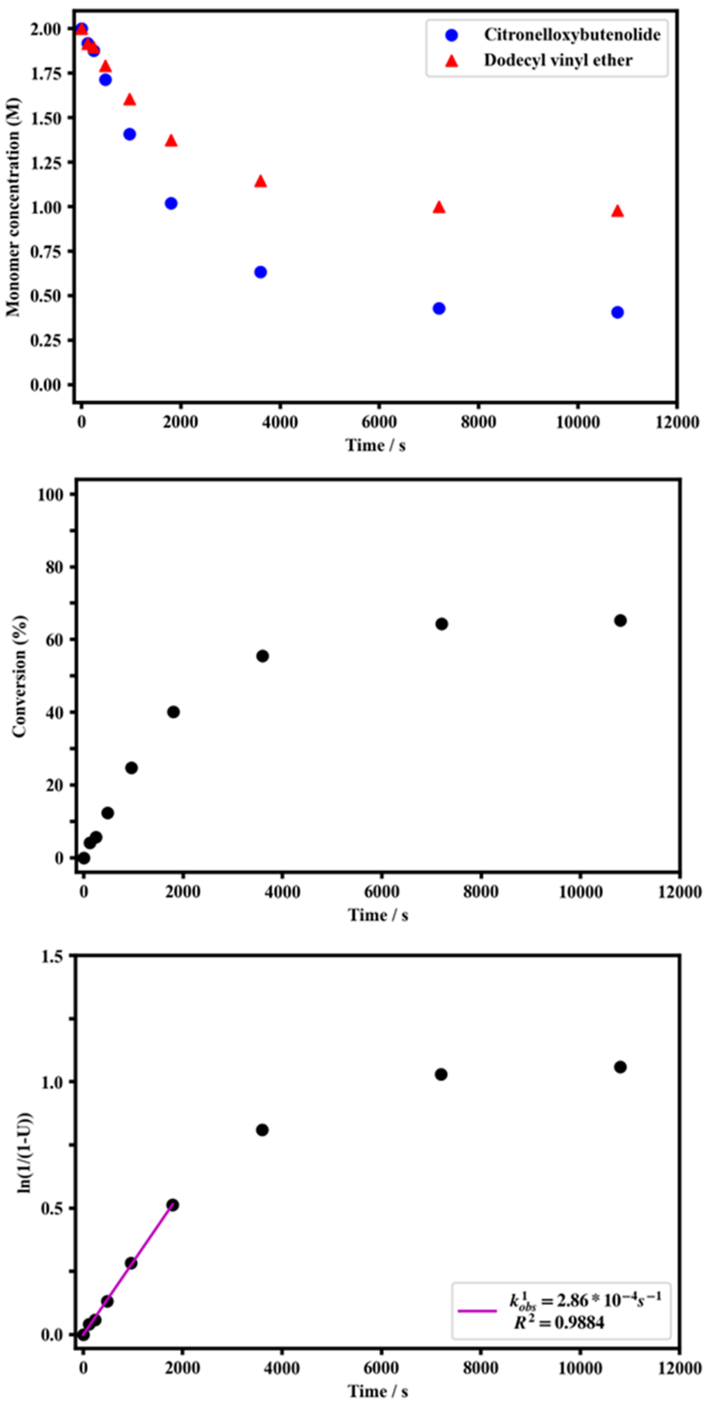

Figure S58. Kinetics of the copolymerization using citronelloxy butenolide and DVE followed by ^{1}H-NMR spectroscopy at regular intervals throughout the reaction. Top: concentration of monomers over time. Middle: conversion of monomers over time. Bottom: initial rate of copolymerization.

Poly(citronelloxy butenolide-co-ethylene glycol vinyl ether) (4-EGVE)

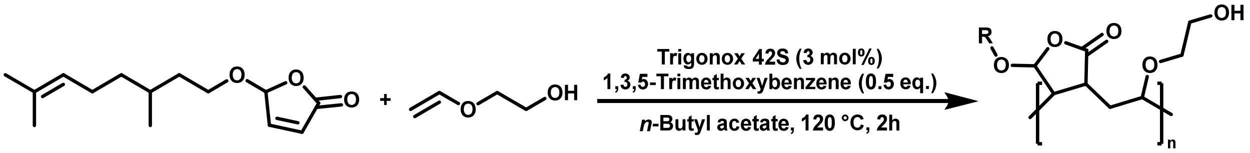

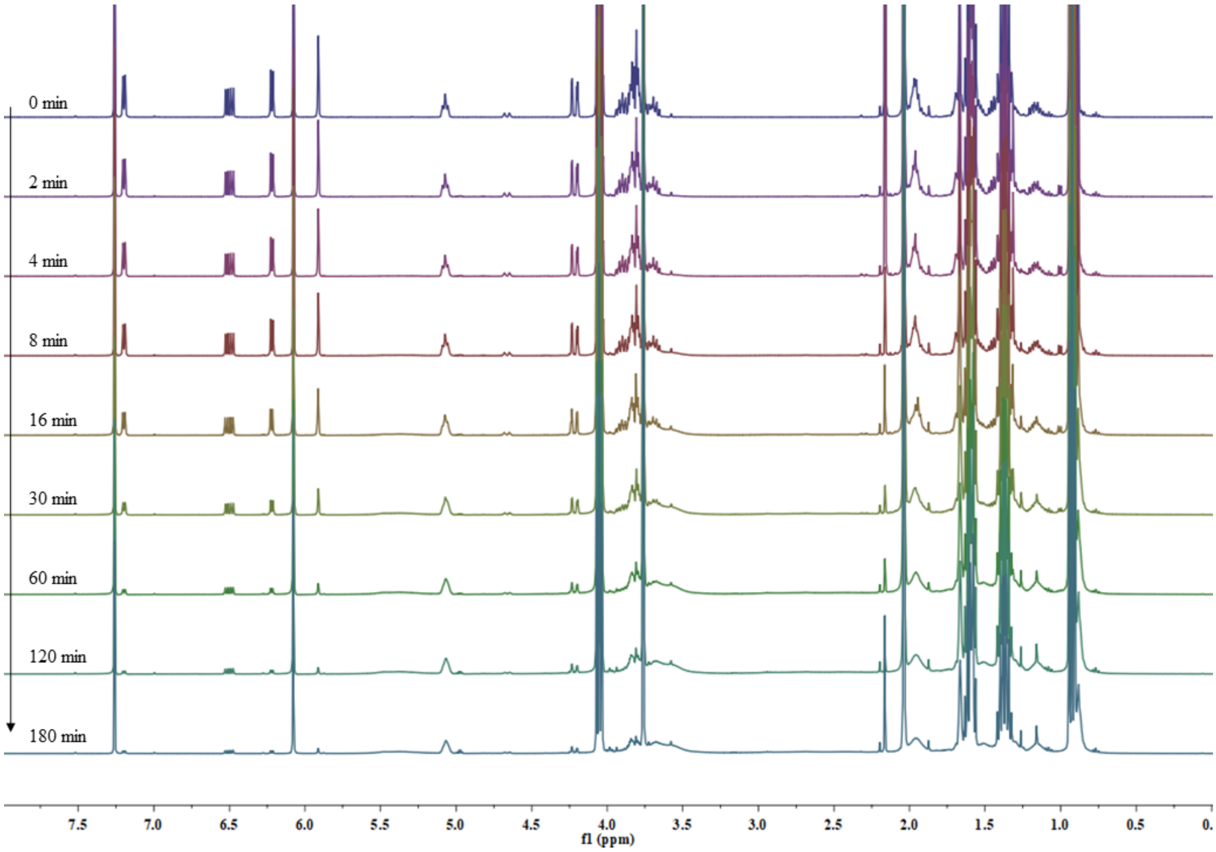

Figure S59. ^{1}H-NMR spectra of the co-polymerization of citronelloxy butenolide with EGVE over time.

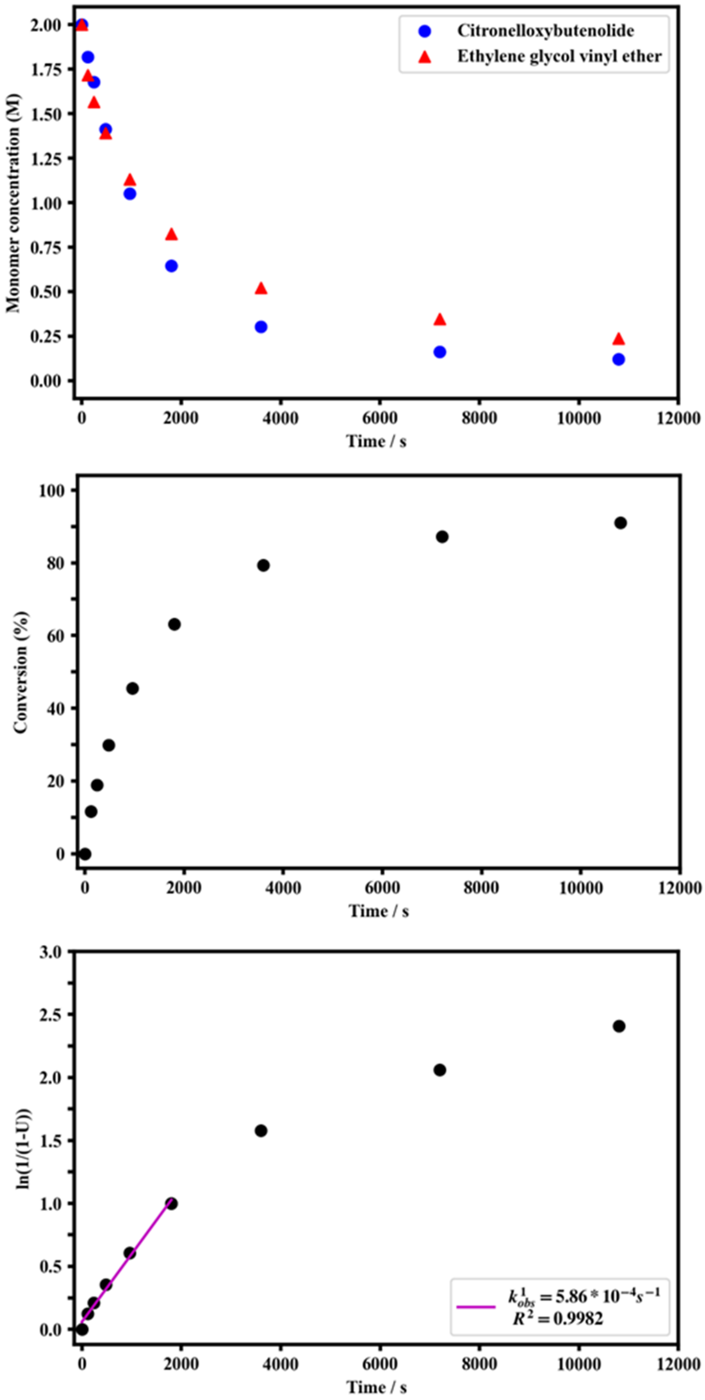

Figure S60. Kinetics of the copolymerization using citronelloxy butenolide and EGVE followed by ^{1}H-NMR spectroscopy at regular intervals throughout the reaction. Top: concentration of monomers over time. Middle: conversion of monomers over time. Bottom: initial rate of copolymerization.

Poly(dihydrocitronelloxy butenolide-co-dodecyl vinyl ether) (5-DVE)

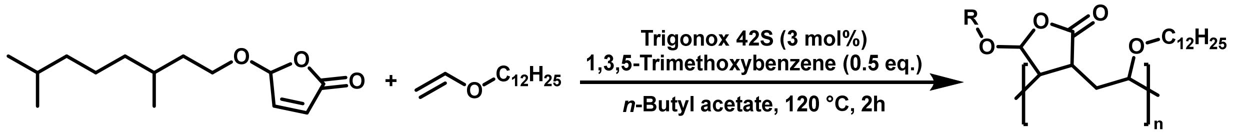

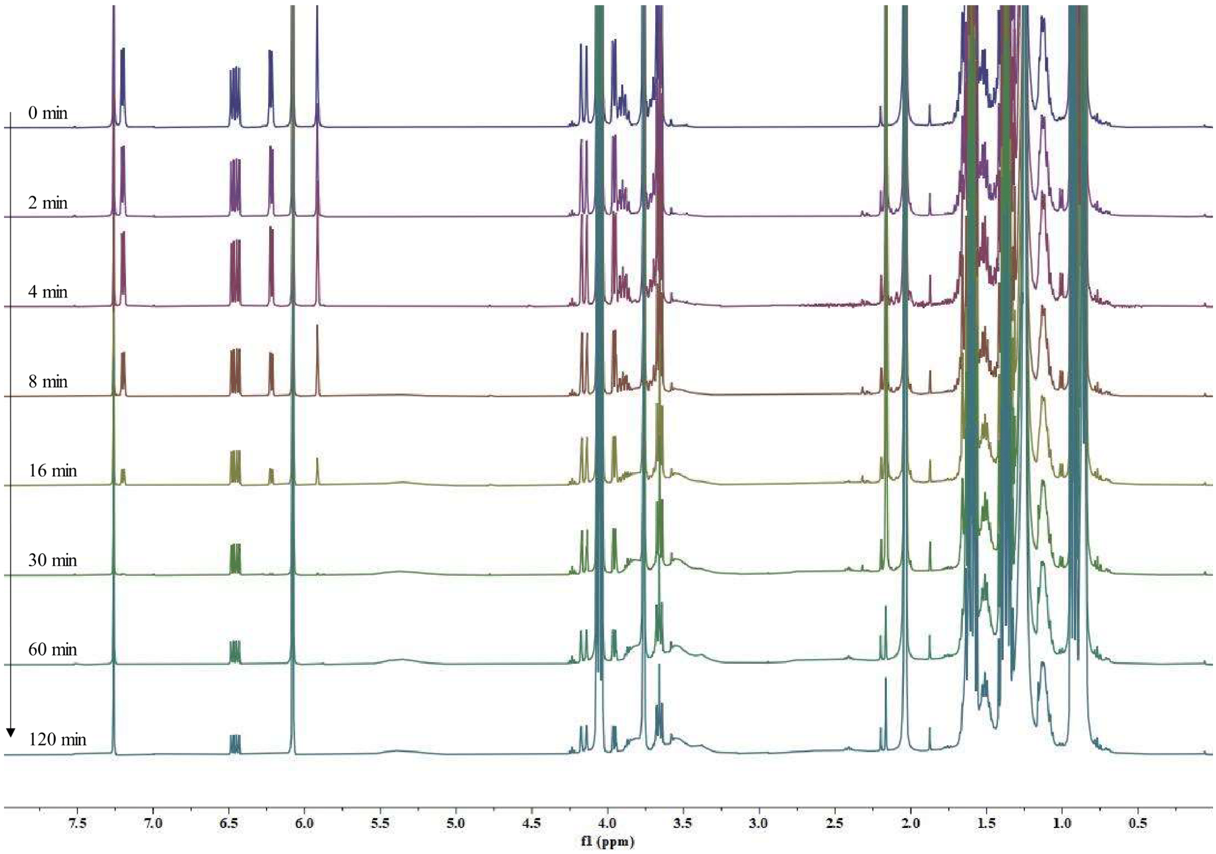

Figure S61. ^{1}H-NMR spectra of the co-polymerization of dihydrocitronelloxy butenolide with DVE over time.

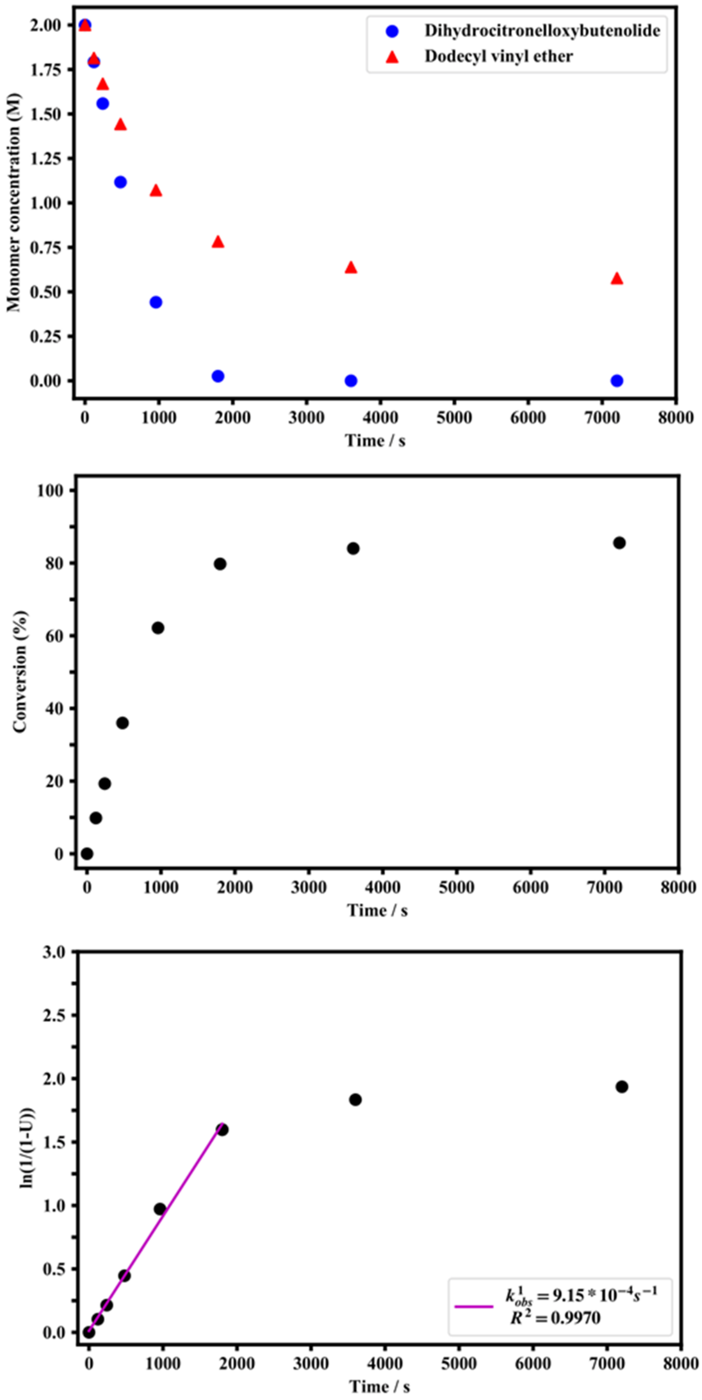

Figure S62. Kinetics of the copolymerization using dihydrocitronelloxy butenolide and DVE followed by ^{1}H-NMR spectroscopy at regular intervals throughout the reaction. Top: concentration of monomers over time. Middle: conversion of monomers over time. Bottom: initial rate of copolymerization.

Poly(dihydrocitronelloxy butenolide-co-ethylene glycol vinyl ether) (5-EGVE)

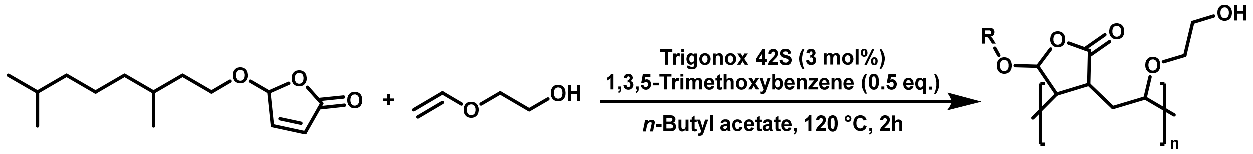

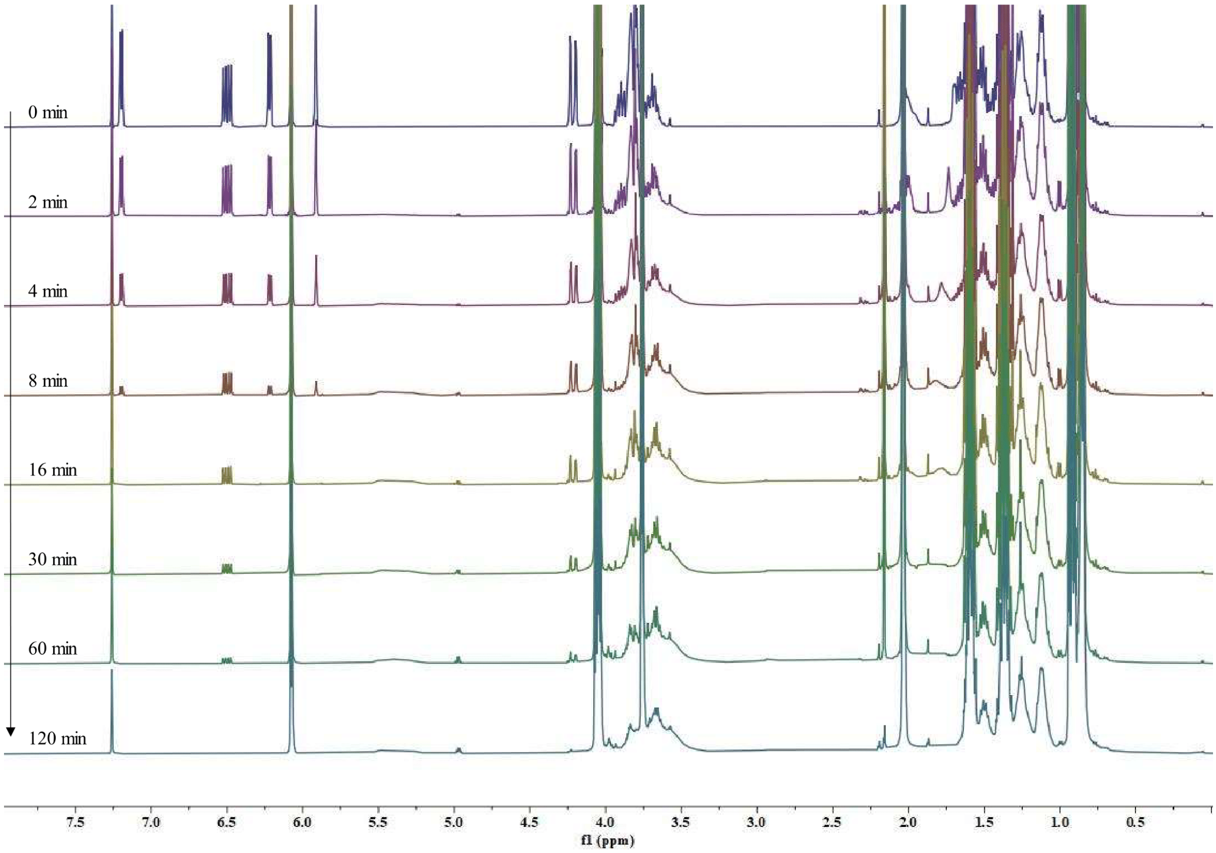

Figure S63. ^{1}H-NMR spectra of the co-polymerization of dihydrocitronelloxy butenolide with EGVE over time.

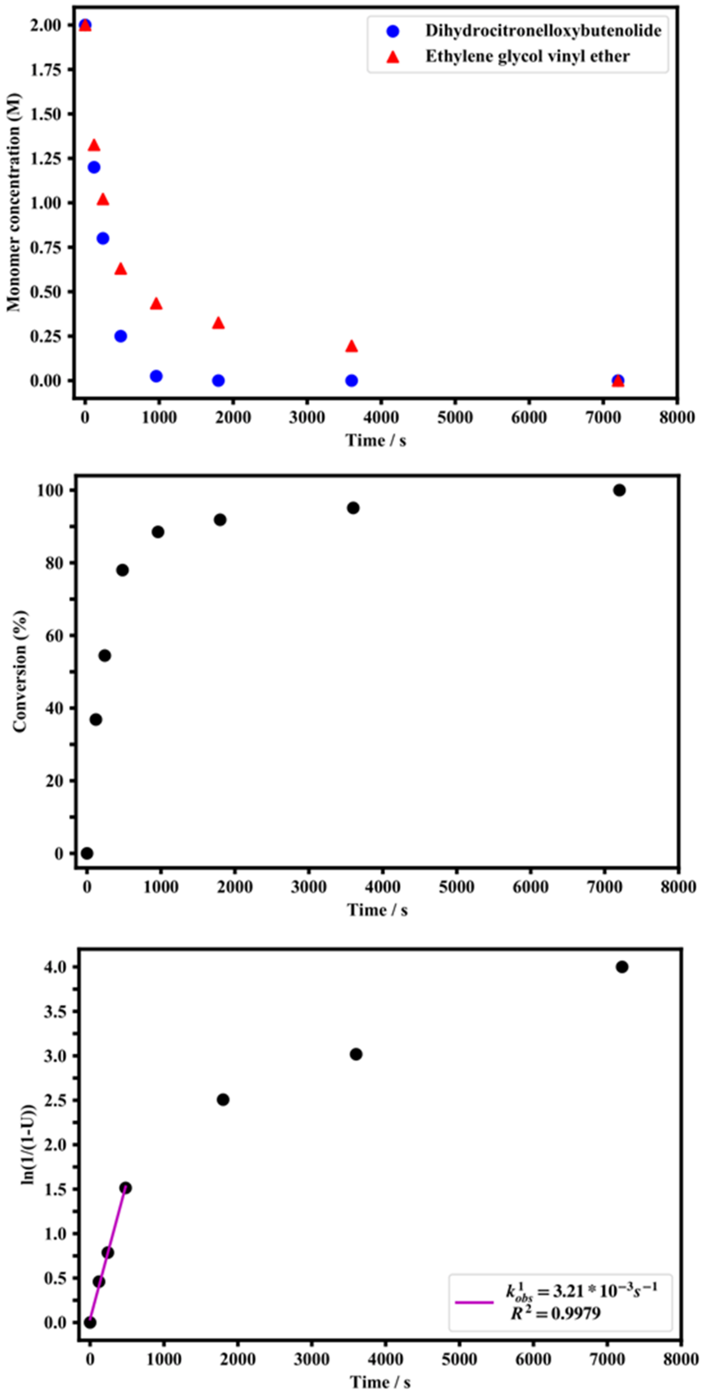

Figure S64. Kinetics of the copolymerization using dihydrocitronelloxy butenolide and EGVE followed by ^{1}H-NMR spectroscopy at regular intervals throughout the reaction. Top: concentration of monomers over time. Middle: conversion of monomers over time. Bottom: initial rate of copolymerization.

Poly(phenylethoxy butenolide-co-dodecyl vinyl ether) (6-DVE)

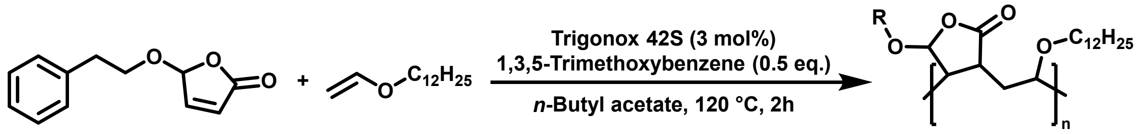

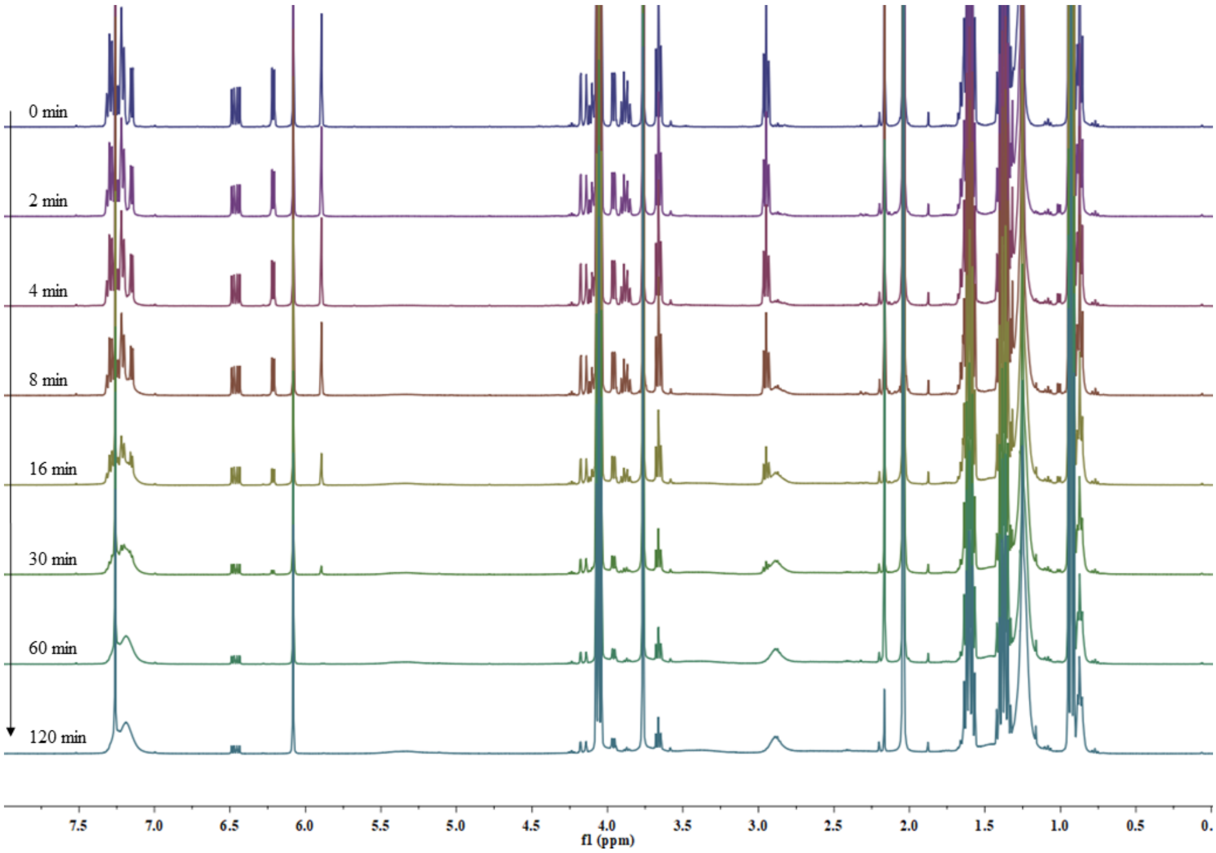

Figure S65. ^{1}H-NMR spectra of the co-polymerization of phenylethoxy butenolide with DVE over time.

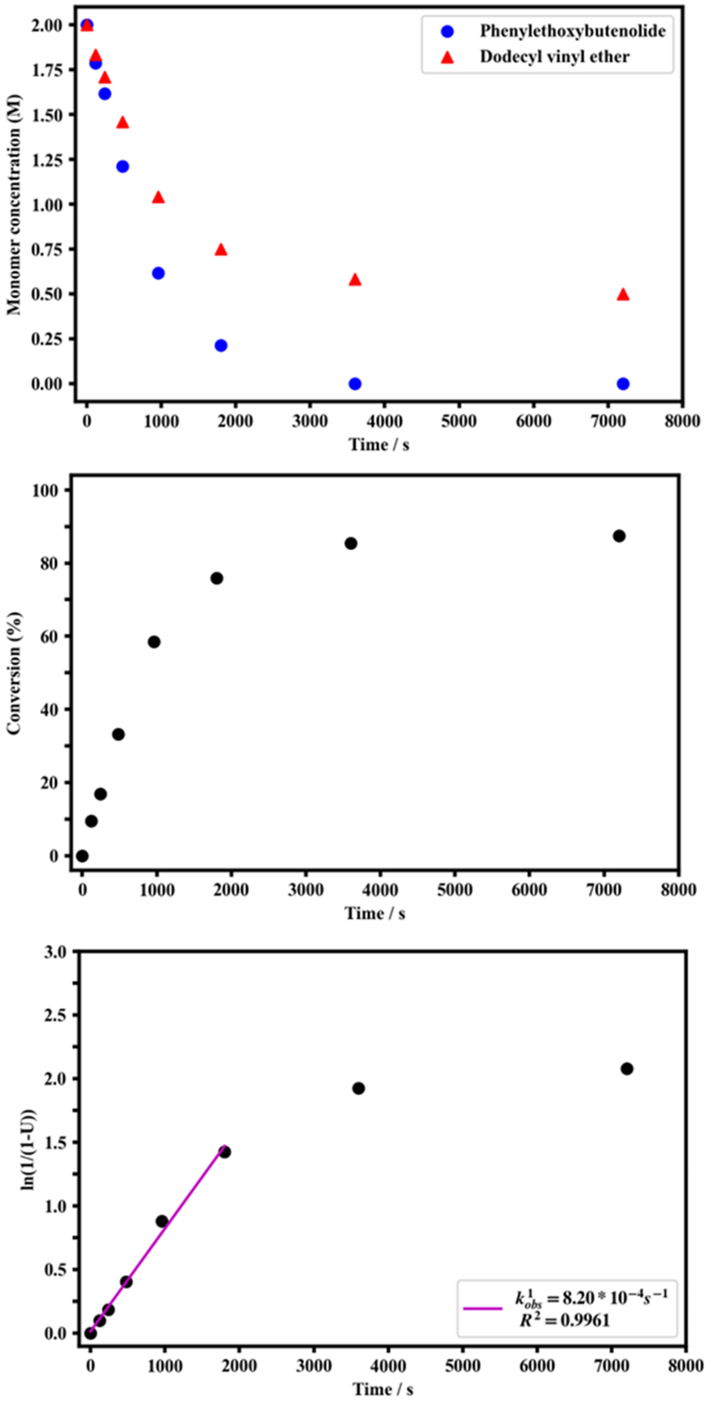

Figure S66. Kinetics of the copolymerization using phenylethoxy butenolide and DVE followed by ^{1}H-NMR spectroscopy at regular intervals throughout the reaction. Top: concentration of monomers over time. Middle: conversion of monomers over time. Bottom: initial rate of copolymerization.

Poly(phenylethoxy butenolide-co-ethylene glycol vinyl ether) (6-EGVE)

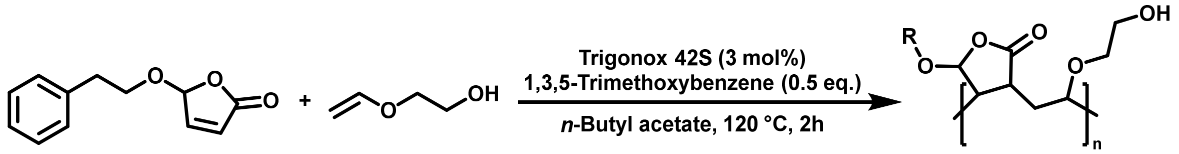

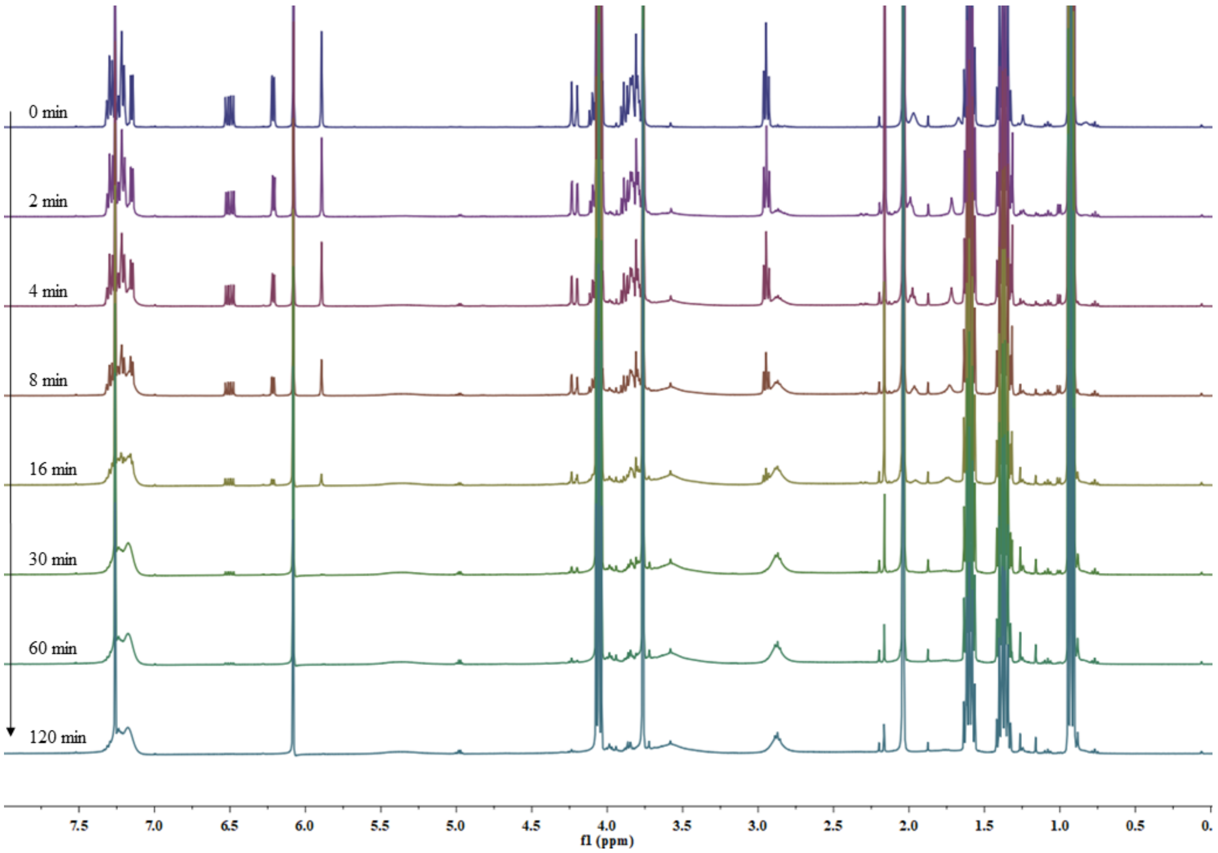

Figure S67. ^{1}H-NMR spectra of the co-polymerization of phenylethoxy butenolide with EGVE over time.

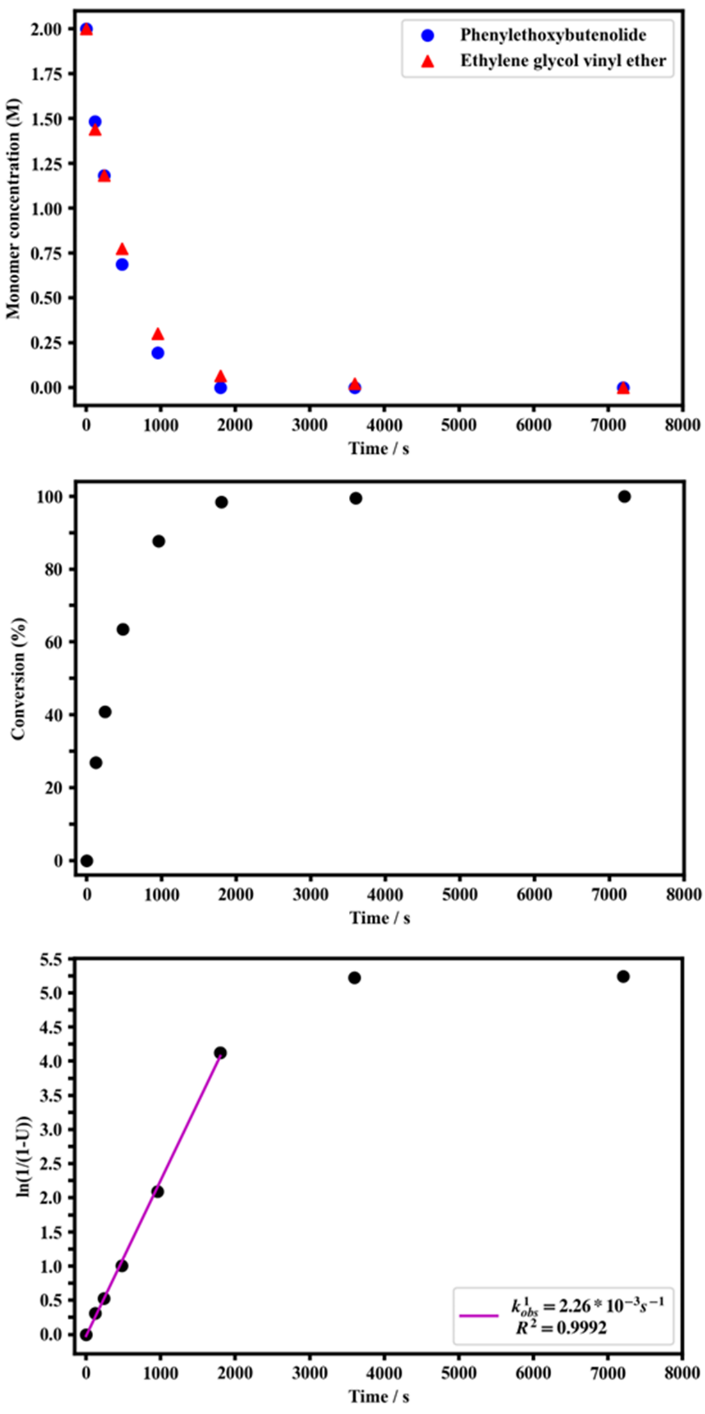

Figure S68. Kinetics of the copolymerization using phenylethoxy butenolide and EGVE followed by ^{1}H-NMR spectroscopy at regular intervals throughout the reaction. Top: concentration of monomers over time. Middle: conversion of monomers over time. Bottom: initial rate of copolymerization.

Poly(menthyloxy butenolide-co-dodecyl vinyl ether) (7-DVE)

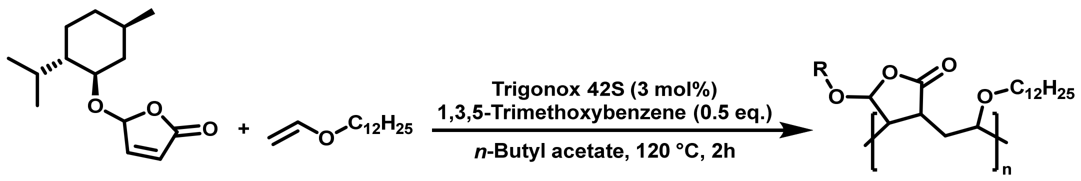

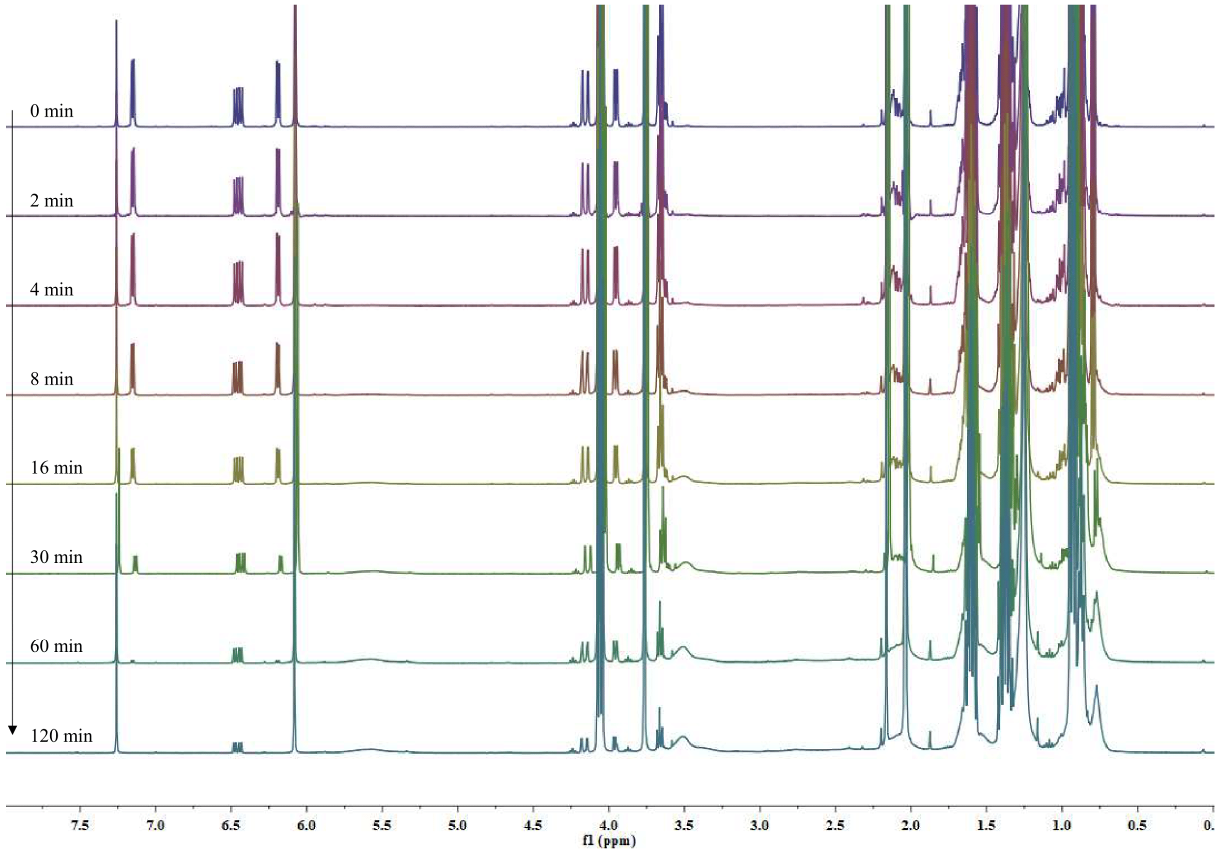

Figure S69. ^{1}H-NMR spectra of the co-polymerization of menthyloxy butenolide with DVE over time.

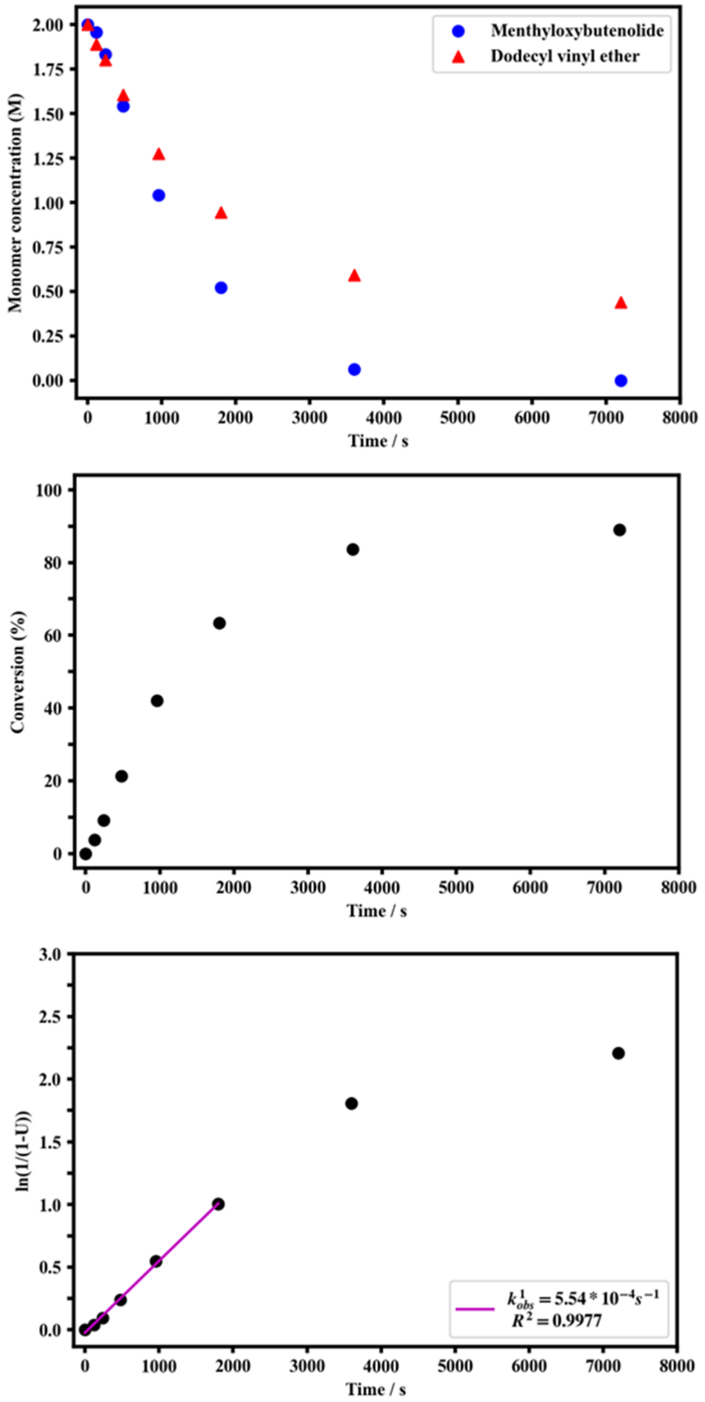

Figure S70. Kinetics of the copolymerization using menthyloxy butenolide and DVE followed by ^{1}H-NMR spectroscopy at regular intervals throughout the reaction. Top: concentration of monomers over time. Middle: conversion of monomers over time. Bottom: initial rate of copolymerization.

Poly(menthyloxy butenolide-co-ethylene glycol vinyl ether) (7-EGVE)

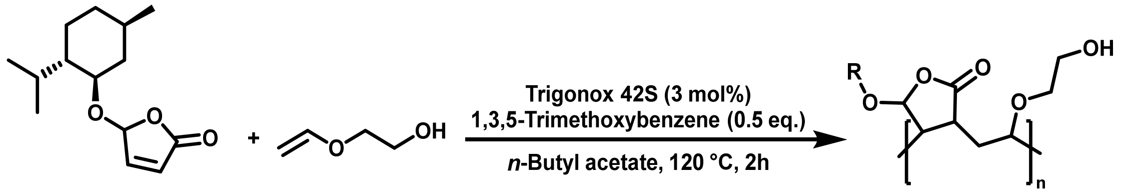

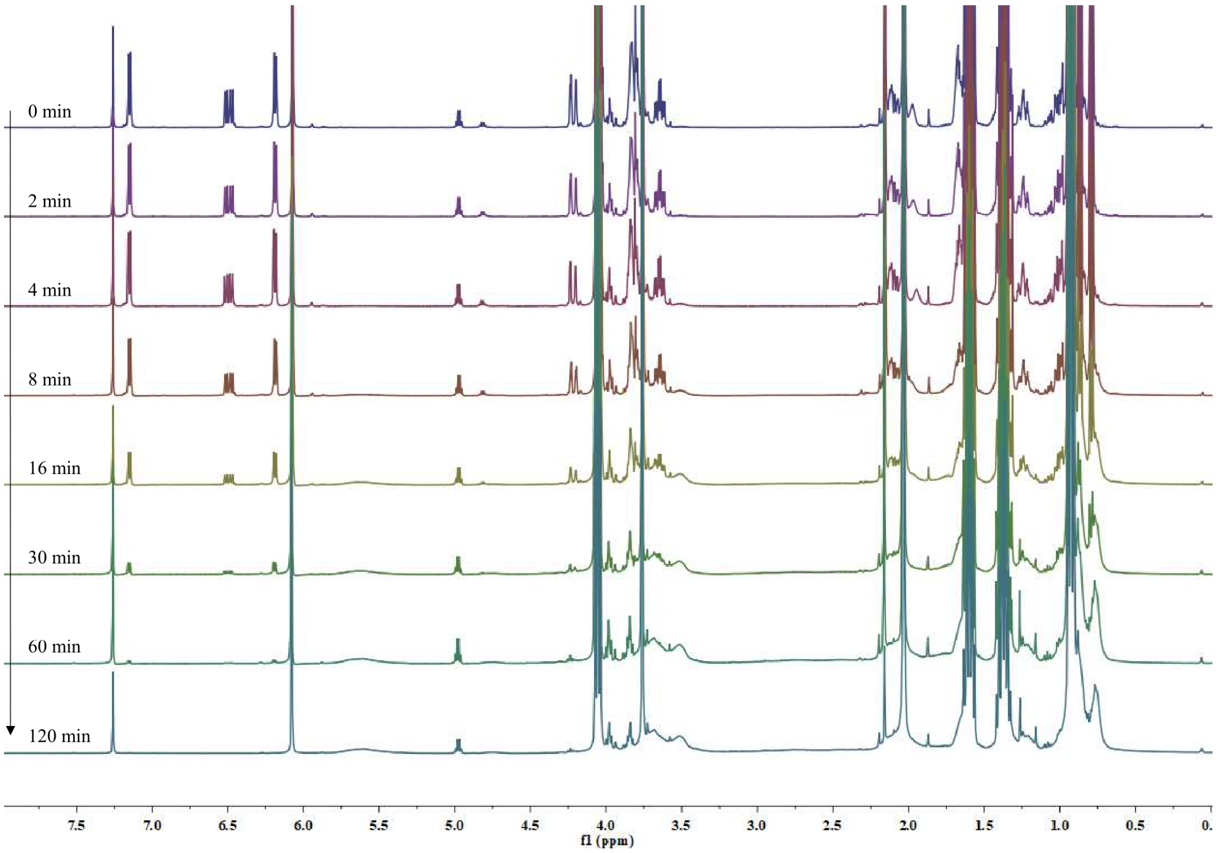

Figure S71. ^{1}H-NMR spectra of the co-polymerization of menthyloxy butenolide with EGVE over time.

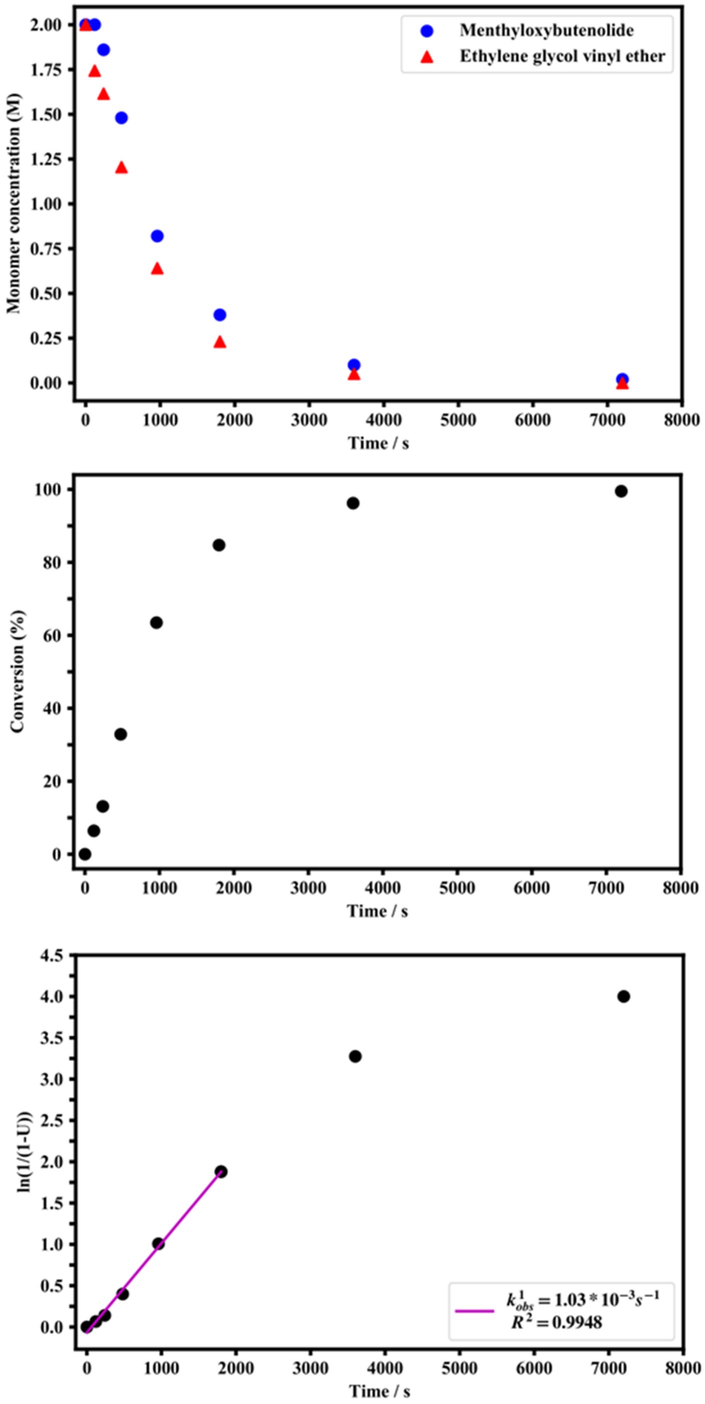

Figure S72. Kinetics of the copolymerization using menthyloxy butenolide and EGVE followed by ^{1}H-NMR spectroscopy at regular intervals throughout the reaction. Top: concentration of monomers over time. Middle: conversion of monomers over time. Bottom: initial rate of copolymerization.

Gel permeation chromatography (GPC)

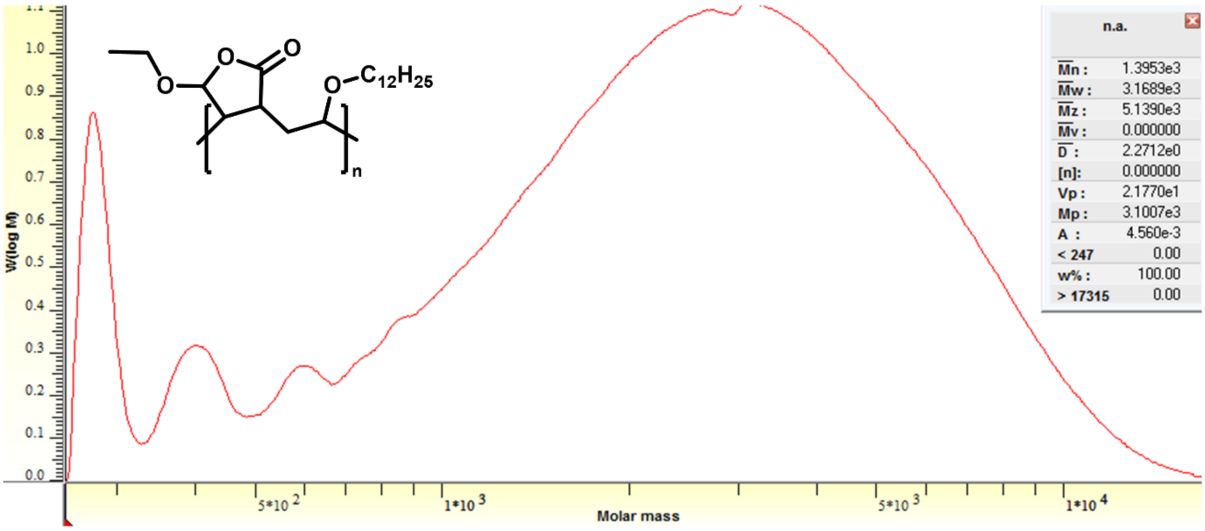

Figure S73. GPC spectrum (using THF as stationary phase) of poly(ethoxy butenolide-co-dodecyl vinyl ether) (**1-DVE**).

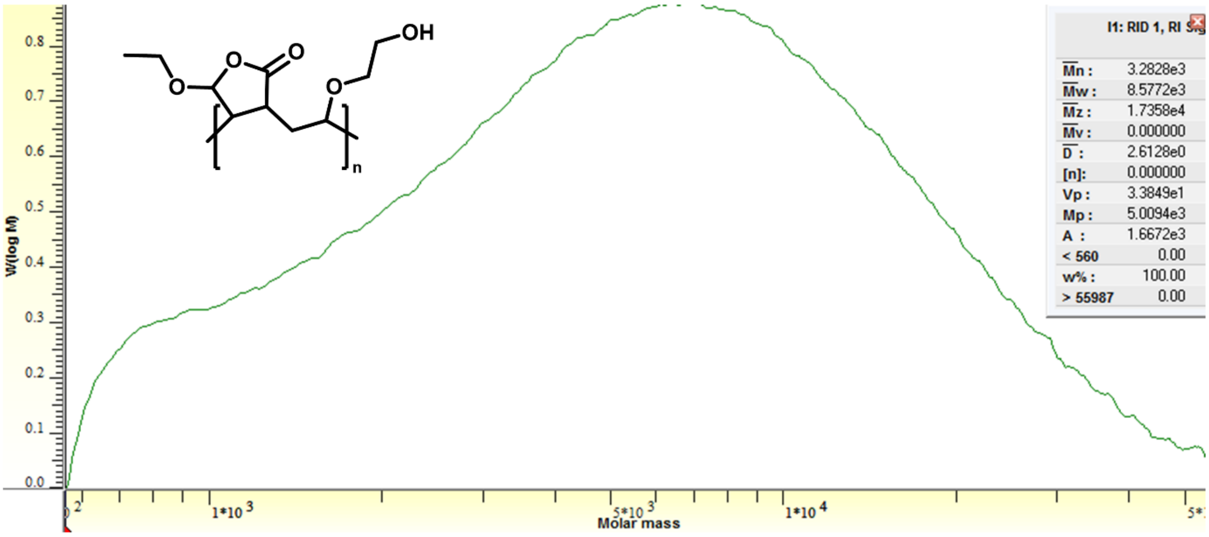

Figure S74. GPC spectrum (using DMF as stationary phase) of poly(ethoxy butenolide-co-ethylene glycol vinyl ether) (**1-EGVE**).

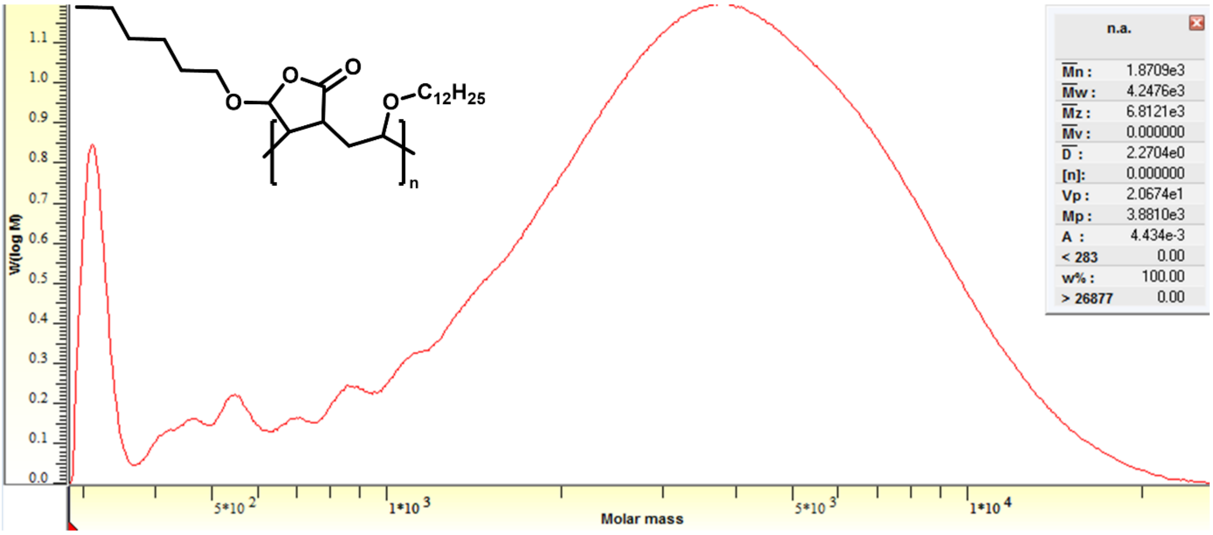

Figure S75. GPC spectrum (using THF as stationary phase) of poly(hexyloxy butenolide-co-dodecyl vinyl ether) (**2-DVE**).

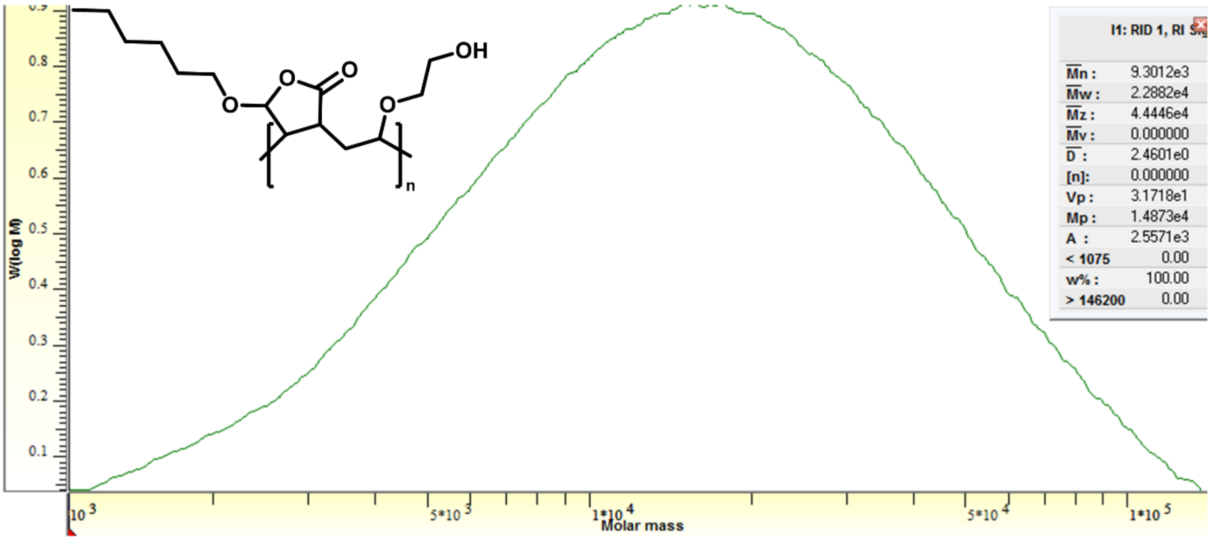

Figure S76. GPC spectrum (using DMF as stationary phase) of poly(hexyloxy butenolide-co-ethylene glycol vinyl ether) (**2-EGVE**).

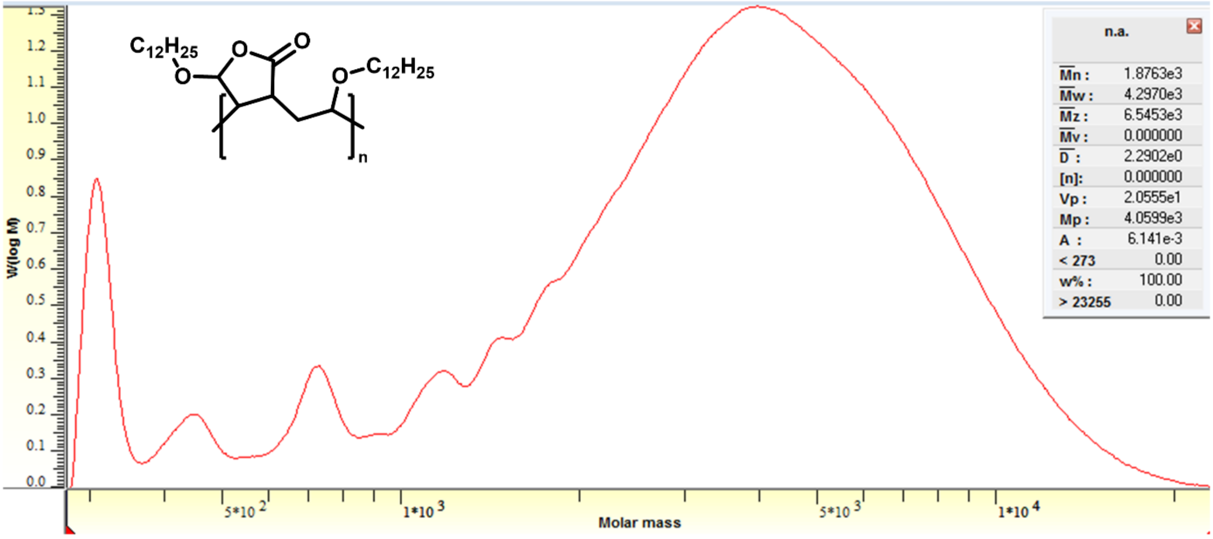

Figure S77. GPC spectrum (using THF as stationary phase) of poly(dodecyloxy butenolide-co-dodecyl vinyl ether) (**3-DVE**).

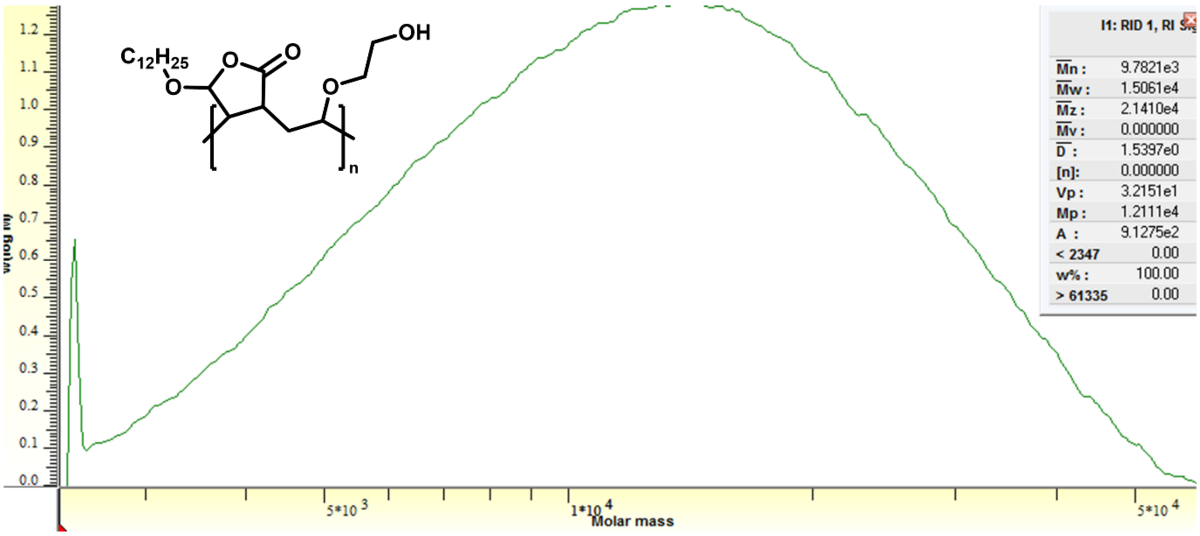

Figure S78. GPC spectrum (using DMF as stationary phase) of poly(dodecyloxy butenolide-co-ethylene glycol vinyl ether) (**3-EGVE**).

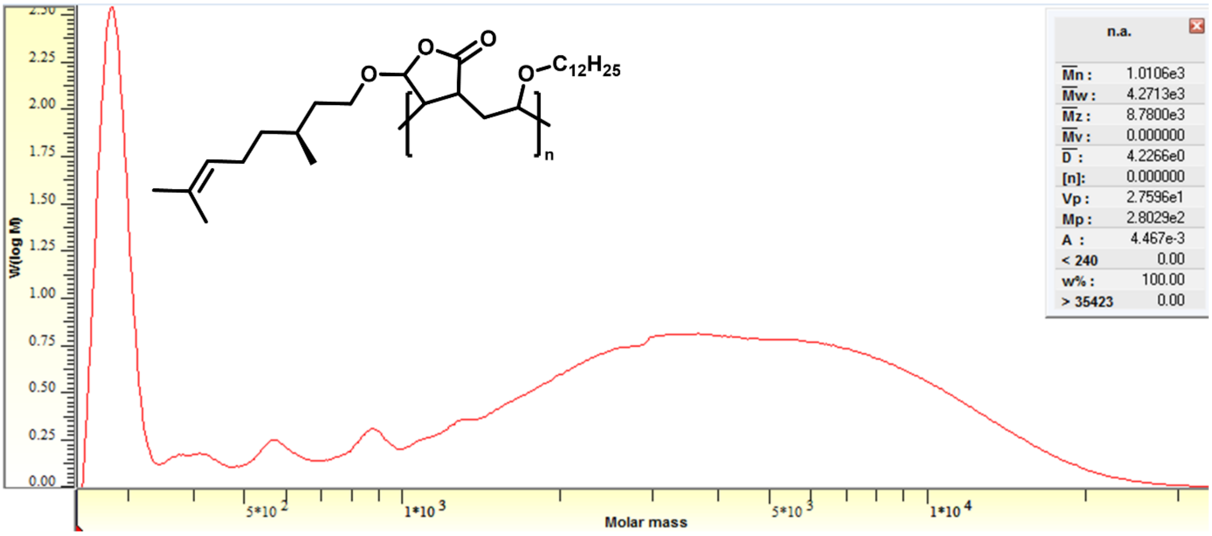

Figure S79. GPC spectrum (using THF as stationary phase) of poly(citronelloxy butenolide-co-dodecyl vinyl ether) (**4-DVE**).

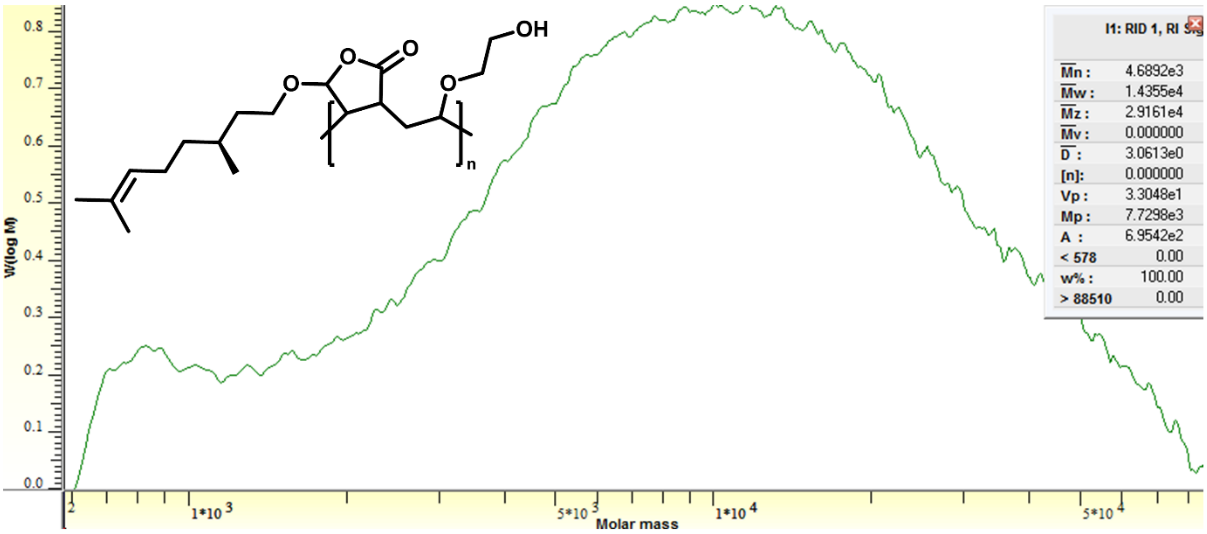

Figure S80. GPC spectrum (using DMF as stationary phase) of poly(citronelloxy butenolide-co-ethylene glycol vinyl ether) (**4-EGVE**).

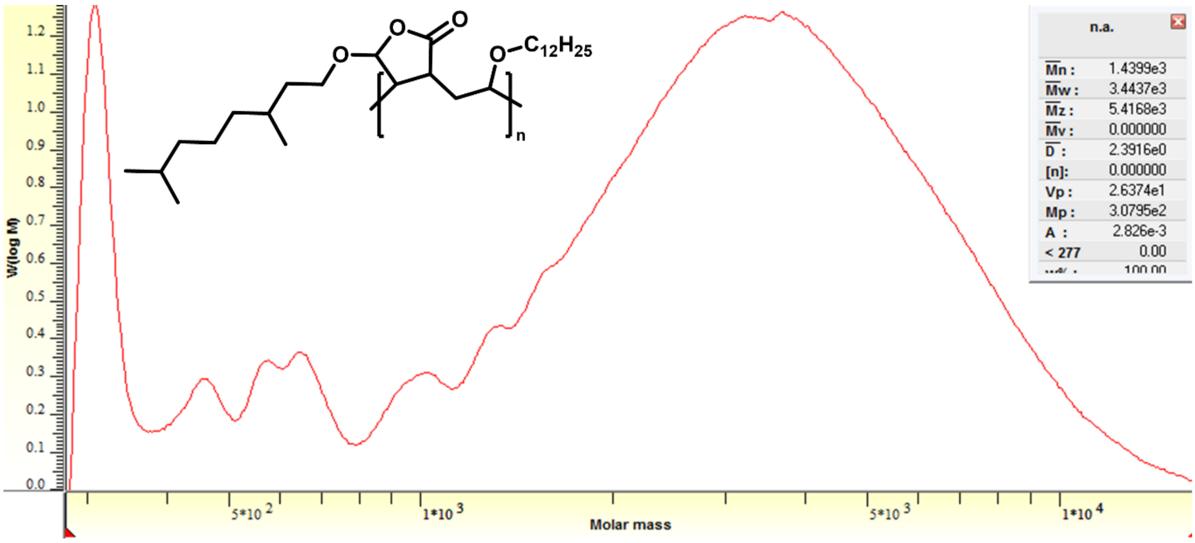

Figure S81. GPC spectrum (using THF as stationary phase) of poly(dihydrocitronelloxy butenolide-co-dodecyl vinyl ether) (**5-DVE**).

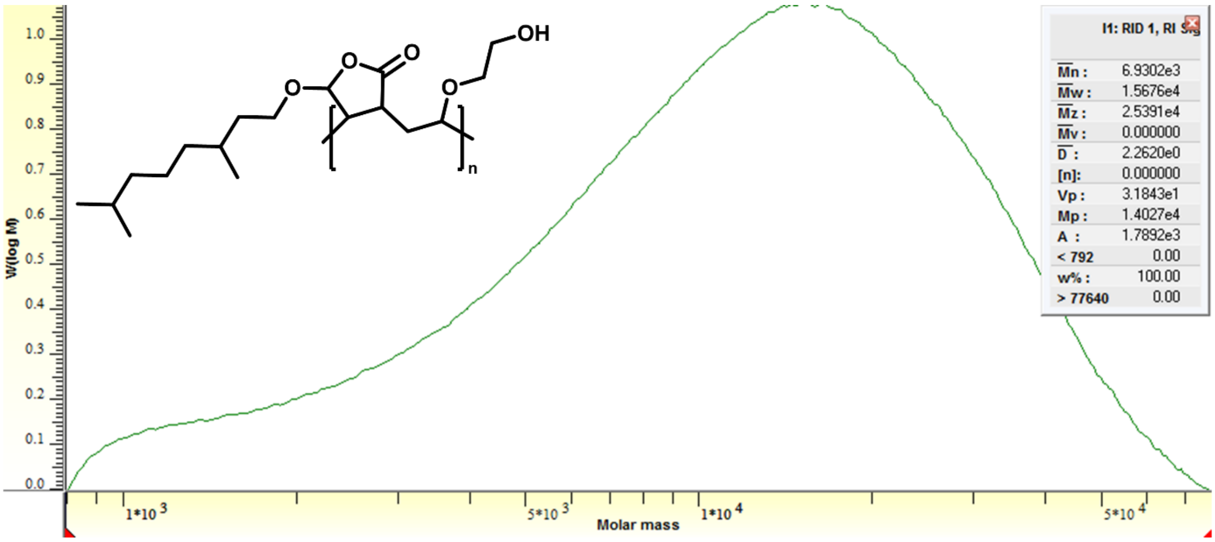

Figure S82. GPC spectrum (using DMF as stationary phase) of poly(dihydrocitronelloxy butenolide-co-ethylene glycol vinyl ether) (**5-EGVE**).

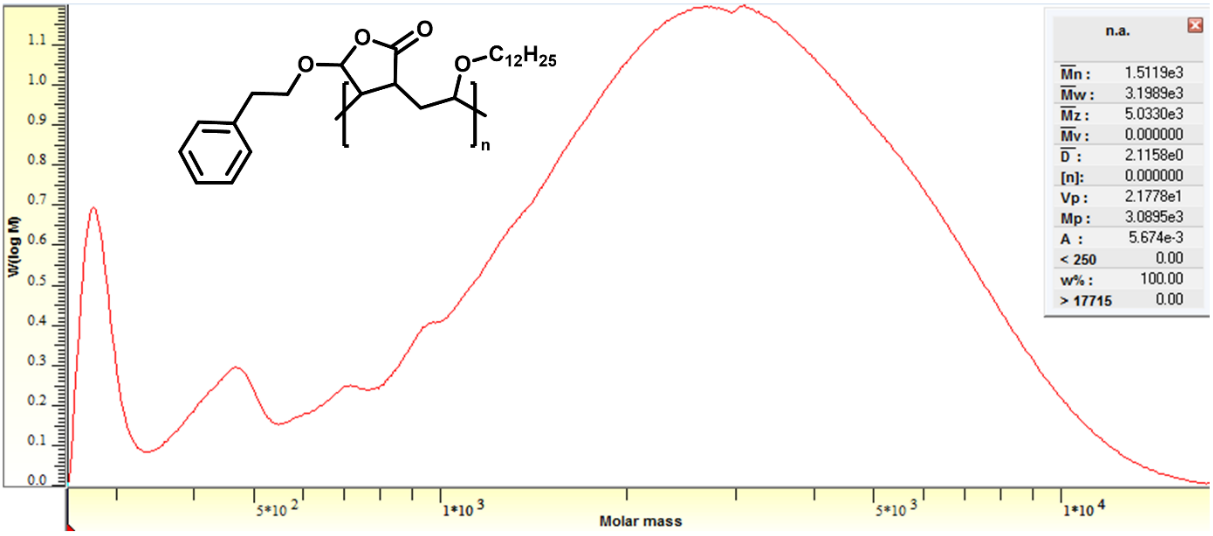

Figure S83. GPC spectrum (using THF as stationary phase) of poly(phenylethoxy butenolide-co-dodecyl vinyl ether) (**6-DVE**).

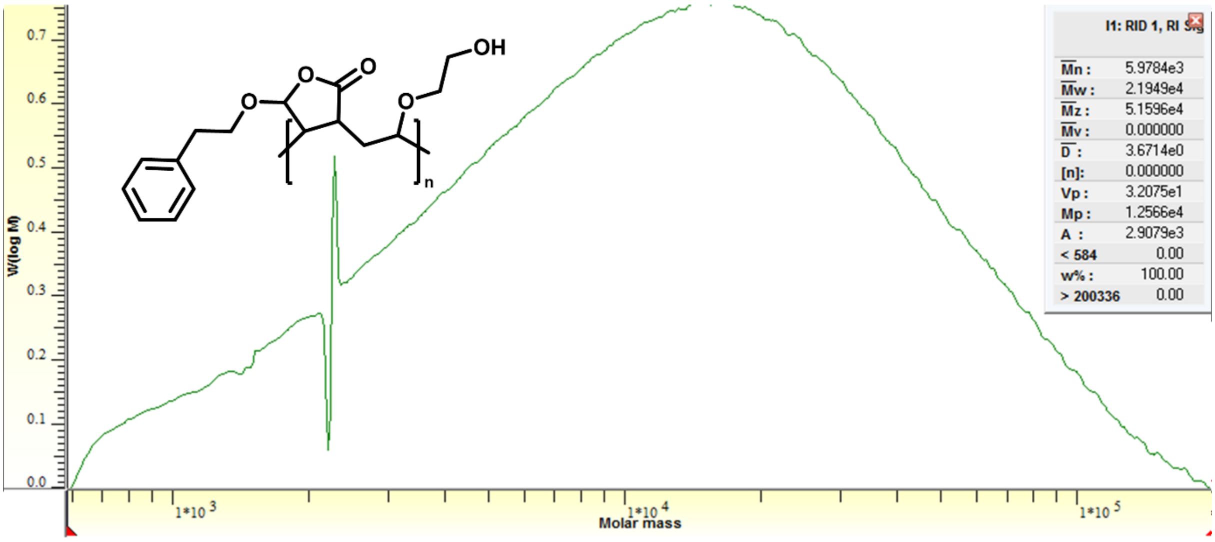

Figure S84. GPC spectrum (using DMF as stationary phase) of poly(phenylethoxy butenolide-co-ethylene glycol vinyl ether) (**6-EGVE**).

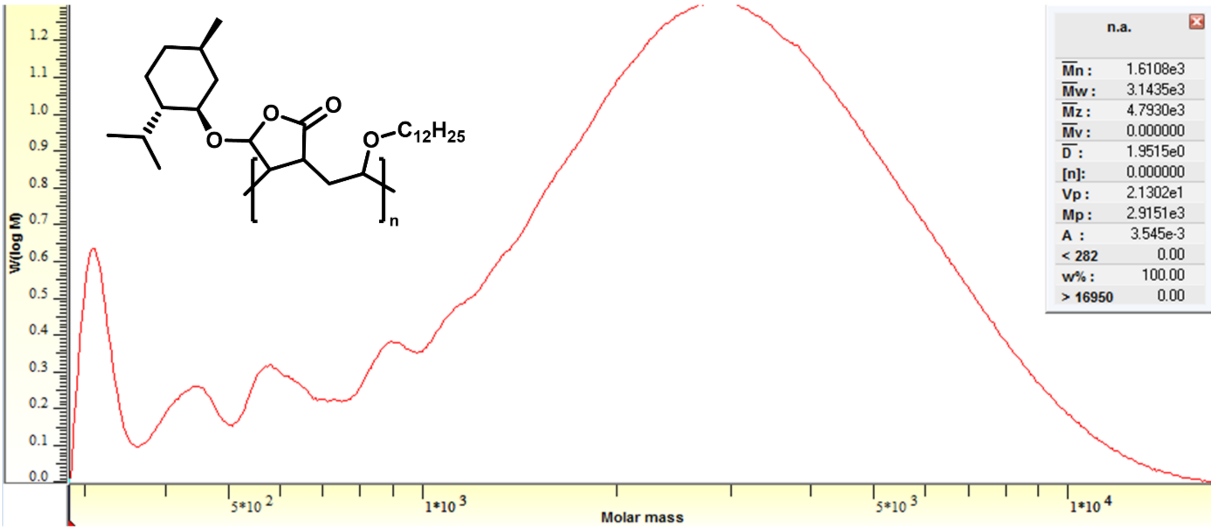

Figure S85. GPC spectrum (using THF as stationary phase) of poly(menthyloxy butenolide-co-dodecyl vinyl ether) (**7-DVE**).

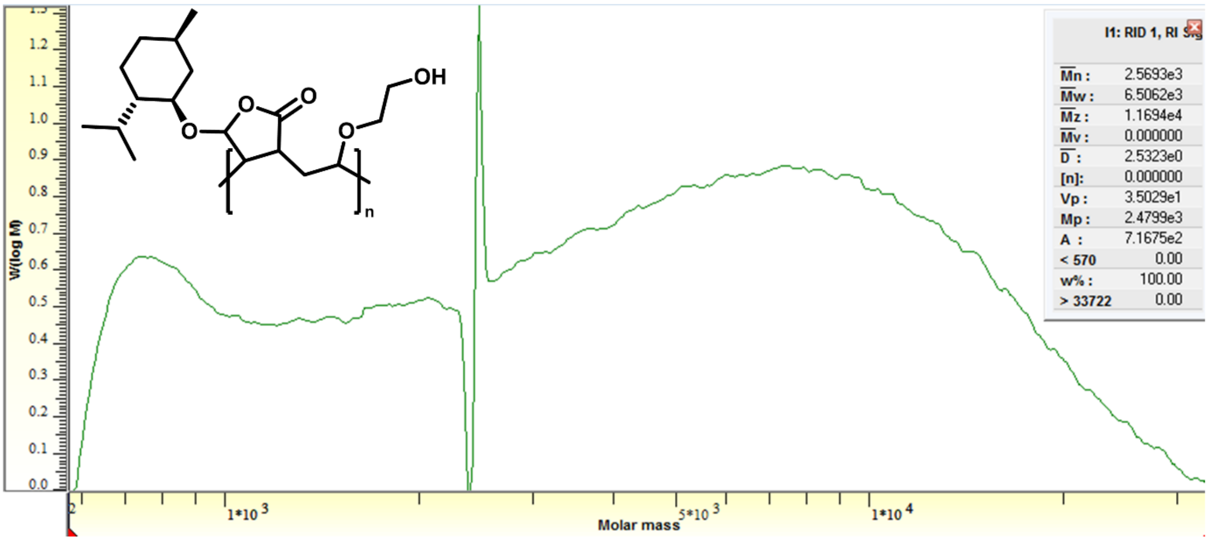

Figure S86. GPC spectrum (using DMF as stationary phase) of poly(menthyloxy butenolide-co-ethylene glycol vinyl ether) (**7-EGVE**).

Differential Scanning calorimetry (DSC)

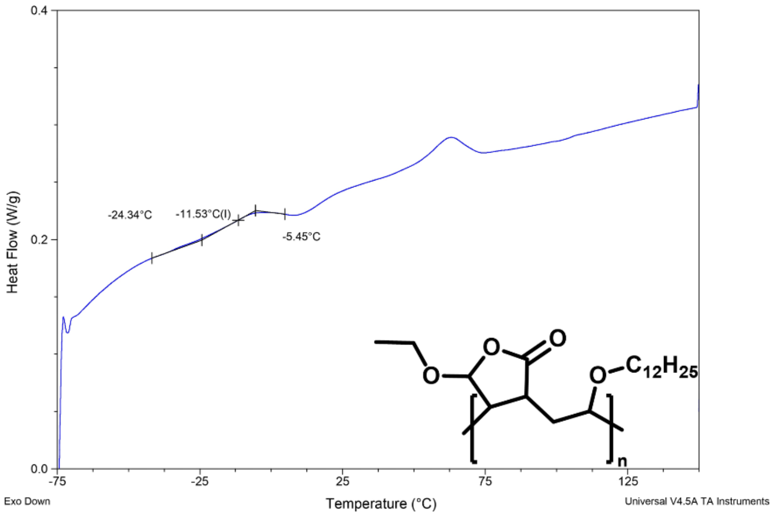

Figure S87. DSC spectrum of poly(ethoxy butenolide-co-dodecyl vinyl ether) (**1-DVE**).

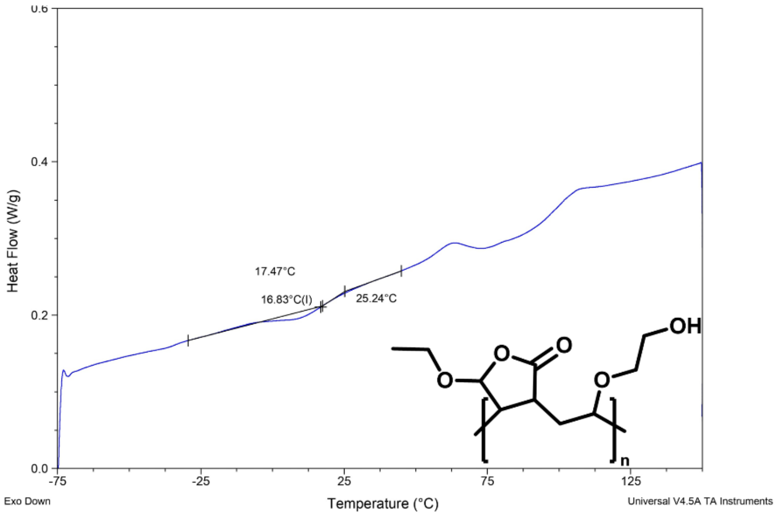

Figure S88. DSC spectrum of poly(ethoxy butenolide-co-ethylene glycol vinyl ether) (**1-EGVE**).

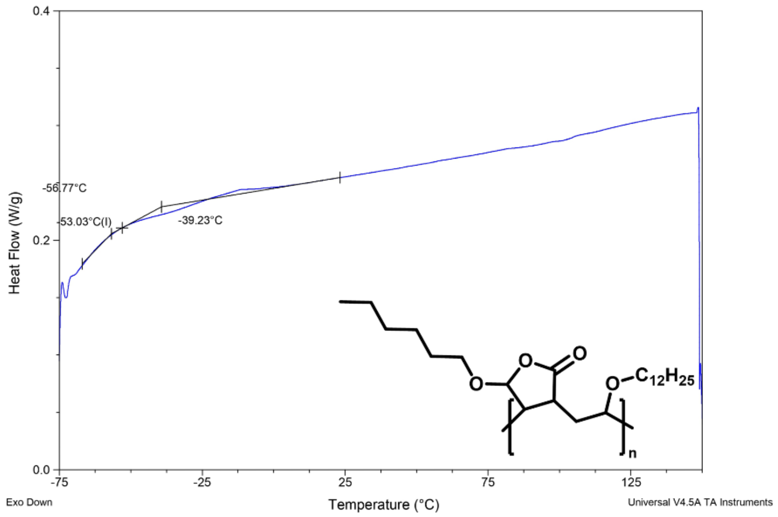

Figure S89. DSC spectrum of poly(hexyloxy butenolide-co-dodecyl vinyl ether) (**2-DVE**).

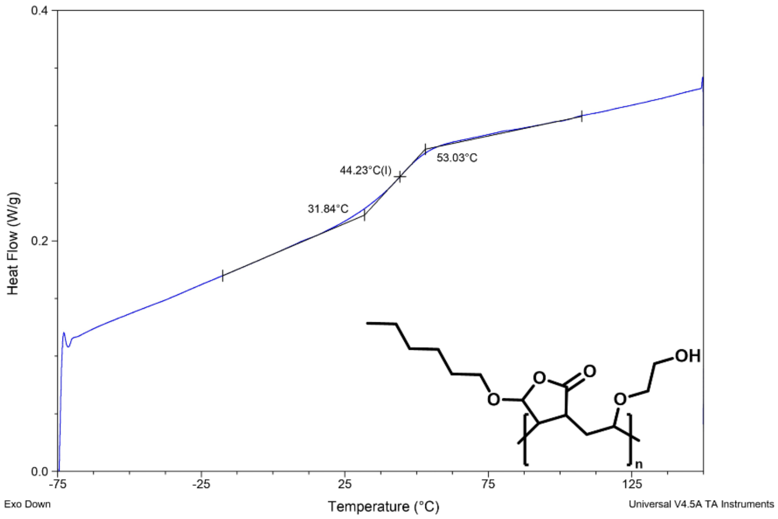

Figure S90. DSC spectrum of poly(hexyloxy butenolide-co-ethylene glycol vinyl ether) (**2-EGVE**).

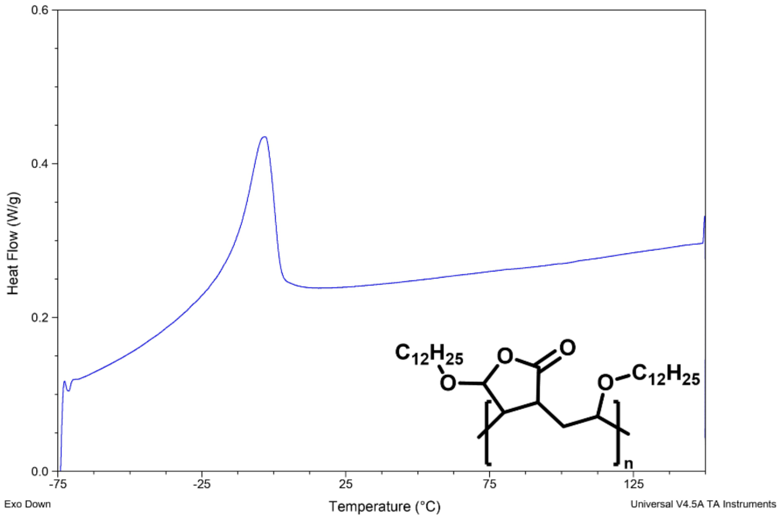

Figure S91. DSC spectrum of poly(dodecyloxy butenolide-co-dodecyl vinyl ether) (**3-DVE**).

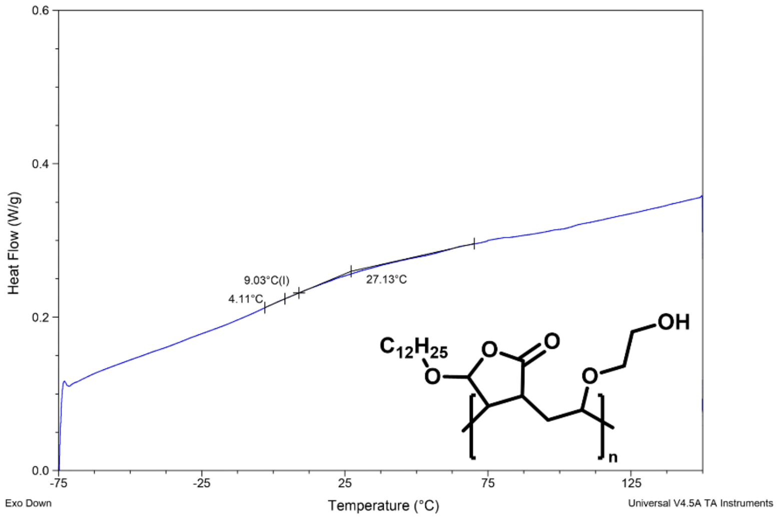

Figure S92. DSC spectrum of poly(dodecyloxy butenolide-co-ethylene glycol vinyl ether) (**3-EGVE**).

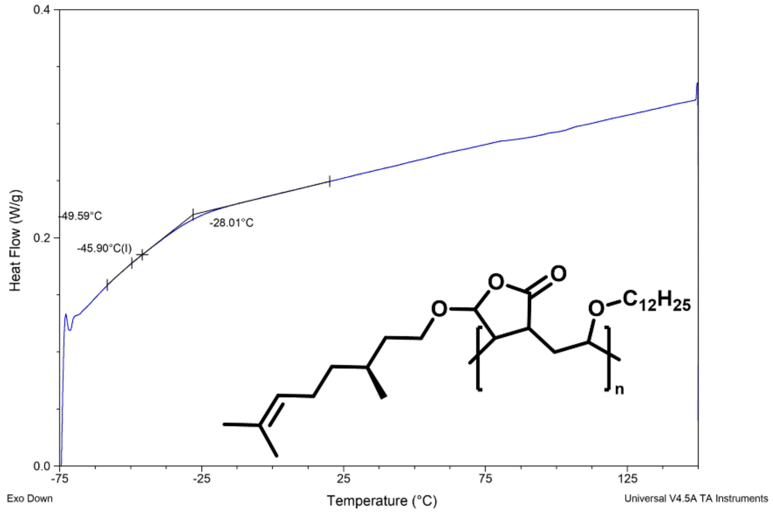

Figure S93. DSC spectrum of poly(citronelloxy butenolide-co-dodecyl vinyl ether) (**4-DVE**).

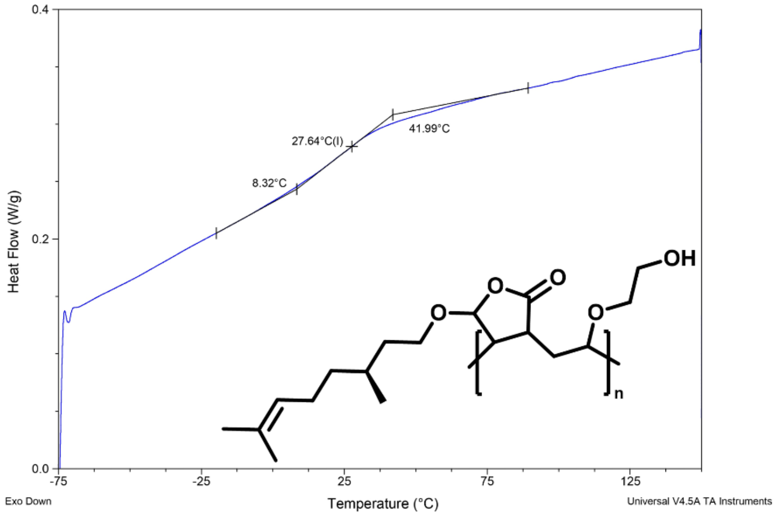

Figure S94. DSC spectrum of poly(citronelloxy butenolide-co-ethylene glycol vinyl ether) (**4-EGVE**).

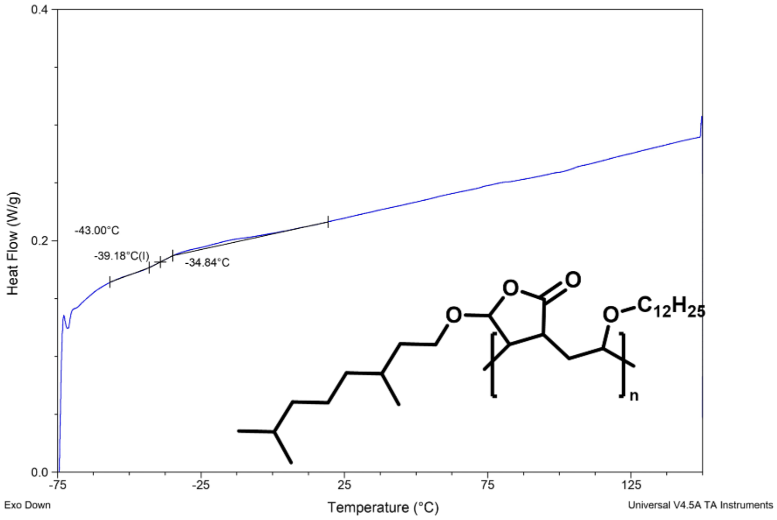

Figure S95. DSC spectrum of poly(dihydrocitronelloxy butenolide-co-dodecyl vinyl ether) (**5-DVE**).

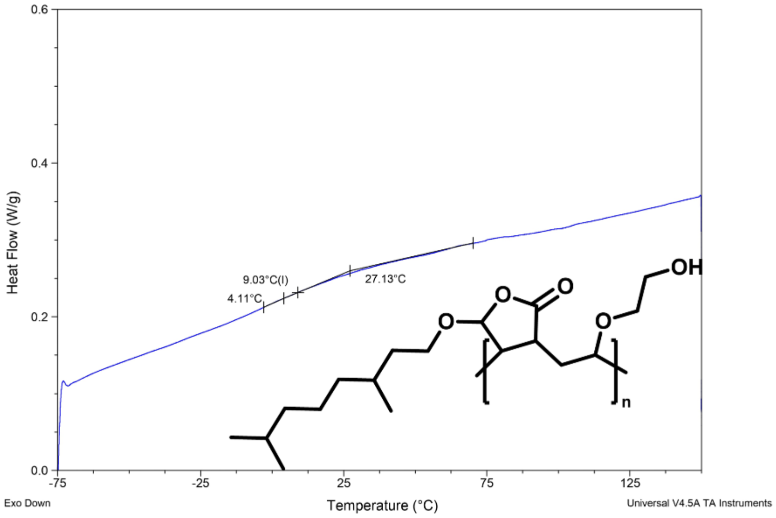

Figure S96. DSC spectrum of poly(dihydrocitronelloxy butenolide-co-ethylene glycol vinyl ether) (**5-EGVE**).

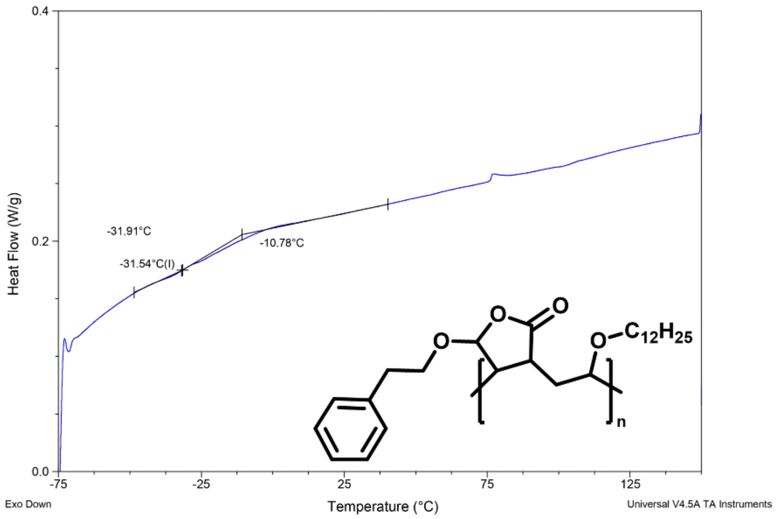

Figure S97. DSC spectrum of poly(phenylethoxy butenolide-co-dodecyl vinyl ether) (**6-DVE**).

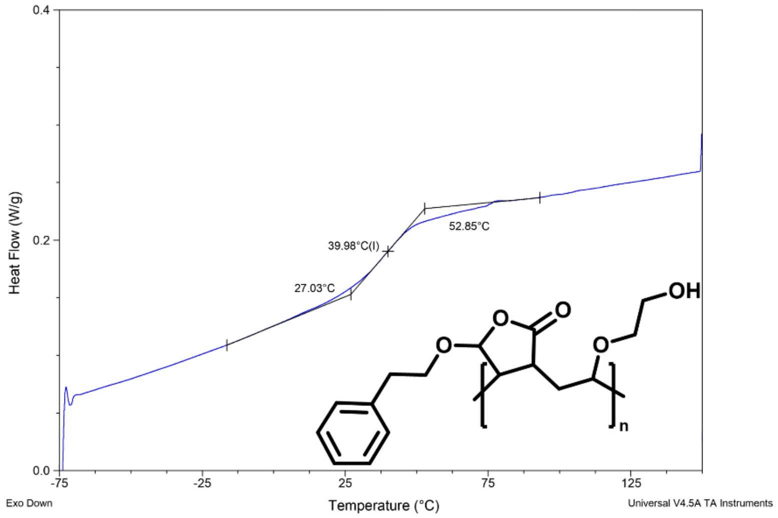

Figure S98. DSC spectrum of poly(phenylethoxy butenolide-co-ethylene glycol vinyl ether) (**6-EGVE**).

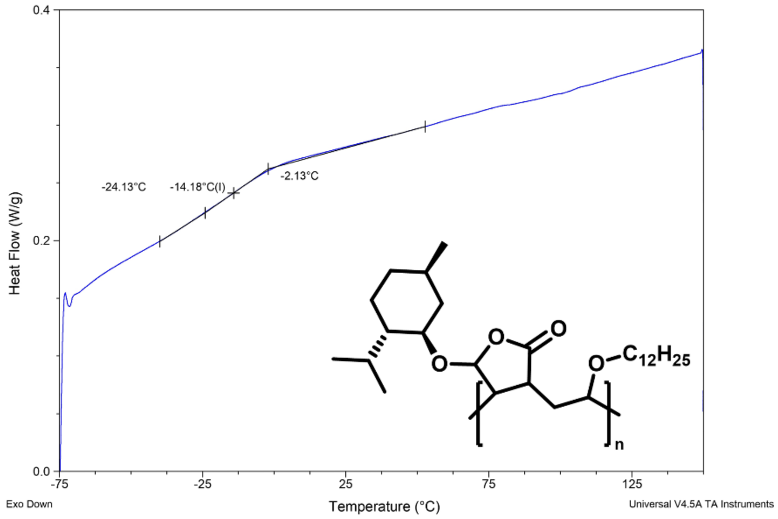

Figure S99. DSC spectrum of poly(menthyloxy butenolide-co-dodecyl vinyl ether) (**7-DVE**).

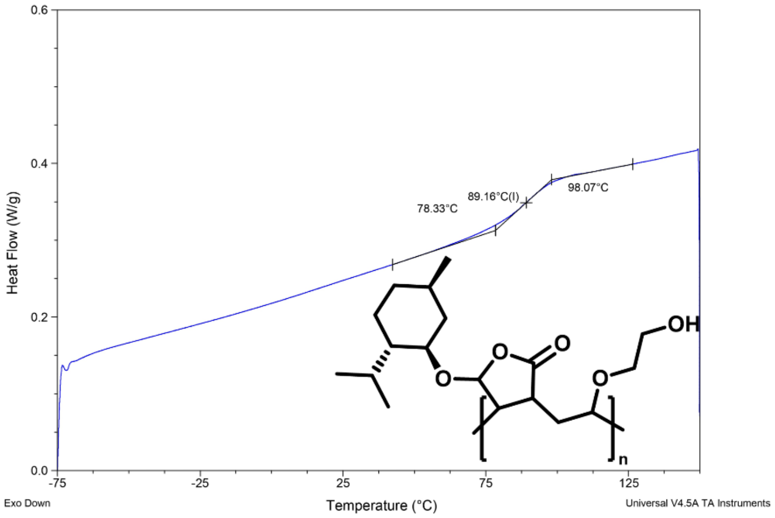

Figure S100. DSC spectrum of poly(menthyloxy butenolide-co-ethylene glycol vinyl ether) (**7-EGVE**).

Release of alcohols from butenolide monomers

Release of alcohols from ethoxy butenolide and hexyloxy butenolide in aqueous media were followed by ^{1}H-NMR on a 400 MHz spectrometer (typically D1 = 5, ns = 8) using DMSO-d_6. The amount of released alcohol was calculated through the ratio between the integrals of the alkoxy butenolide and the hydroxy butenolide peak in the ^{1}H-NMR spectrum. The following ^{1}H-NMR shifts were used:

Table S2. Followed ^{1}H-NMR shifts in alcohol release from alkoxy butenolide monomers.

Compound	^{1}H-NMR shift
Hydroxy butenolide	δ 6.29 (1H)
Ethoxy butenolide	δ 6.39 (1H)
Hexyloxy butenolide	δ 6.38 (1H)

Ethoxy butenolide (pH 1 / 10 mol% H⁺)

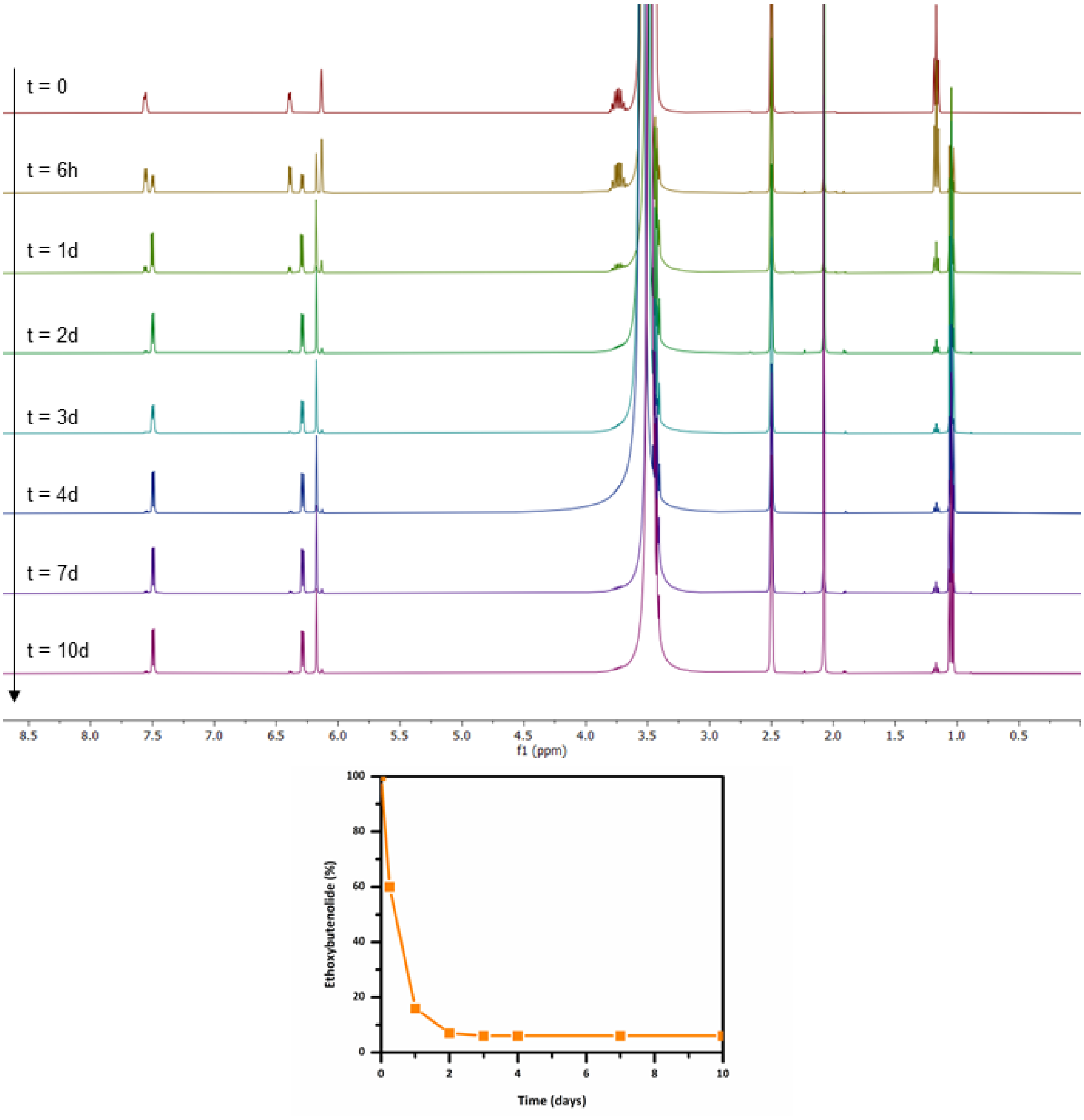

Figure S101. Hydrolysis of ethoxy butenolide in aqueous media (10 mol% HCl) followed by ^{1}H-NMR (in DMSO-d_6, top) and plotted as a function of time (bottom).

Ethoxy butenolide (pH 2 / 1 mol% H$^+$)

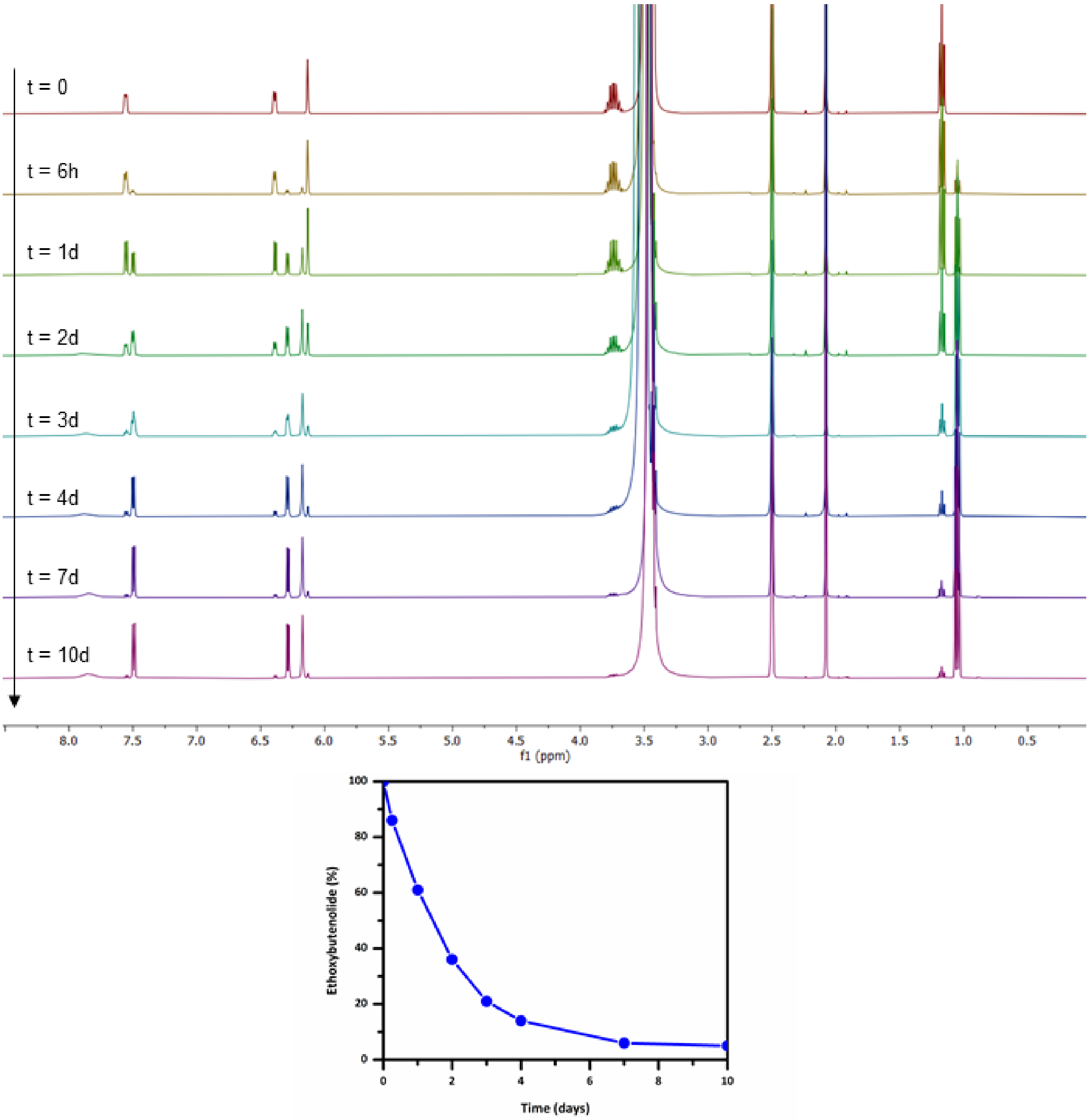

Figure S102. Hydrolysis of ethoxy butenolide in aqueous media (1 mol% HCl) followed by ^{1}H-NMR (in DMSO-d_6, top) and plotted as a function of time (bottom).

Ethoxy butenolide (pH 6 / 0.0001 mol% H^+)

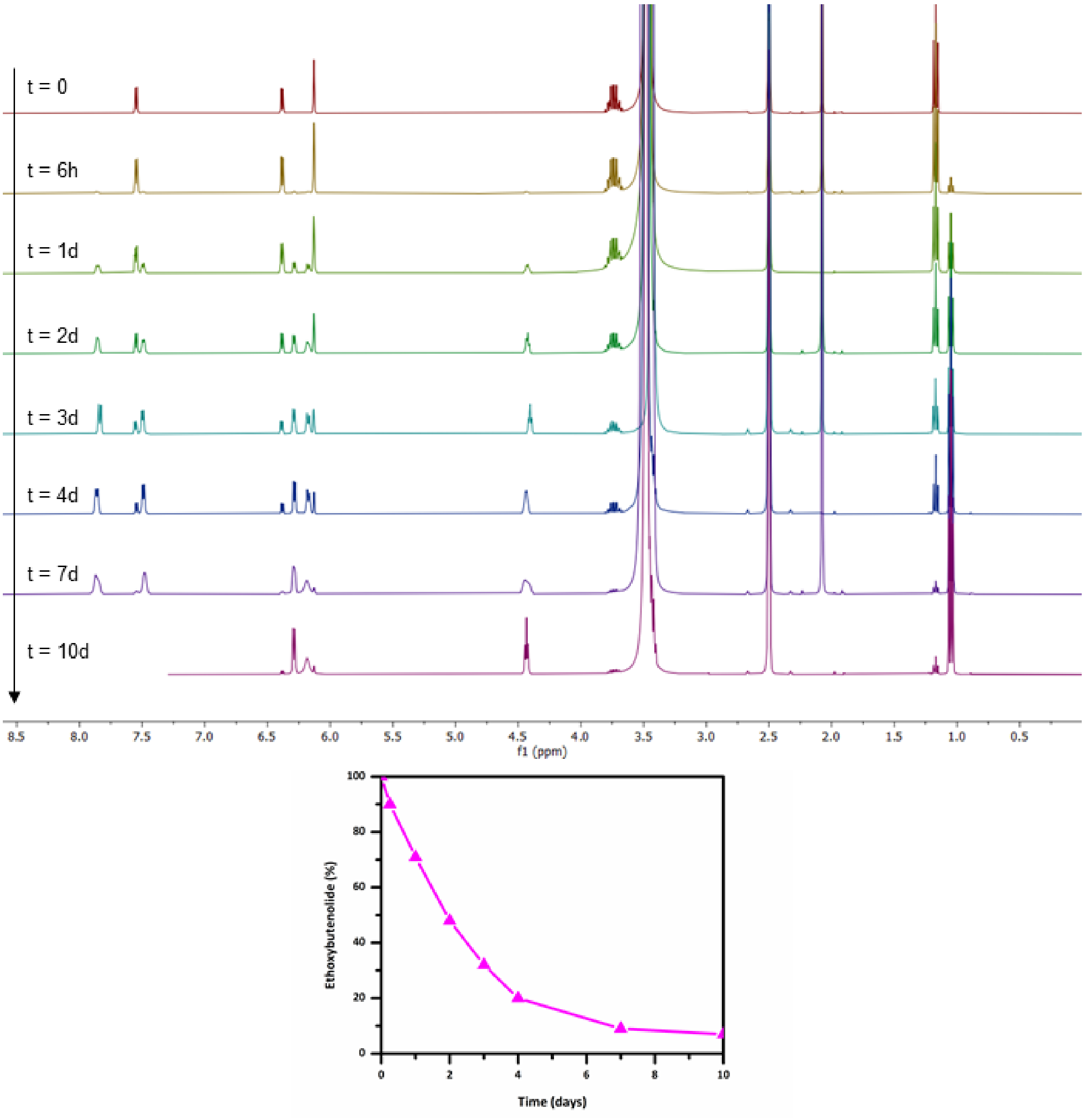

Figure S103. Hydrolysis of ethoxy butenolide in aqueous media (0.0001 mol% HCl) followed by ^{1}H-NMR (in DMSO-d_6, top) and plotted as a function of time (bottom). Note: the ^{1}H-NMR spectrum at 10 d was locked on the wrong solvent, causing a significant shift in the spectrum.

Hexyloxy butenolide (pH 1 / 10 mol% H$^+$)

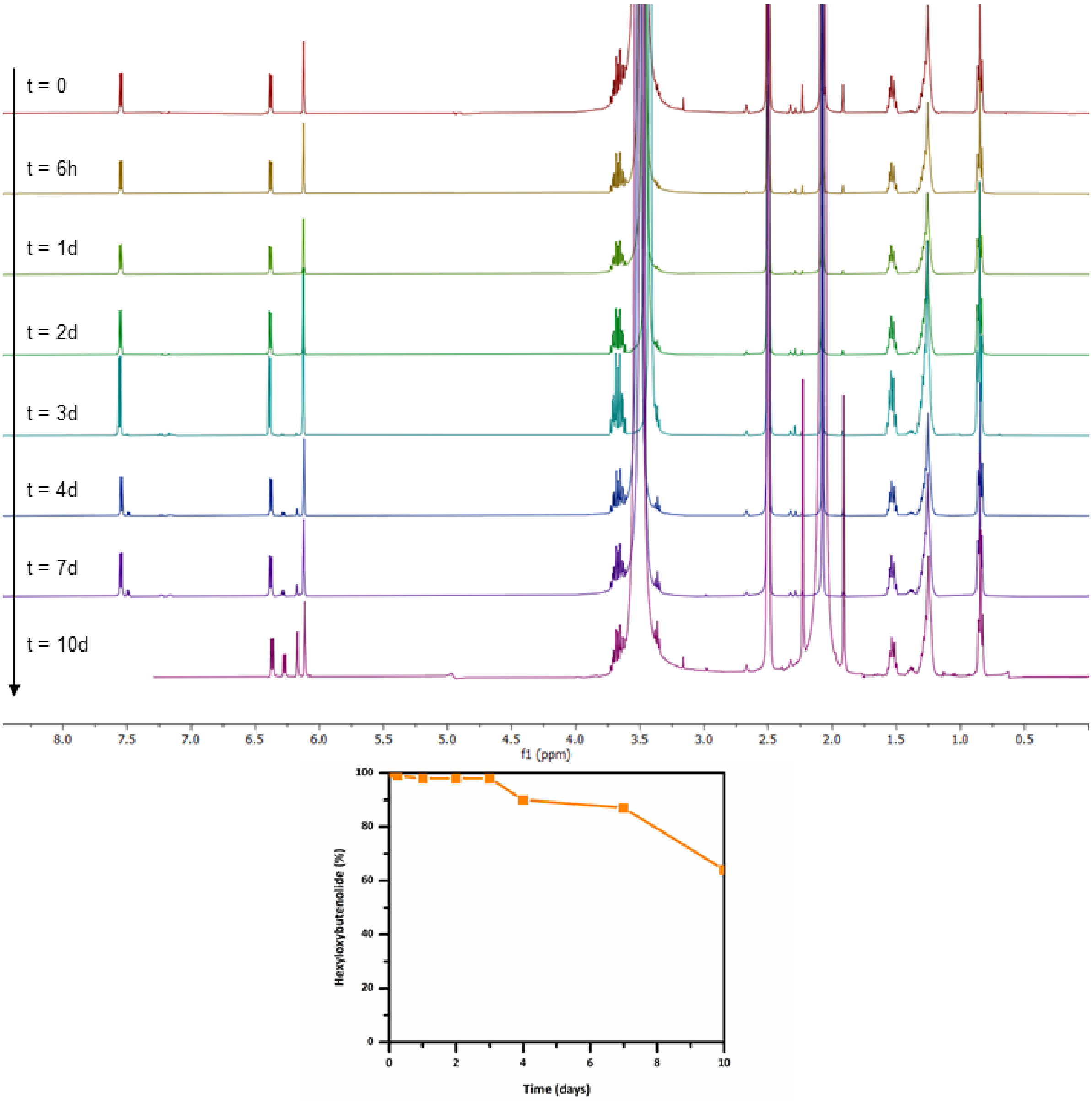

Figure S104. Hydrolysis of hexyloxy butenolide in aqueous media (10 mol% HCl) followed by ^{1}H-NMR (in DMSO-d_6, top) and plotted as a function of time (bottom). Note: the ^{1}H-NMR spectrum at 10 d was locked on the wrong solvent, causing a significant shift in the spectrum.

Hexyloxy butenolide (pH 2 / 1 mol% H⁺)

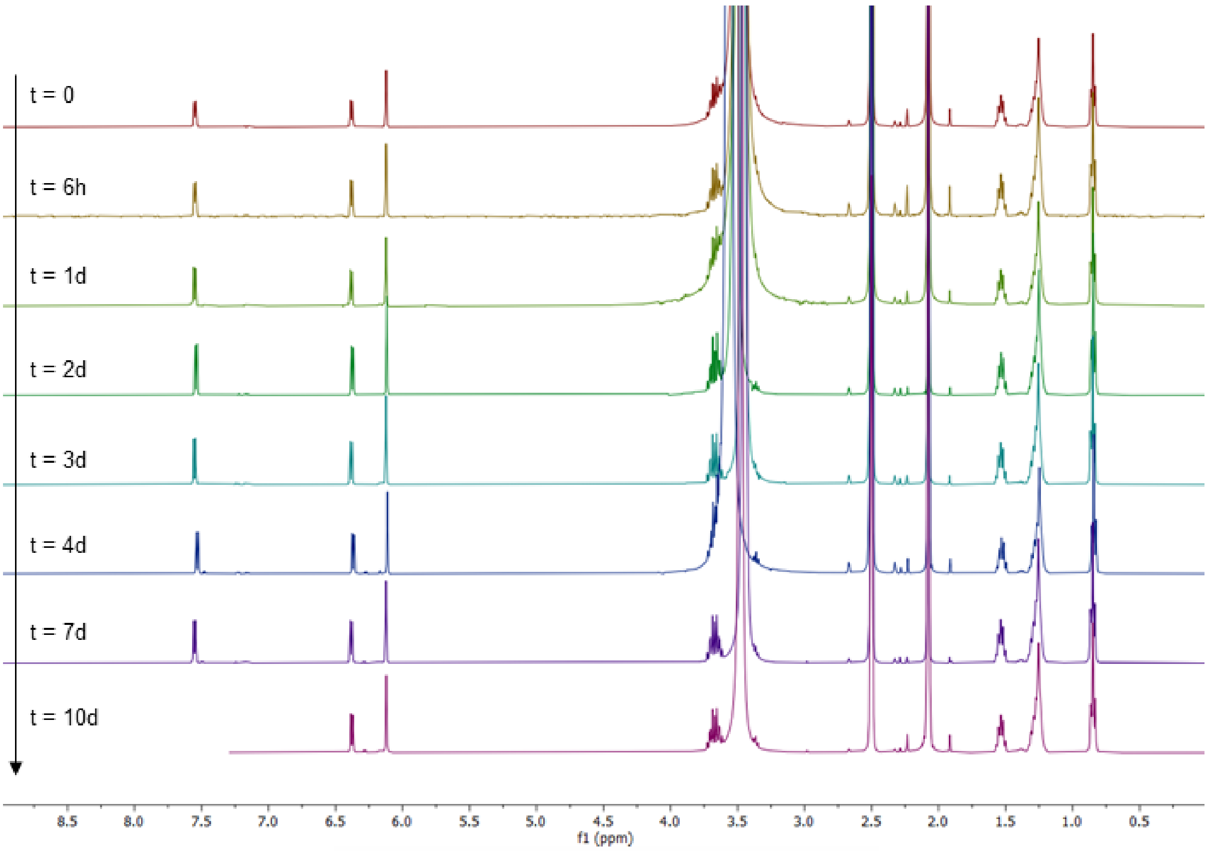

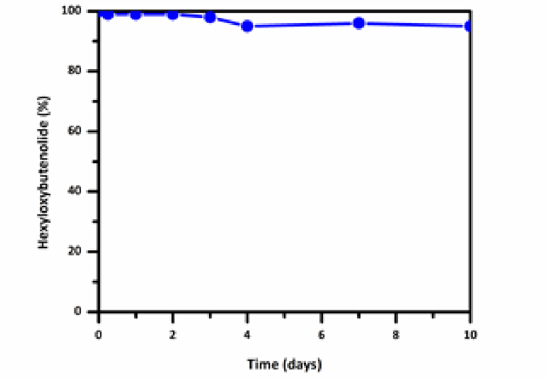

Figure S105. Hydrolysis of hexyloxy butenolide in aqueous media (1 mol% HCl) followed by ^{1}H-NMR (in DMSO-d_6, top) and plotted as a function of time (bottom). Note: the ^{1}H-NMR spectrum at 10 d was locked on the wrong solvent, causing a significant shift in the spectrum.

Hexyloxy butenolide (pH 6 / 0.0001 mol% H$^+$)

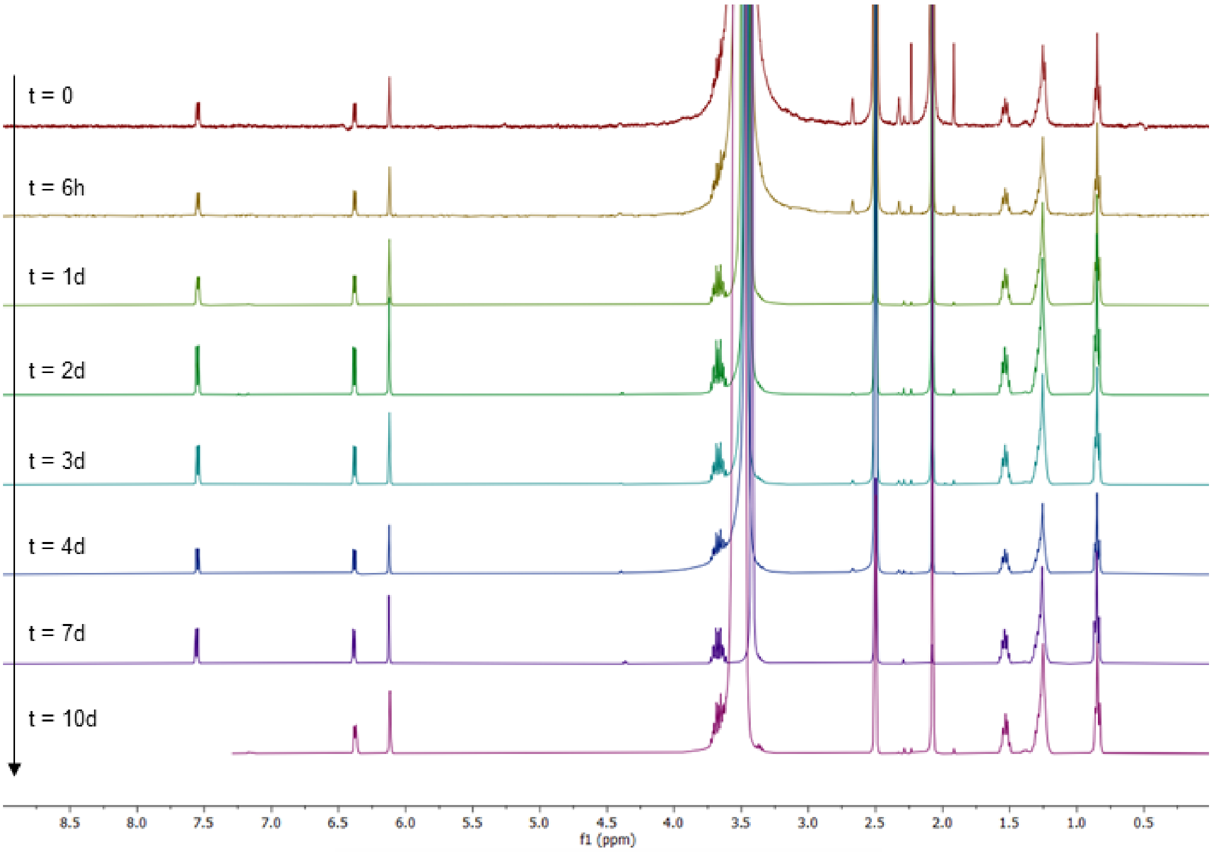

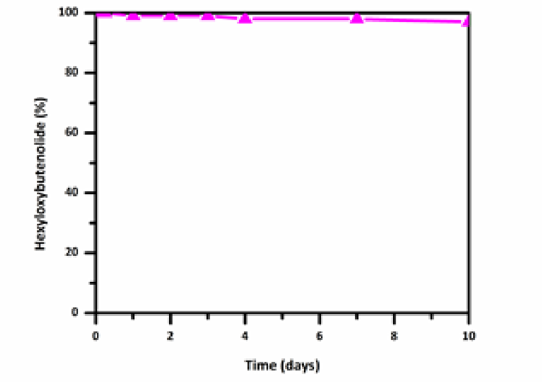

Figure S106. Hydrolysis of hexyloxy butenolide in aqueous media (0.0001 mol% HCl) followed by ^{1}H-NMR (in DMSO-d$_6$, top) and plotted as a function of time (bottom). Note: the ^{1}H-NMR spectrum at 10 d was locked on the wrong solvent, causing a significant shift in the spectrum.

Release of alcohols from butenolide copolymers and coatings

Release of alcohols from butenolide co-polymers in aqueous media were quantified by headspace GC-MS. First, a calibration curve of known quantities of alcohol in water were made. Using this calibration curve the amount of alcohol release from the butenolide co-polymers could be quantified. The quantities of release are shown in this section, including an example headspace GC-MS measurement and the calibration curves.

Summary of alcohol release from co-polymers and coatings

Table S3. Ethanol release from of poly(ethoxy butenolide-co-dodecyl vinyl ether) (**1-DVE**) in water with different acid concentrations after 10 d. The area of the ethanol peak and the calculated release as a percentage of the total amount of ethoxy butenolide are shown.

Acid Conc.	Vial 1		Vial 2		Vial 3	
	Area	Release (%)	Area	Release (%)	Area	Release (%)
H_2O	461548	16.14	490549	16.07	562513	20.67
10 mol% HCl	605916	22.62	692174	26.49	728197	28.11
100 mol% HCl	2185622	93.52	2046706	87.286	2132943	91.16

Table S4. Ethanol release from of poly(ethoxy butenolide-co-ethylene glycol vinyl ether) (**1-EGVE**) in water with different acid concentrations after 10 d. The area of the ethanol peak and the calculated release as a percentage of the total amount of ethoxy butenolide are shown.

Acid Conc.	Vial 1		Vial 2		Vial 3	
	Area	Release (%)	Area	Release (%)	Area	Release (%)
H_2O	276743	7.849	309836	9.334	650796	24.64
10 mol% HCl	928238	37.09	730678	28.22	963971	38.69
100 mol% HCl	1950423	82.96	1897345	80.58	2174902	93.04

Table S5. Hexanol release from of poly(hexyloxy butenolide-co-dodecyl vinyl ether) (**2-DVE**) in water with different acid concentrations after 10 d. The area of the hexanol peak and the calculated release as a percentage of the total amount of hexyloxy butenolide are shown.

Acid Conc.	Vial 1		Vial 2		Vial 3	
	Area	Release (%)	Area	Release (%)	Area	Release (%)
H_2O	20017	0.193	70451	0.320	74038	0.329
10 mol% HCl	27818	0.212	52199	0.274	37839	0.238
100 mol% HCl	47753	0.263	108176	0.415	32407	0.224

Table S6. Ethanol release from of poly(hexyloxy butenolide-co-ethylene glycol vinyl ether) (**2-EGVE**) in water with different acid concentrations after 10 d. The area of the hexanol peak and the calculated release as a percentage of the total amount of hexyloxy butenolide are shown.

Acid Conc.	Vial 1		Vial 2		Vial 3	
	Area	Release (%)	Area	Release (%)	Area	Release (%)
H_2O	343731	1.008	349612	1.023	368460	1.071
10 mol% HCl	307918	0.918	339038	0.996	394758	1.137
100 mol% HCl	618411	1.700	738619	2.003	779138	2.105

Table S7. Kinetic data for hexanol release from of poly(hexyloxy butenolide-co-dodecyl vinyl ether) (**2-DVE**) in water over time. The area of the hexanol peak and the calculated release as a percentage of the total amount of hexyloxy butenolide are shown.

Day	Vial 1		Vial 2		Vial 3	
	Area	Release (%)	Area	Release (%)	Area	Release (%)
2	17930	0.187	12973	0.175	71504	0.322
5	24778	0.205	65618	0.308	72077	0.324
10	20017	0.193	70451	0.320	74038	0.329
20	27971	0.213	34598	0.229	34293	0.229
40	50805	0.270	52397	0.274	61231	0.297

Table S8. Kinetic data for hexanol release from of poly(hexyloxy butenolide-co-ethylene glycol vinyl ether) (**2-EGVE**) in water over time. The area of the hexanol peak and the calculated release as a percentage of the total amount of hexyloxy butenolide are shown.

Day	Vial 1		Vial 2		Vial 3	
	Area	Release (%)	Area	Release (%)	Area	Release (%)
2	194659	0.633	254586	0.784	242403	0.753
5	283571	0.857	275548	0.836	274960	0.835
10	343731	1.008	349612	1.023	368460	1.071
20	480216	1.352	446340	1.267	506830	1.419
40	596800	1.646	607162	1.672	635107	1.742

Table S9. Alcohol release from of poly(alkoxy butenolide-co-ethylene glycol vinyl ether) (**5-EGVE, 6-EGVE and 7-EGVE**) in water after 10 d. The area of the alcohol peak and the calculated release as a percentage of the total amount of alkoxy butenolide are shown.

Alcohol	Vial 1		Vial 2		Vial 3	
	Area	Release (%)	Area	Release (%)	Area	Release (%)
Dihydrocitronellol	781854	0.578	626134	0.439	550590	0.371
Phenylethanol	654654	1.861	683074	1.904	724766	1.969
Menthol	55943	0.114	56977	0.115	50894	0.109

Table S10. Alcohol release from of **2-EGVE** and **6-EGVE** polymers and coatings in water after 10 d. The area of the alcohol peak and the calculated release as mmol and as a percentage of the total amount of alkoxy butenolide (2 mmol) are shown.

Alcohol	Area	Release (mmol)	Release (%)
Hexanol (polymer)	343731	0.02016	1.008
Hexanol (coating)	128996	0.009345	0.467
Phenylethanol (polymer)	654654	0.03809	1.861
Phenylethanol (coating)	137276	0.02122	1.061

Odor Detection Threshold and Odor Perception Evaluation

The odor detection threshold (ODT) is defined as the lowest concentration at which an odor can be distinguished from a blank stimulus. It corresponds to the concentration at which half of a test panel can detect the odor. The ODT is determined using dynamic olfactometry, a European standardized method (EN 13725) based on the human sense of smell.[37] In this method, a panel of examiners is exposed to successive dilutions of an odor sample with odor-free air. The ODT is expressed in European odor units per cubic meter (ou_E/m^3). For example, if an odor sample must be diluted 80 times before it becomes non-perceptible, its concentration is 80 ou_E/m^3. Although this technique allows quantitative odor measurement, it does not distinguish between different odor types.

Odors are perceived when volatile molecules bind to receptors in the nose, activating the olfactory receptor neurons located in the olfactory epithelium. This tissue, which measures approximately 10 cm^2 in humans and about 170 cm^2 in dogs, transmits signals through the olfactory bulb to the brain, where odors are recognized. These signals can also trigger memories and emotions due to neural connections near the olfactory bulb.[38] For an odor to be perceived, its concentration in air must exceed the ODT so that enough molecules interact with the olfactory receptors.

Cometto-Muñiz *et al.* studied the concentration–detection functions of homologous *n*-alcohols (ethanol, 1-butanol, 1-hexanol, and 1-octanol) using an 8-station vapor delivery device (VDD-8).[39] This system generated and delivered odor vapors to subjects in increasing concentrations. Each station consisted of three cones, one of which released the odor (the active cone), while the others delivered carbon-filtered air (blanks). The active cone was randomly assigned, and subjects were asked to identify which cone smelled different, rating their confidence each time. Measurements were repeated 21 times per odorant, and the vapor concentrations were determined by gas chromatography before and during the tests to ensure accurate quantification. The study found that ODTs shifted to lower concentrations with increasing carbon chain length of the alcohols.

Table S11. Odor detection thresholds (ODT) of *n*-alcohols.[39-41]

Alcohol	ODT (ppb)
Methanol	100 ppm
Ethanol	331 ppb
1-Butanol	8 ppb
1-Hexanol	8 ppb
1-Octanol	4 ppb

from the polymers and coatings would be detectable. To estimate this, the **ideal gas law** was applied:

$$PV = nRT$$

In this equation, pressure (P), temperature (T), and the gas constant (R) are constants under standard conditions. Therefore, only the volume (V) and the number of released odor molecules (n) vary. The coatings had a surface area of roughly 0.0066 m², and odor detection was evaluated at a distance of 0.30 m, corresponding to a volume of approximately 0.00198 m³. Then with the ideal gas law we can calculate the amount of moles present in the volume, which is 0.0823 moles. To be detectable, the coatings should generate concentrations of 8 ppb of 1-hexanol within that volume.

The released quantities of 1-hexanol obtained from earlier GC–MS measurements ($9.34*10^{-6}$ moles) were used to calculate the corresponding air concentrations in parts per billion (Equation 2) and parts per million (Equation 3). The corresponding values were calculated as 1.13*10

$$ppb = \frac{\text{amount of released odor molecules}}{\text{total amount of molecules in defined volume}} \times 10^9$$

$$ppm = \frac{\text{amount of released odor molecules}}{\text{total amount of molecules in defined volume}} \times 10^6$$

To estimate how long the odor molecules remained in the defined volume, diffusion coefficients from Lapuearta *et al.* were used.[42] At 25 °C, the diffusion coefficients in air are 0.1777 cm²/s for methanol and 0.0532 cm²/s for *n*-pentanol, the latter applied as an approximation for 1-hexanol because shorter-chain alcohols diffuse faster. Both values indicate that the odor molecules move less than 0.2 cm² per second, meaning they remain within the 1980 cm³ volume for several minutes, neglecting airflow.

Table S12. Concentrations of odor molecules released within a volume of 1980 cm^3 after one minute.[39.40]

Sample	1-Hexanol (ODT = 8 ppb)
10-day coating release at neutral conditions	113 ppm

All 1-hexanol-releasing polymers and coatings produced detectable concentrations within 30 cm of the surface. Consistent with these calculations, five colleagues were able to perceive the odor of 1-hexanol in released samples, confirming the “smellability” of the coatings. These results demonstrate that acid-mediated acetal hydrolysis successfully triggered the release of perceivable odor molecules from the developed polymers and coatings, supporting their feasibility for future applications such as odor-releasing functional coatings.

Ethanol release from co-polymers 1-DVE and 1-EGVE

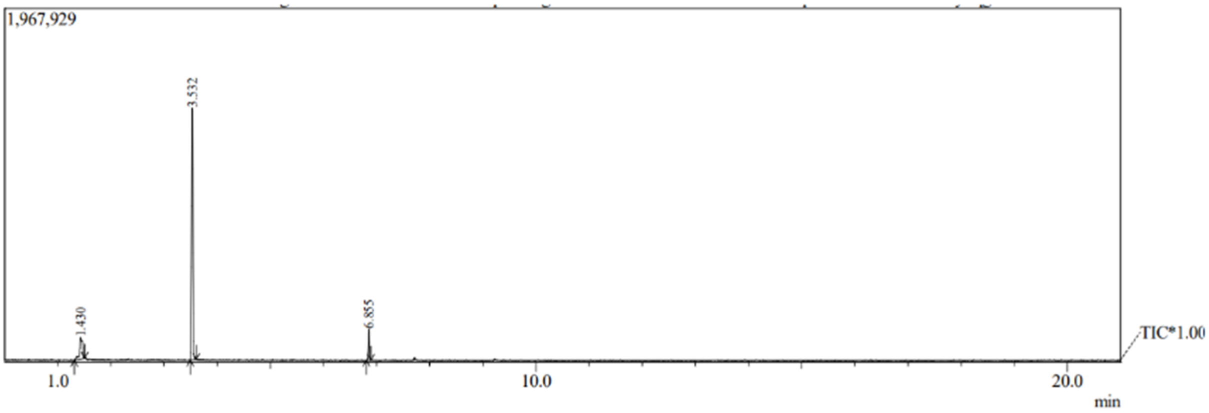

Peak Report TIC

Peak#	R.Time	Area	Area%	Height	Height%	Name	Base m/z	Base Int.
1	1.430	461548	15.24	120917	7.12	Ethanol	44.05	108926
2	3.532	2329057	76.92	1403091	82.65	Acetic acid, butyl ester	56.10	585086
3	6.855	237179	7.83	173553	10.22	Ether, 2-ethylhexyl tert-butyl	57.10	82945
		3027784	100.00	1697561	100.00			

(a)

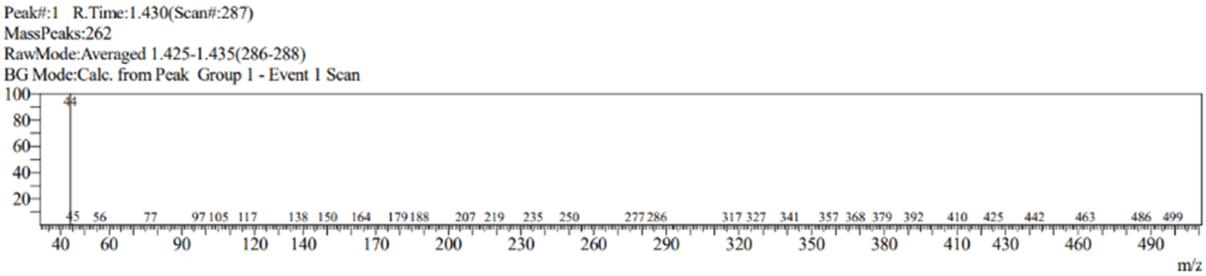

(b)

Figure S107. Ethanol release from **1-DVE** co-polymer measured by headspace GC-MS after 10 days mixing in water. (a) Headspace GC chromatogram. (b) MS diffraction of ethanol. It is important to note that the ethanol peak in the chromatogram overlaps with the signal of carbon dioxide from the air. However, since this is also the case for the calibration measurements, this is accounted for in the calculated release.

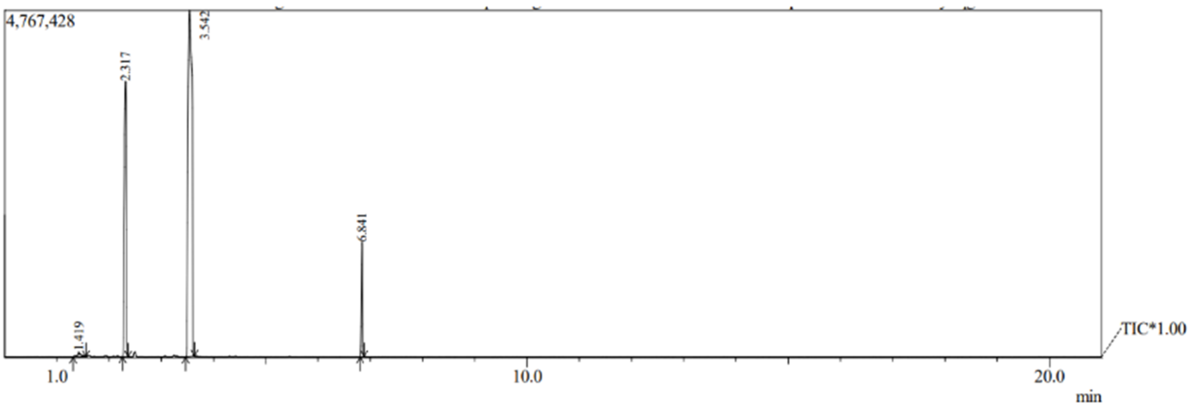

Peak Report TIC

Peak#	R.Time	Area	Area%	Height	Height%	Name	Base m/z	Base Int.
1	1.419	309836	0.83	72563	0.71	Ethanol	44.05	54329
2	2.317	10182770	27.26	3784199	37.15	Pentane, 2,2,4-trimethyl-	57.10	2373489
3	3.542	24627140	65.93	4752420	46.66	Acetic acid, butyl ester	56.10	1968827
4	6.841	2232207	5.98	1576781	15.48	Ether, 2-ethylhexyl tert-butyl	57.10	743773
		37351953	100.00	10185963	100.00			

(a)

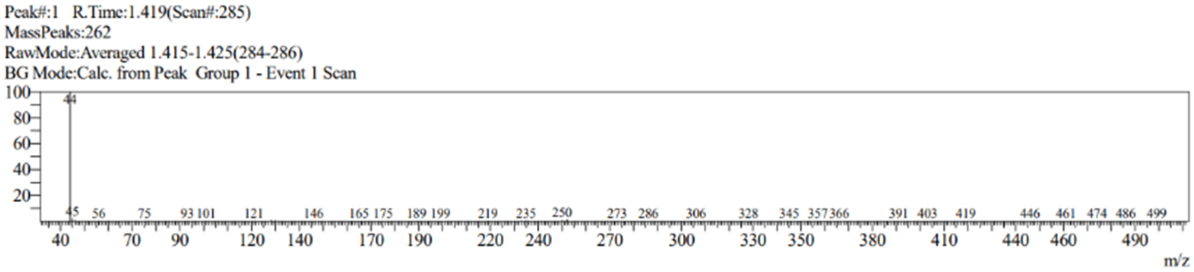

(b)

Figure S108. Ethanol release from **1-EGVE** co-polymer measured by headspace GC-MS after 10 days mixing in water. (a) Headspace GC chromatogram. (b) MS diffraction of ethanol. It is important to note that the ethanol peak in the chromatogram overlaps with the signal of carbon dioxide from the air. However, since this is also the case for the calibration measurements, this is accounted for in the calculated release.

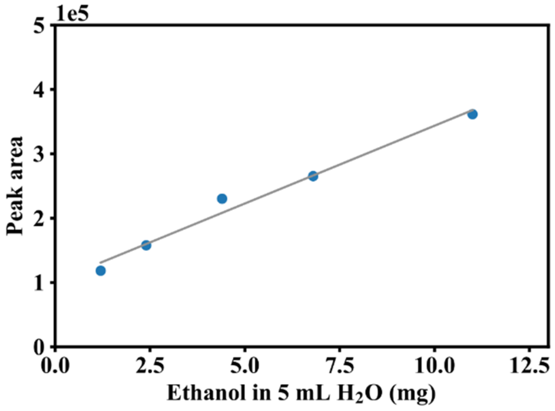

Figure S109. Calibration curve of ethanol measured at 30 °C headspace GC-MS oven temperature.

Hexanol release from co-polymers 2-DVE and 2-EGVE

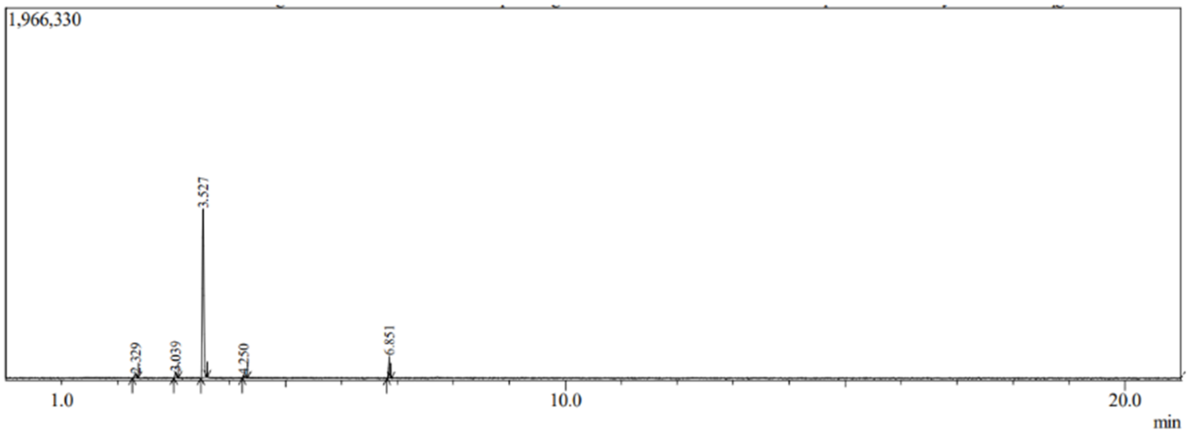

Peak Report TIC

Peak#	R.Time	Area	Area%	Height	Height%	Name	Base m/z	Base Int.
1	2.329	47392	2.72	20039	1.88		57.10	11686
2	3.039	45512	2.61	27771	2.60	Toluene	91.10	11546
3	3.527	1452067	83.35	891922	83.47	Acetic acid, butyl ester	56.10	378143
4	4.250	34598	1.99	13658	1.28	1-Hexanol	56.10	5649
5	6.851	162496	9.33	115113	10.77	Ether, 2-ethylhexyl tert-butyl	57.10	54929
		1742065	100.00	1068503	100.00			

(a)

Peak#:4 R.Time:4.250(Scan#:851)
MassPeaks:249
RawMode:Averaged 4.245-4.255(850-852)
BG Mode:Calc. from Peak Group 1 - Event 1 Scan

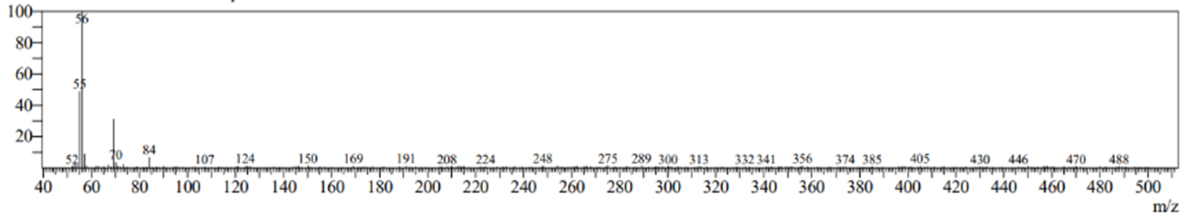

(b)

Figure S110. Hexanol release from **2-DVE** co-polymer measured by headspace GC-MS after 10 days mixing in water. (a) Headspace GC chromatogram. (b) MS diffraction of hexanol.

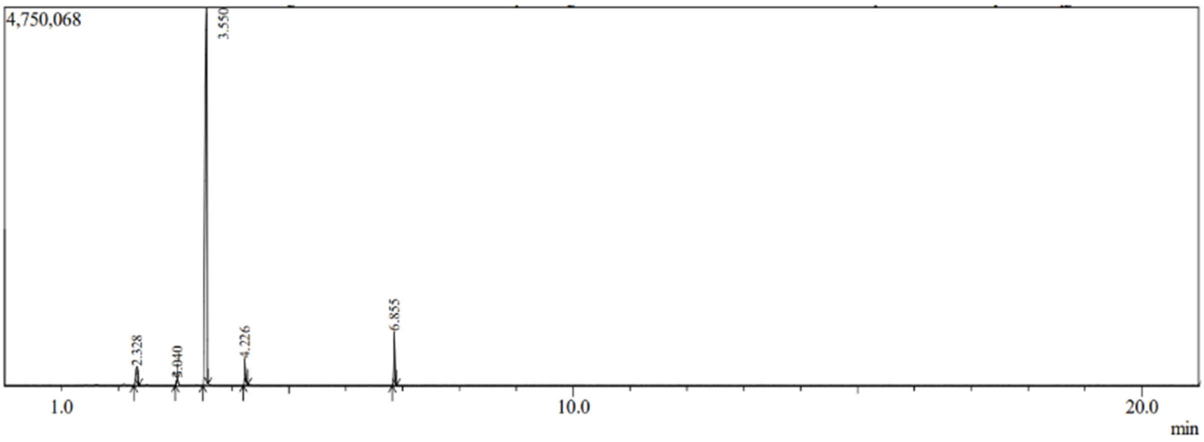

Peak Report TIC

Peak#	R.Time	Area	Area%	Height	Height%	Name	Base m/z	Base Int.
1	2.328	578196	4.17	230008	3.85	Pentane, 2,2,4-trimethyl-	57.05	144080
2	3.040	14761	0.11	0	0.00		70.10	79
3	3.550	11843317	85.39	4725301	79.08	Acetic acid, butyl ester	56.10	2035268
4	4.226	480216	3.46	335342	5.61	1-Hexanol	56.10	143099
5	6.855	953537	6.87	685065	11.46	Ether, 2-ethylhexyl tert-butyl	57.10	325757
		13870027	100.00	5975716	100.00			

(a)

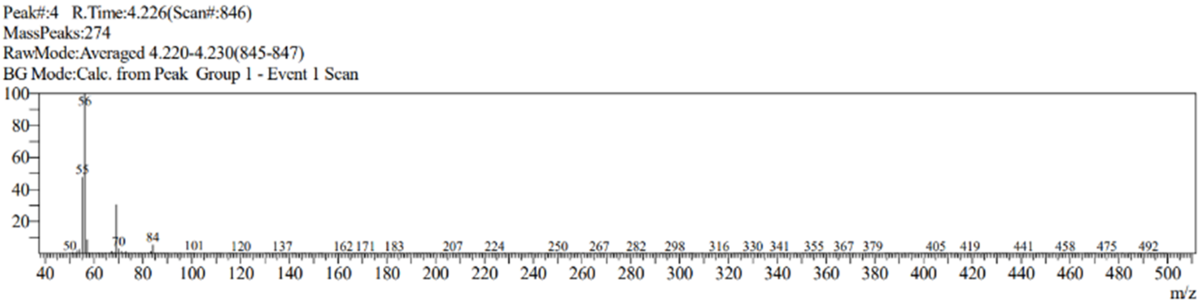

(b)

Figure S111. Hexanol release from **2-EGVE** co-polymer measured by headspace GC-MS after 10 days mixing in water. (a) Headspace GC chromatogram. (b) MS diffraction of hexanol.

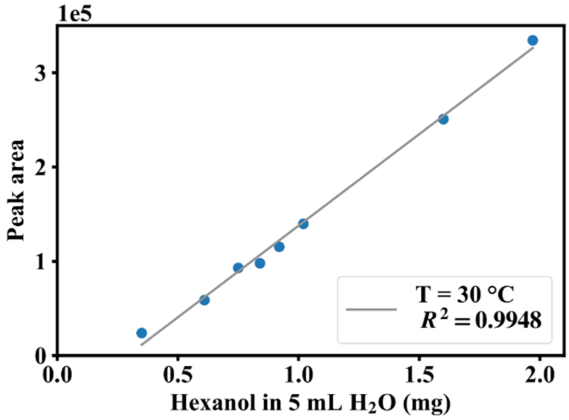

Figure S112. Calibration curve of hexanol measured at 30 °C headspace GC-MS oven temperature.

Dihydrocitronellol release from co-polymer 5-EGVE

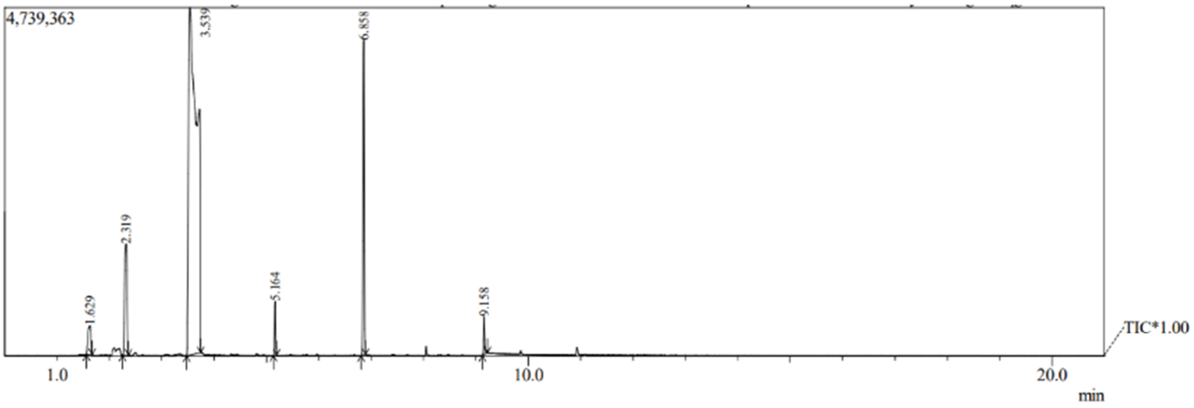

Peak Report TIC

Peak#	R.Time	Area	Area%	Height	Height%	Name	Base m/z	Base Int.
1	1.629	1592167	2.43	398142	3.27	2-Propanol, 2-methyl-	59.10	320737
2	2.319	4822797	7.37	1519893	12.49	Pentane, 2,2,4-trimethyl-	57.10	967243
3	3.539	50624047	77.40	4717724	38.76	Acetic acid, butyl ester	56.10	2013760
4	5.164	943058	1.44	740546	6.08	Octane, 2,6-dimethyl-	57.10	234920
5	6.858	6643972	10.16	4276044	35.13	Ether, 2-ethylhexyl tert-butyl	57.10	1918205
6	9.158	781854	1.20	519177	4.27	1-Octanol, 2,7-dimethyl-	56.10	71107
		65407895	100.00	12171526	100.00			

(a)

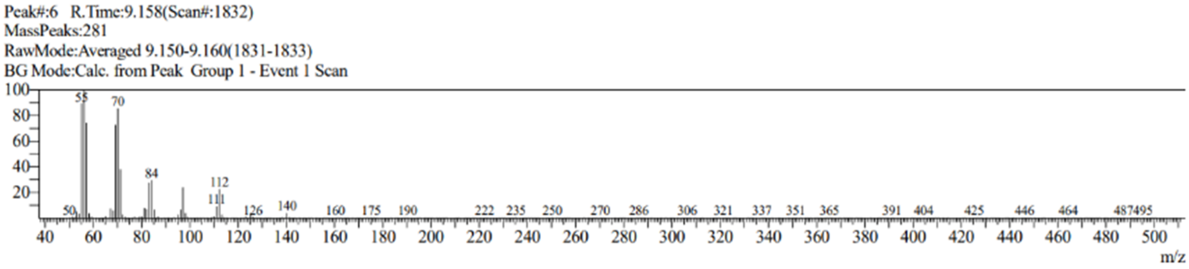

(b)

Figure S113. Dihydrocitronellol release from **5-EGVE** co-polymer measured by headspace GC-MS after 10 days mixing in water. (a) Headspace GC chromatogram. (b) MS diffraction of dihydrocitronellol.

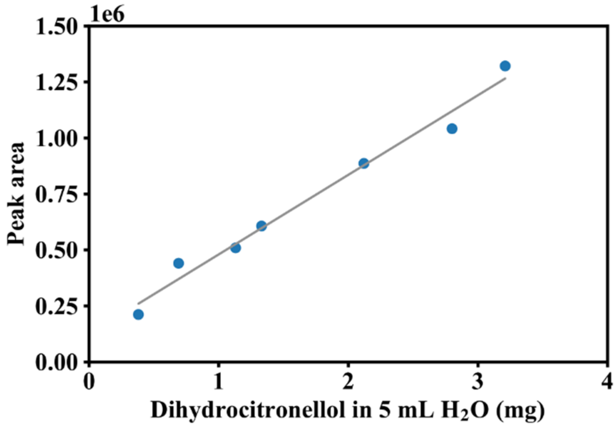

Figure S114. Calibration curve of dihydrocitronellol measured at 80 °C headspace GC-MS oven temperature.

Phenylethanol release from co-polymer 6-EGVE

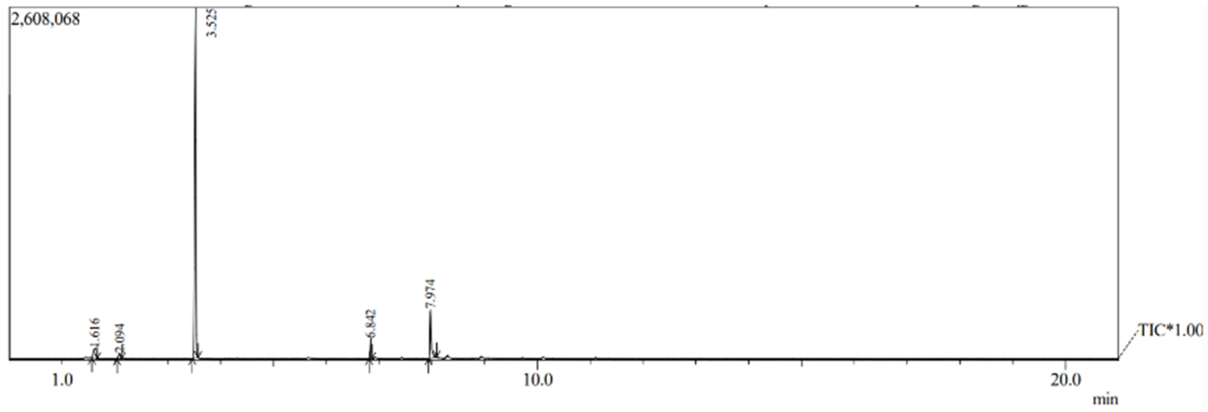

Peak Report TIC

Peak#	R.Time	Area	Area%	Height	Height%	Name	Base m/z	Base Int.
1	1.616	253231	4.25	73351	2.28	2-Propanol, 2-methyl-	59.10	55218
2	2.094	110044	1.85	37984	1.18	1,3-Dioxolane, 2-methyl-	73.05	23891
3	3.525	4736590	79.58	2592538	80.64	Acetic acid, butyl ester	56.10	1094827
4	6.842	197136	3.31	147451	4.59	Ether, 2-ethylhexyl tert-butyl	57.10	66487
5	7.974	654654	11.00	363496	11.31	Phenylethyl Alcohol	91.10	129506
		5951655	100.00	3214820	100.00			

(a)

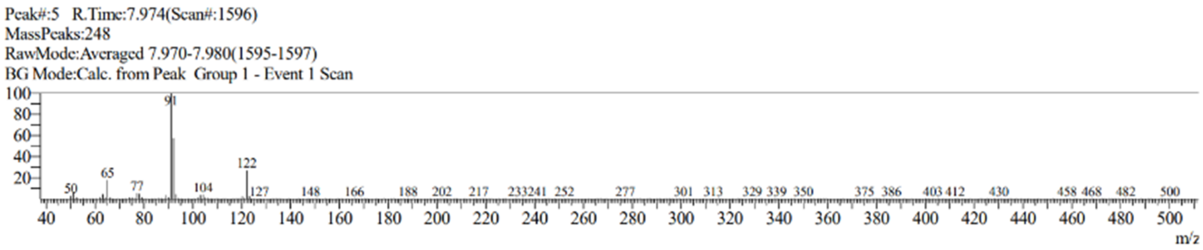

(b)

Figure S115. Phenylethanol release from **6-EGVE** co-polymer measured by headspace GC-MS after 10 days mixing in water. (a) Headspace GC chromatogram. (b) MS diffraction of phenylethanol.

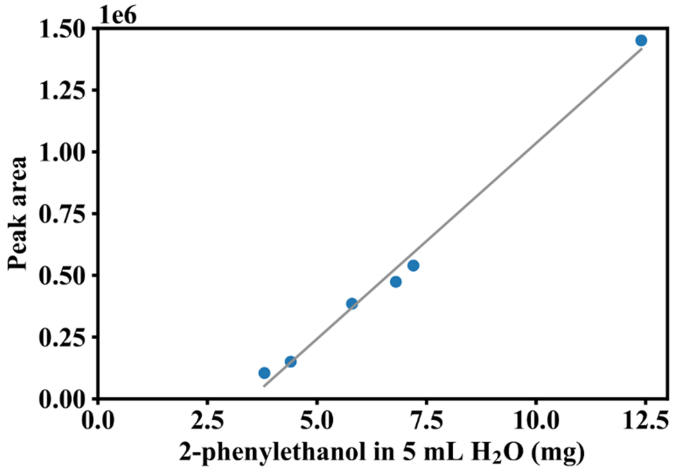

Figure S116. Calibration curve of phenylethanol measured at 80 °C headspace GC-MS oven temperature.

Menthol release from co-polymer 7-EGVE

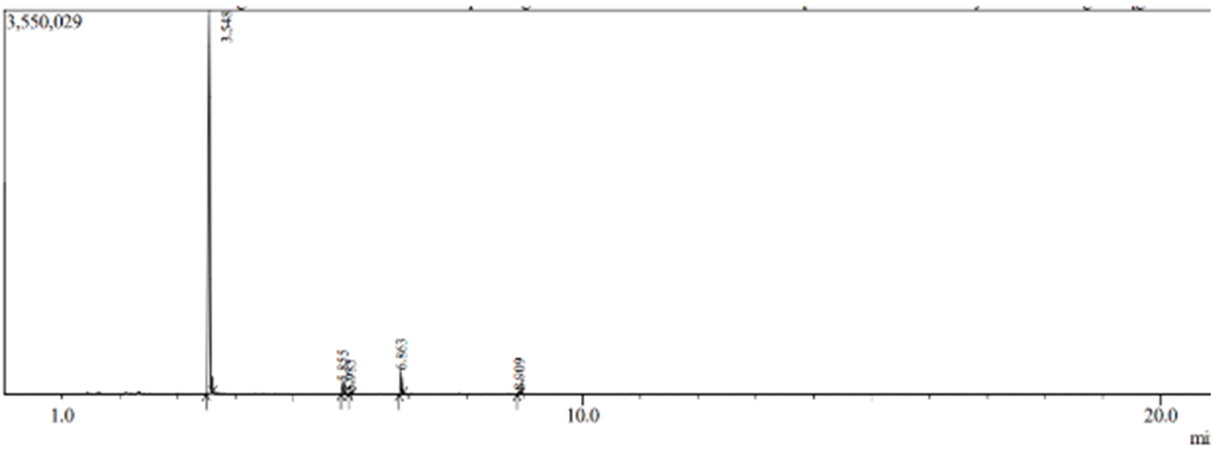

Peak Report TIC

Peak#	R.Time	Area	Area%	Height	Height%	Name	Base m/z	Base Int.
1	3.548	6925939	93.12	3534984	90.91	Acetic acid, butyl ester	56.10	1438804
2	5.855	148983	2.00	103236	2.65	Cyclohexane, 1-methyl-4-(1-methylethyl)-, cis	55.10	26686
3	5.985	18550	0.25	15040	0.39		95.15	2914
4	6.863	293175	3.94	211592	5.44	Ether, 2-ethylhexyl tert-butyl	57.10	99318
5	8.909	50894	0.68	23758	0.61	L-Menthyl chloroformate	95.15	2623
		7437541	100.00	3888610	100.00			

(a)

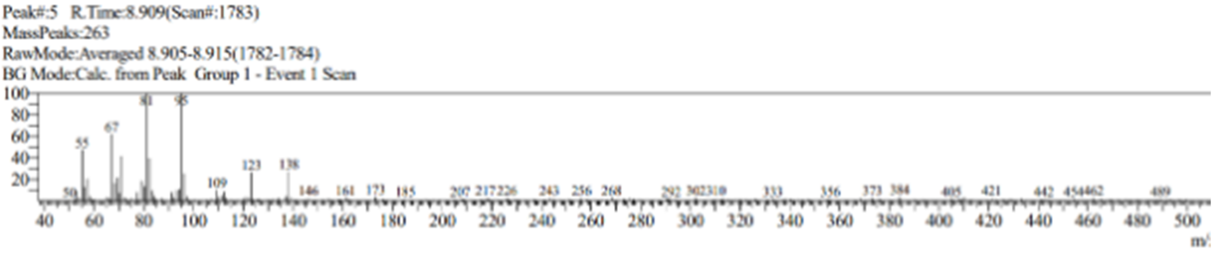

(b)

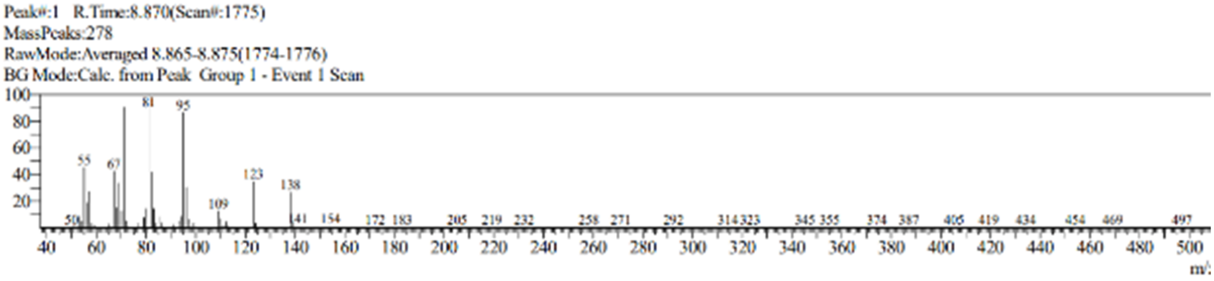

(c)

Figure S117. Menthol release from **7-EGVE** co-polymer measured by headspace GC-MS after 10 days mixing in water. The similarity search indicates a different compound than menthol. However, comparison with a reference measurement of menthol showed a very high similarity. (a) Headspace GC chromatogram. (b) MS diffraction of released menthol. (c) Reference MS diffraction spectrum of menthol.

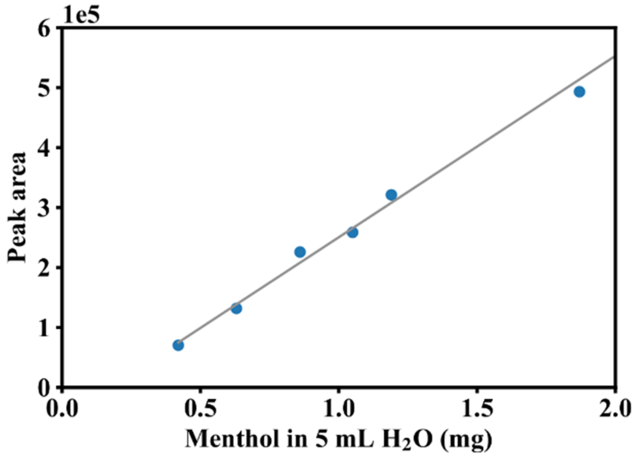

Figure S118. Calibration curve of menthol measured at 40 °C headspace GC-MS oven temperature.

DSC of polymers before and after release

In order to check the properties of the polymers before and after release, 2 different co-polymers were investigated (1-EGVE and 1-DVE). We reasoned that release on these co-polymers should have the largest impact on polymer properties, as they had the most alcohol release. Unfortunately, due to solubility issues we could not measure GPC of the polymers post-release. Therefore, we only checked the glass transition temperature of the co-polymers before and after release in 100 mol% HCl aqueous solutions, and is shown in Table S11. For 1-DVE (where 90% release took place), we see no significant change in the T_g before and after release. For 1-EGVE, where more than 80% release was observed, we see a significant increase in the glass transition temperature.

Table S13. Glass transition temperatures (T_g) of 2-DVE and 2-EGVE polymers before and after release in 100 mol% HCl in water (5 mL) for 10 days.

Co-polymer	Moment of measurement	T_g (°C)
1-DVE	Before release	-12
	After release	-10
1-EGVE	Before release	17
	After release	44

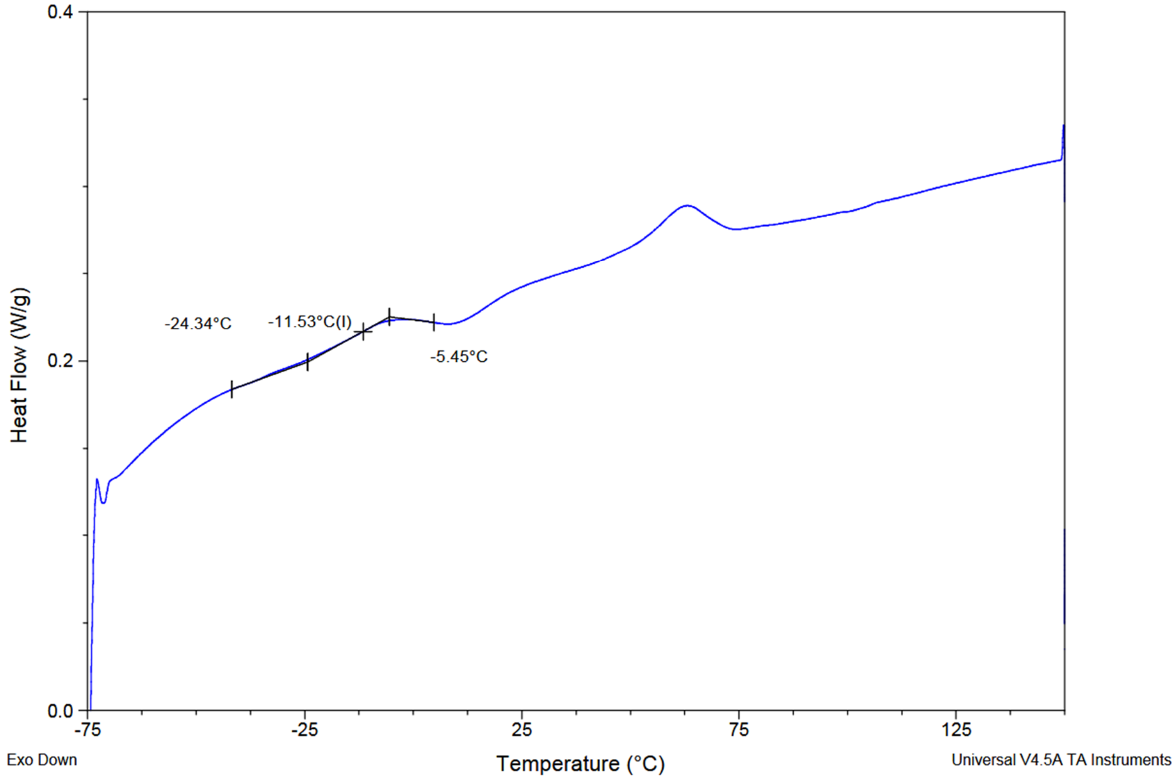

Figure S119. DSC of co-polymer **1-DVE** before release.

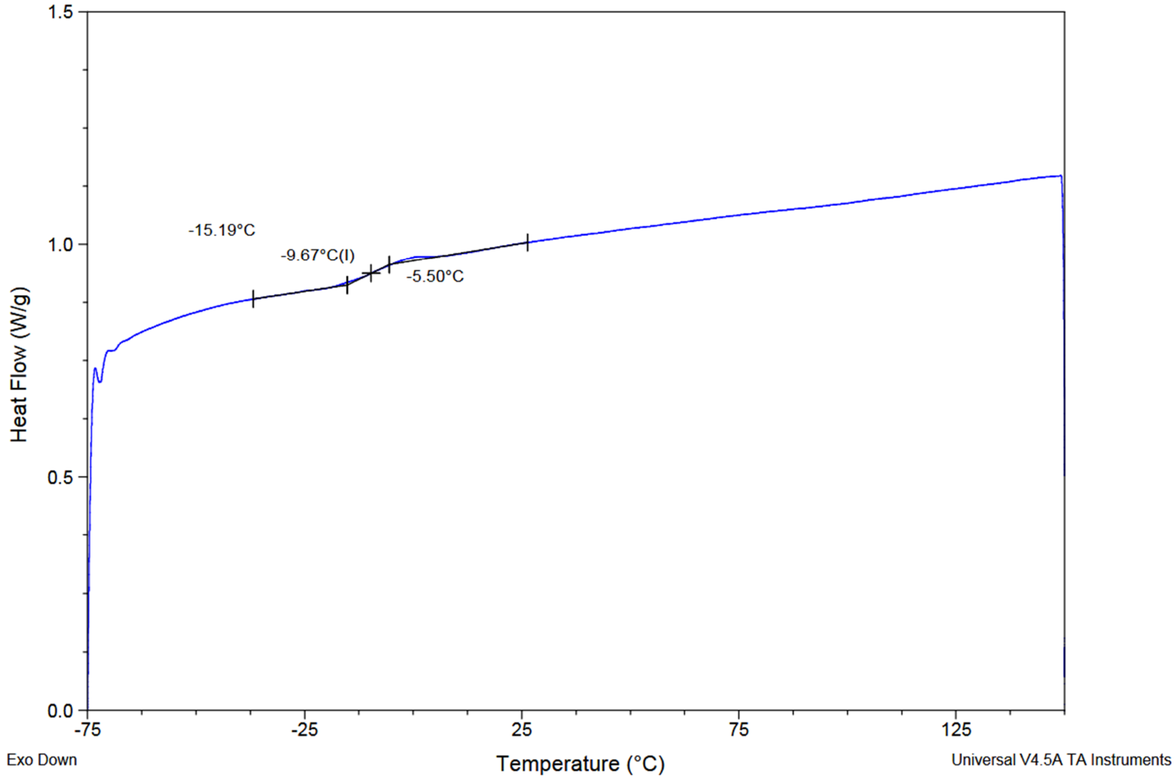

Figure S120. DSC of co-polymer **1-DVE** after release in 100 mol% HCl in water (5 mL) for 10 days.

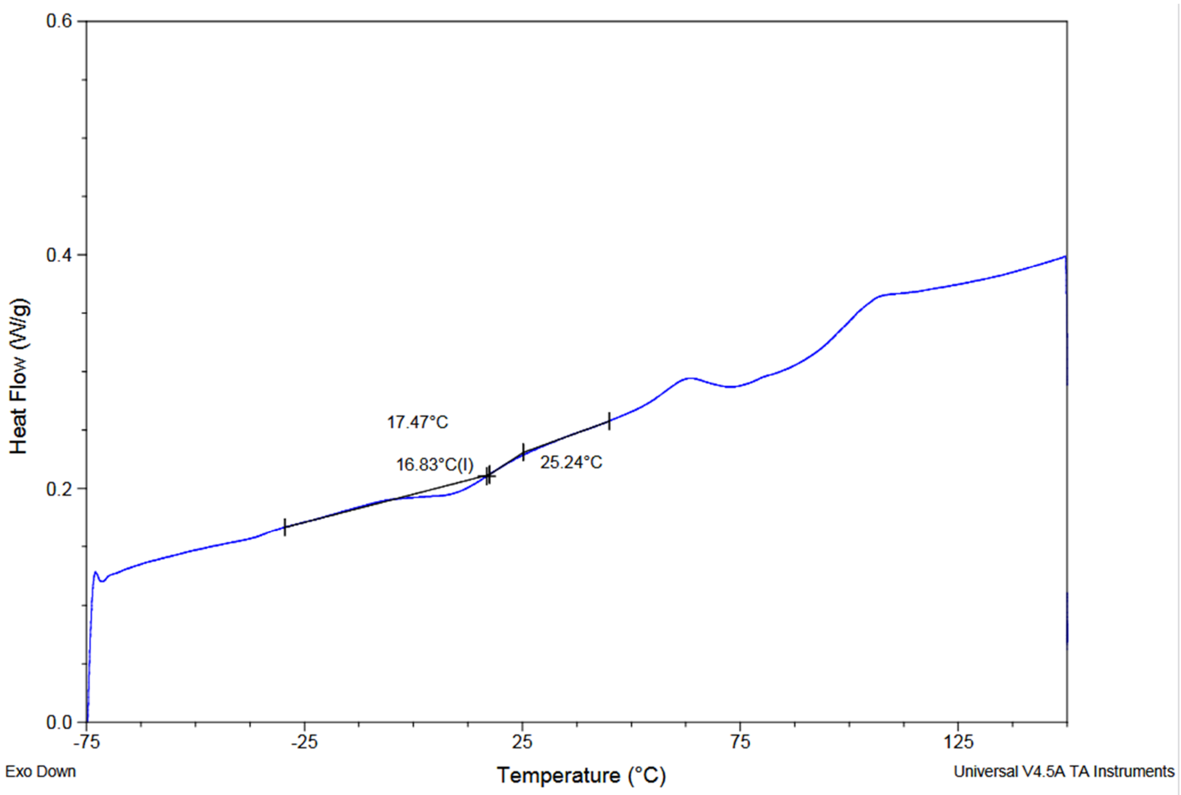

Figure S121. DSC of co-polymer **1-EGVE** before release.

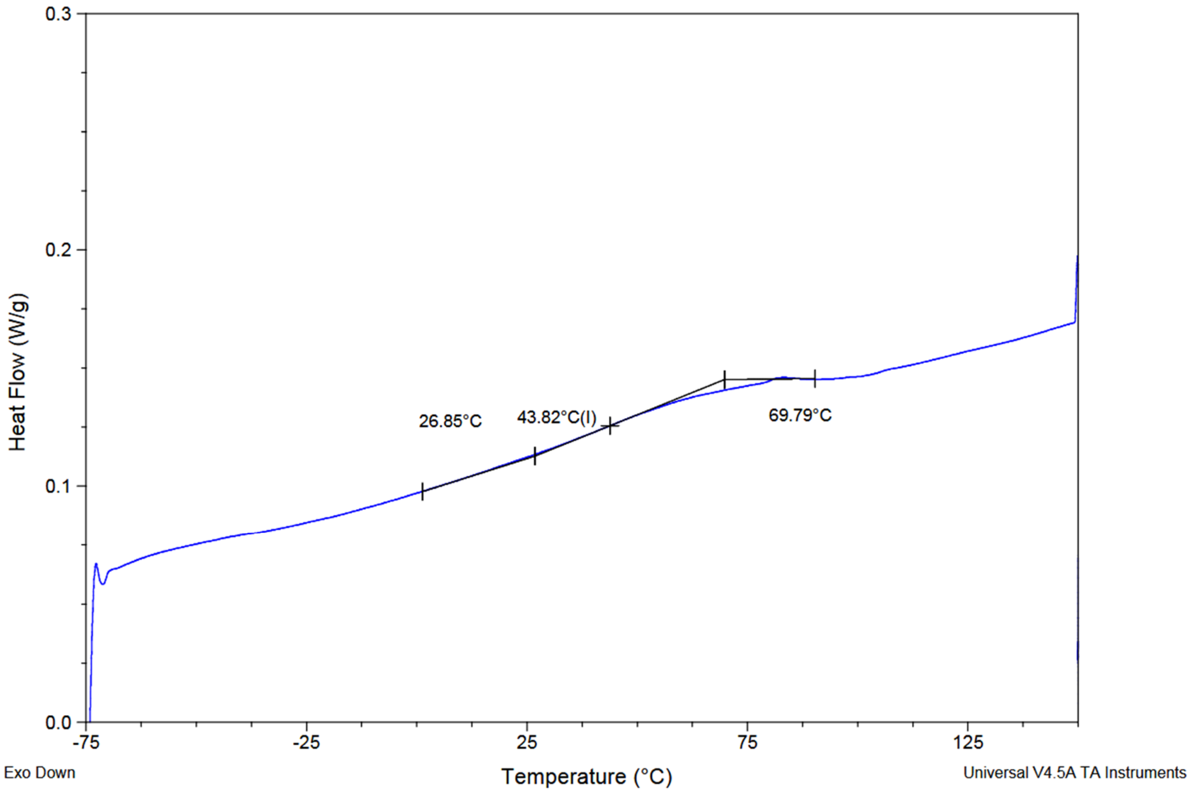

Figure S122. DSC of co-polymer **1-EGVE** after release in 100 mol% HCl in water (5 mL) for 10 days.

References

[28] J. G. H. Hermens, M. L. Lepage, A. Kloekhorst, E. Keller, R. Bloem, M. Meijer, B. L. Feringa, Development of a modular photoreactor for the upscaling of continuous flow photochemistry, *React. Chem. Eng.*, **2022**, *7*, 2280-2284.

[36] T. Freese, J. P. Kaniraj, B. L. Feringa, A. Jensma, N. Elders, K. J. van den Berg, P139663NL00, **2025**.

[37] C. Bax, S. Sironi, L. Capelli, How Can Odors Be Measured? An Overview Of Methods and Their Applications, *Atmosphere,* **2020**, *11*, 92.

[38] C. A. De March, S. Ryu, G. Sicard, C. Moon, J. Golebiowski, Structure–odour relationships reviewed in the postgenomic era, *Flavour and Fragrance Journal,* **2015**, *30(5)*, 342-361.

[39] J. E. Cometto-Muñiz, M. H. Abraham, Human olfactory detection of homologues n-alcohols measured via concentration-response functions, *Pharmacology,* **2008**, *89*, 279-291.

[40] M. Zarzo, Effect of Functional Group and Carbon Chain Length on the Odor Detection Threshold of Aliphatic Compounds, *Sensors,* **2012**, *12,* 4105-4112.

[41] G. Leonardos, D. Kendall, N. Barnard, Odor Threshold Determinations Of 53 Odorant Chemicals, *Journal of the Air Pollution Control Association,* **1969**, *19(2),* 91-95.

[42] M. Lapuerta, J. P. Hernández, J. Agudelo, An equation for the estimation of alcohol-air diffusion coefficients for modelling evaporation losses in fuel systems, *Applied Thermal Engineering,* **2014**, 73(1), 539-548.
